# Supplementary figures and images for: Music-induced emotion flow modeling by ENMI Network (part 2 of 2)
Source: PLoS One. 2024 Oct 21;19(10):e0297712. doi: 10.1371/journal.pone.0297712 (PMC11493256; doi:10.1371/journal.pone.0297712)

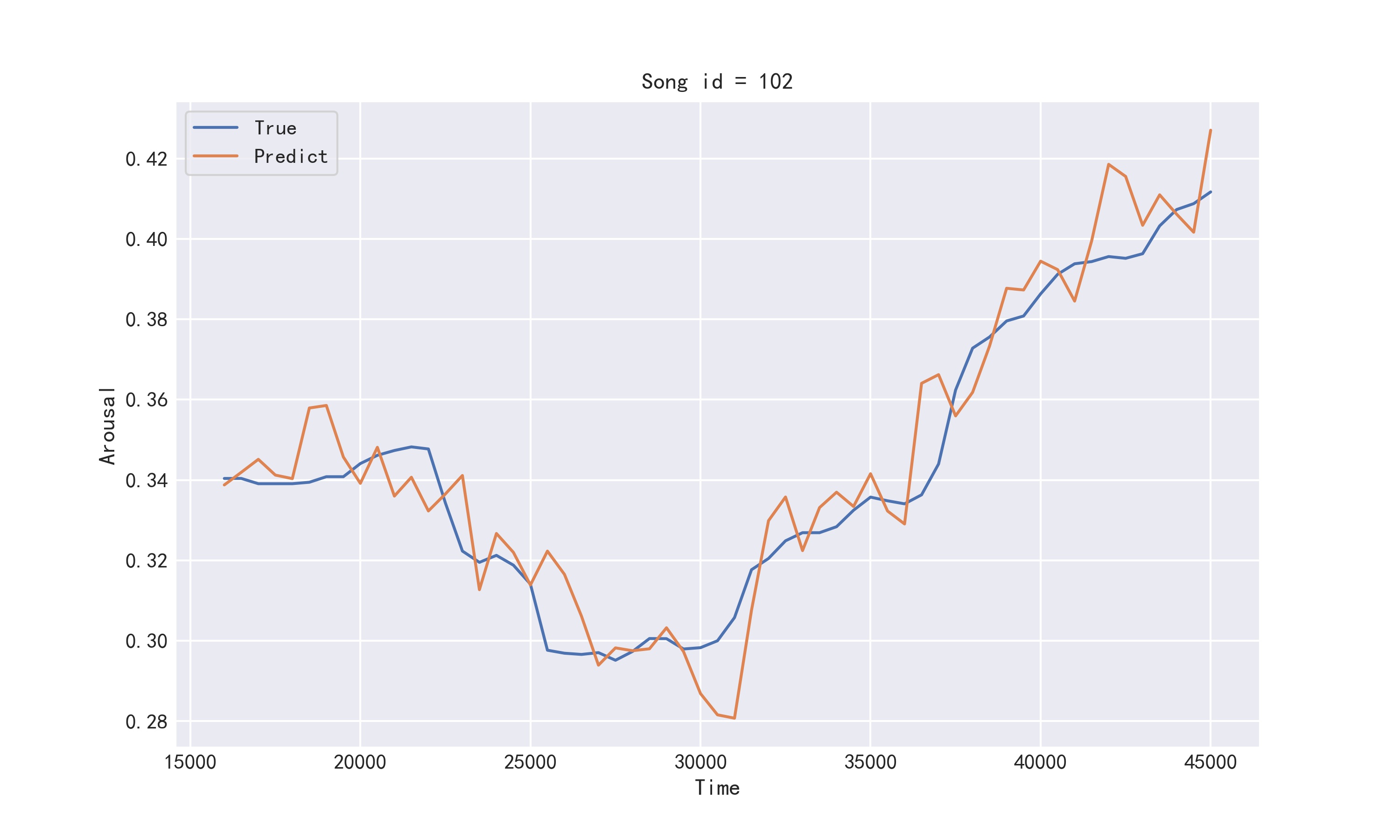

Supplement: S5 File — (ZIP) [file pone.0297712.s005.zip › All prediction results/prediction picture results(Emomusic_75)/song_id_102.jpg]

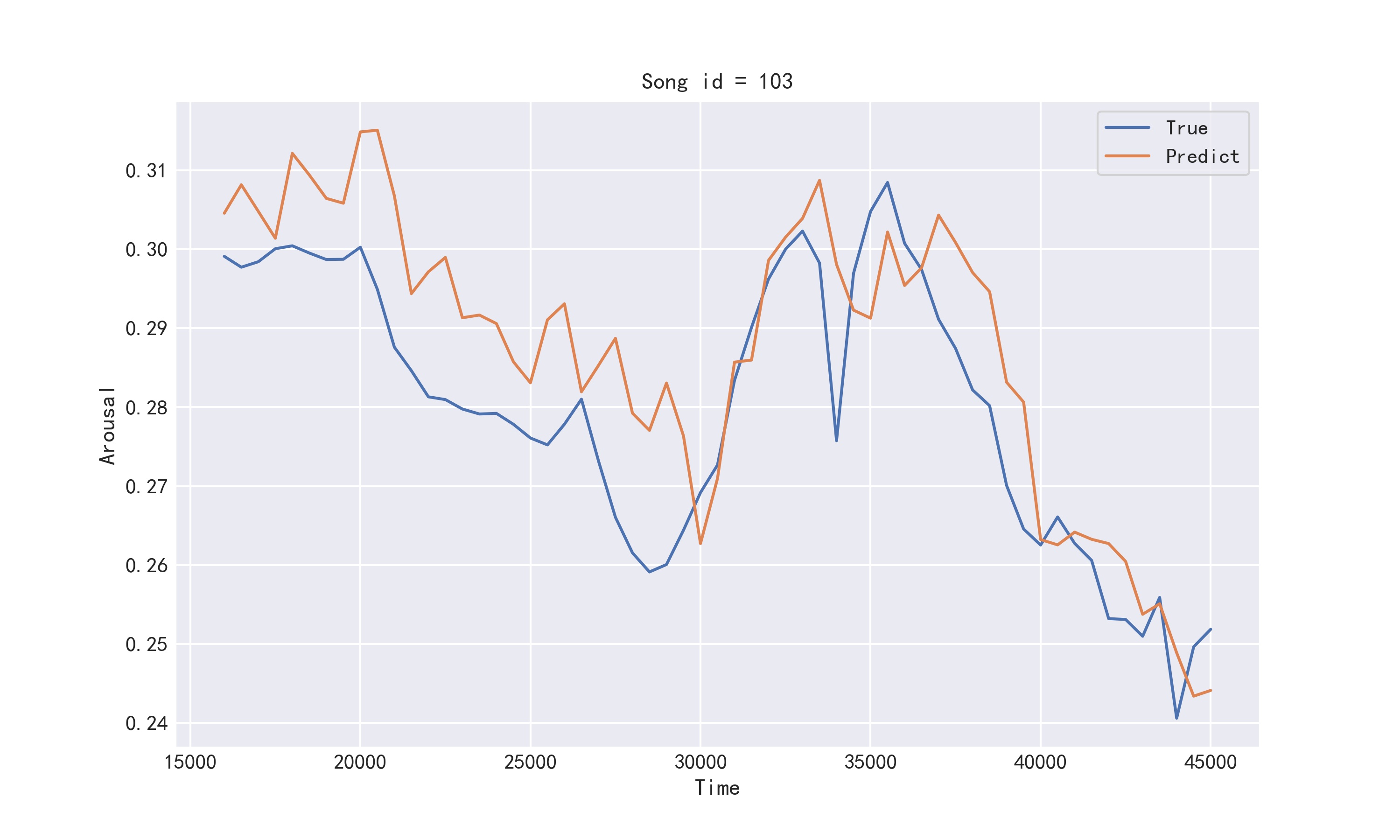

Supplement: S5 File — (ZIP) [file pone.0297712.s005.zip › All prediction results/prediction picture results(Emomusic_75)/song_id_103.jpg]

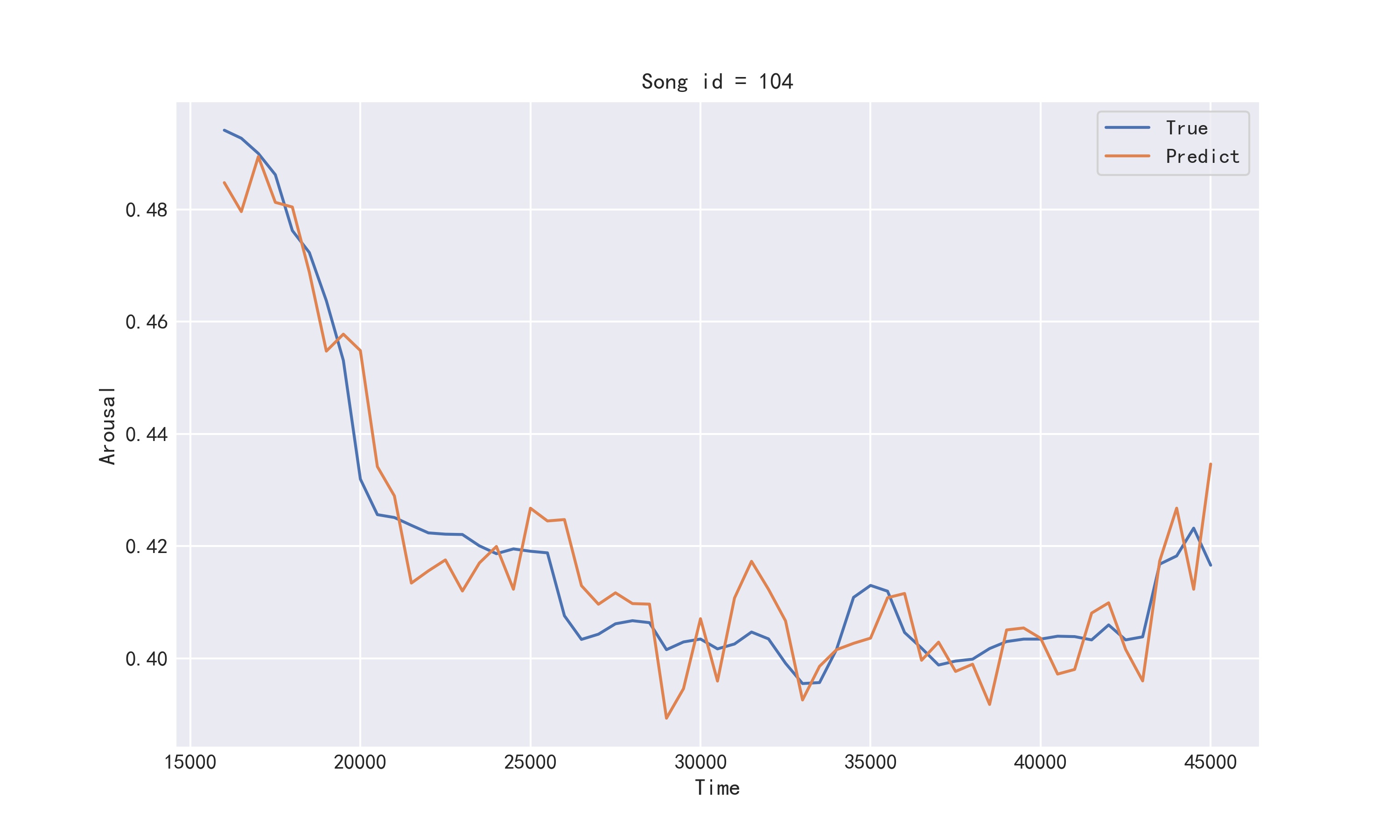

Supplement: S5 File — (ZIP) [file pone.0297712.s005.zip › All prediction results/prediction picture results(Emomusic_75)/song_id_104.jpg]

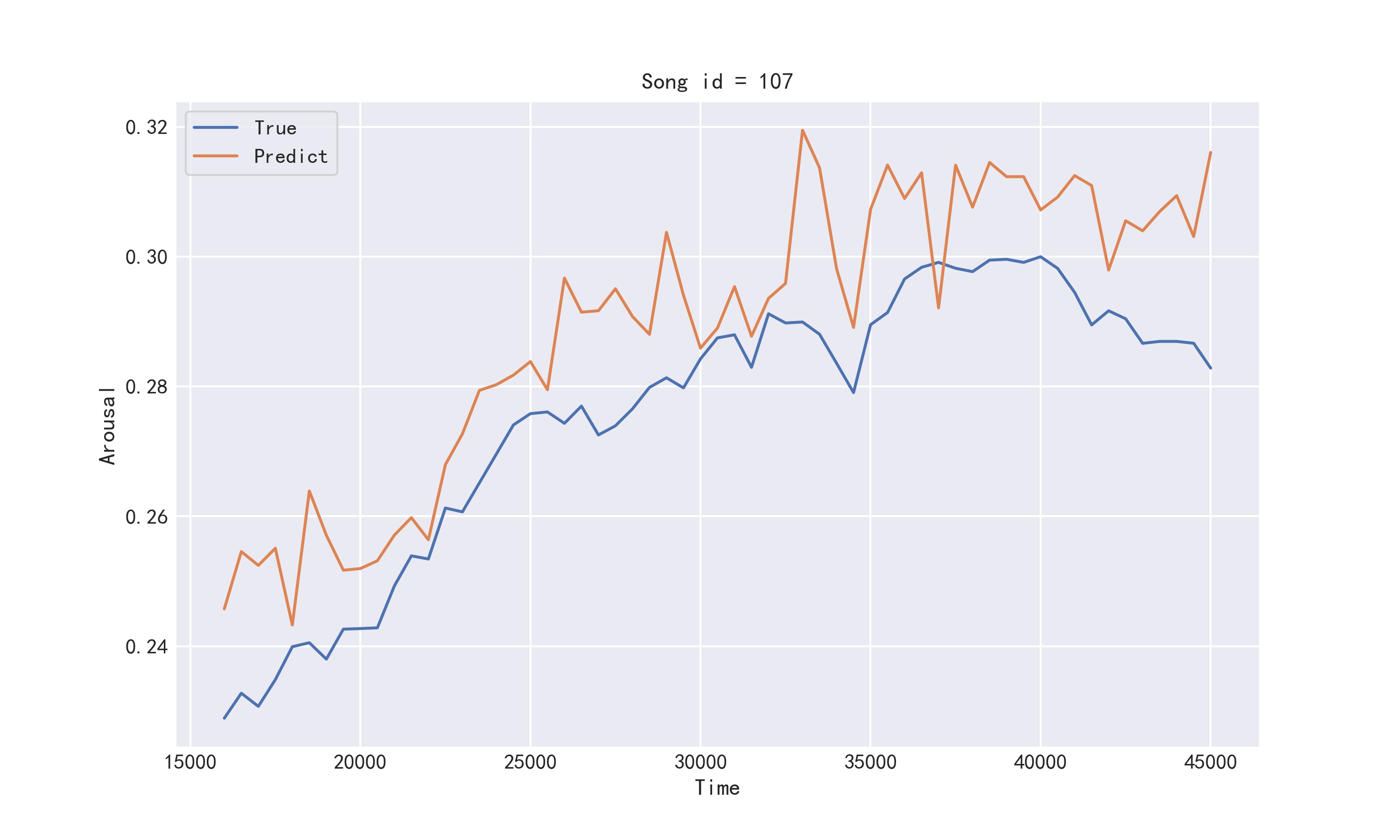

Supplement: S5 File — (ZIP) [file pone.0297712.s005.zip › All prediction results/prediction picture results(Emomusic_75)/song_id_107.jpg]

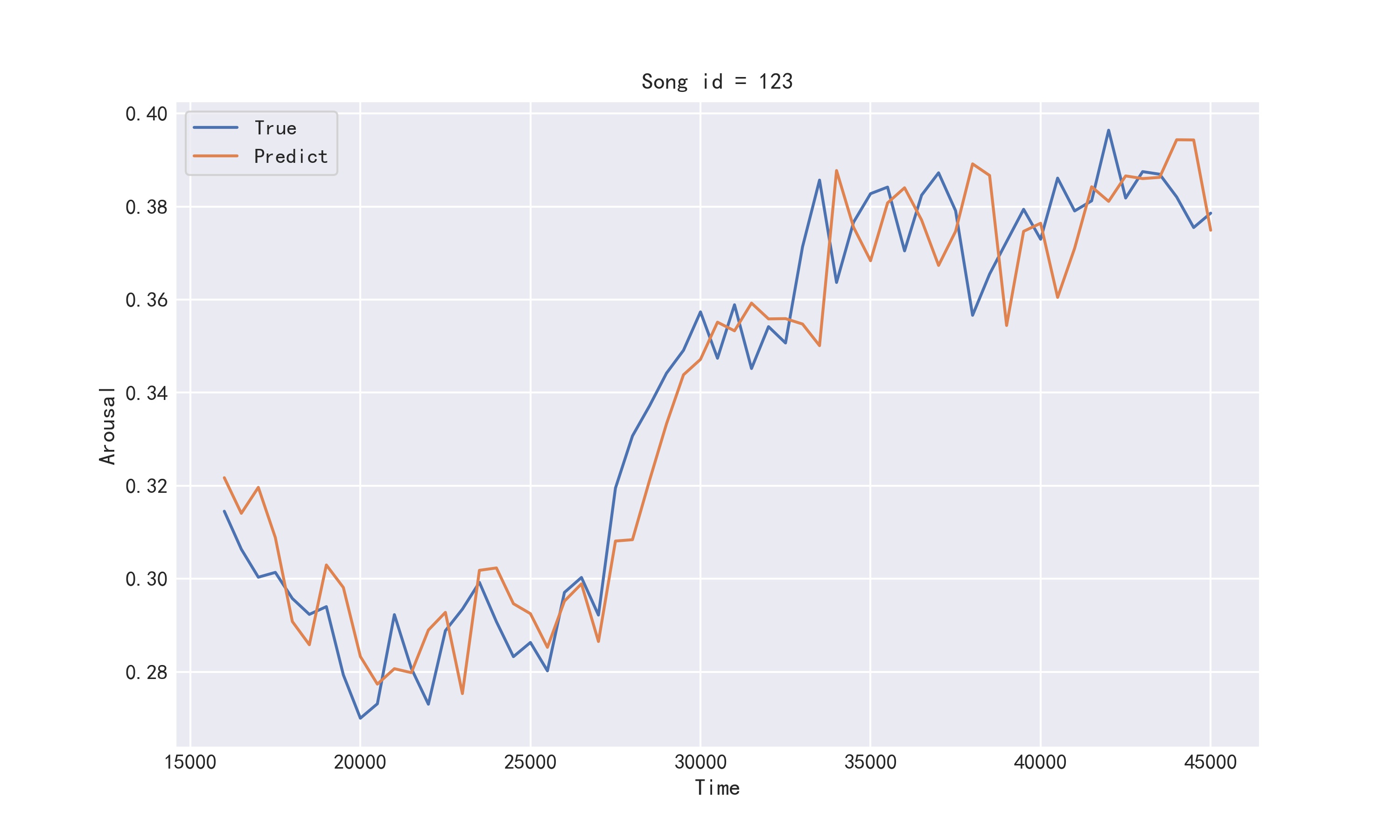

Supplement: S5 File — (ZIP) [file pone.0297712.s005.zip › All prediction results/prediction picture results(Emomusic_75)/song_id_123.jpg]

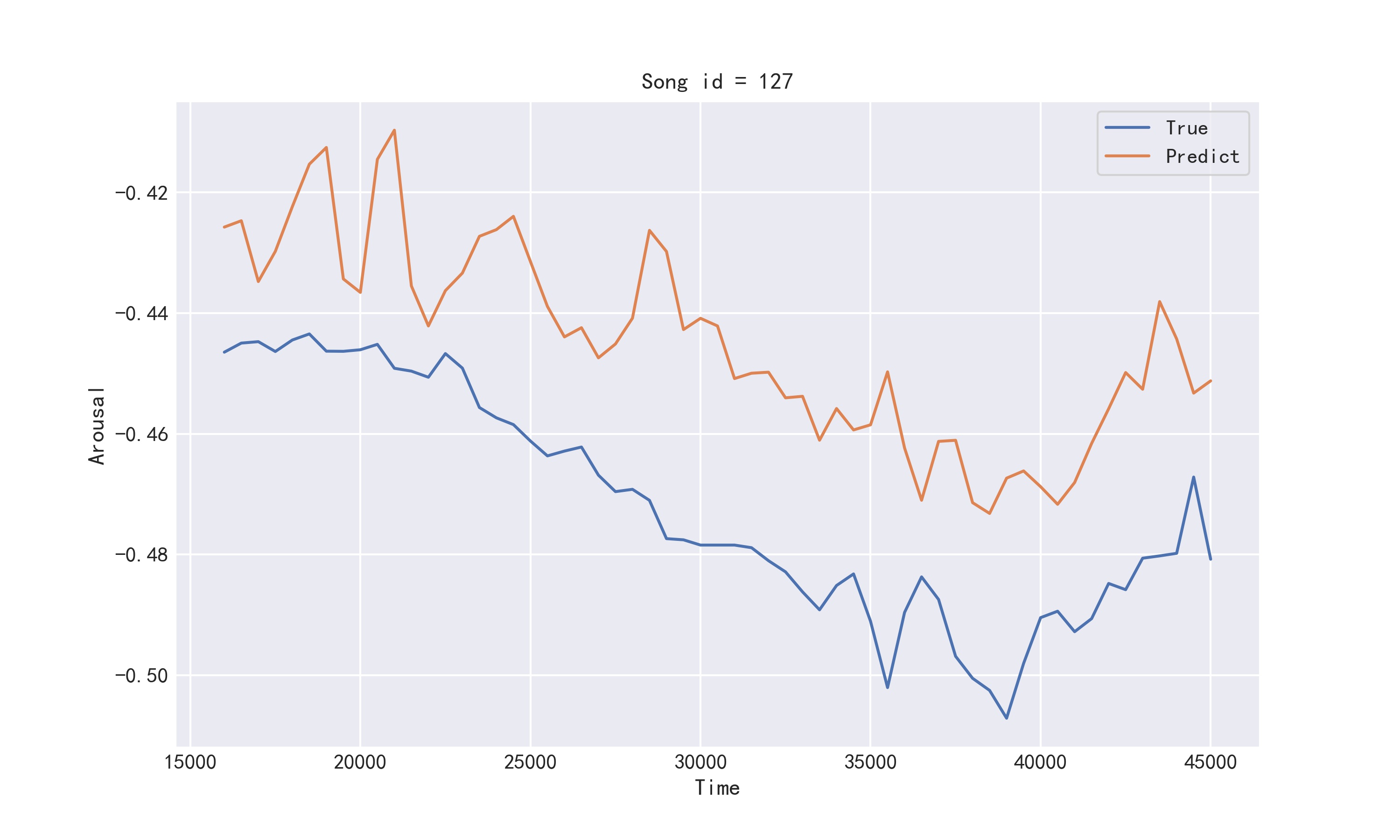

Supplement: S5 File — (ZIP) [file pone.0297712.s005.zip › All prediction results/prediction picture results(Emomusic_75)/song_id_127.jpg]

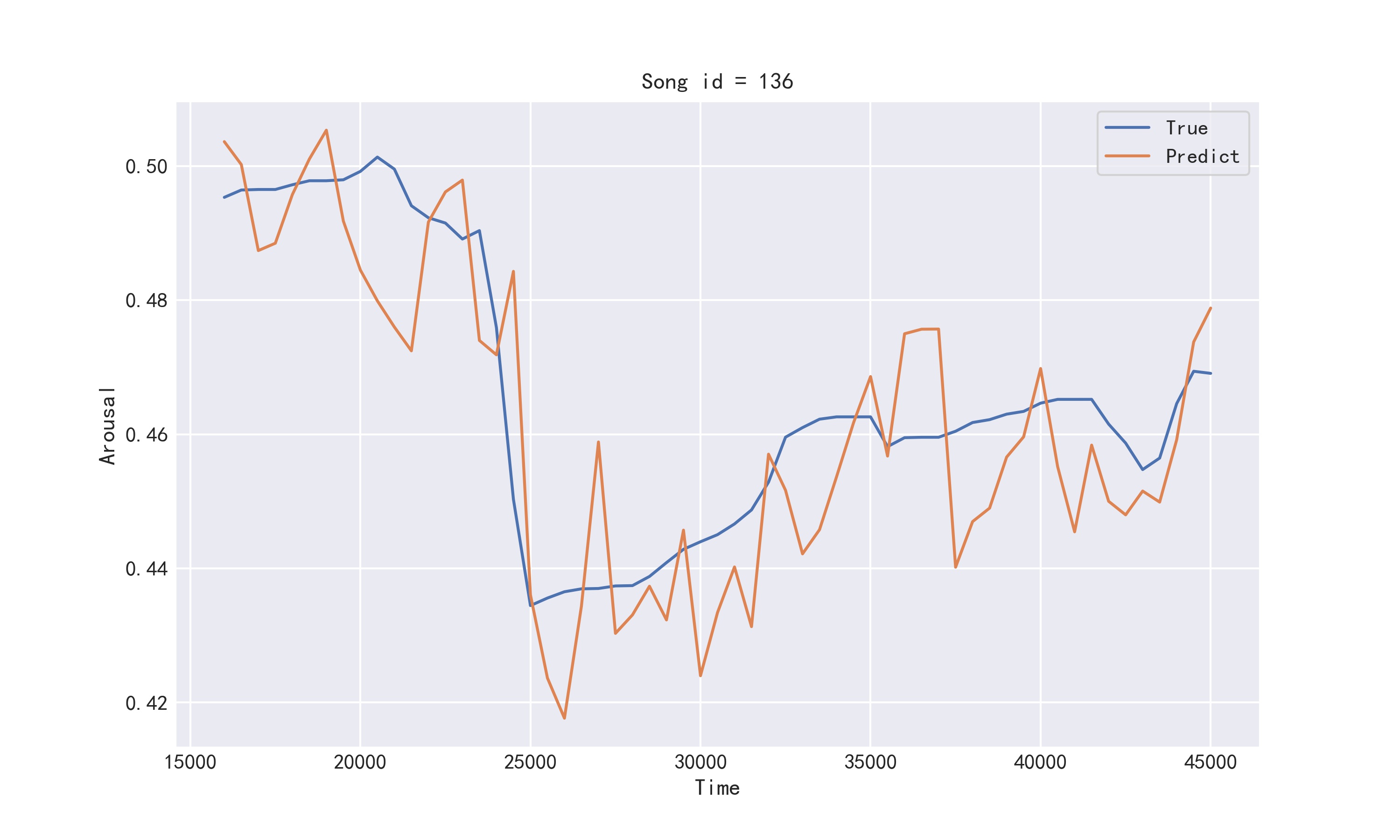

Supplement: S5 File — (ZIP) [file pone.0297712.s005.zip › All prediction results/prediction picture results(Emomusic_75)/song_id_136.jpg]

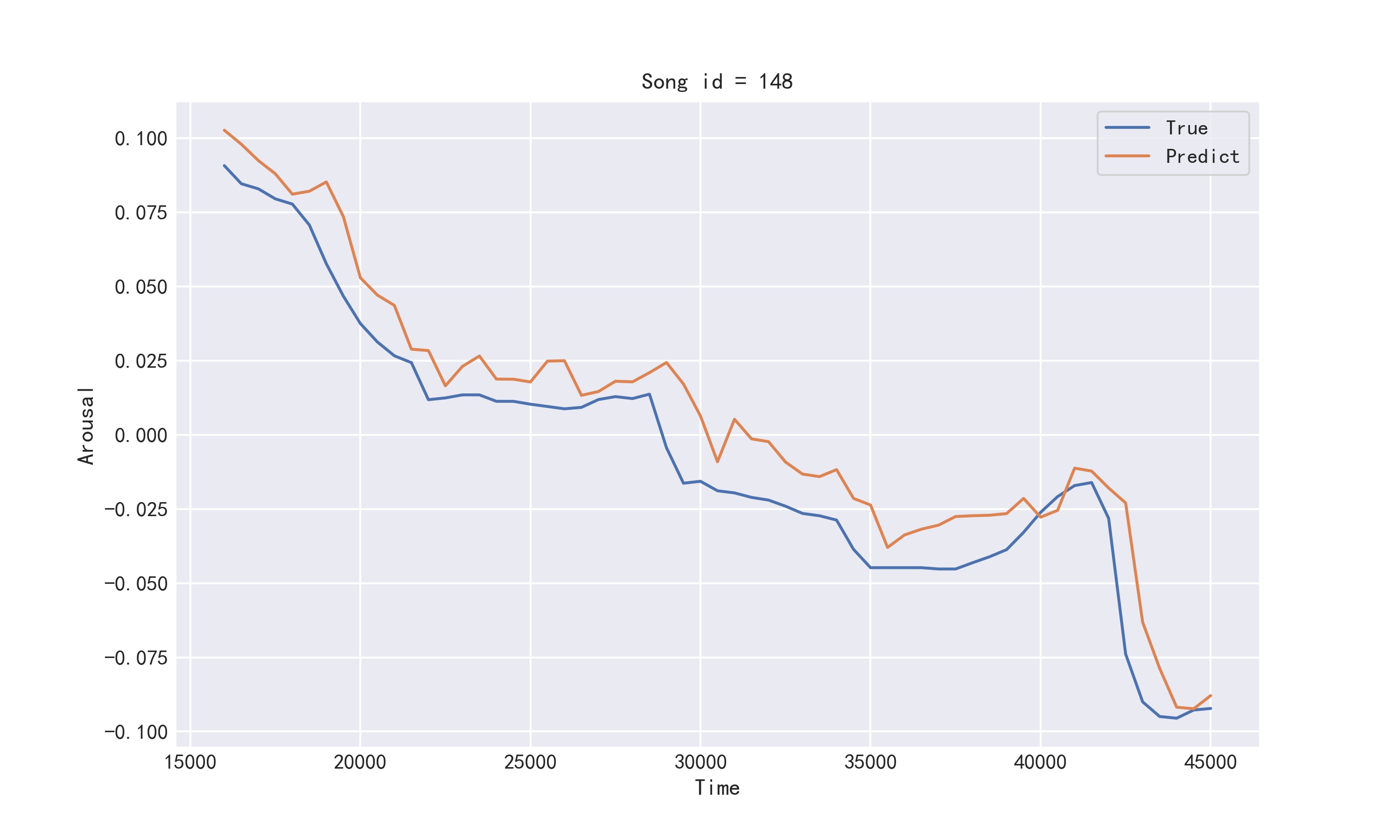

Supplement: S5 File — (ZIP) [file pone.0297712.s005.zip › All prediction results/prediction picture results(Emomusic_75)/song_id_148.jpg]

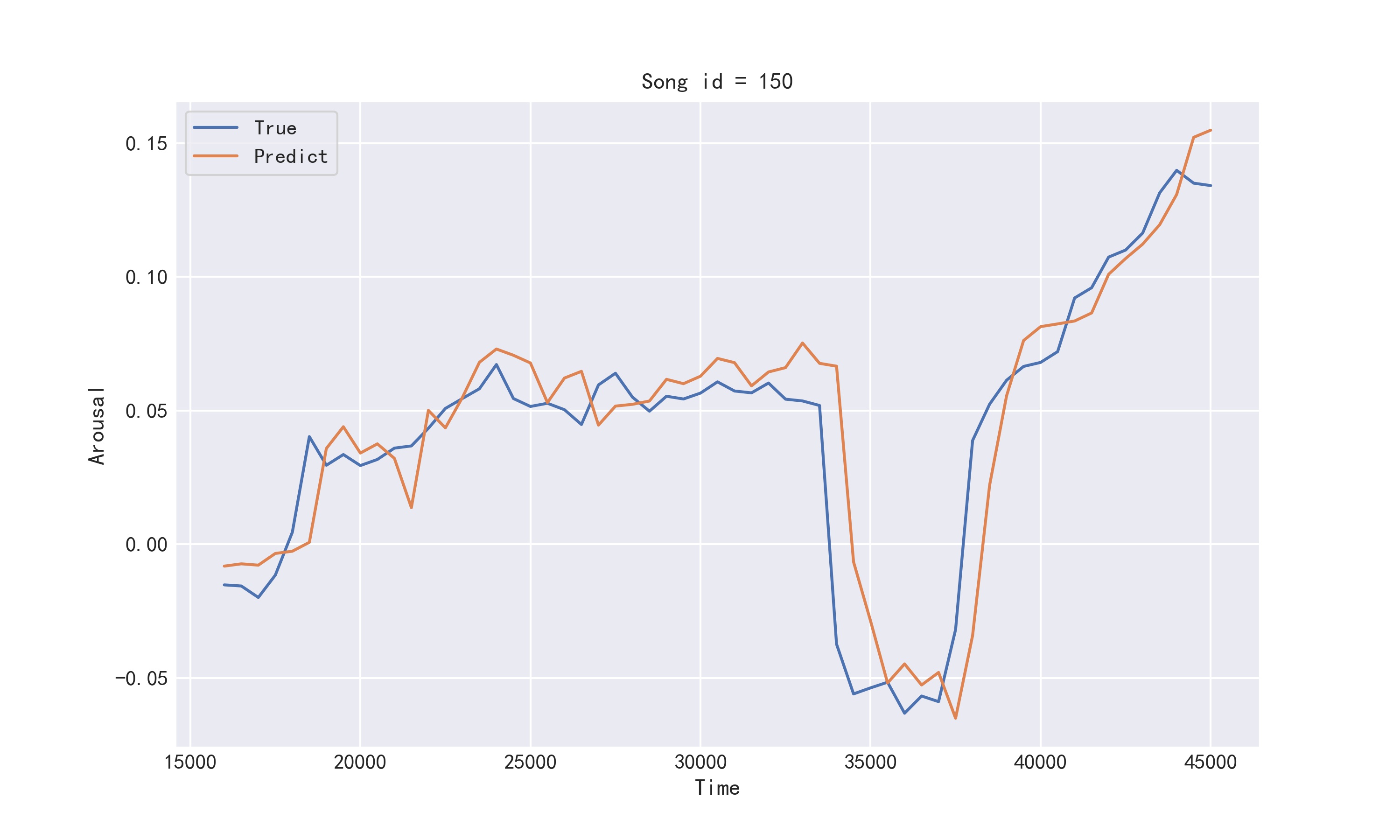

Supplement: S5 File — (ZIP) [file pone.0297712.s005.zip › All prediction results/prediction picture results(Emomusic_75)/song_id_150.jpg]

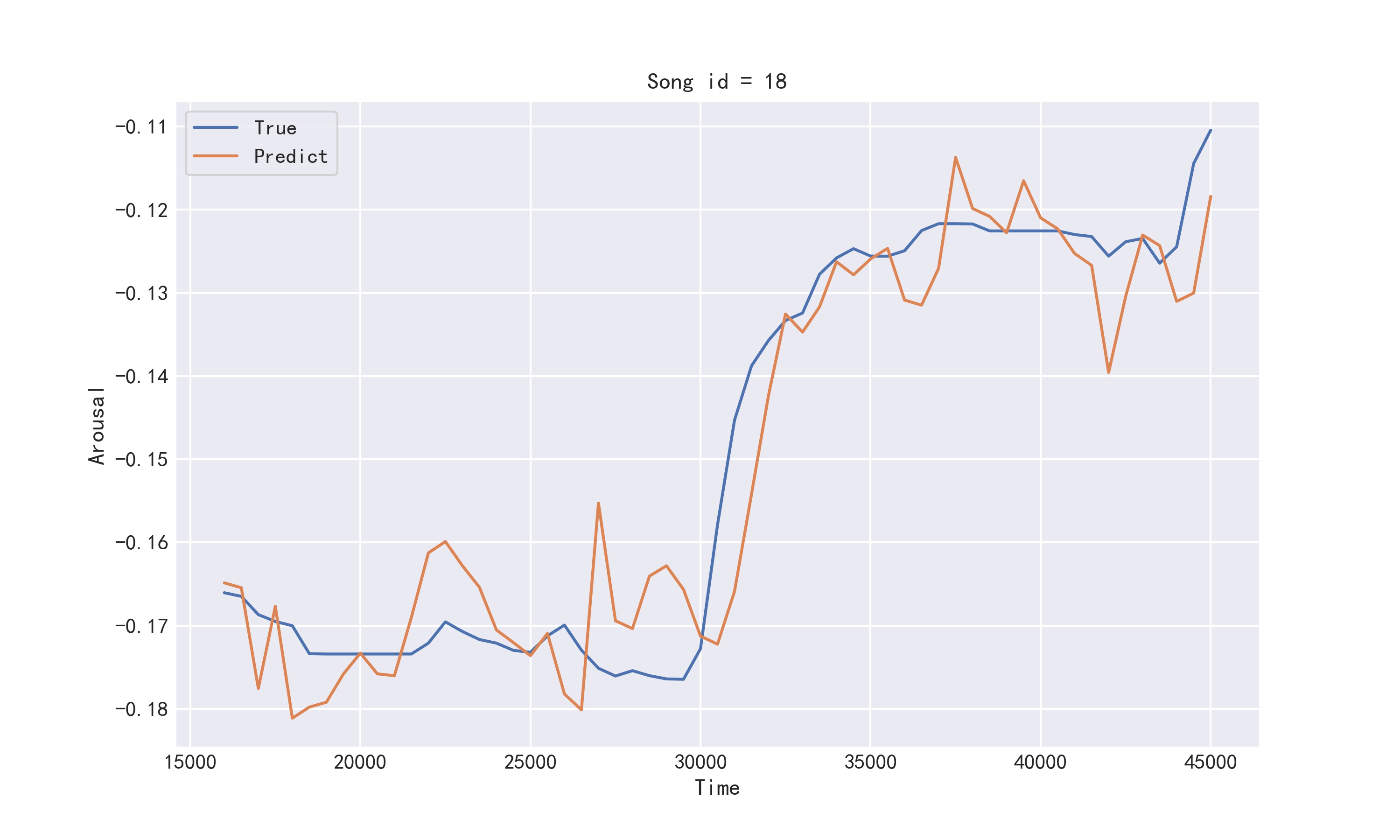

Supplement: S5 File — (ZIP) [file pone.0297712.s005.zip › All prediction results/prediction picture results(Emomusic_75)/song_id_18.jpg]

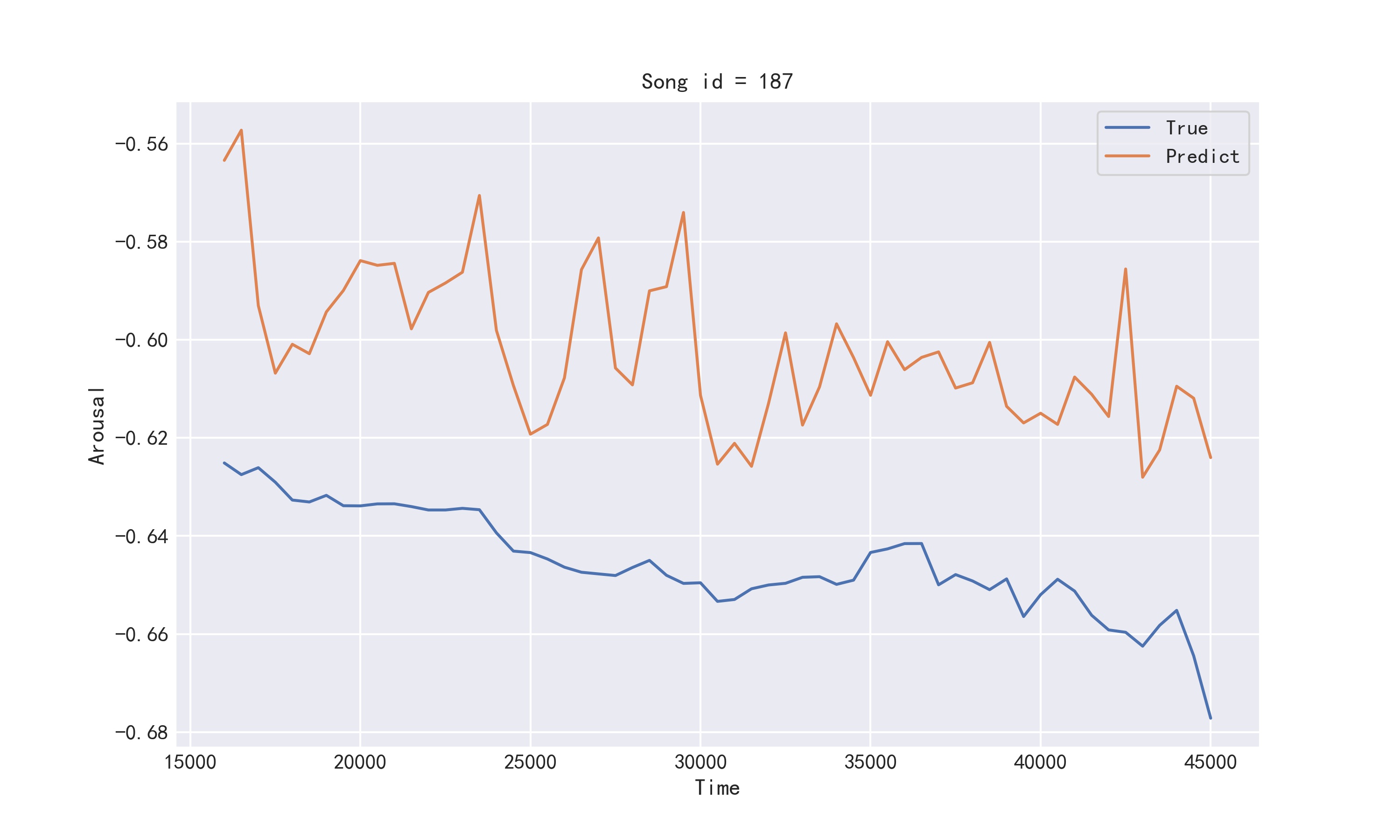

Supplement: S5 File — (ZIP) [file pone.0297712.s005.zip › All prediction results/prediction picture results(Emomusic_75)/song_id_187.jpg]

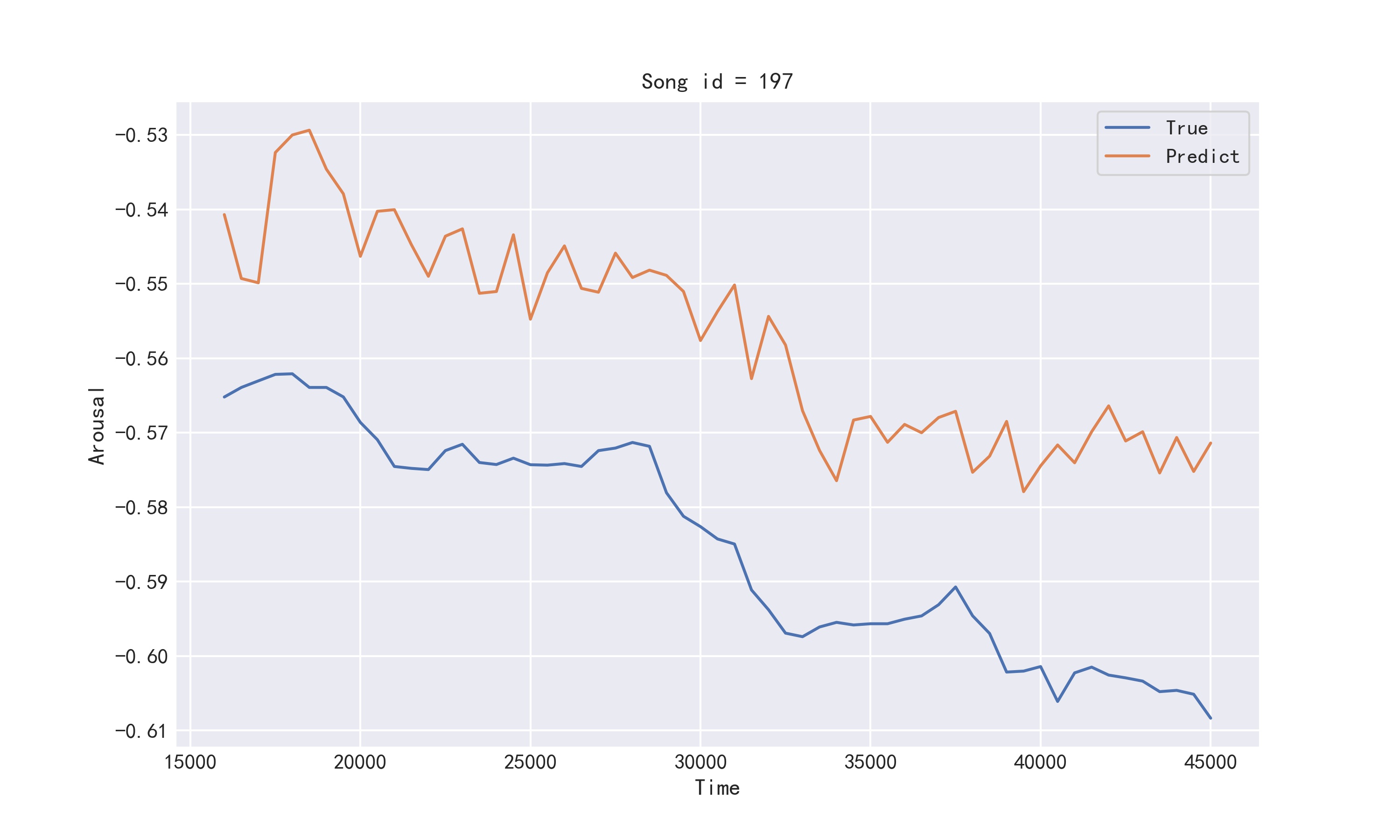

Supplement: S5 File — (ZIP) [file pone.0297712.s005.zip › All prediction results/prediction picture results(Emomusic_75)/song_id_197.jpg]

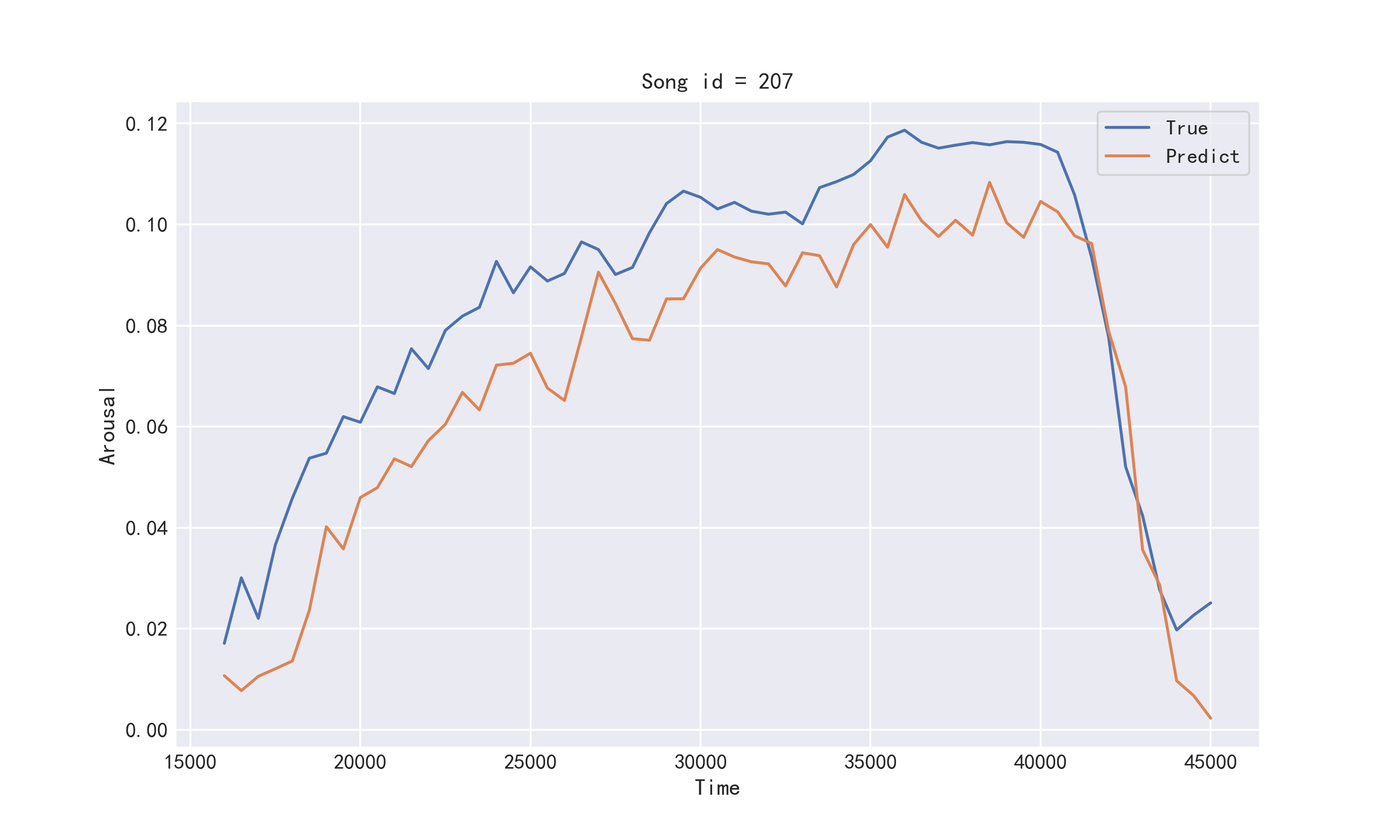

Supplement: S5 File — (ZIP) [file pone.0297712.s005.zip › All prediction results/prediction picture results(Emomusic_75)/song_id_207.jpg]

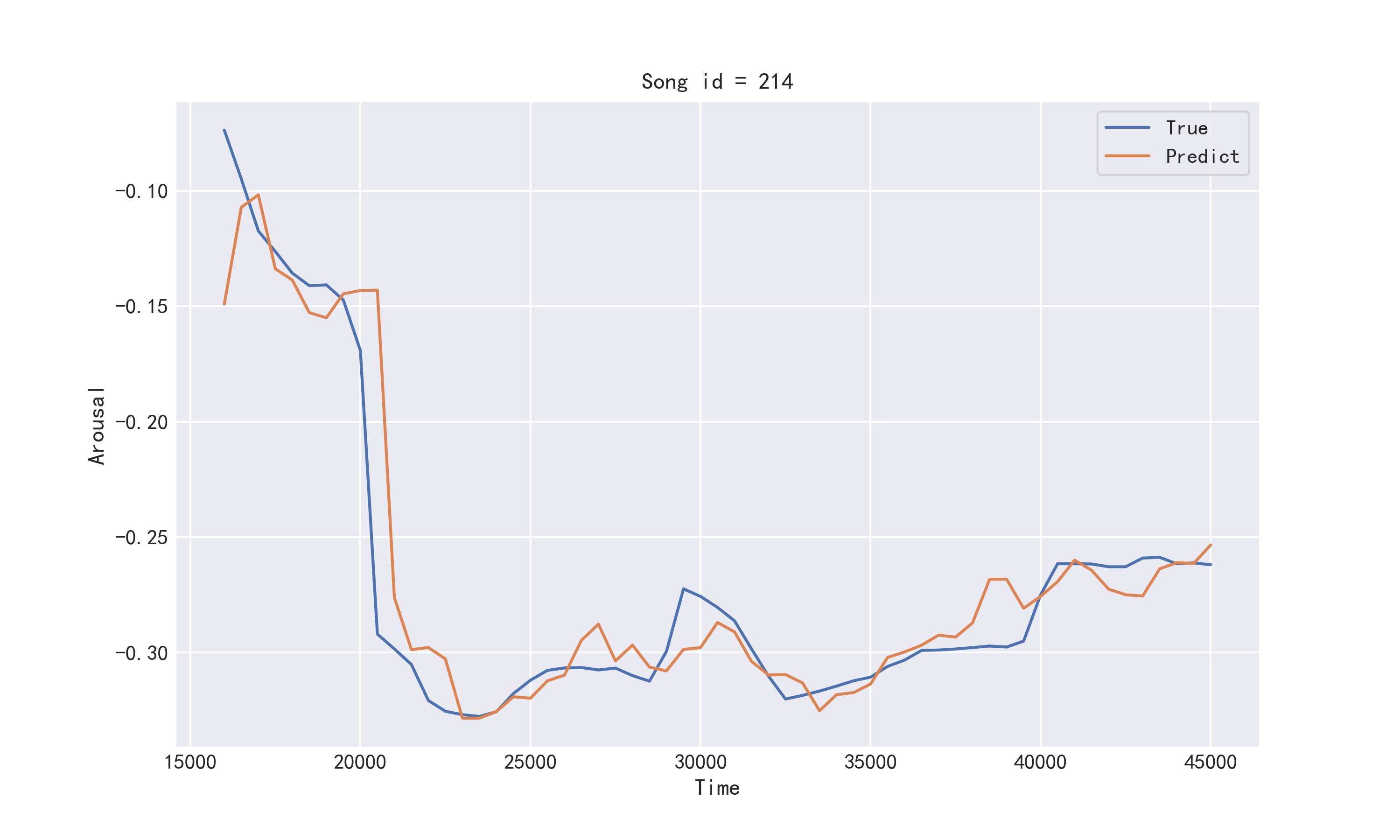

Supplement: S5 File — (ZIP) [file pone.0297712.s005.zip › All prediction results/prediction picture results(Emomusic_75)/song_id_214.jpg]

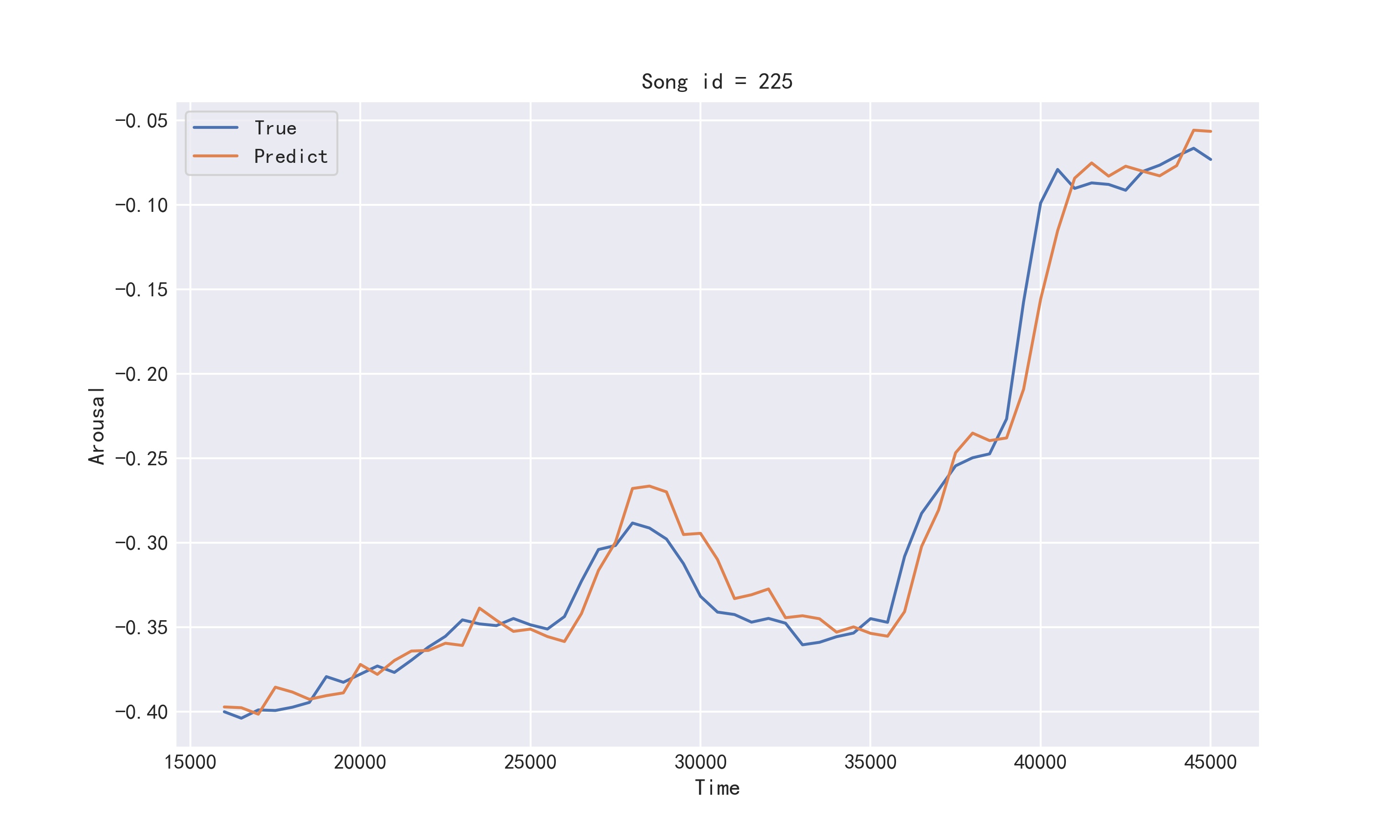

Supplement: S5 File — (ZIP) [file pone.0297712.s005.zip › All prediction results/prediction picture results(Emomusic_75)/song_id_225.jpg]

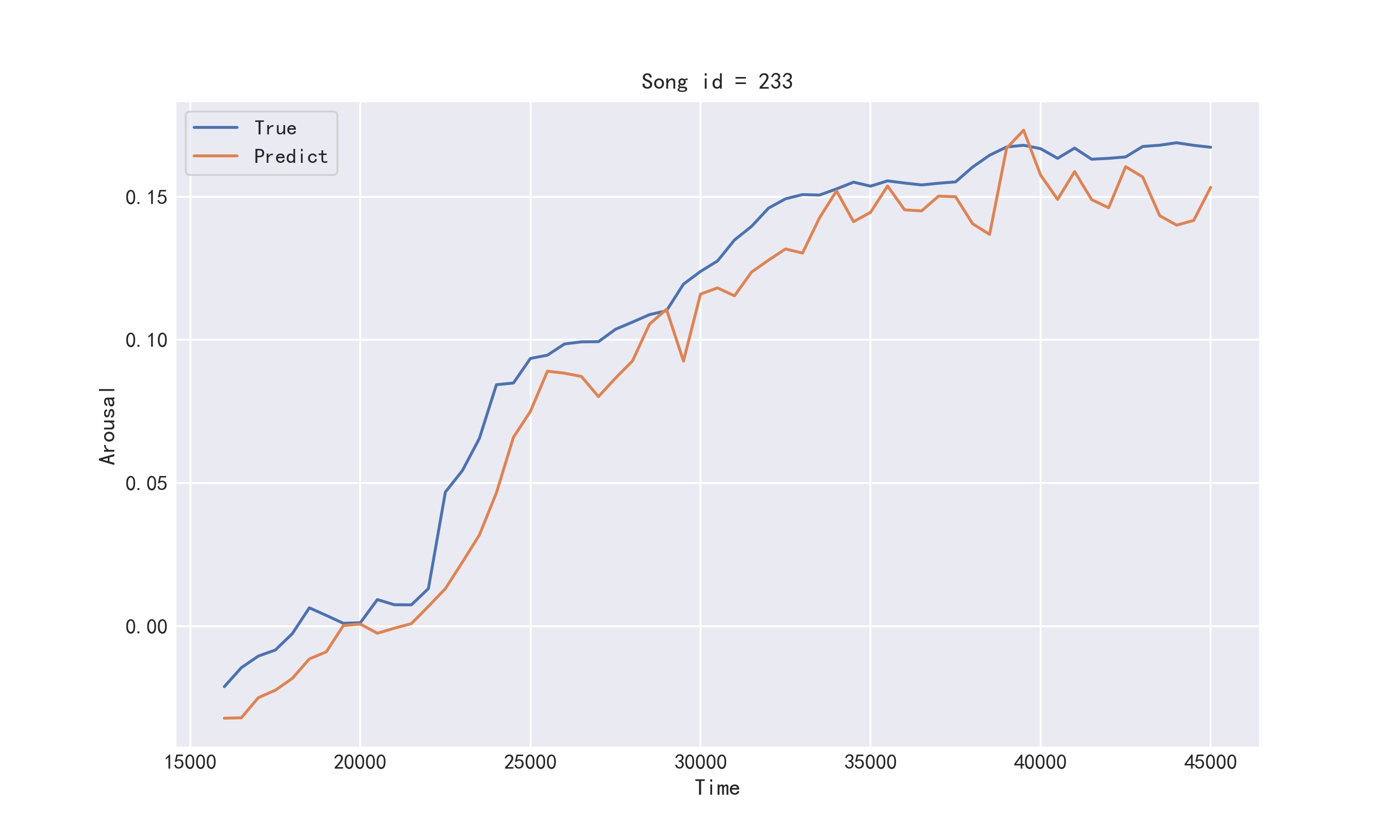

Supplement: S5 File — (ZIP) [file pone.0297712.s005.zip › All prediction results/prediction picture results(Emomusic_75)/song_id_233.jpg]

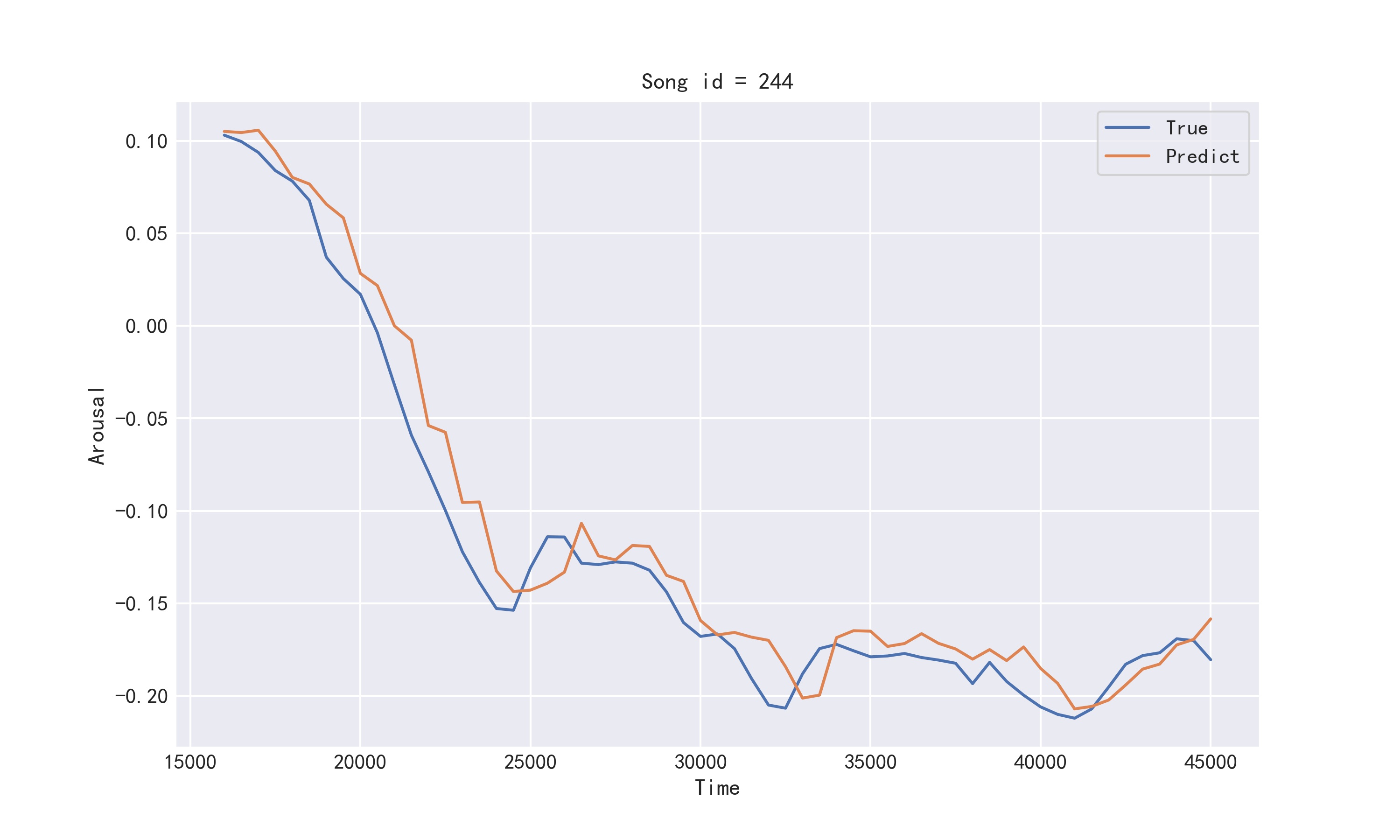

Supplement: S5 File — (ZIP) [file pone.0297712.s005.zip › All prediction results/prediction picture results(Emomusic_75)/song_id_244.jpg]

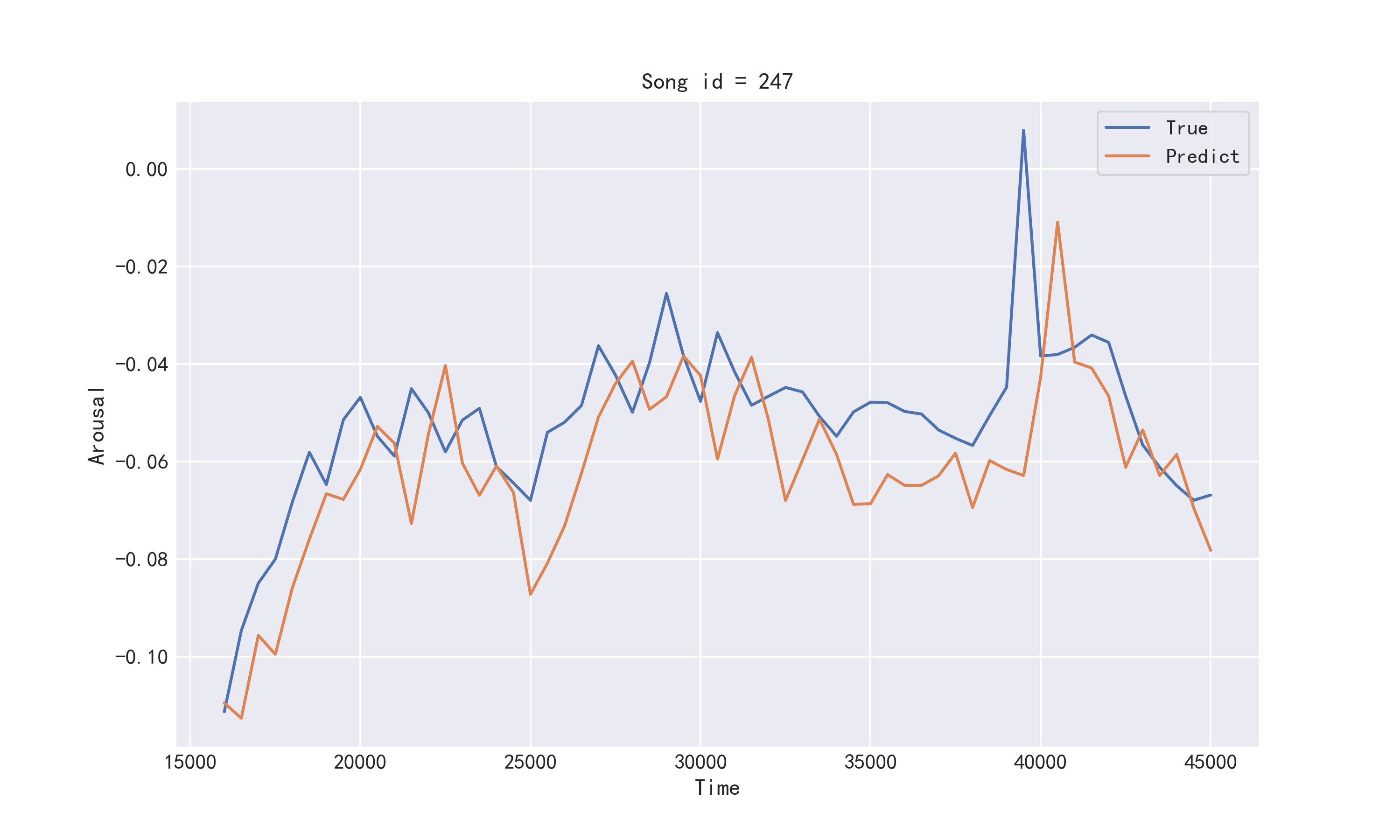

Supplement: S5 File — (ZIP) [file pone.0297712.s005.zip › All prediction results/prediction picture results(Emomusic_75)/song_id_247.jpg]

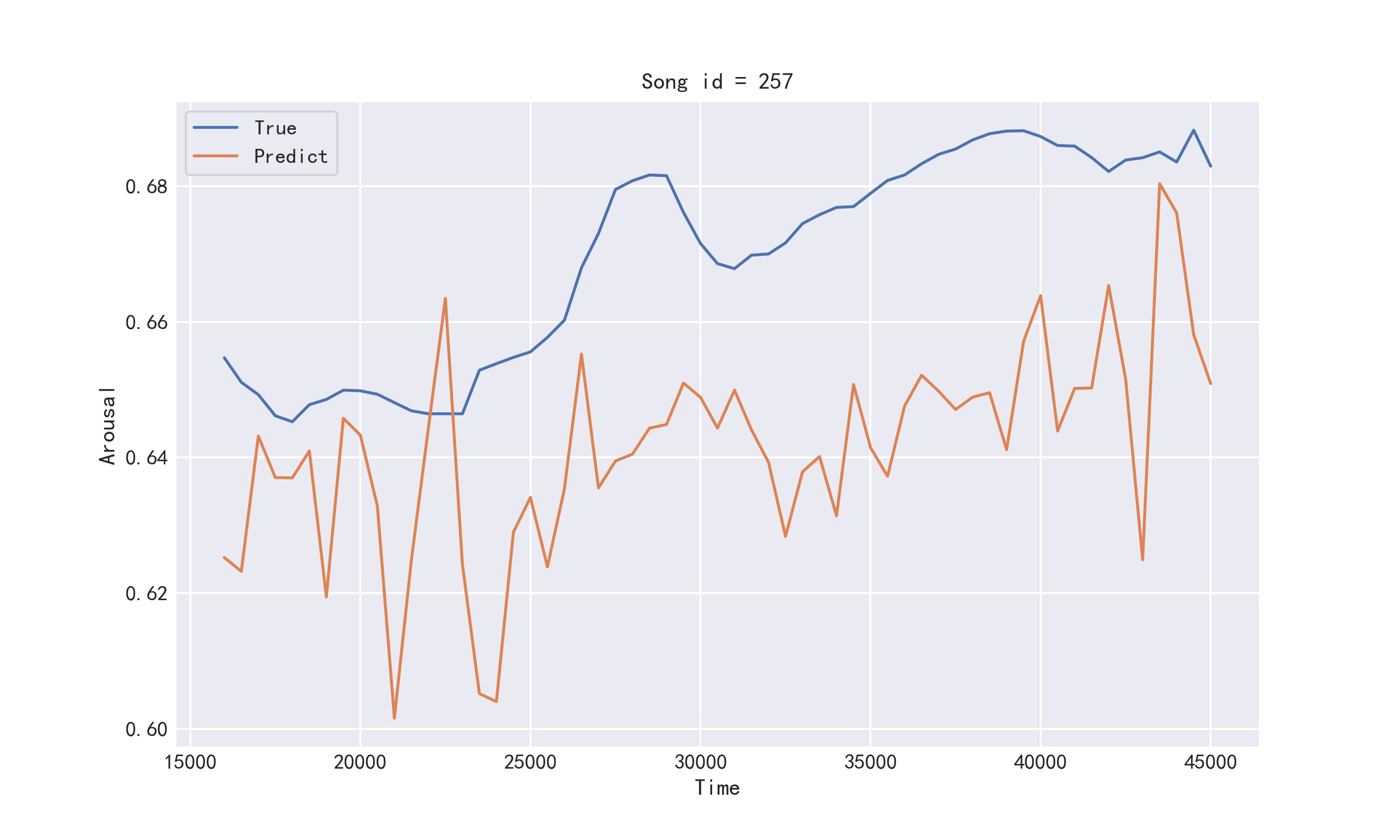

Supplement: S5 File — (ZIP) [file pone.0297712.s005.zip › All prediction results/prediction picture results(Emomusic_75)/song_id_257.jpg]

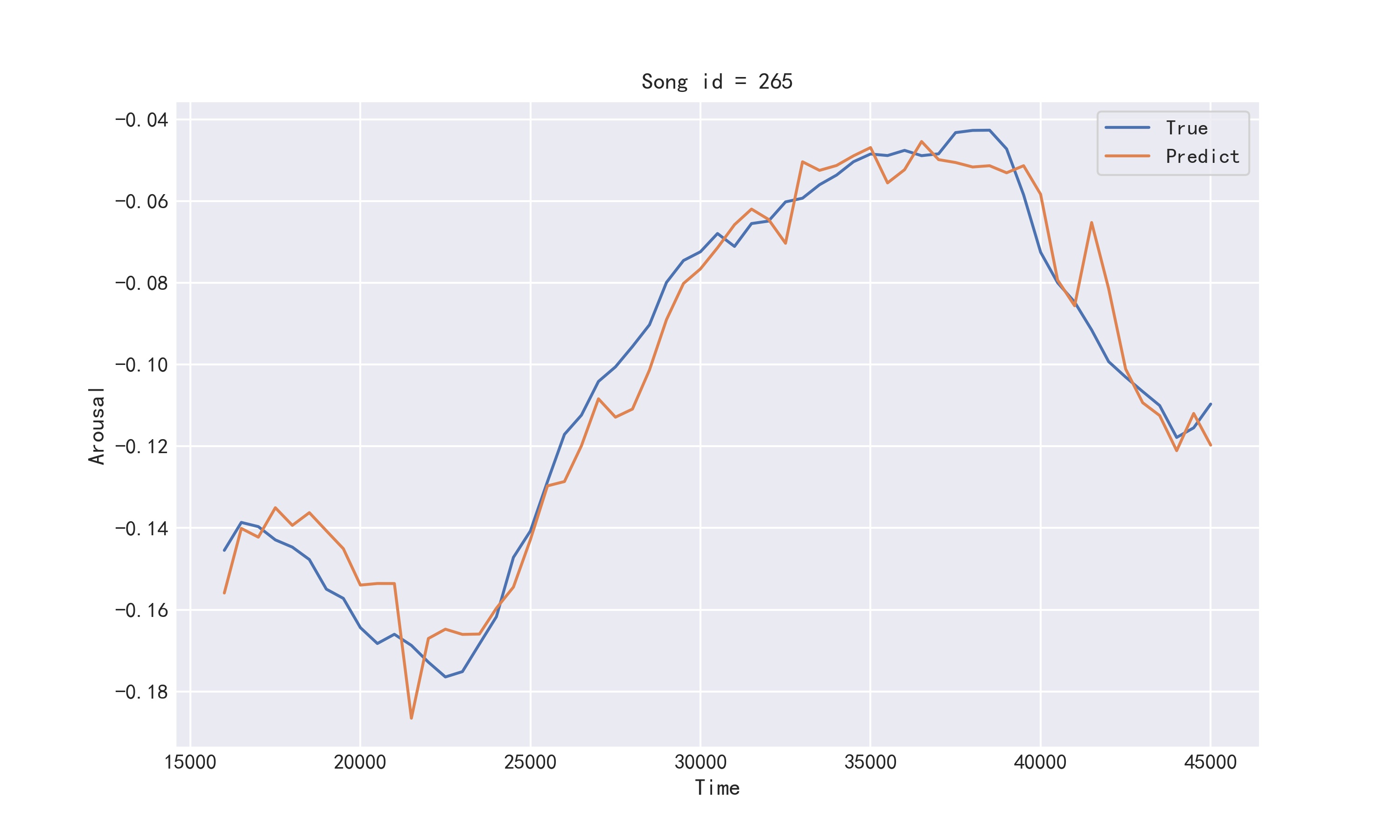

Supplement: S5 File — (ZIP) [file pone.0297712.s005.zip › All prediction results/prediction picture results(Emomusic_75)/song_id_265.jpg]

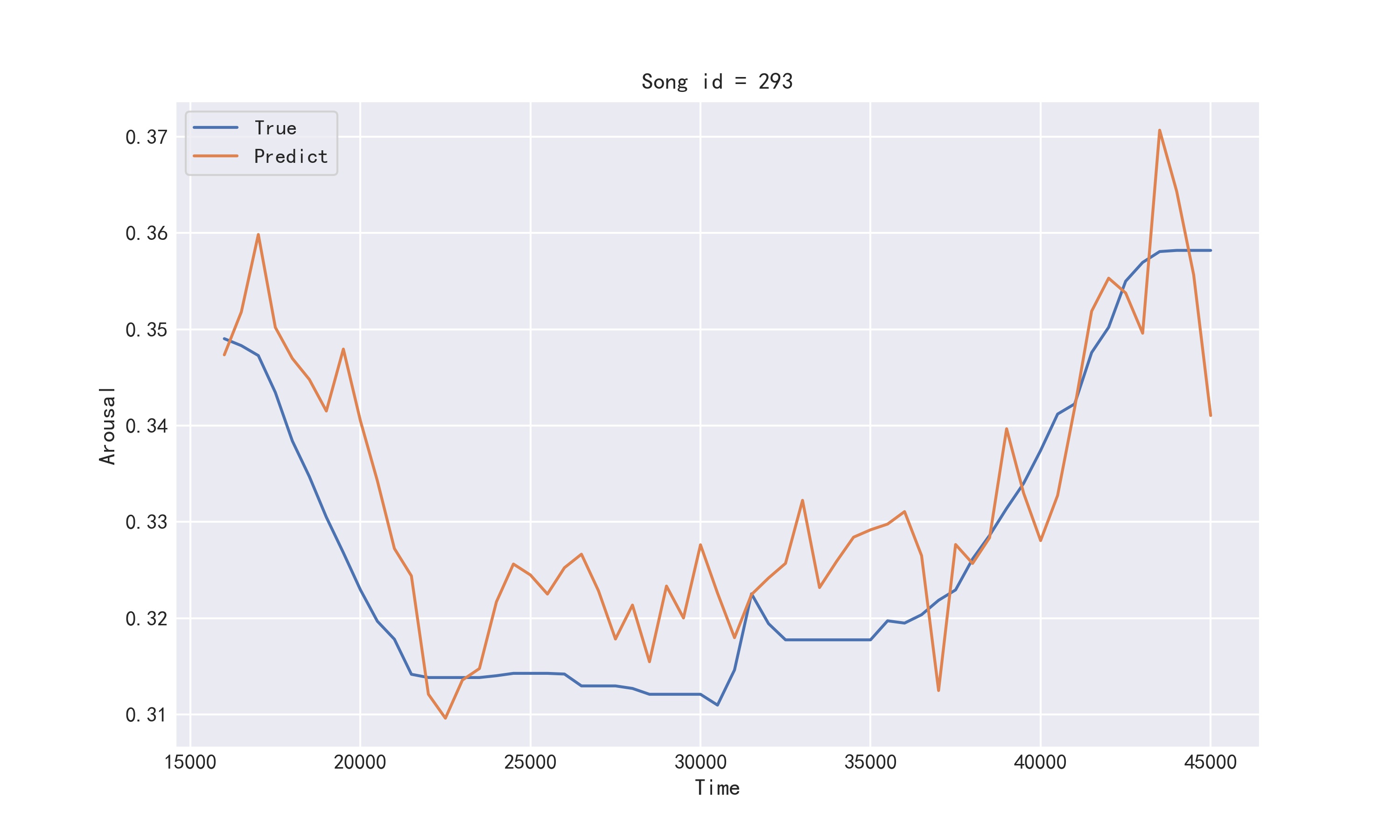

Supplement: S5 File — (ZIP) [file pone.0297712.s005.zip › All prediction results/prediction picture results(Emomusic_75)/song_id_293.jpg]

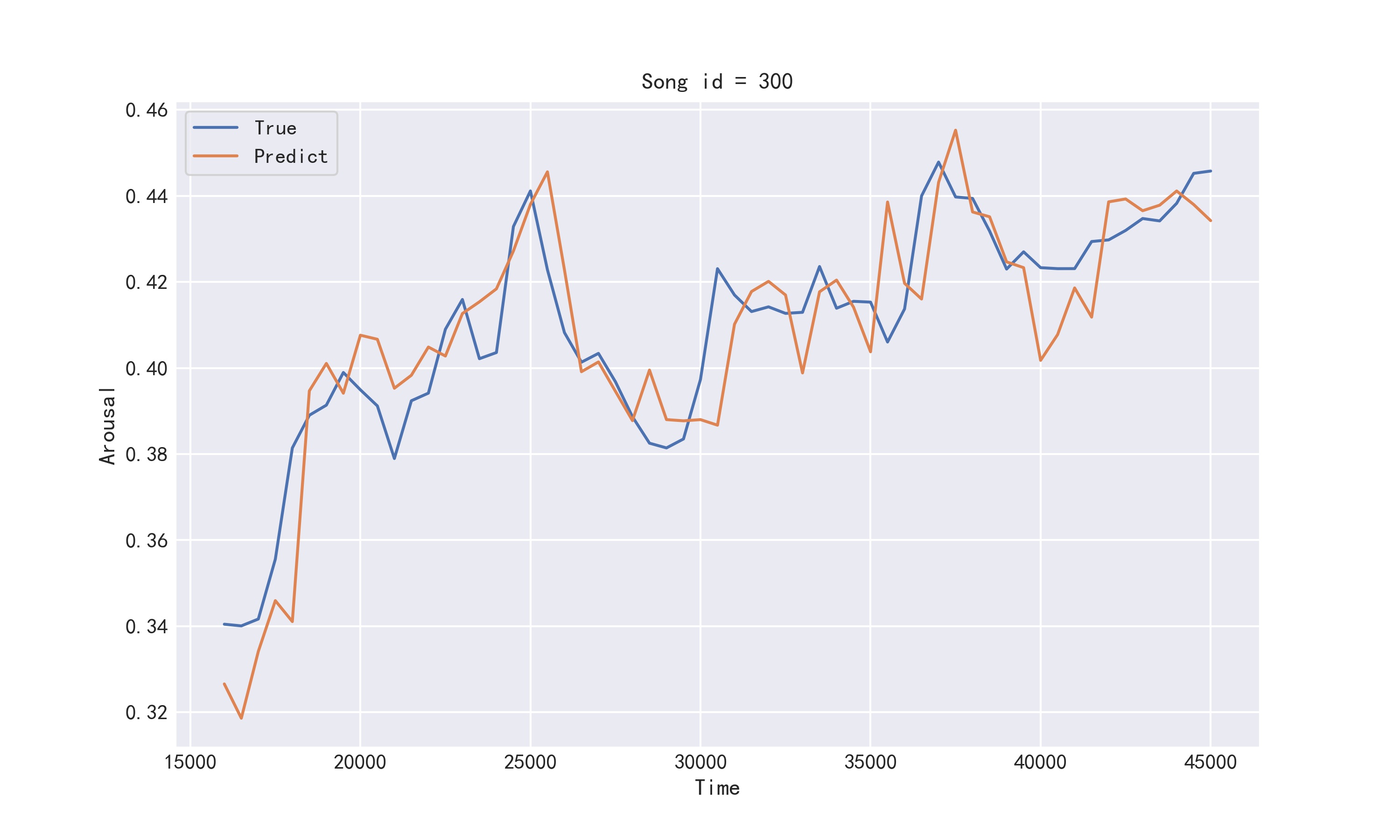

Supplement: S5 File — (ZIP) [file pone.0297712.s005.zip › All prediction results/prediction picture results(Emomusic_75)/song_id_300.jpg]

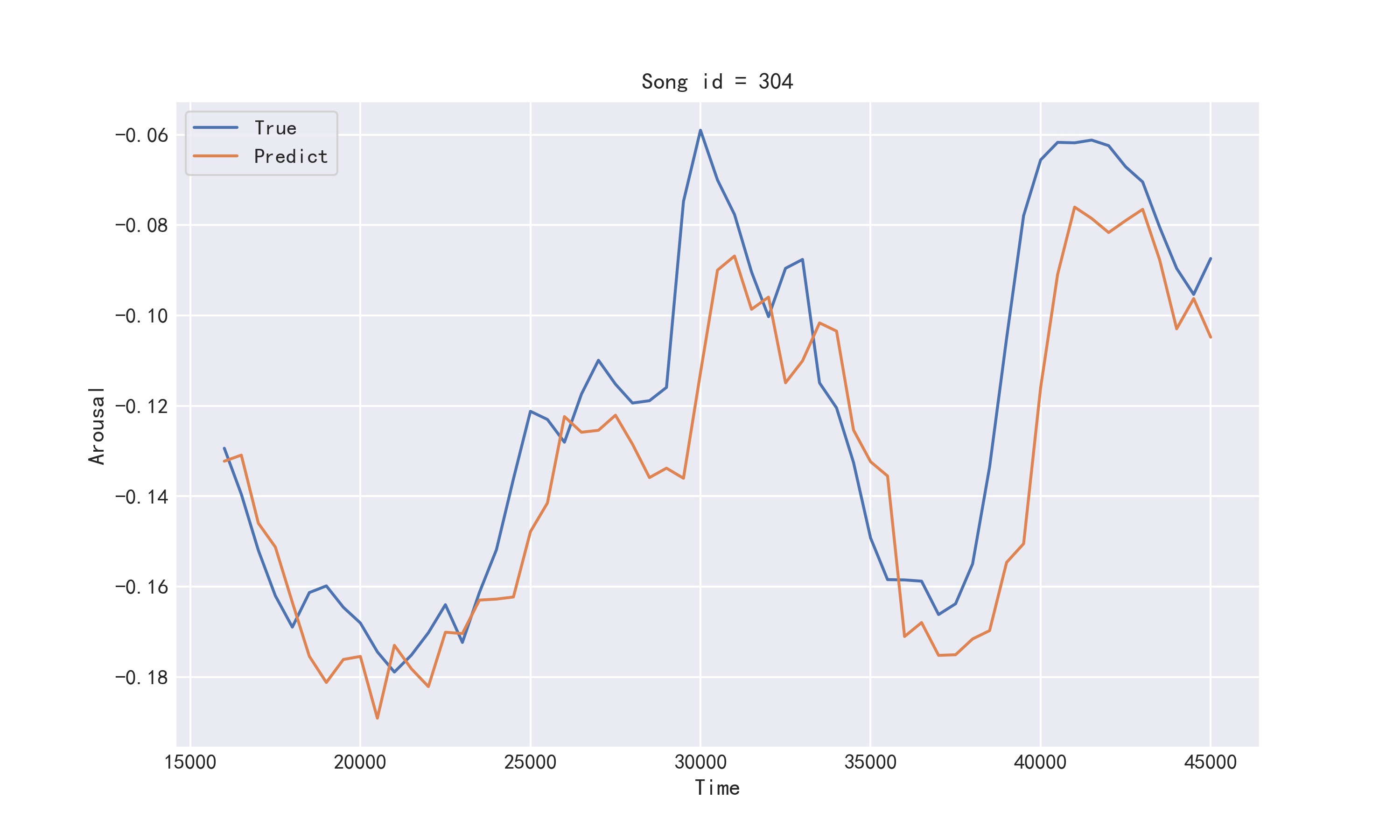

Supplement: S5 File — (ZIP) [file pone.0297712.s005.zip › All prediction results/prediction picture results(Emomusic_75)/song_id_304.jpg]

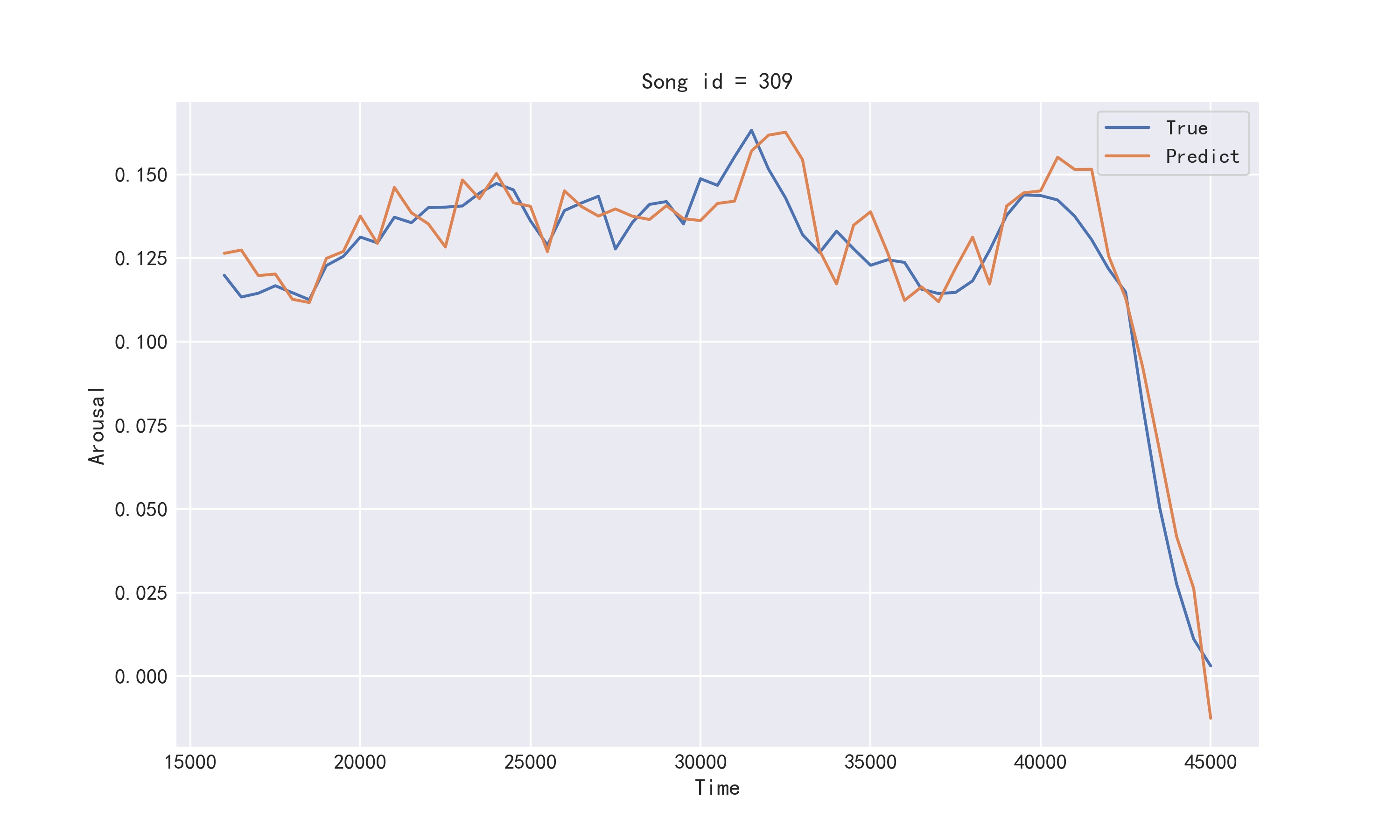

Supplement: S5 File — (ZIP) [file pone.0297712.s005.zip › All prediction results/prediction picture results(Emomusic_75)/song_id_309.jpg]

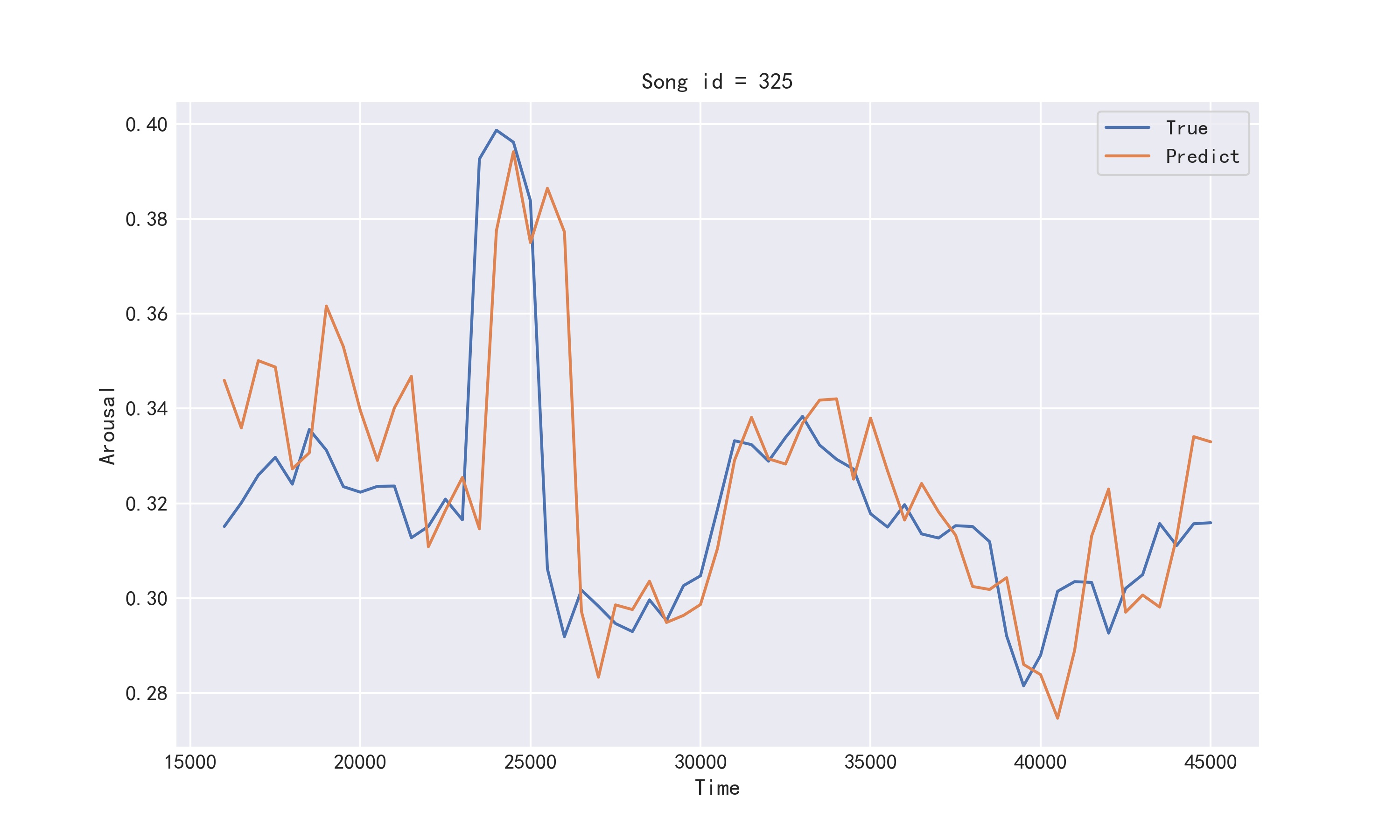

Supplement: S5 File — (ZIP) [file pone.0297712.s005.zip › All prediction results/prediction picture results(Emomusic_75)/song_id_325.jpg]

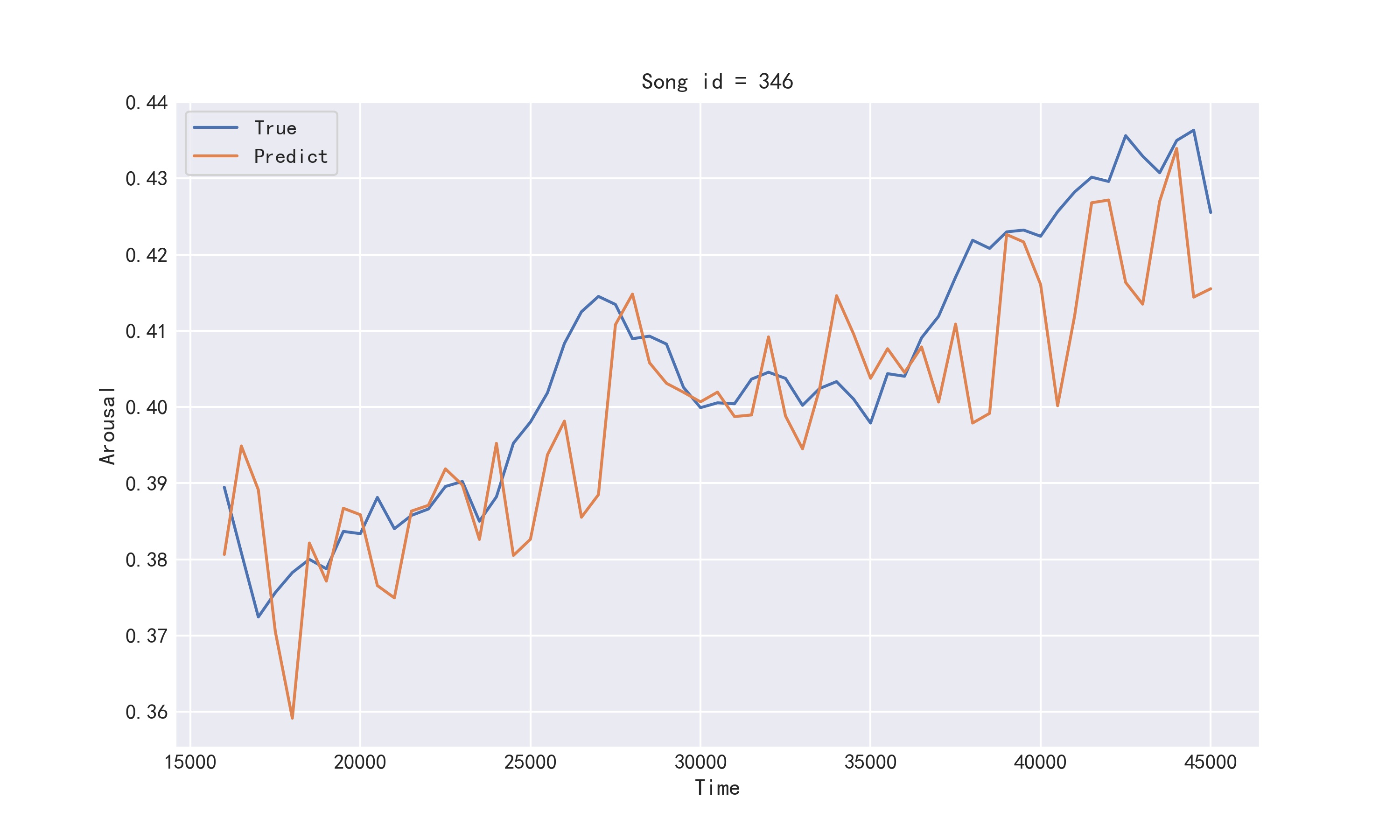

Supplement: S5 File — (ZIP) [file pone.0297712.s005.zip › All prediction results/prediction picture results(Emomusic_75)/song_id_346.jpg]

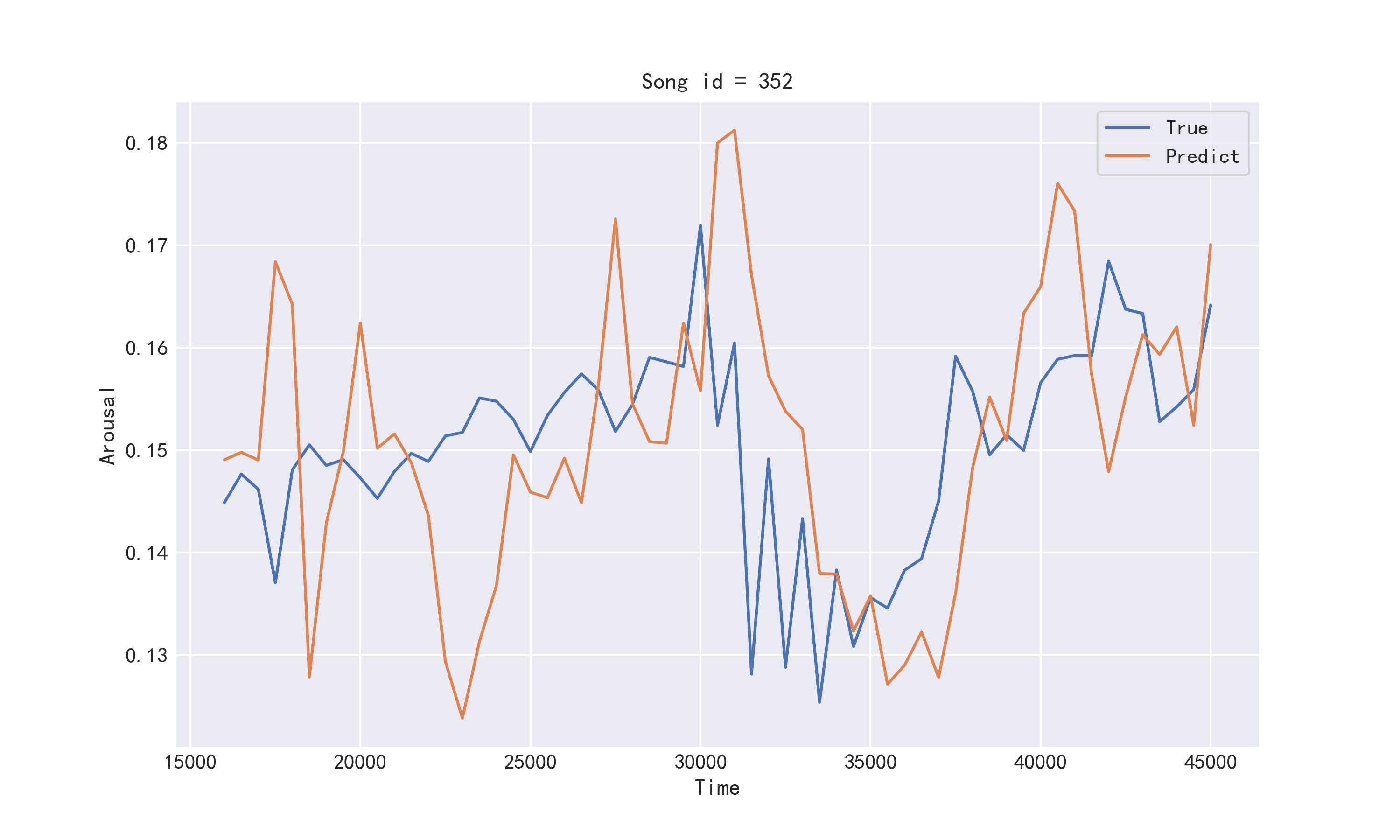

Supplement: S5 File — (ZIP) [file pone.0297712.s005.zip › All prediction results/prediction picture results(Emomusic_75)/song_id_352.jpg]

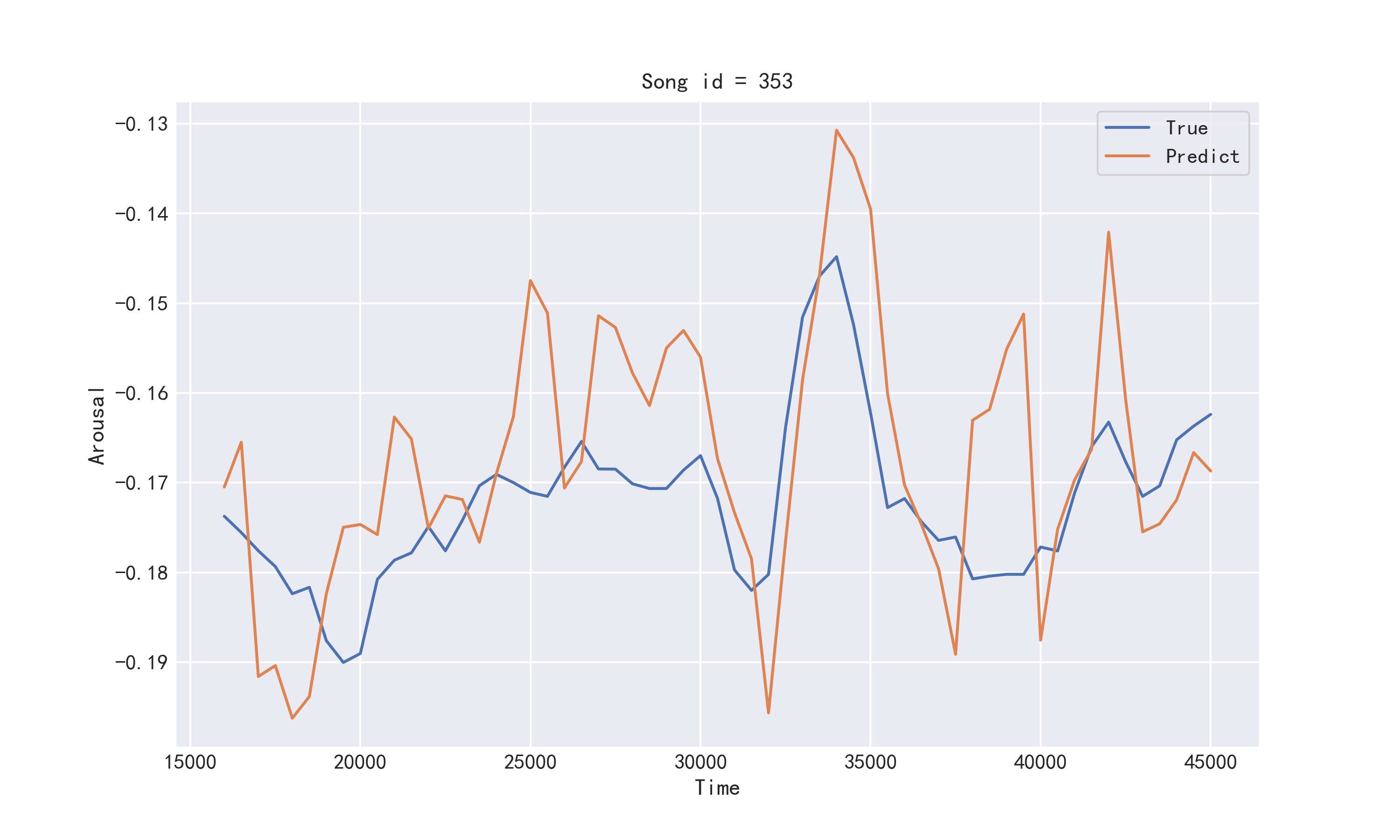

Supplement: S5 File — (ZIP) [file pone.0297712.s005.zip › All prediction results/prediction picture results(Emomusic_75)/song_id_353.jpg]

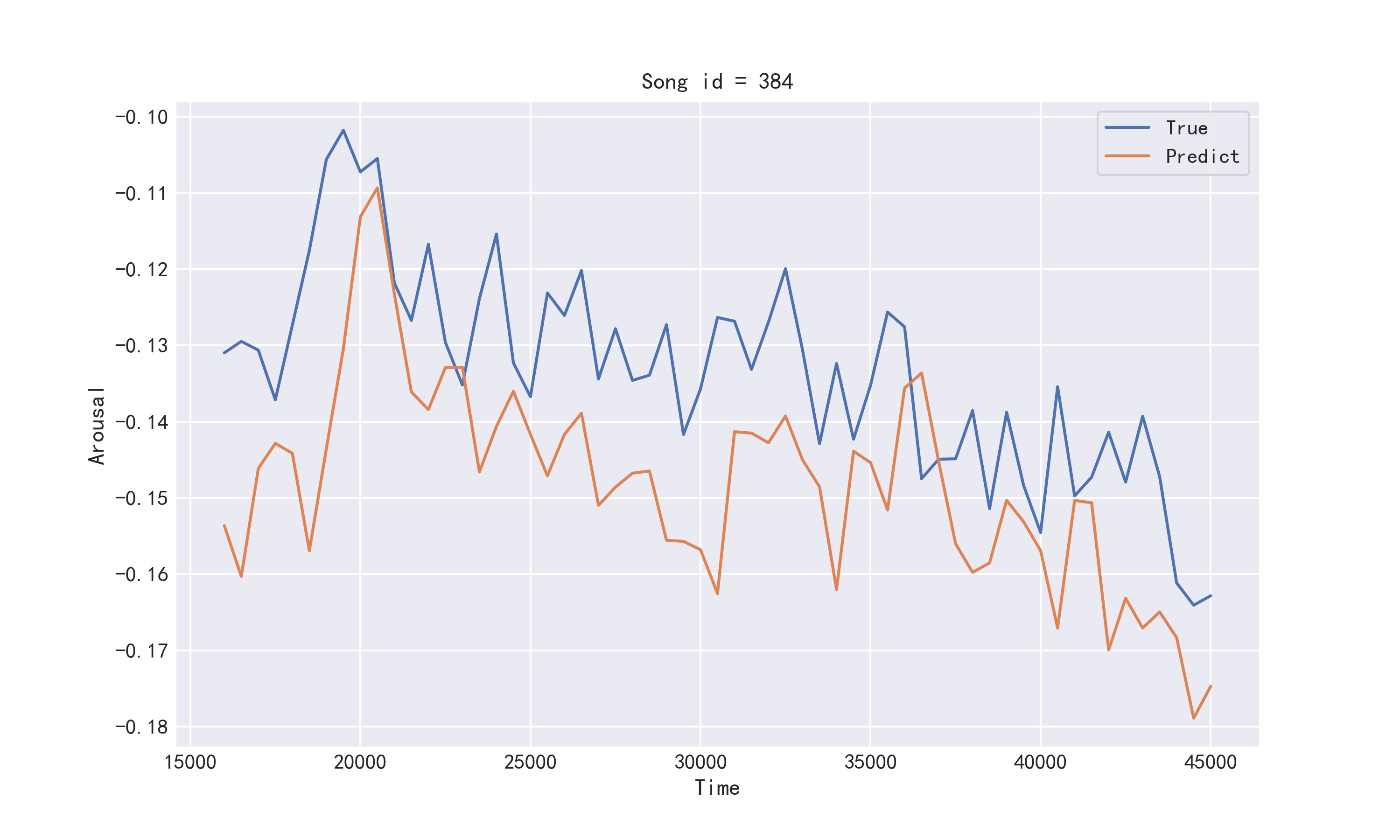

Supplement: S5 File — (ZIP) [file pone.0297712.s005.zip › All prediction results/prediction picture results(Emomusic_75)/song_id_384.jpg]

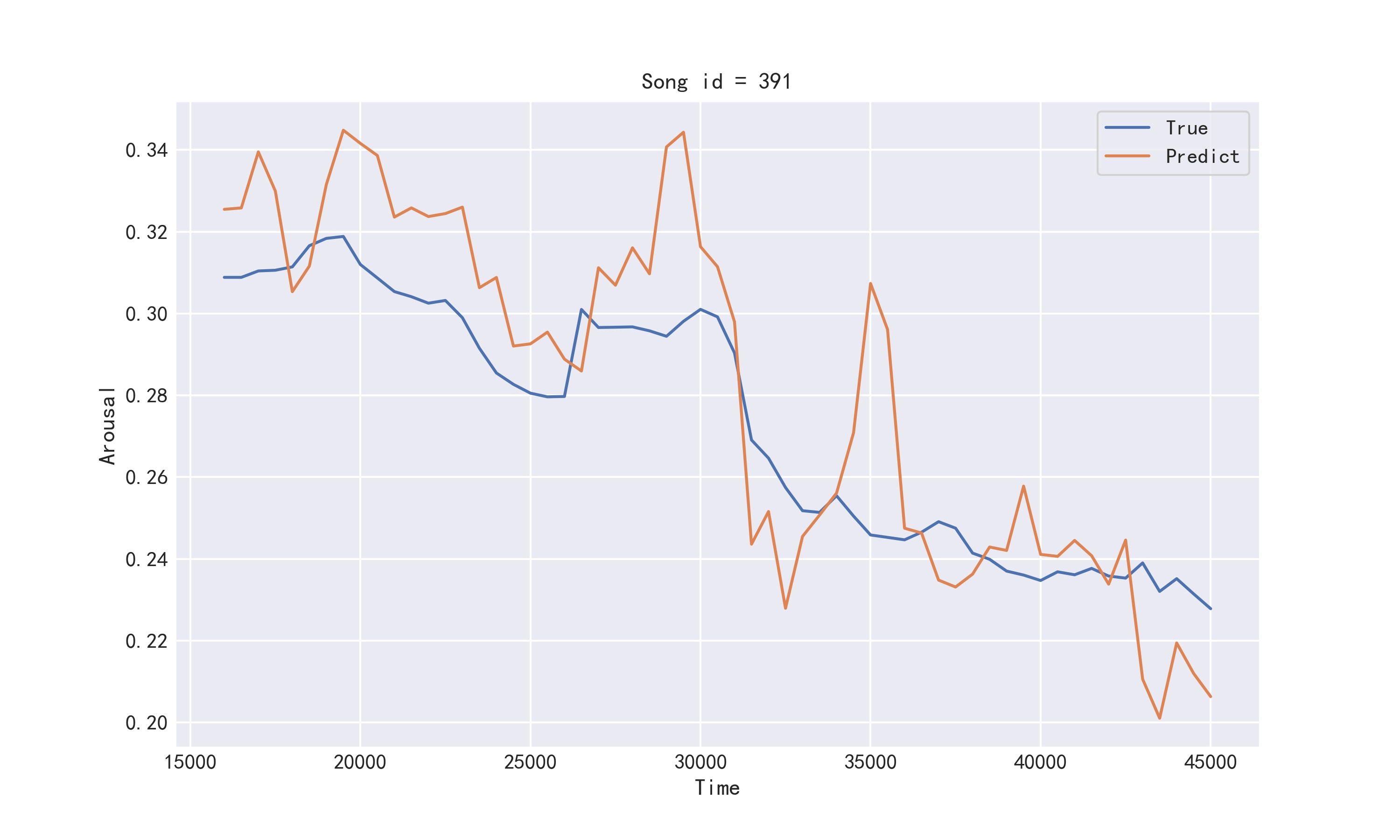

Supplement: S5 File — (ZIP) [file pone.0297712.s005.zip › All prediction results/prediction picture results(Emomusic_75)/song_id_391.jpg]

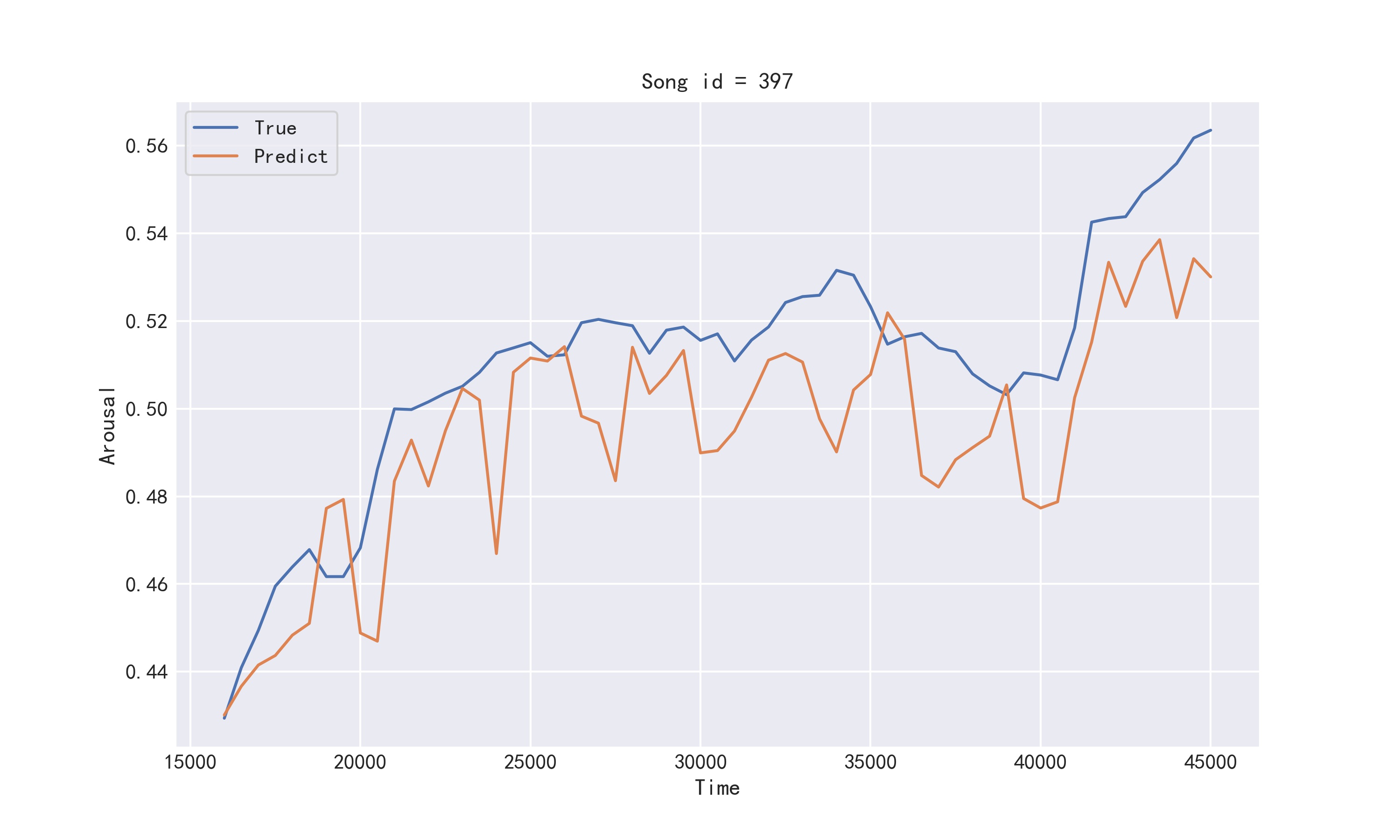

Supplement: S5 File — (ZIP) [file pone.0297712.s005.zip › All prediction results/prediction picture results(Emomusic_75)/song_id_397.jpg]

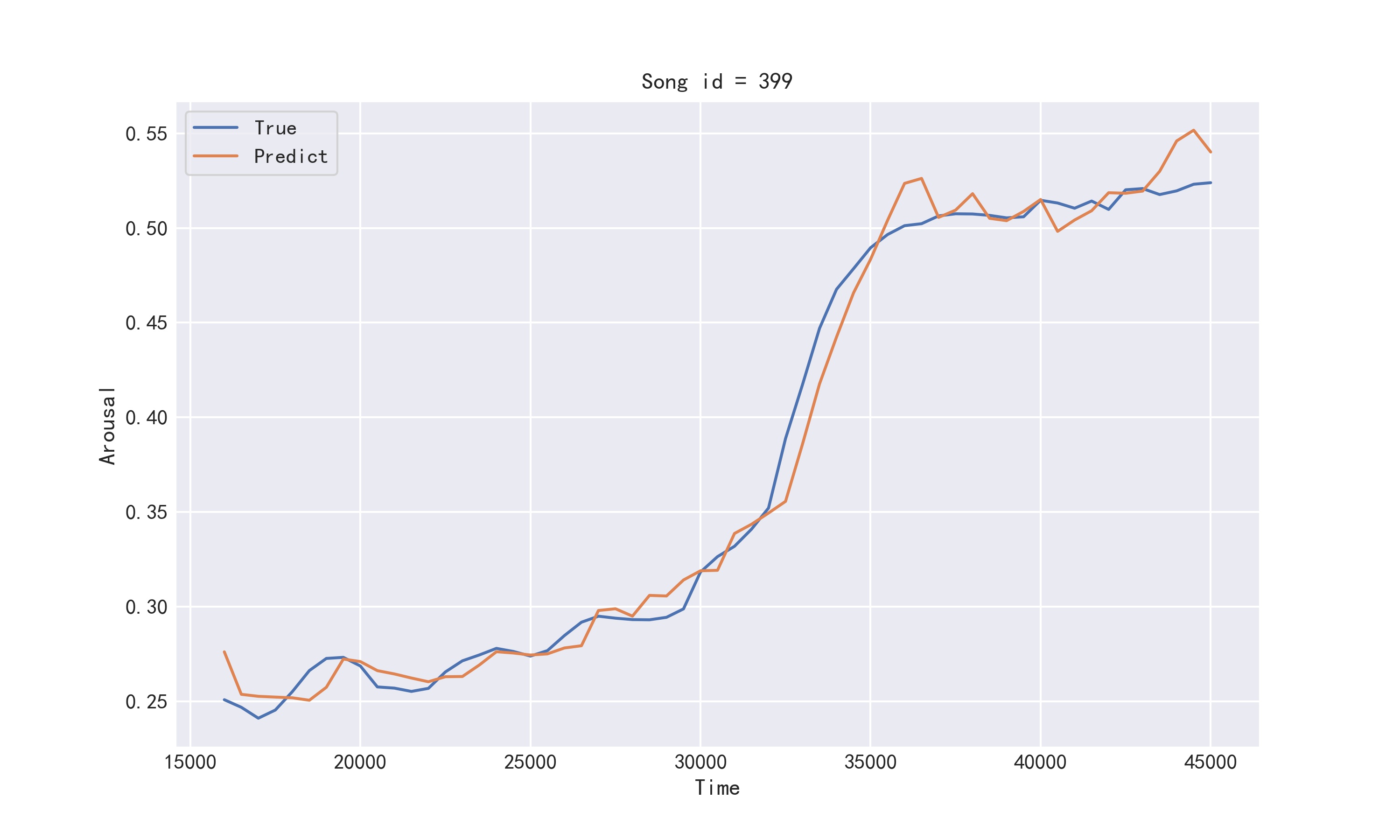

Supplement: S5 File — (ZIP) [file pone.0297712.s005.zip › All prediction results/prediction picture results(Emomusic_75)/song_id_399.jpg]

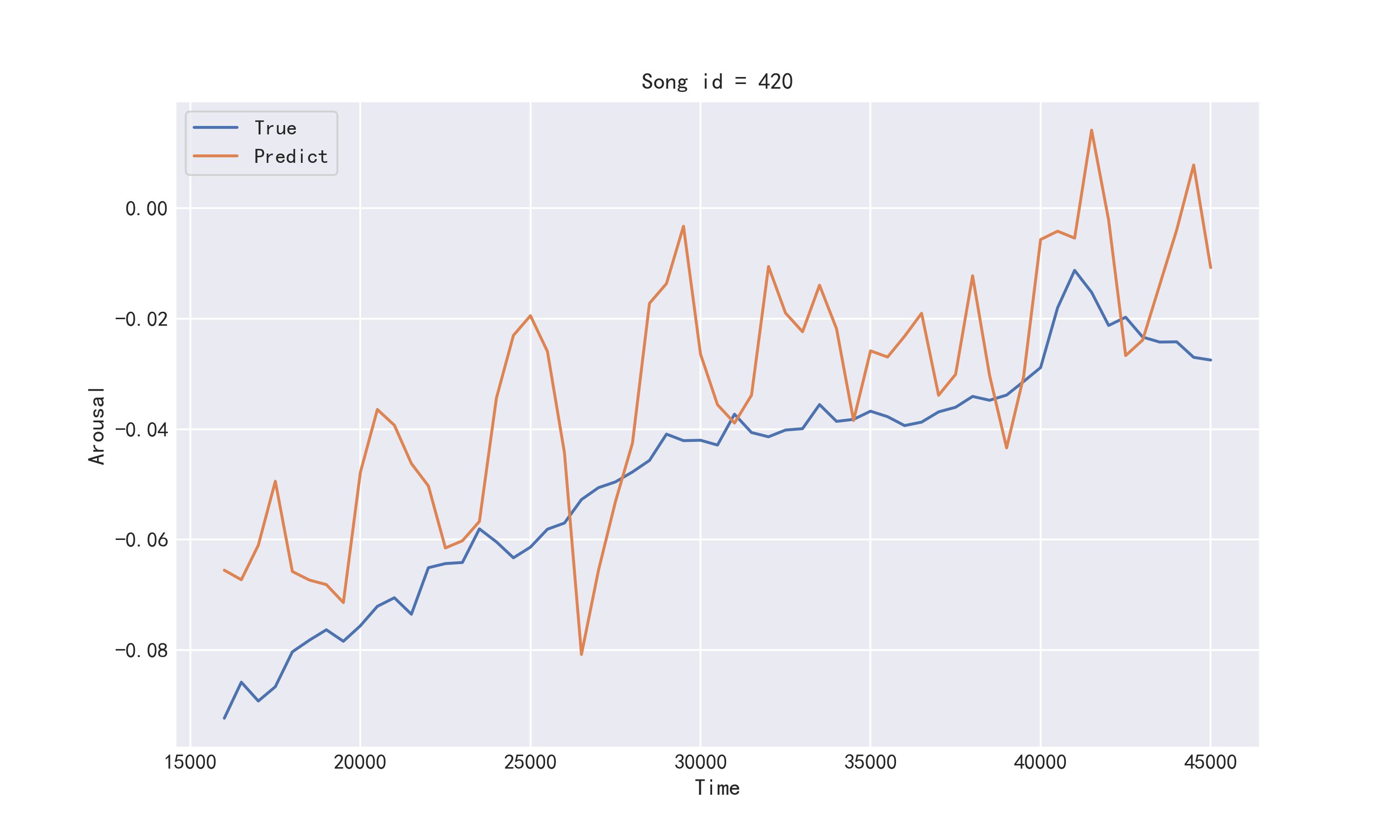

Supplement: S5 File — (ZIP) [file pone.0297712.s005.zip › All prediction results/prediction picture results(Emomusic_75)/song_id_420.jpg]

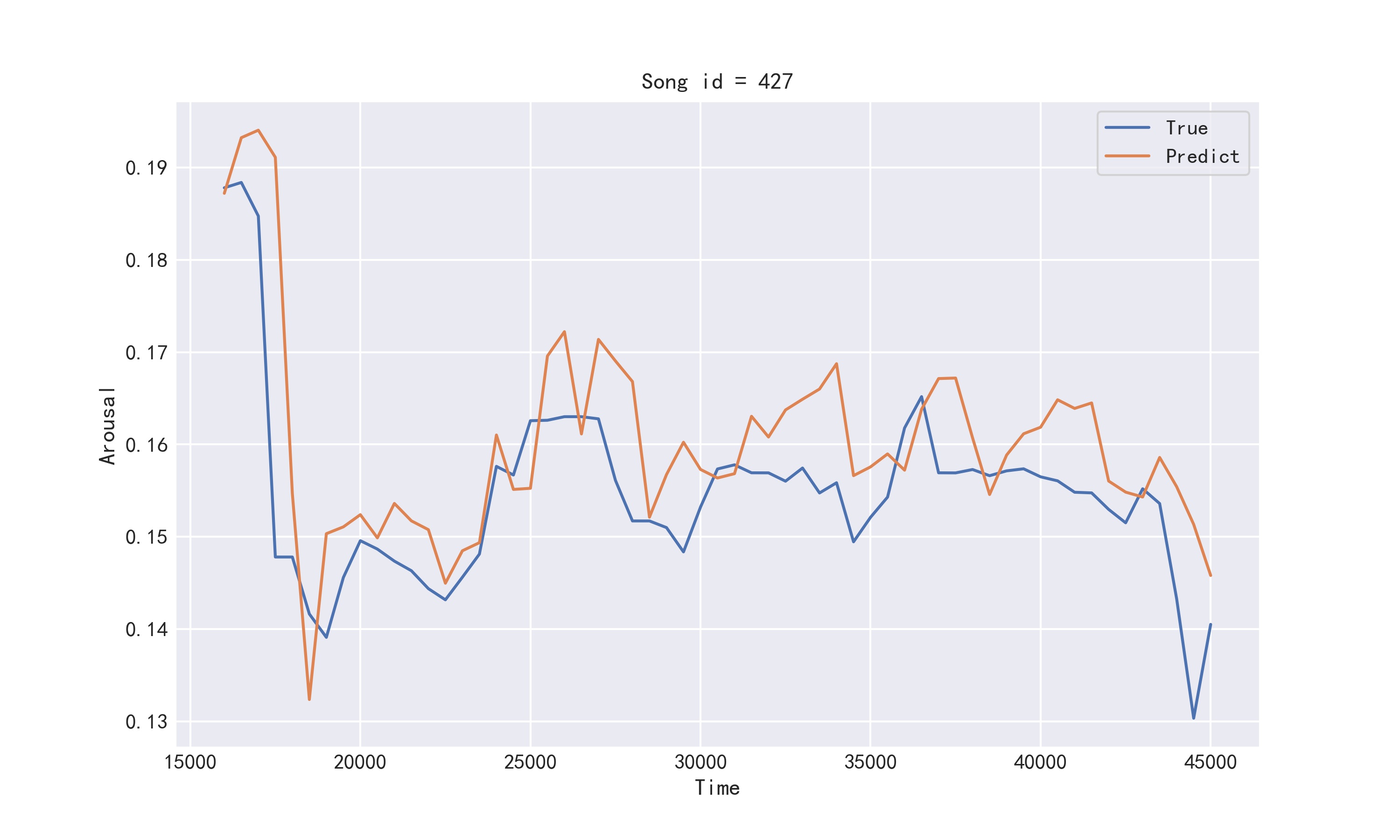

Supplement: S5 File — (ZIP) [file pone.0297712.s005.zip › All prediction results/prediction picture results(Emomusic_75)/song_id_427.jpg]

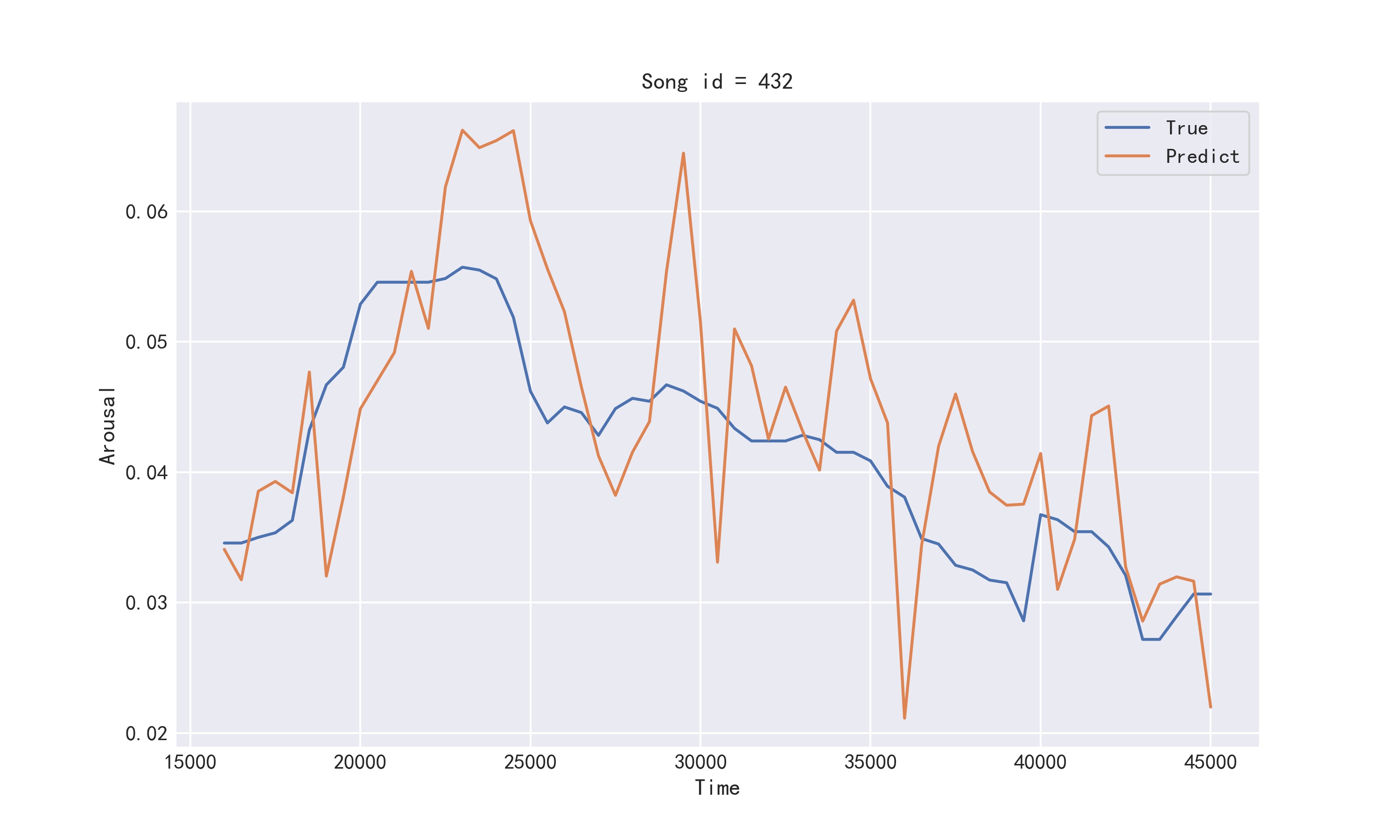

Supplement: S5 File — (ZIP) [file pone.0297712.s005.zip › All prediction results/prediction picture results(Emomusic_75)/song_id_432.jpg]

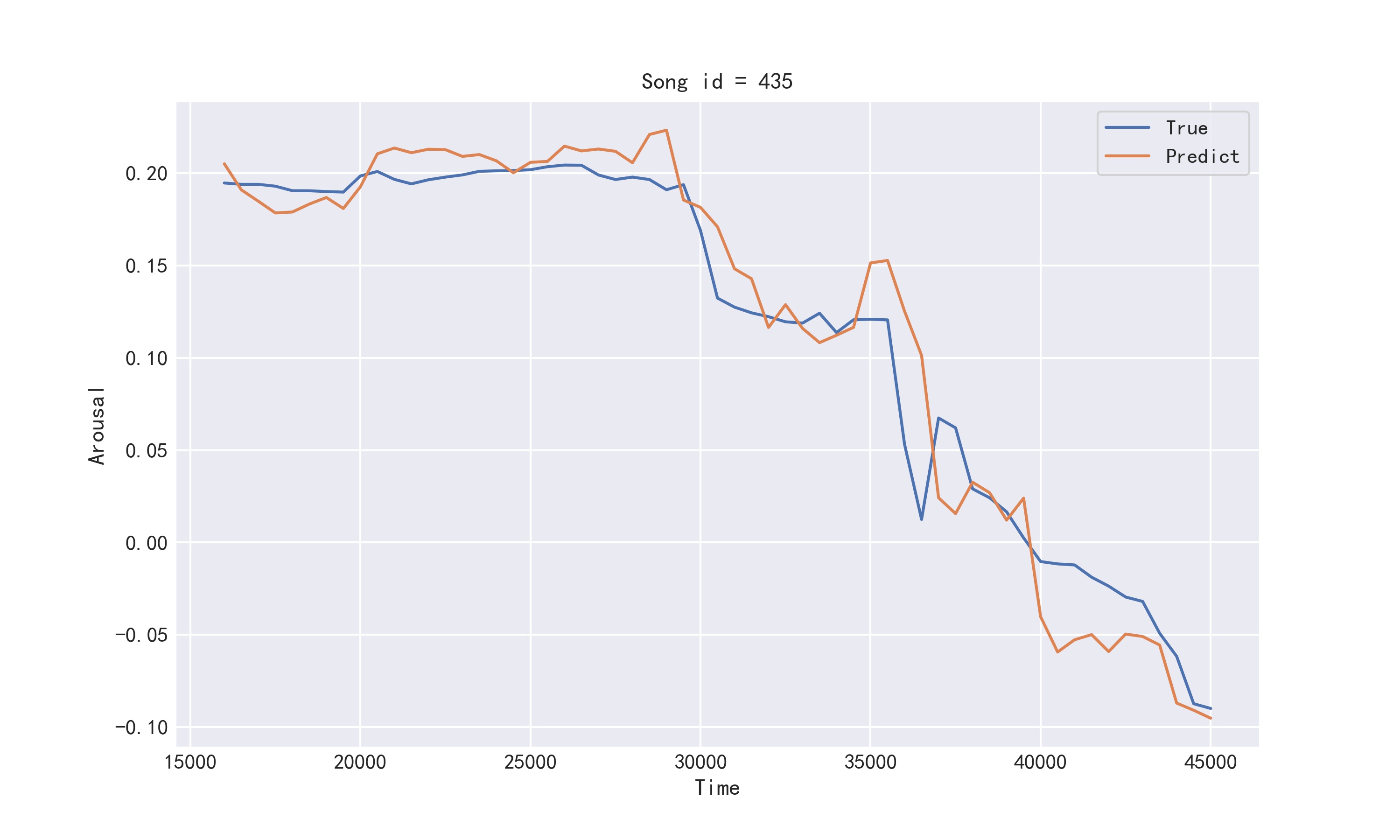

Supplement: S5 File — (ZIP) [file pone.0297712.s005.zip › All prediction results/prediction picture results(Emomusic_75)/song_id_435.jpg]

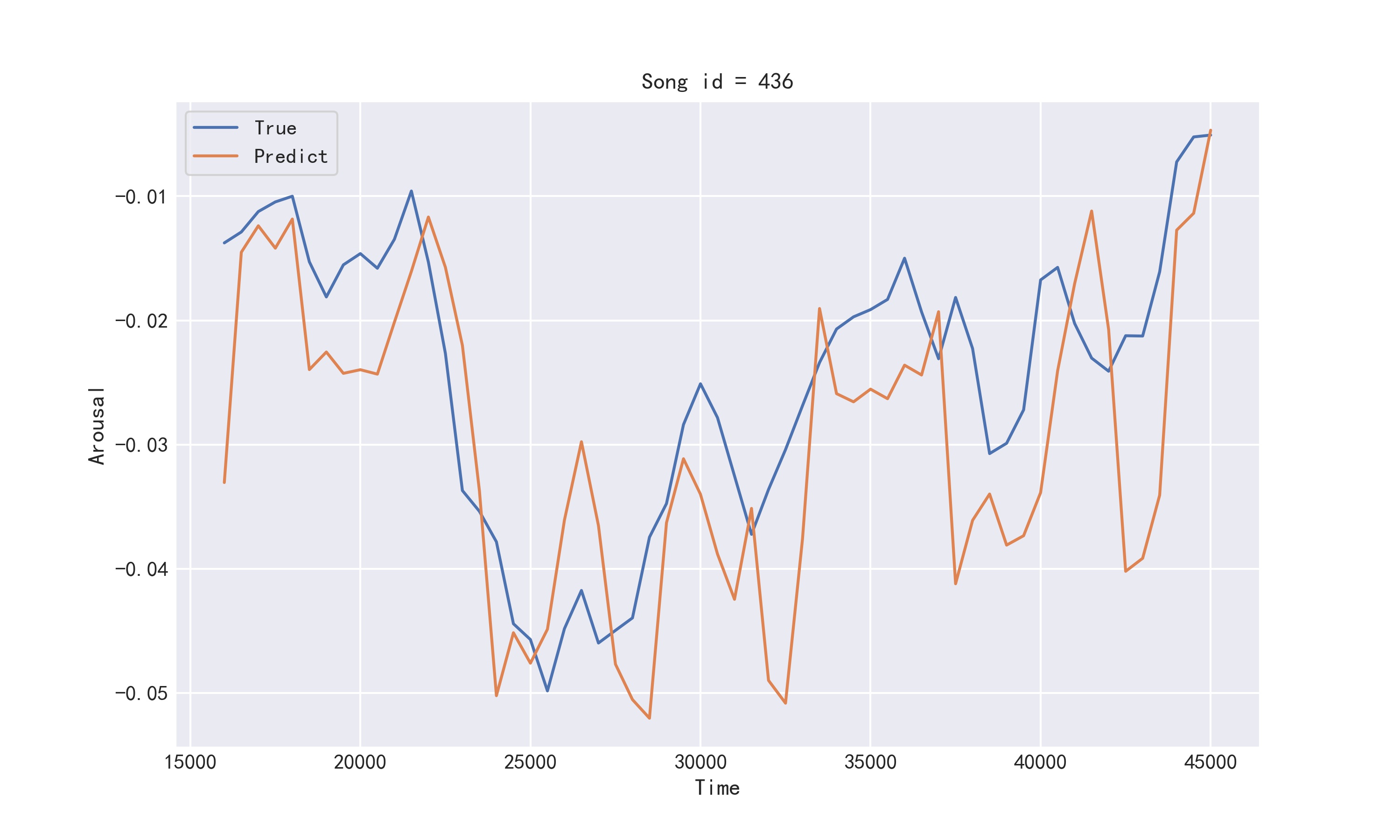

Supplement: S5 File — (ZIP) [file pone.0297712.s005.zip › All prediction results/prediction picture results(Emomusic_75)/song_id_436.jpg]

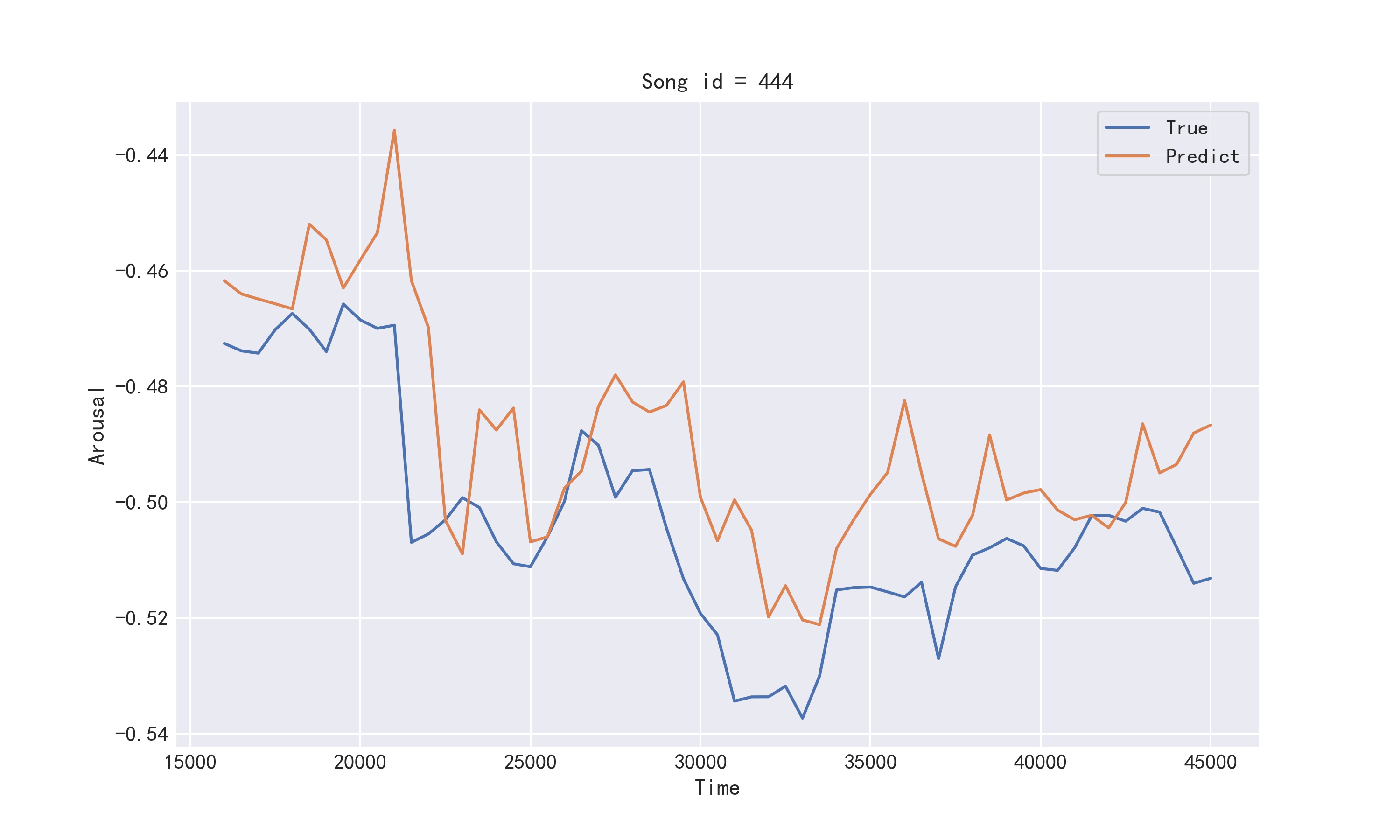

Supplement: S5 File — (ZIP) [file pone.0297712.s005.zip › All prediction results/prediction picture results(Emomusic_75)/song_id_444.jpg]

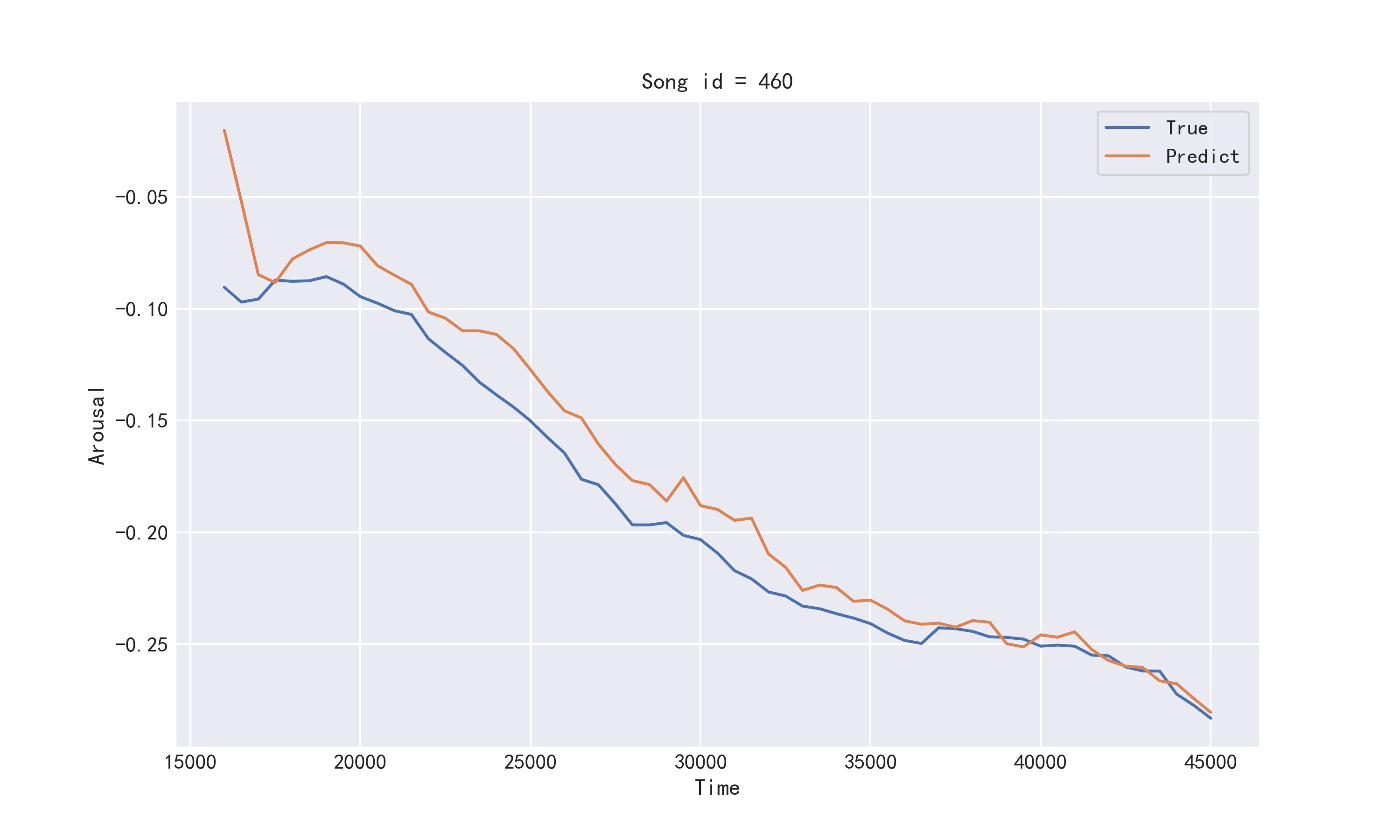

Supplement: S5 File — (ZIP) [file pone.0297712.s005.zip › All prediction results/prediction picture results(Emomusic_75)/song_id_460.jpg]

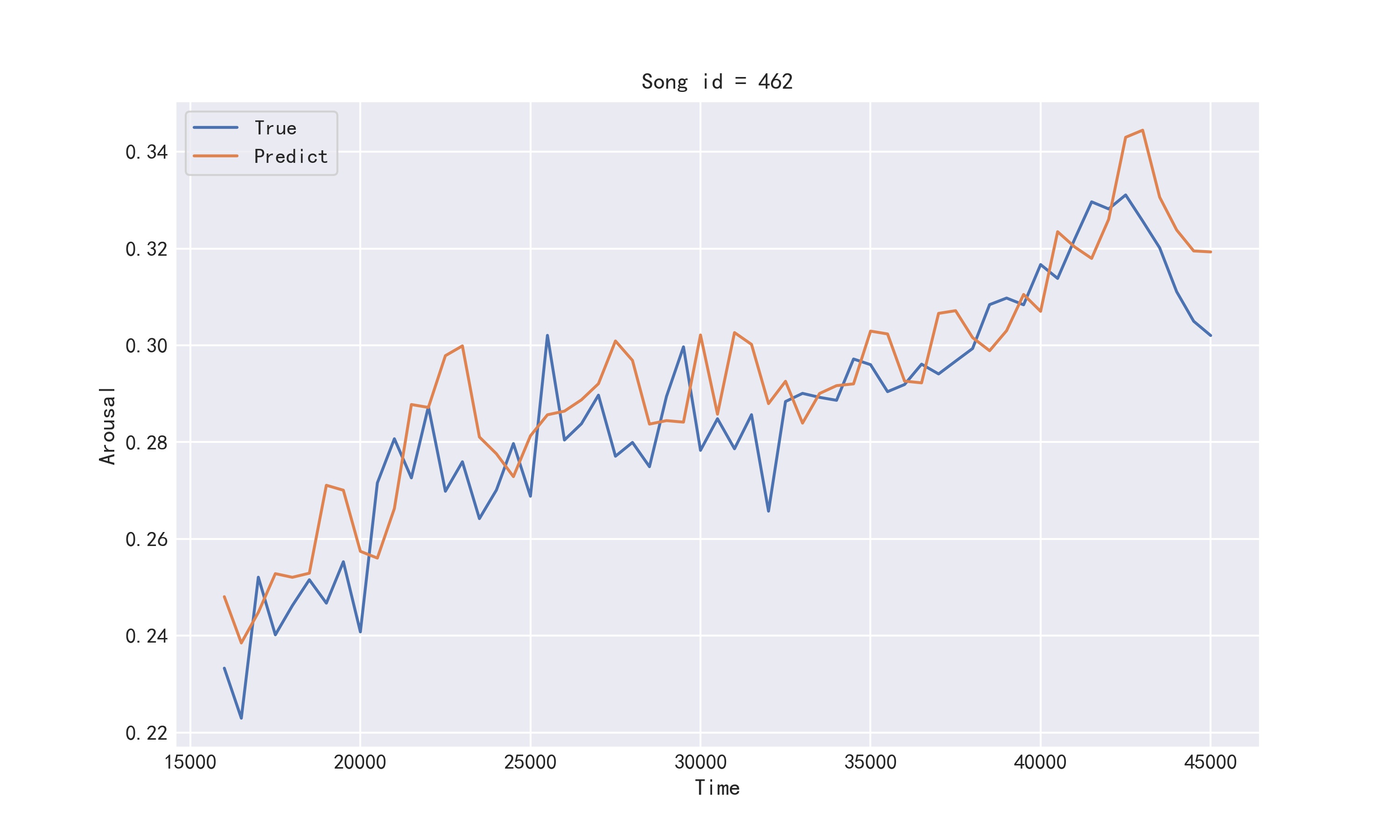

Supplement: S5 File — (ZIP) [file pone.0297712.s005.zip › All prediction results/prediction picture results(Emomusic_75)/song_id_462.jpg]

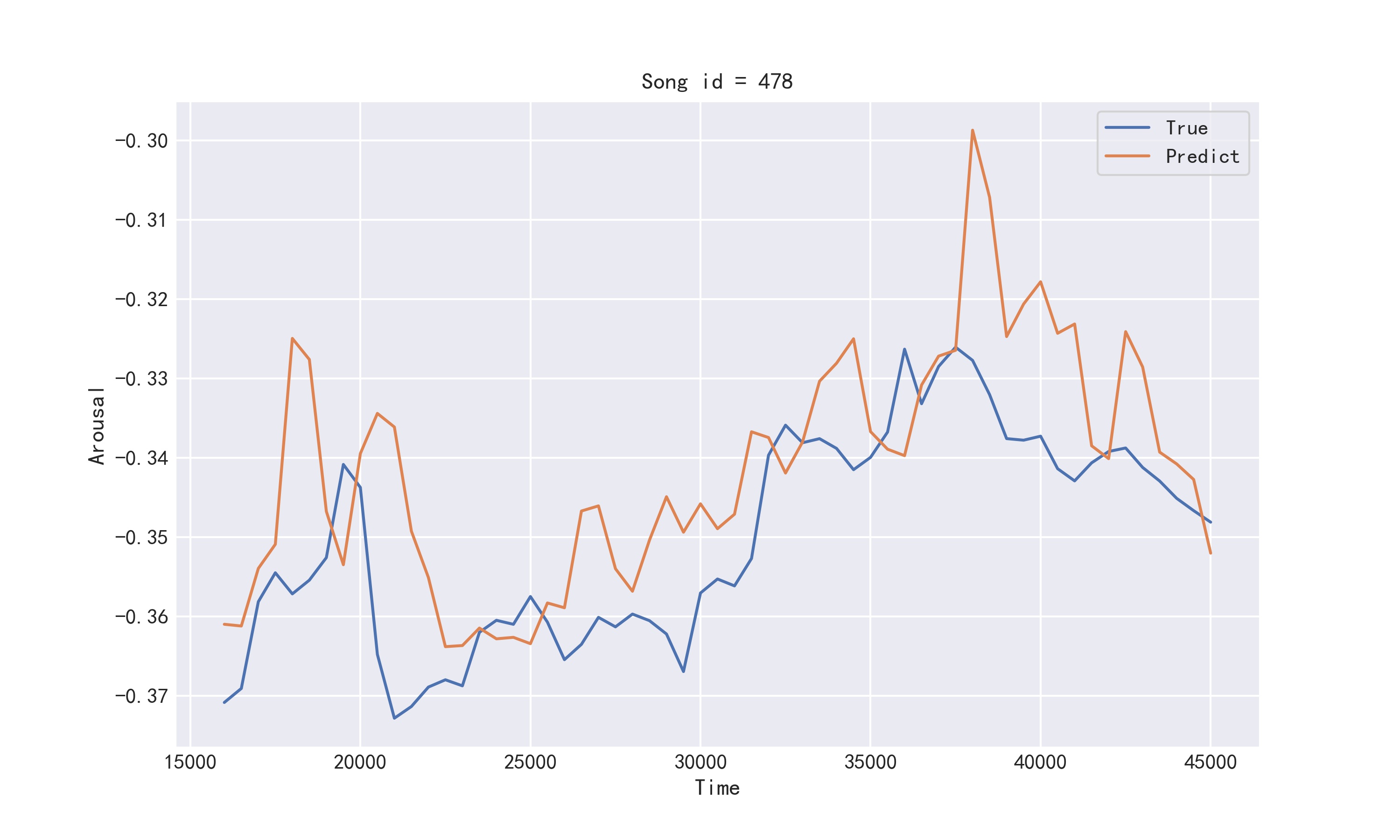

Supplement: S5 File — (ZIP) [file pone.0297712.s005.zip › All prediction results/prediction picture results(Emomusic_75)/song_id_478.jpg]

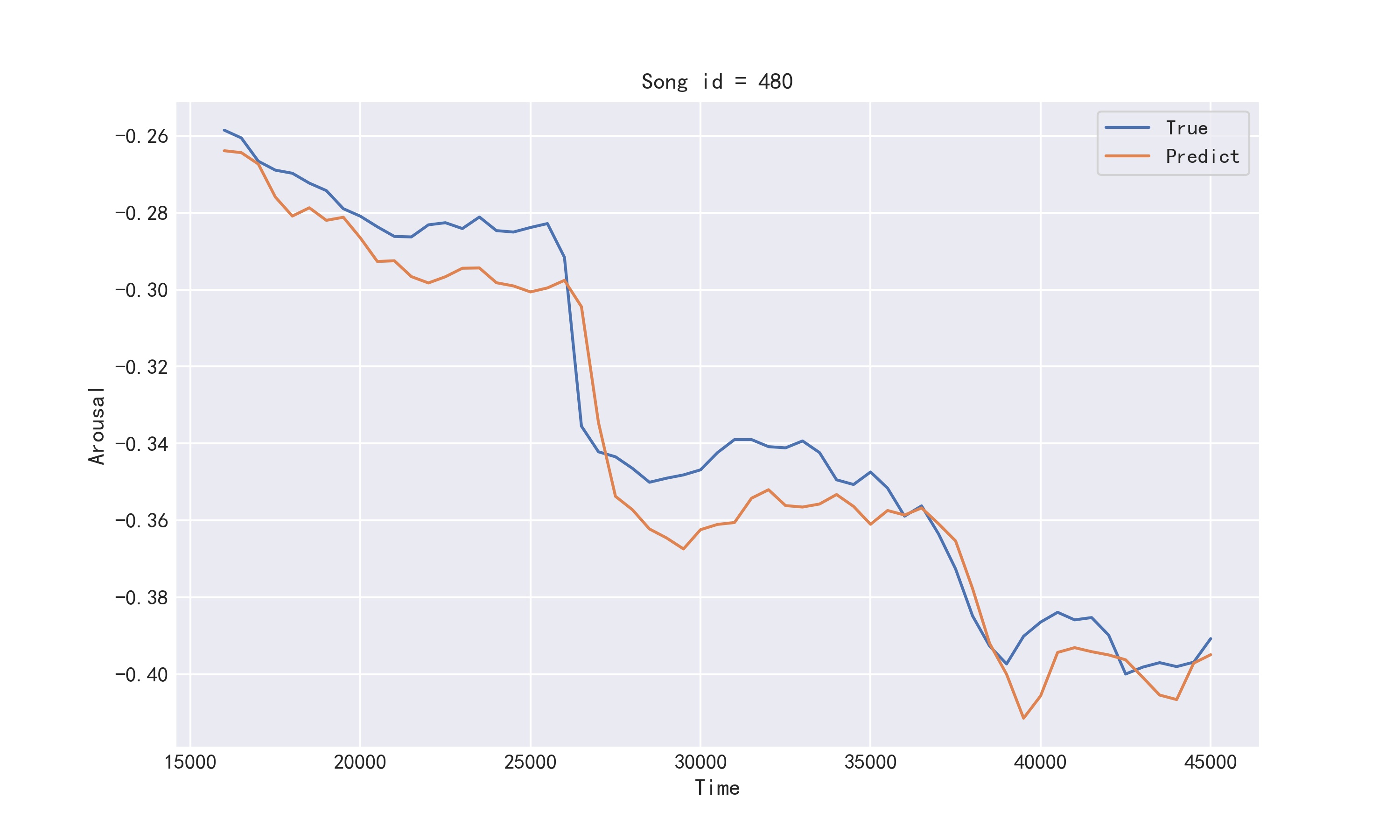

Supplement: S5 File — (ZIP) [file pone.0297712.s005.zip › All prediction results/prediction picture results(Emomusic_75)/song_id_480.jpg]

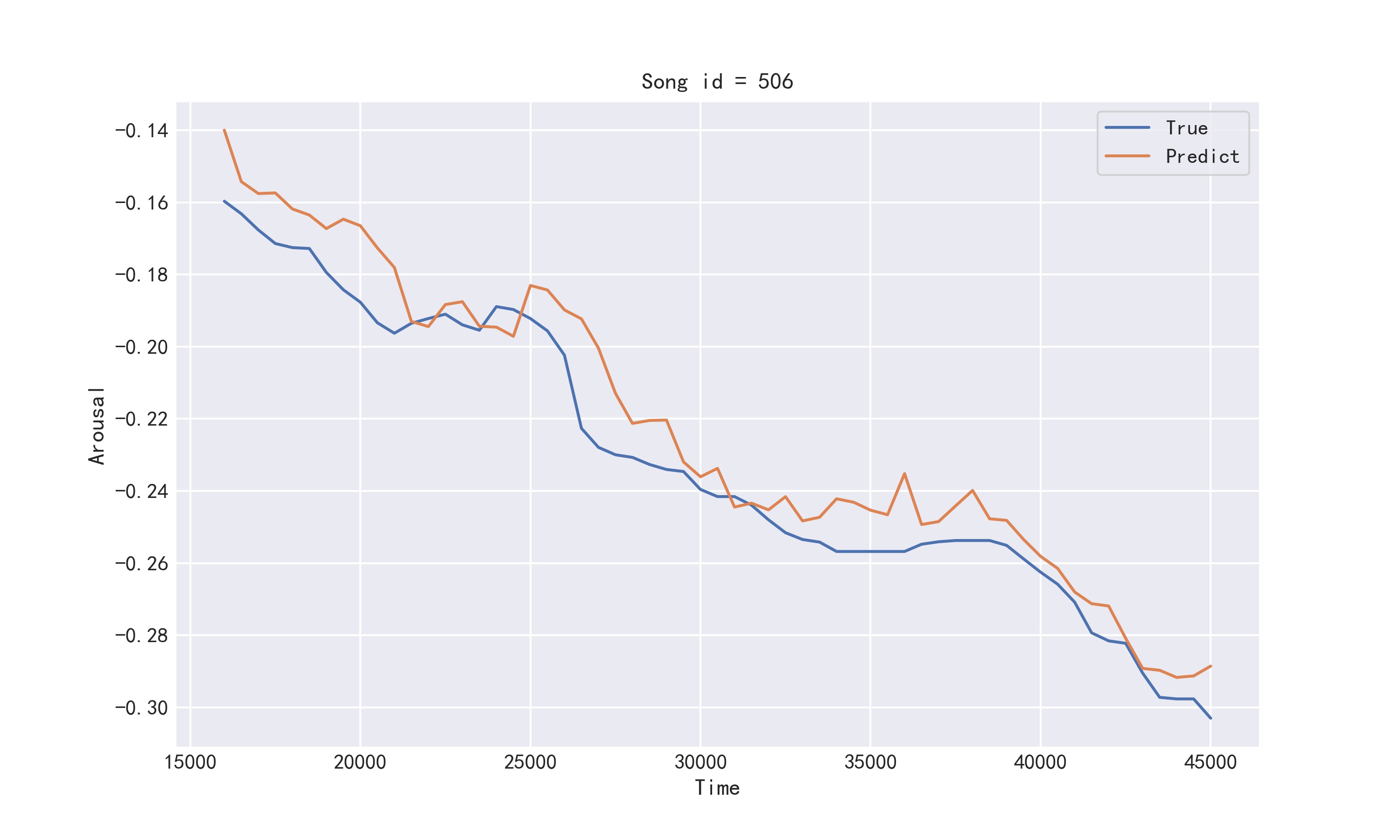

Supplement: S5 File — (ZIP) [file pone.0297712.s005.zip › All prediction results/prediction picture results(Emomusic_75)/song_id_506.jpg]

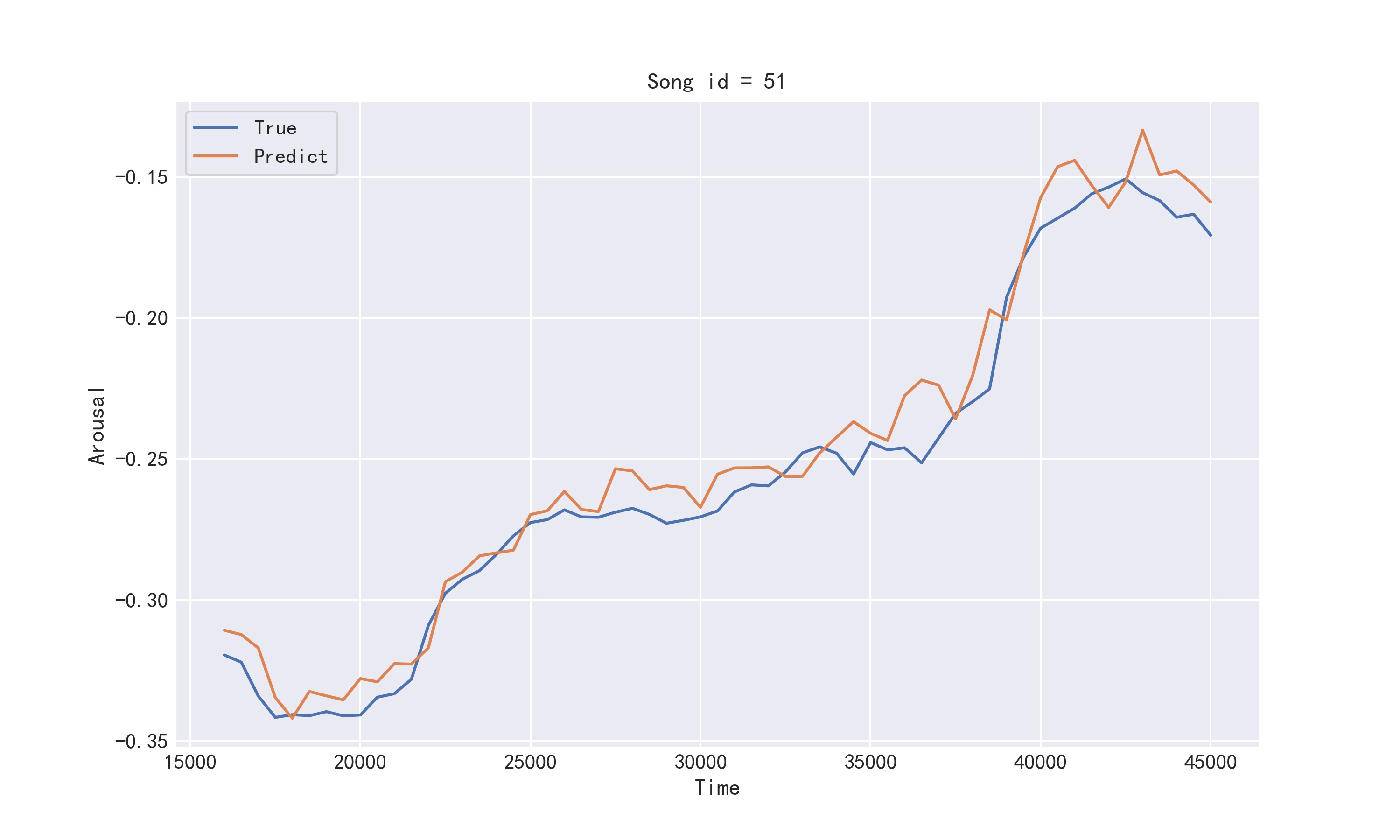

Supplement: S5 File — (ZIP) [file pone.0297712.s005.zip › All prediction results/prediction picture results(Emomusic_75)/song_id_51.jpg]

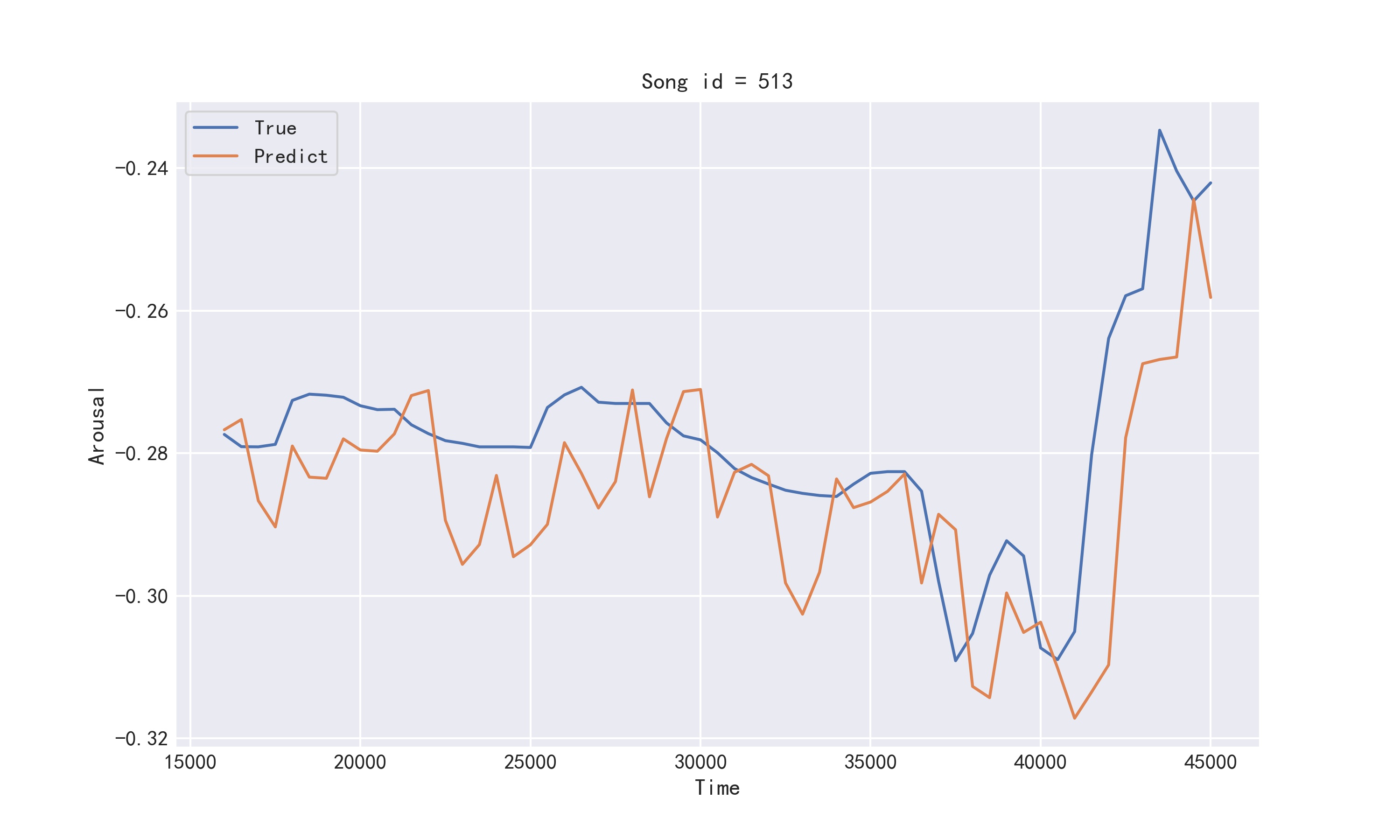

Supplement: S5 File — (ZIP) [file pone.0297712.s005.zip › All prediction results/prediction picture results(Emomusic_75)/song_id_513.jpg]

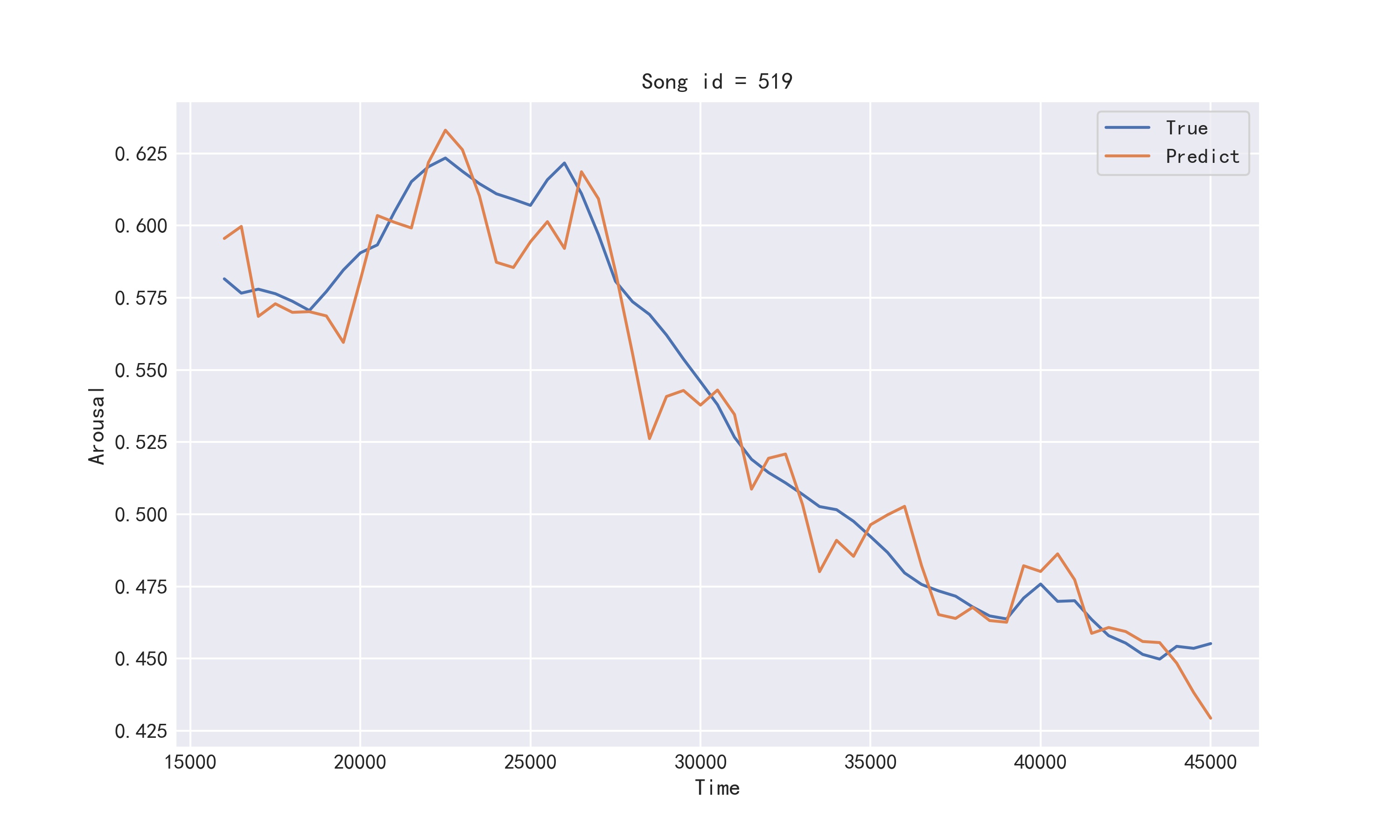

Supplement: S5 File — (ZIP) [file pone.0297712.s005.zip › All prediction results/prediction picture results(Emomusic_75)/song_id_519.jpg]

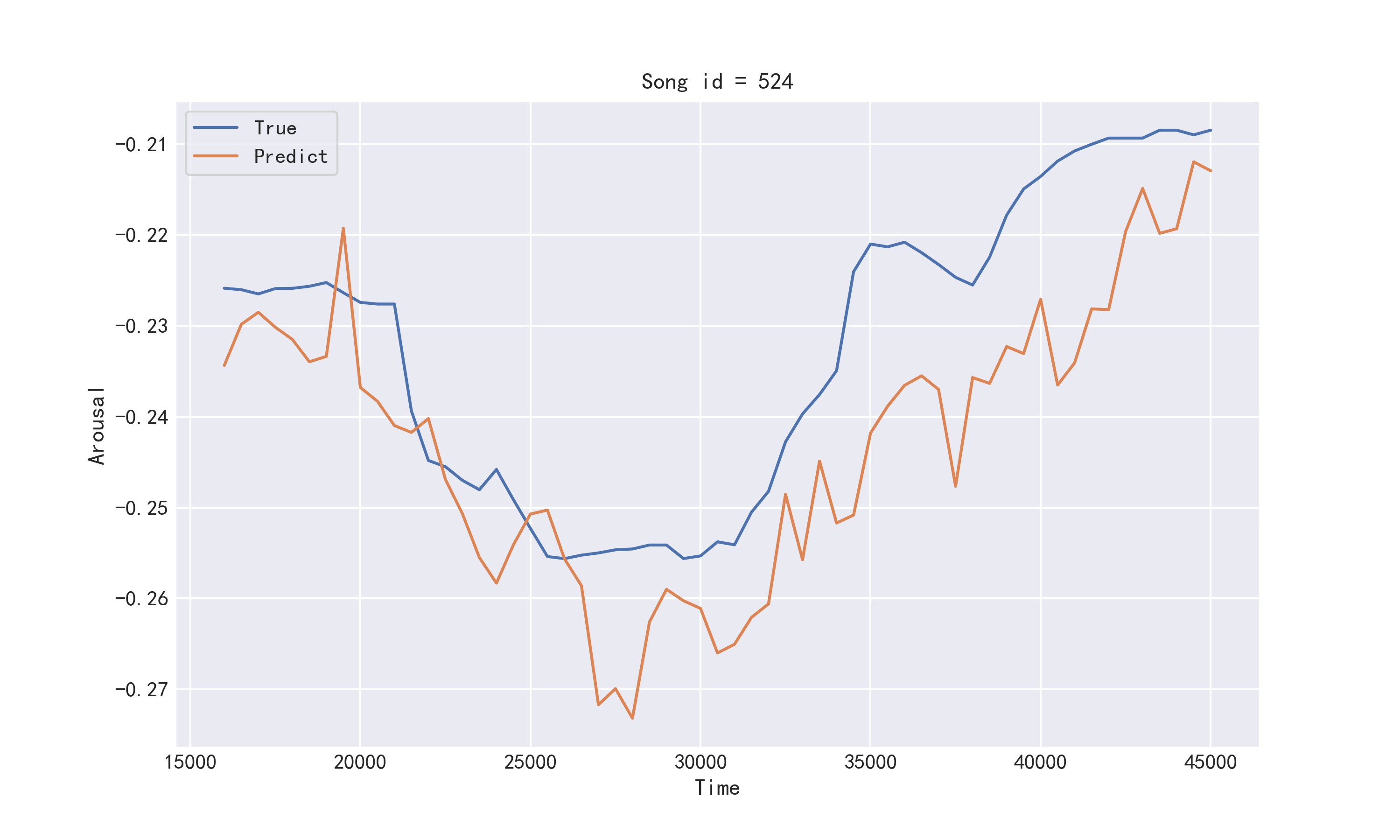

Supplement: S5 File — (ZIP) [file pone.0297712.s005.zip › All prediction results/prediction picture results(Emomusic_75)/song_id_524.jpg]

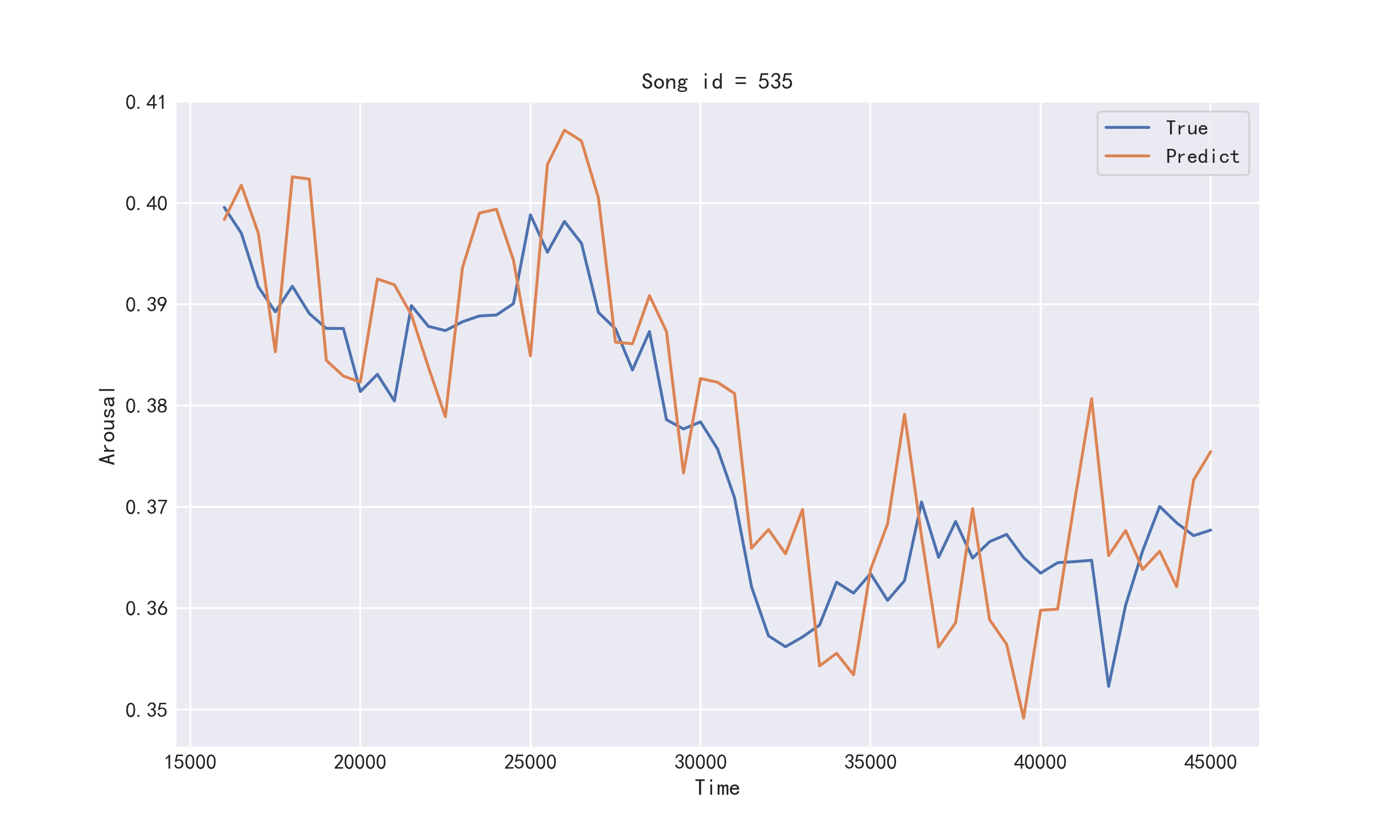

Supplement: S5 File — (ZIP) [file pone.0297712.s005.zip › All prediction results/prediction picture results(Emomusic_75)/song_id_535.jpg]

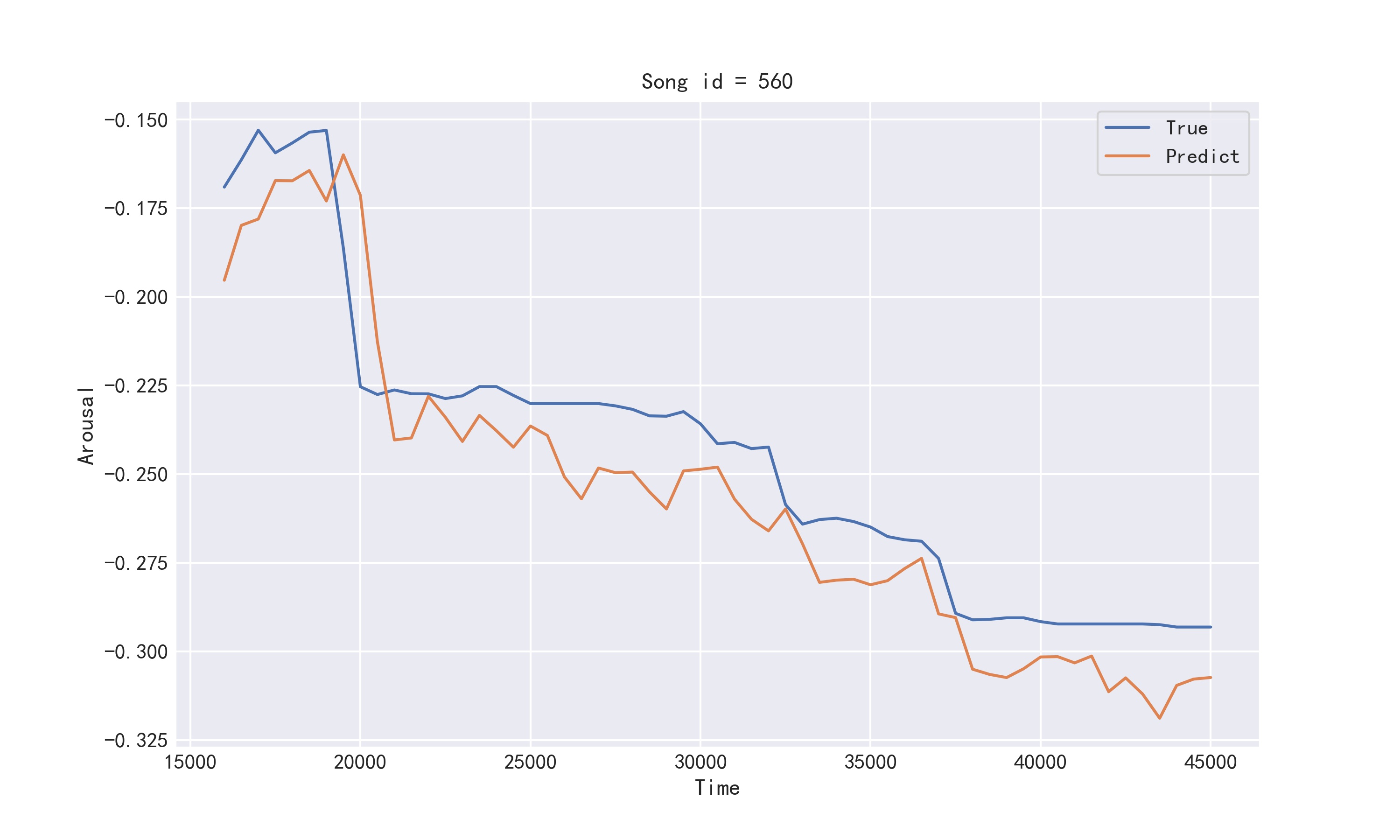

Supplement: S5 File — (ZIP) [file pone.0297712.s005.zip › All prediction results/prediction picture results(Emomusic_75)/song_id_560.jpg]

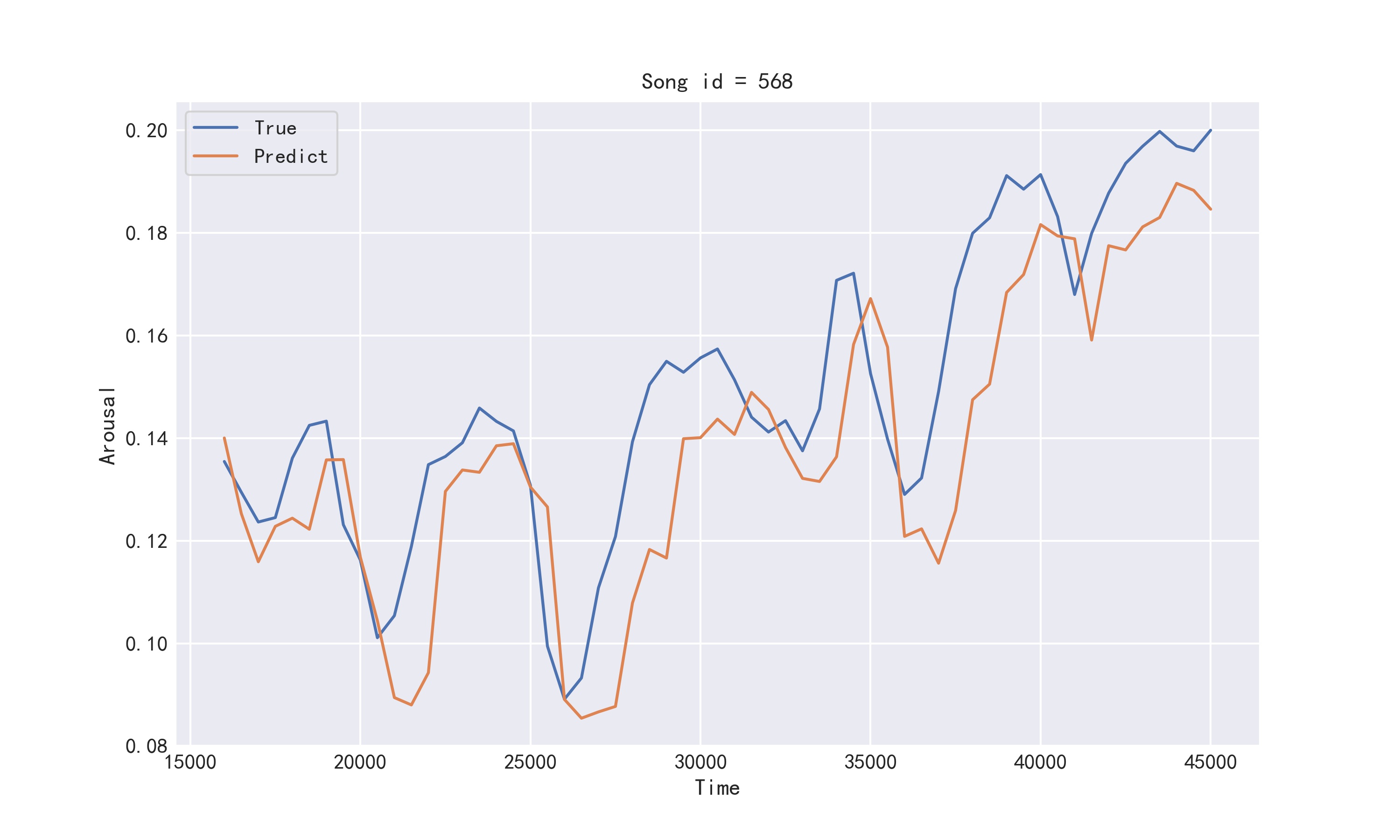

Supplement: S5 File — (ZIP) [file pone.0297712.s005.zip › All prediction results/prediction picture results(Emomusic_75)/song_id_568.jpg]

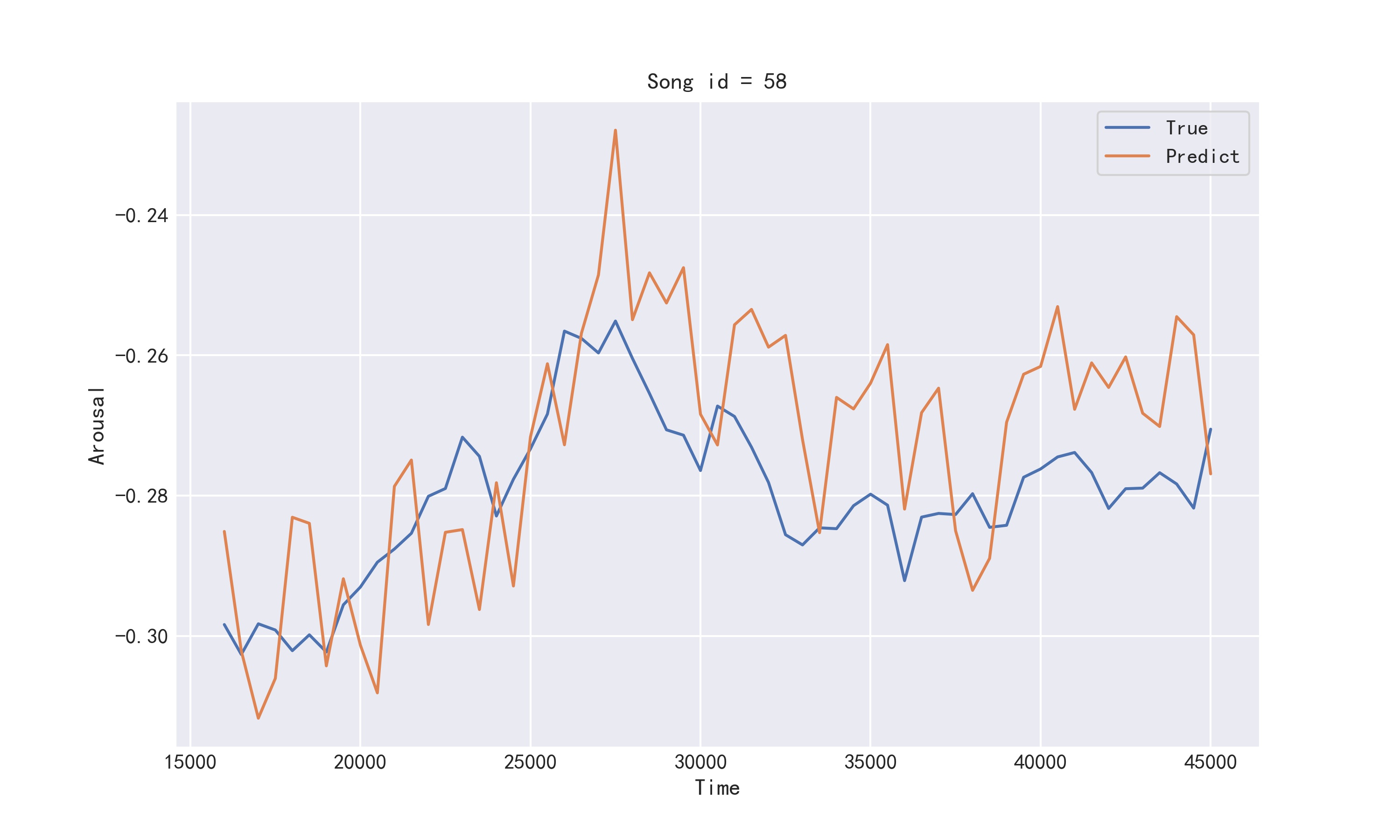

Supplement: S5 File — (ZIP) [file pone.0297712.s005.zip › All prediction results/prediction picture results(Emomusic_75)/song_id_58.jpg]

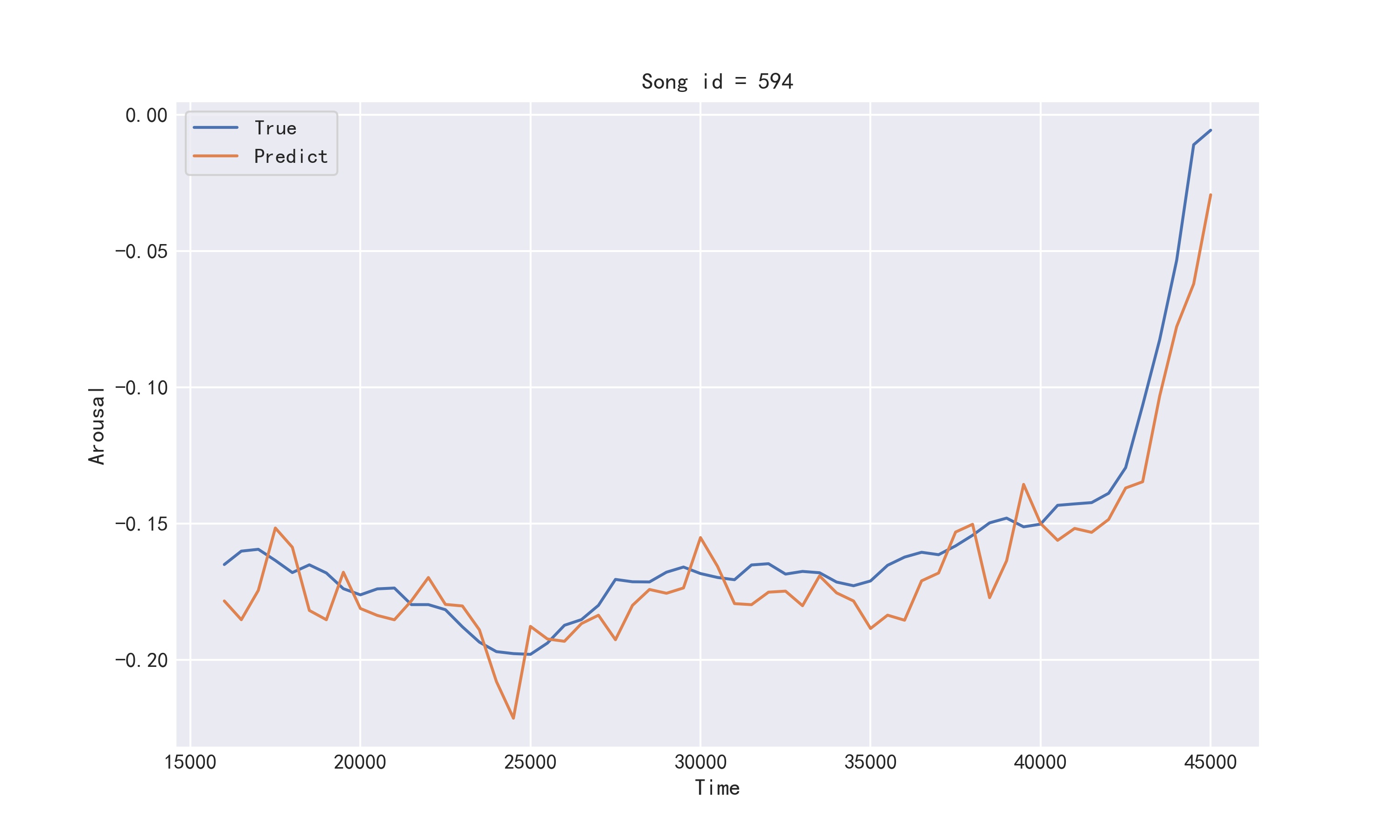

Supplement: S5 File — (ZIP) [file pone.0297712.s005.zip › All prediction results/prediction picture results(Emomusic_75)/song_id_594.jpg]

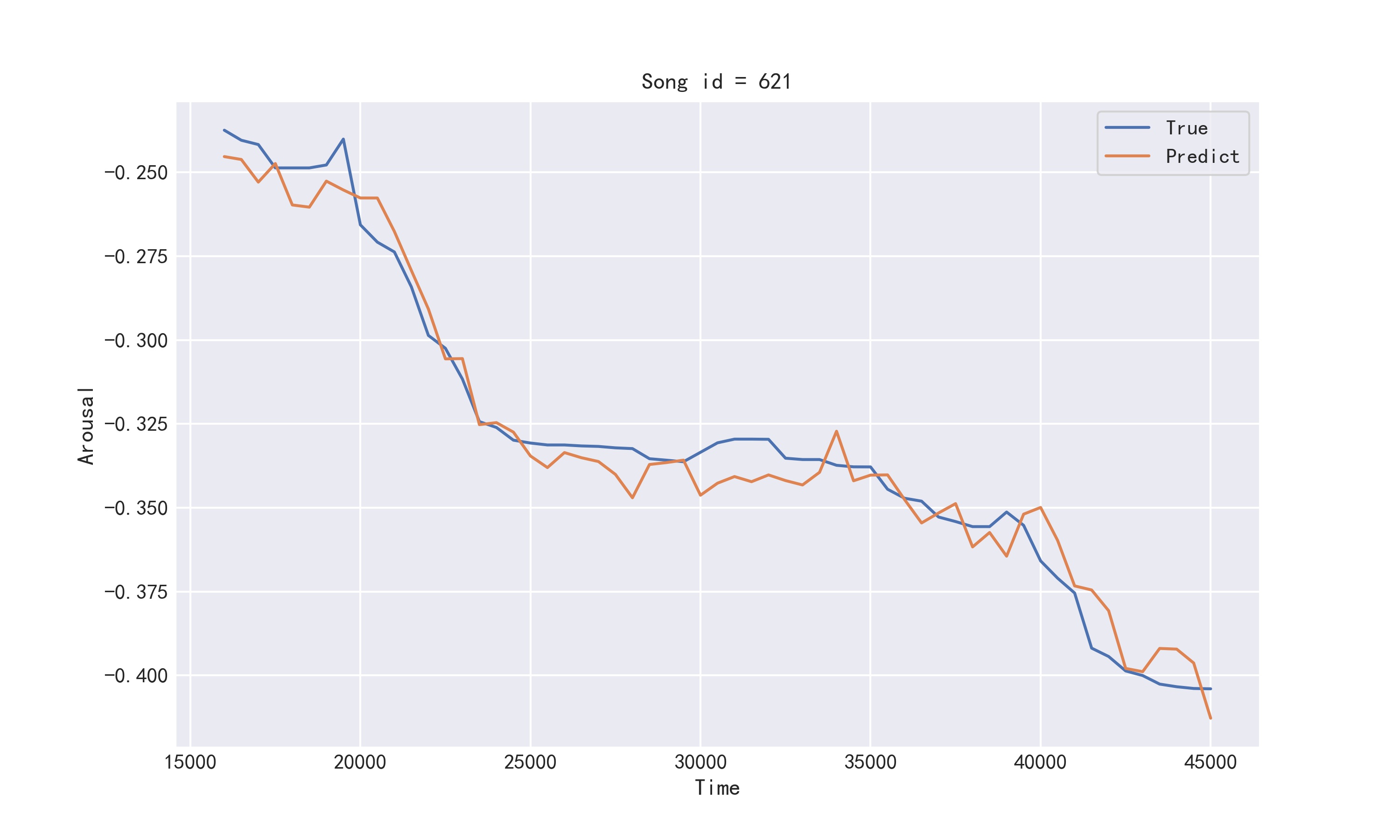

Supplement: S5 File — (ZIP) [file pone.0297712.s005.zip › All prediction results/prediction picture results(Emomusic_75)/song_id_621.jpg]

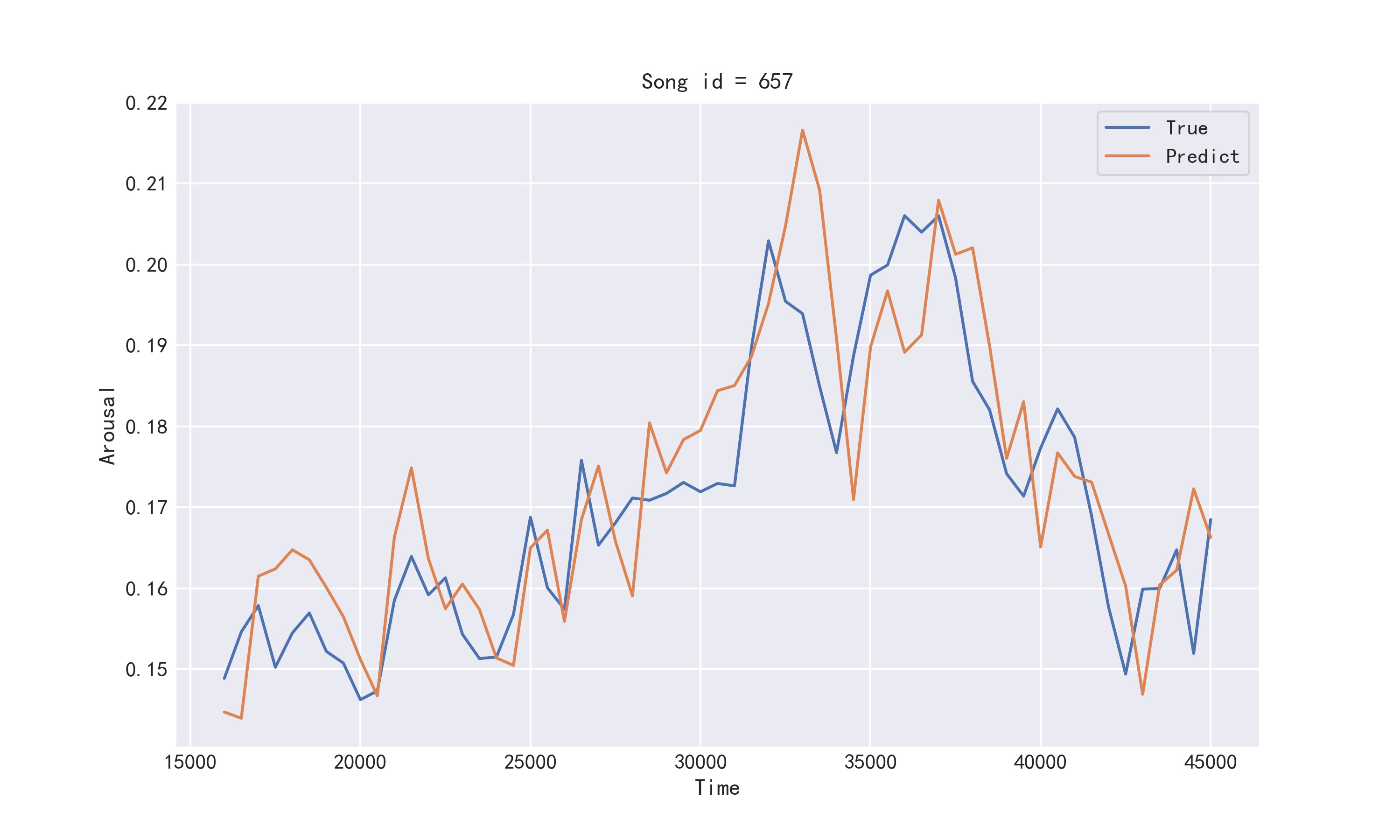

Supplement: S5 File — (ZIP) [file pone.0297712.s005.zip › All prediction results/prediction picture results(Emomusic_75)/song_id_657.jpg]

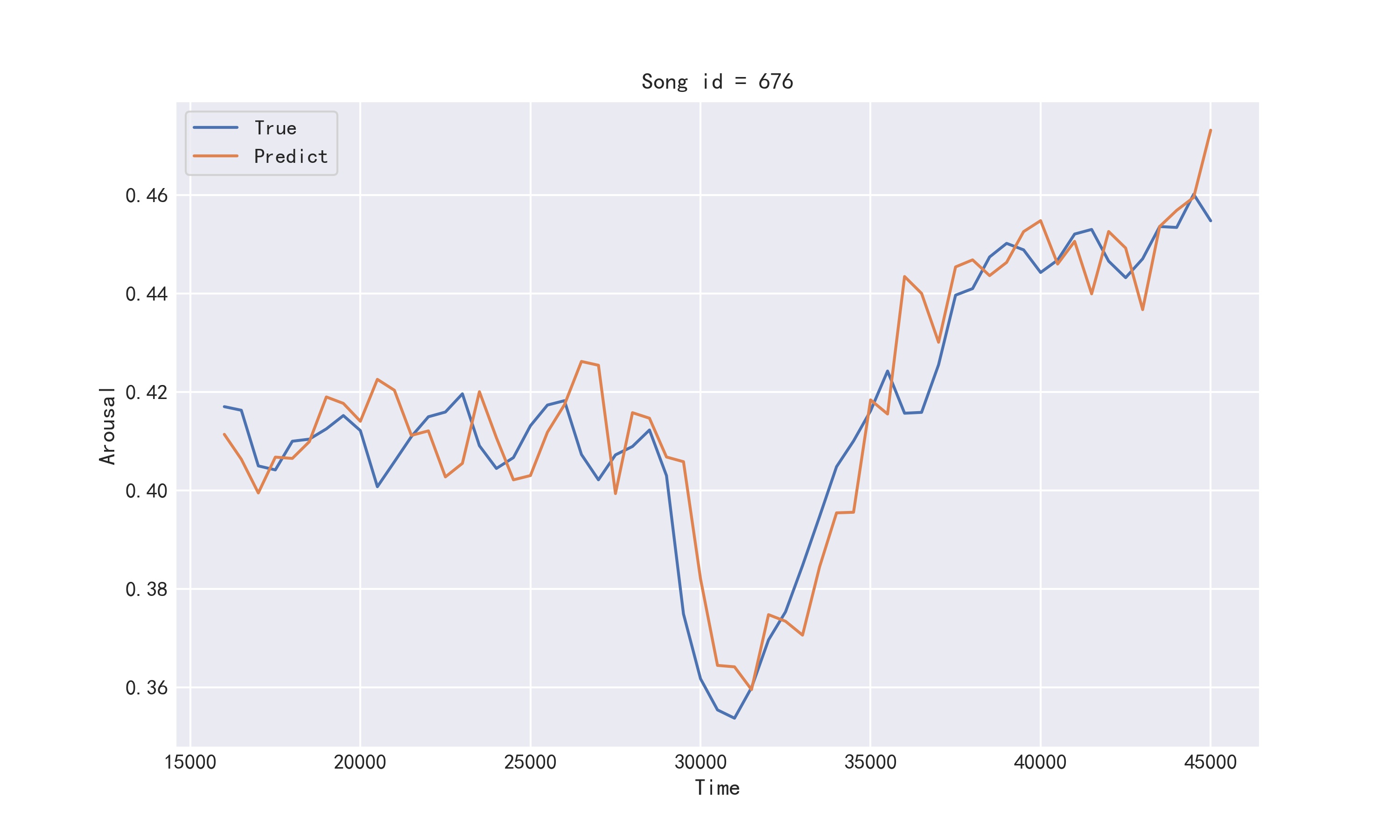

Supplement: S5 File — (ZIP) [file pone.0297712.s005.zip › All prediction results/prediction picture results(Emomusic_75)/song_id_676.jpg]

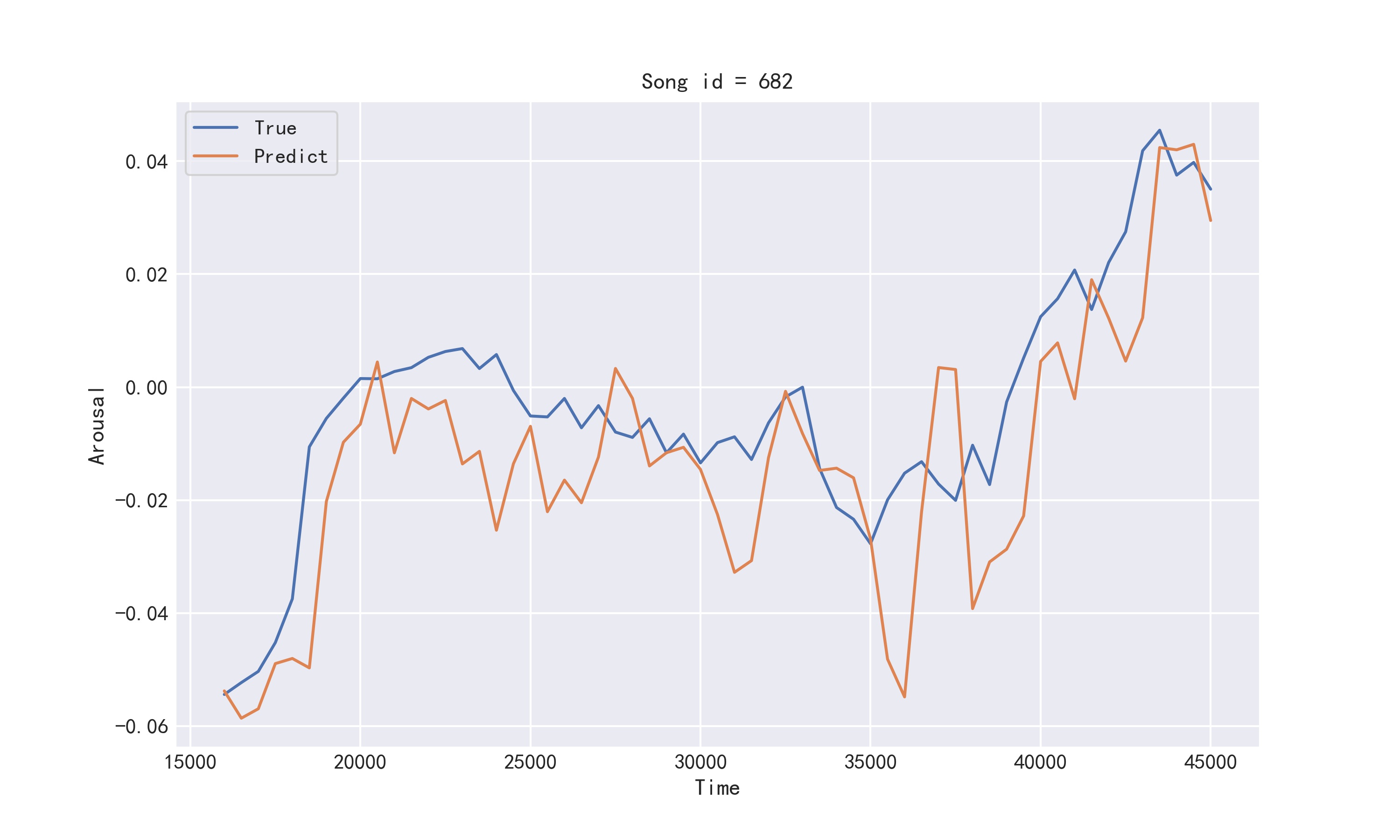

Supplement: S5 File — (ZIP) [file pone.0297712.s005.zip › All prediction results/prediction picture results(Emomusic_75)/song_id_682.jpg]

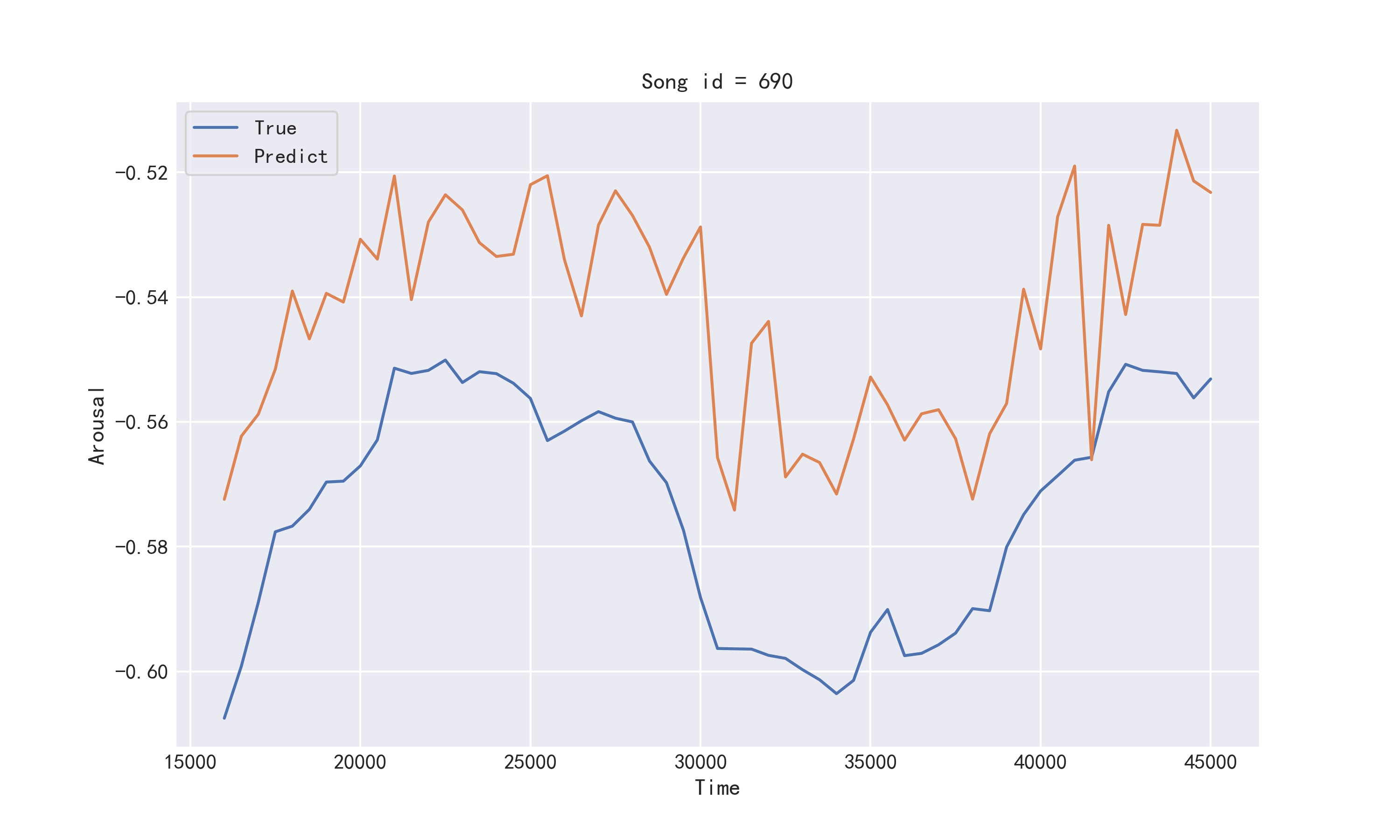

Supplement: S5 File — (ZIP) [file pone.0297712.s005.zip › All prediction results/prediction picture results(Emomusic_75)/song_id_690.jpg]

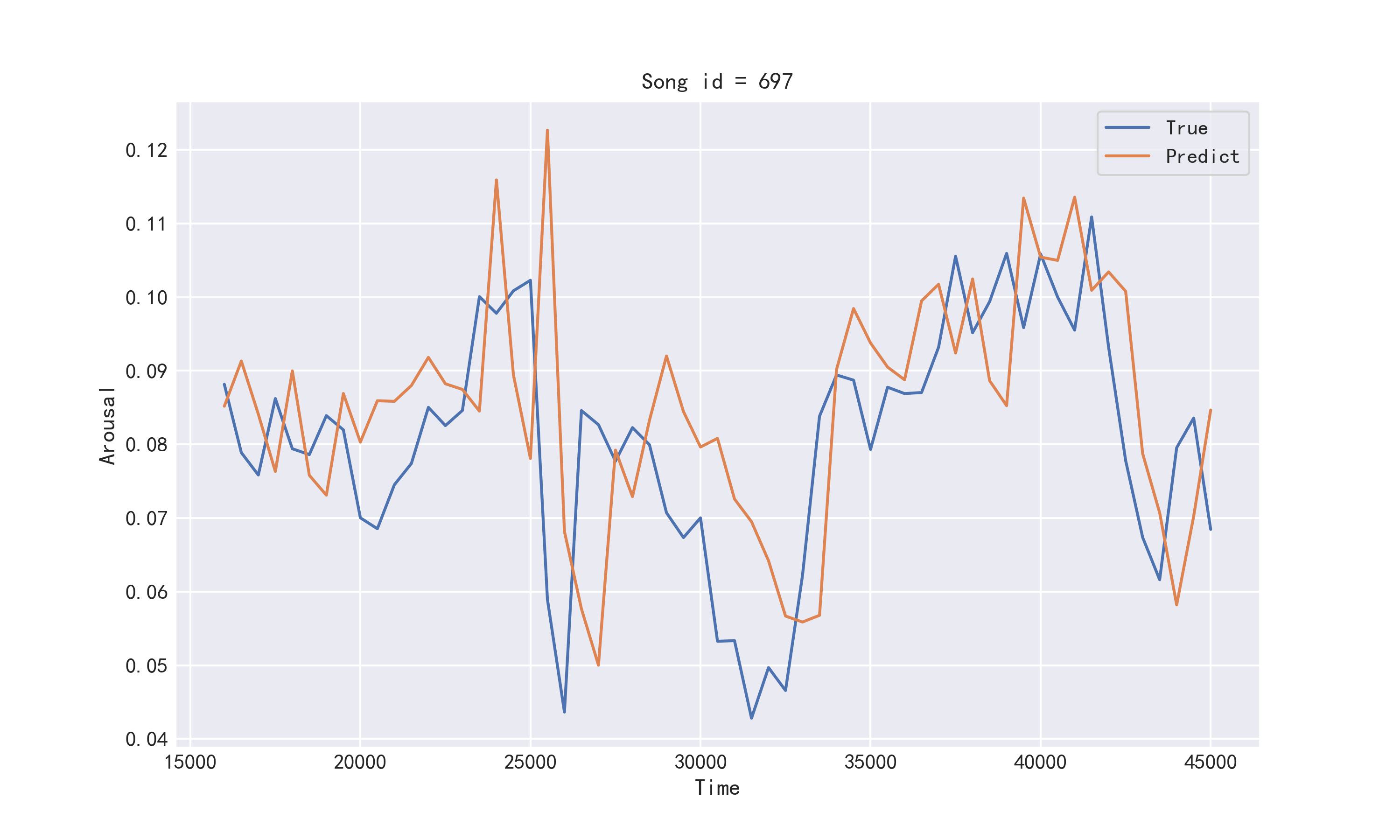

Supplement: S5 File — (ZIP) [file pone.0297712.s005.zip › All prediction results/prediction picture results(Emomusic_75)/song_id_697.jpg]

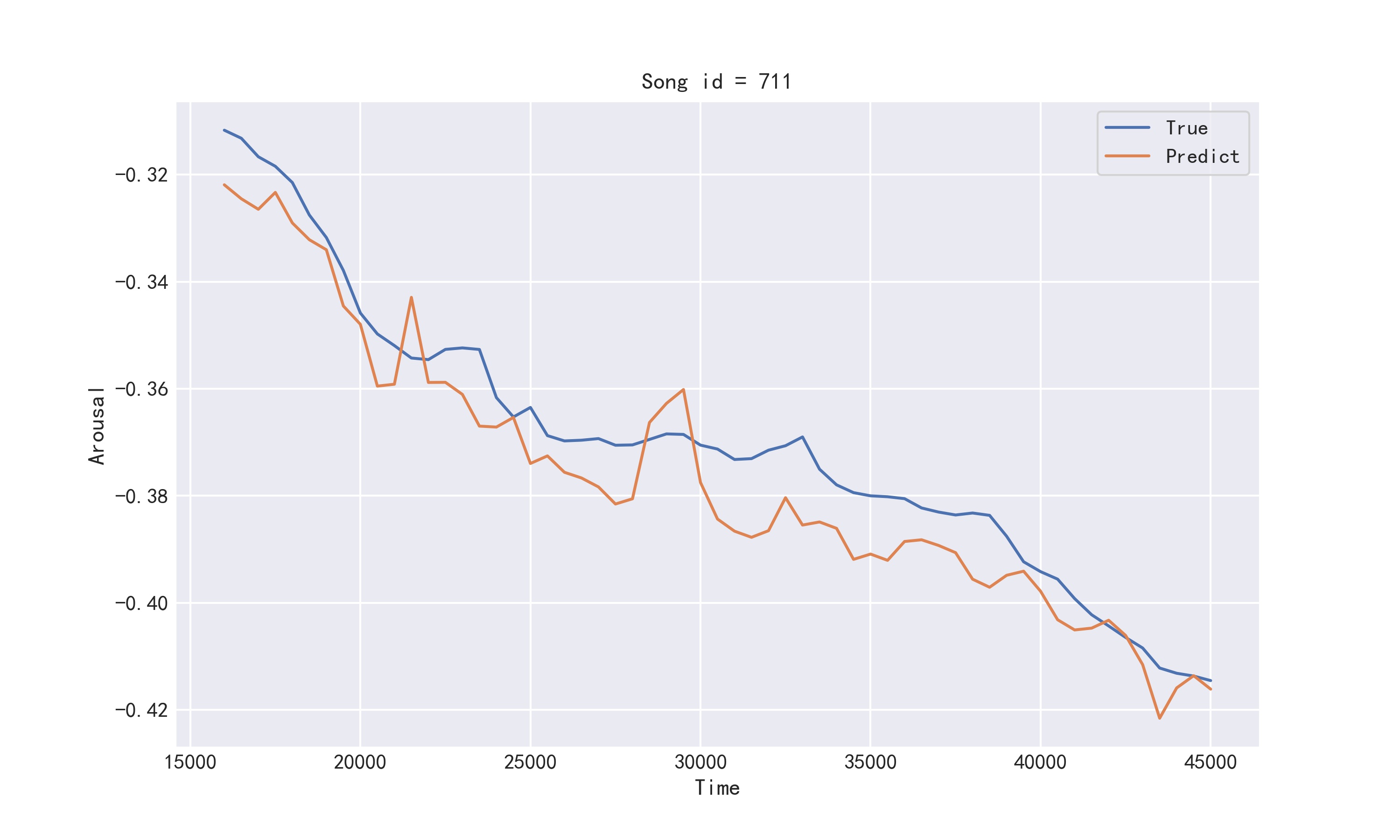

Supplement: S5 File — (ZIP) [file pone.0297712.s005.zip › All prediction results/prediction picture results(Emomusic_75)/song_id_711.jpg]

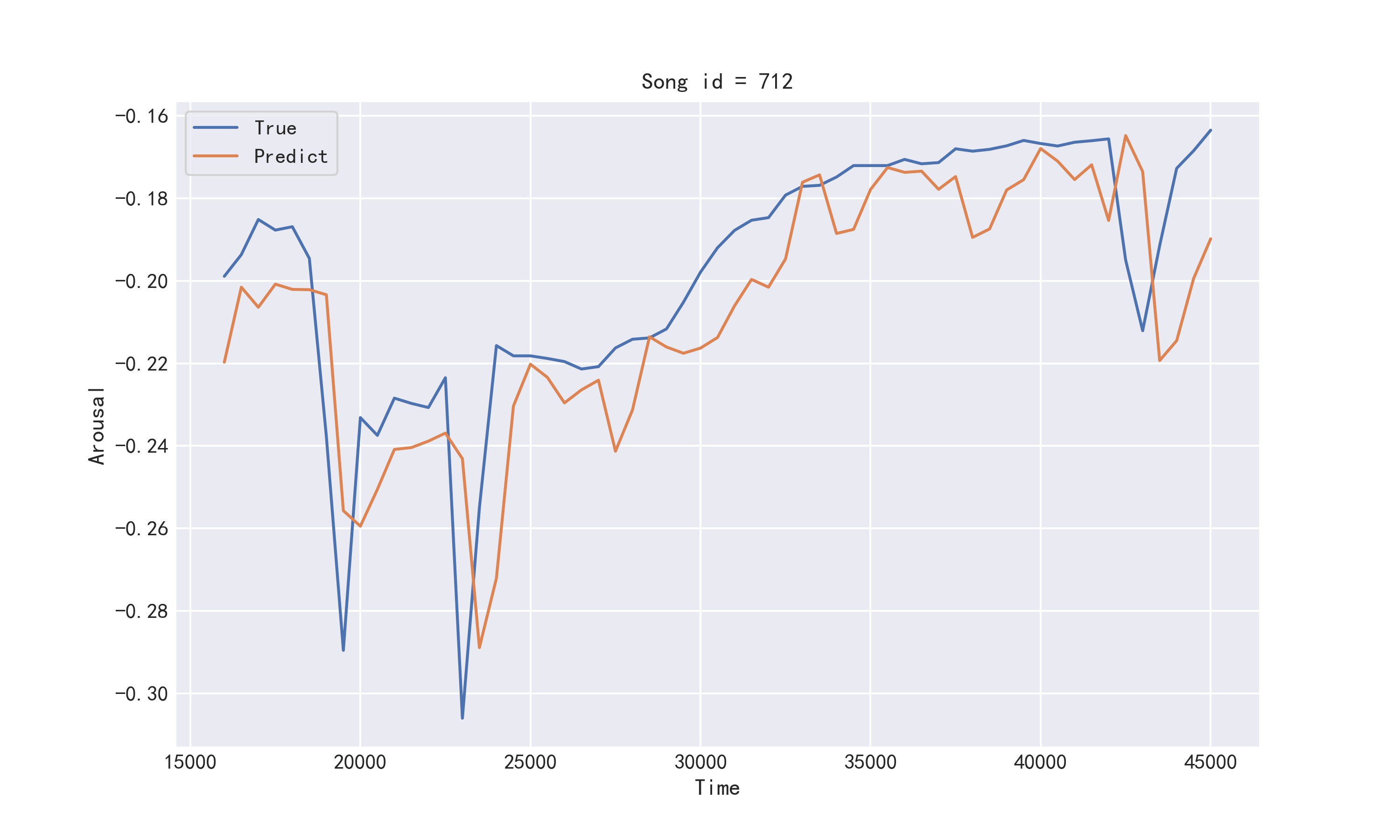

Supplement: S5 File — (ZIP) [file pone.0297712.s005.zip › All prediction results/prediction picture results(Emomusic_75)/song_id_712.jpg]

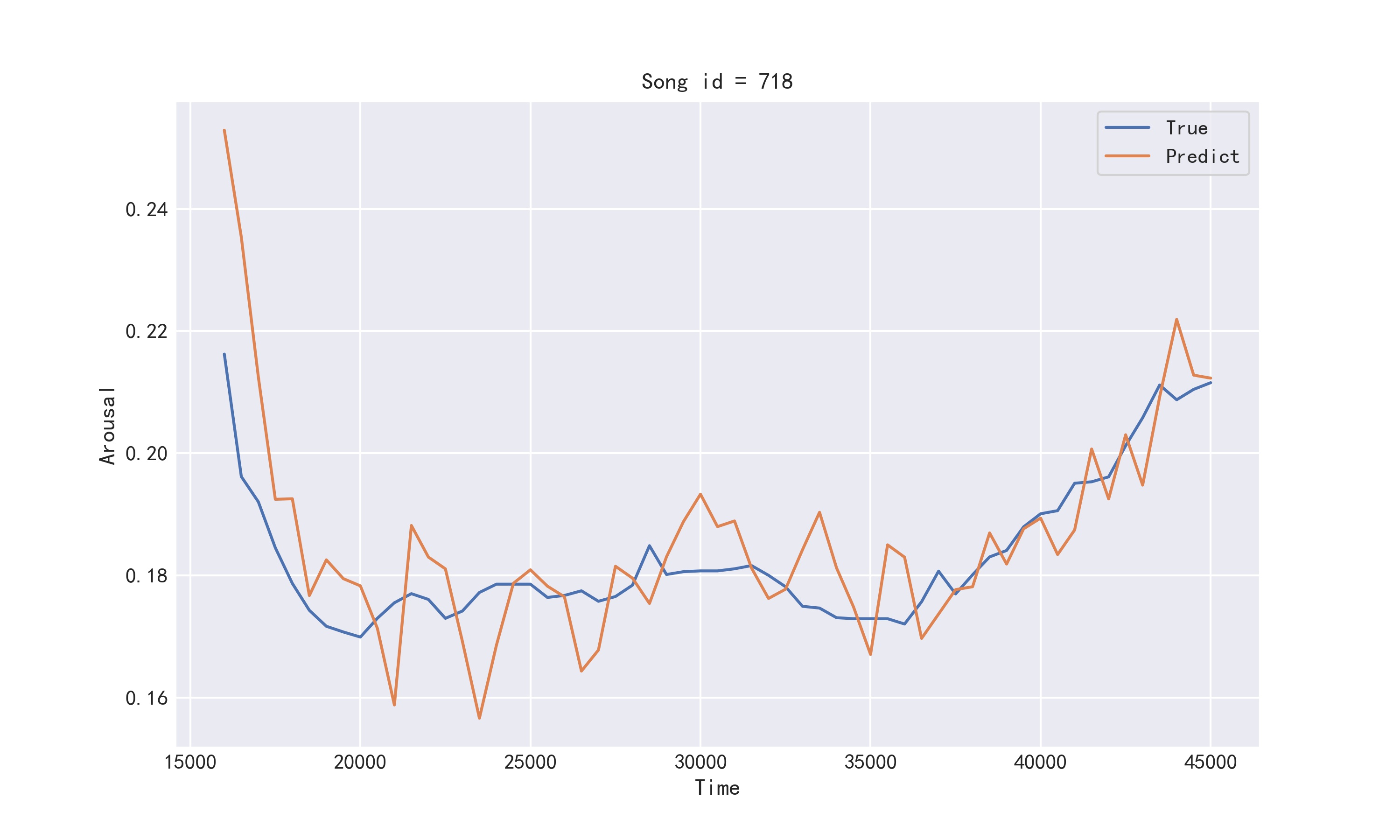

Supplement: S5 File — (ZIP) [file pone.0297712.s005.zip › All prediction results/prediction picture results(Emomusic_75)/song_id_718.jpg]

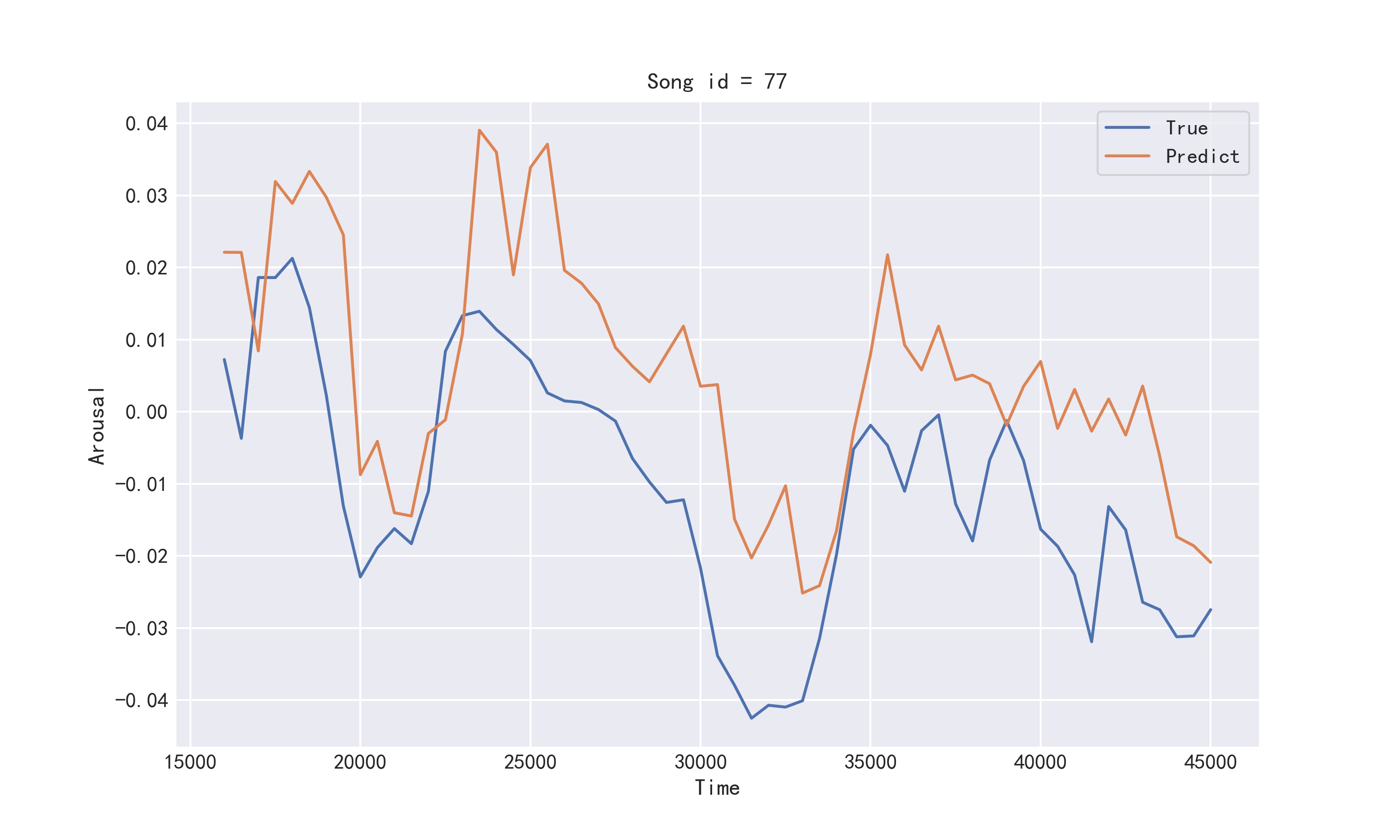

Supplement: S5 File — (ZIP) [file pone.0297712.s005.zip › All prediction results/prediction picture results(Emomusic_75)/song_id_77.jpg]

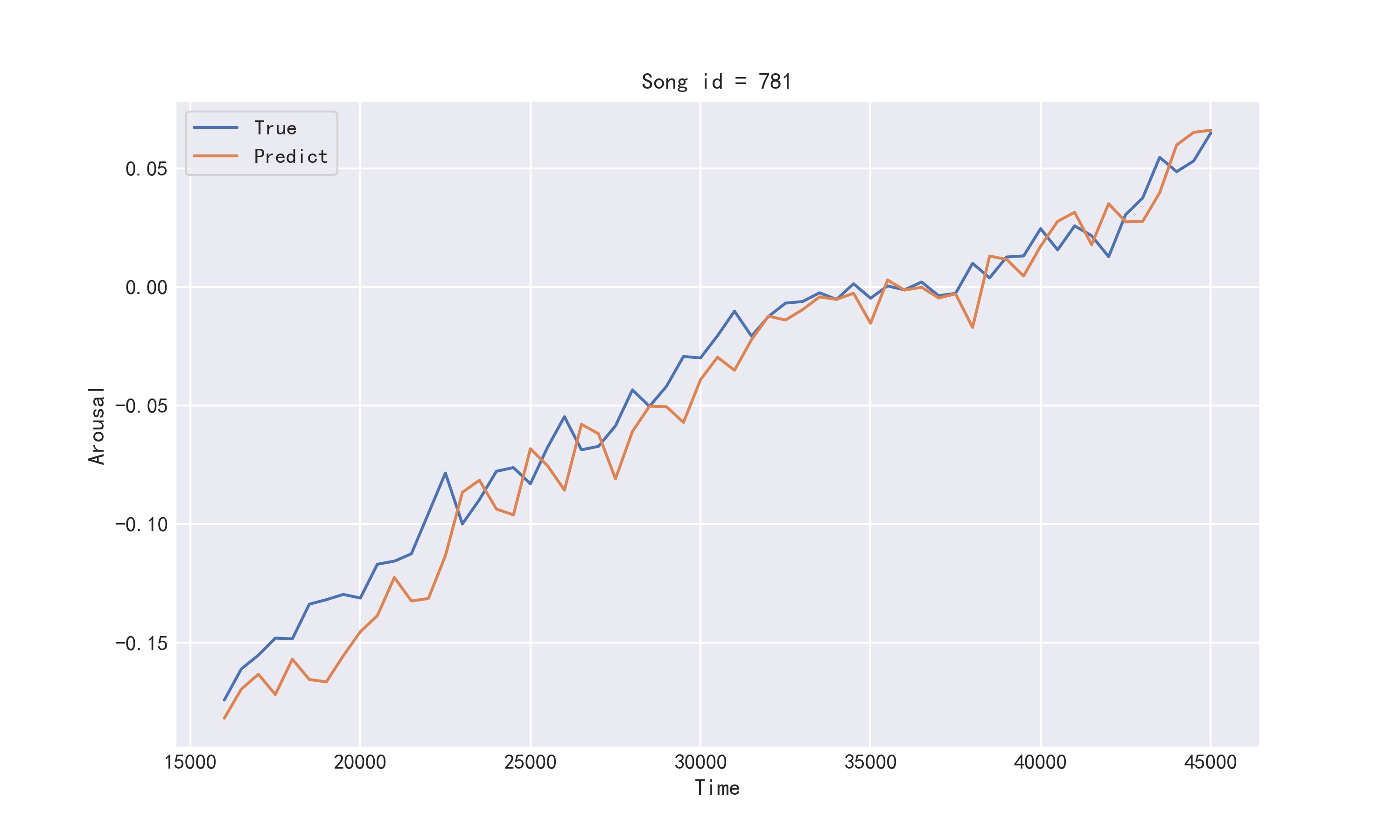

Supplement: S5 File — (ZIP) [file pone.0297712.s005.zip › All prediction results/prediction picture results(Emomusic_75)/song_id_781.jpg]

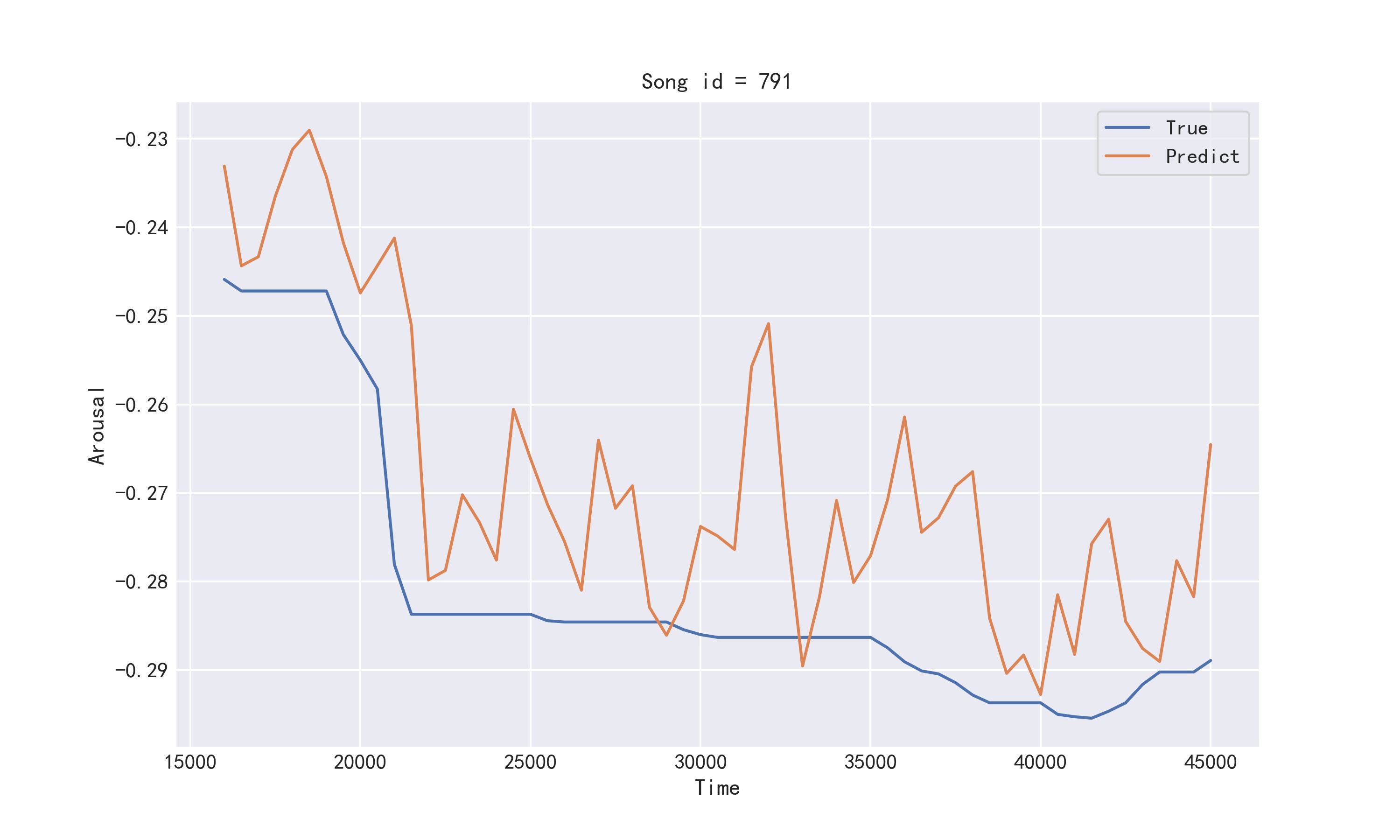

Supplement: S5 File — (ZIP) [file pone.0297712.s005.zip › All prediction results/prediction picture results(Emomusic_75)/song_id_791.jpg]

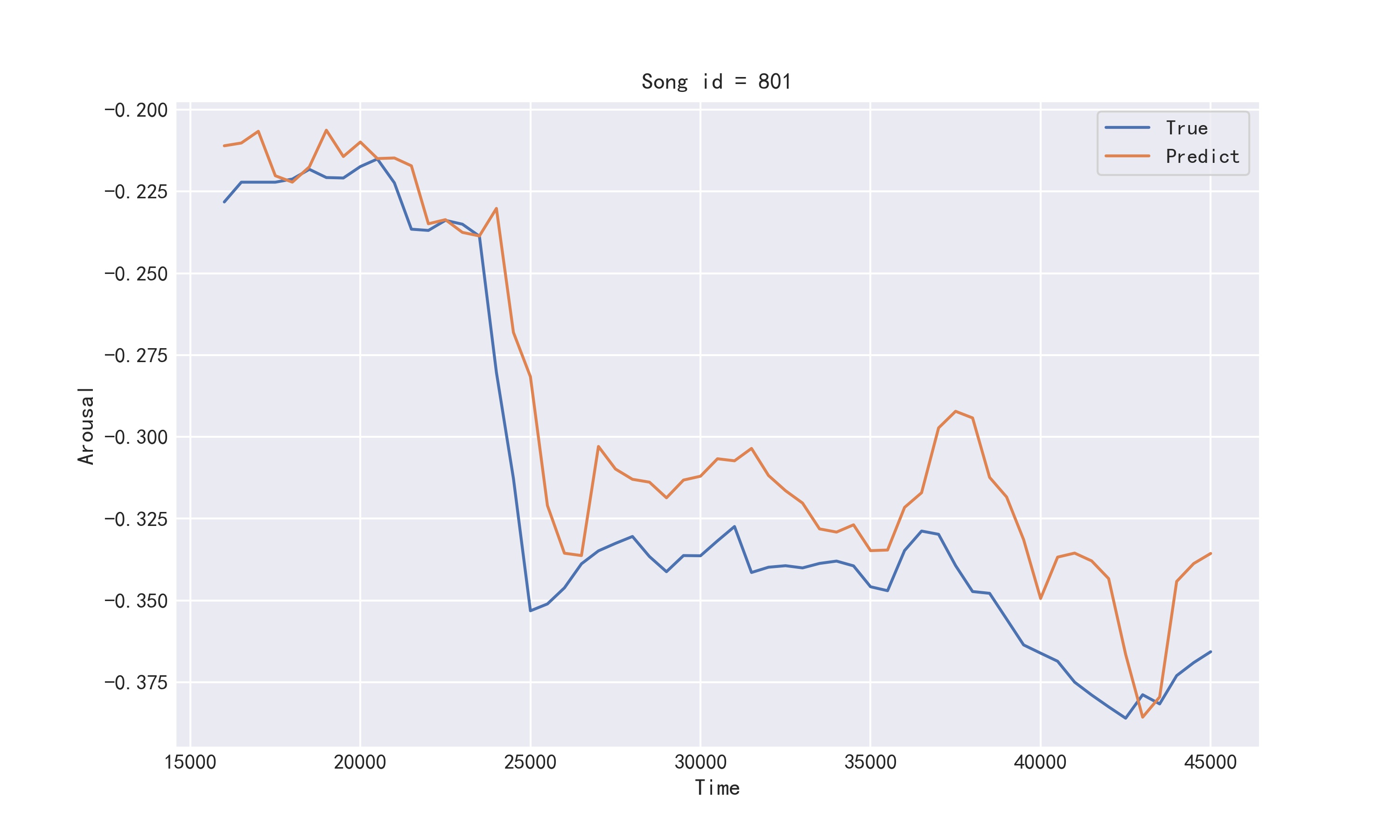

Supplement: S5 File — (ZIP) [file pone.0297712.s005.zip › All prediction results/prediction picture results(Emomusic_75)/song_id_801.jpg]

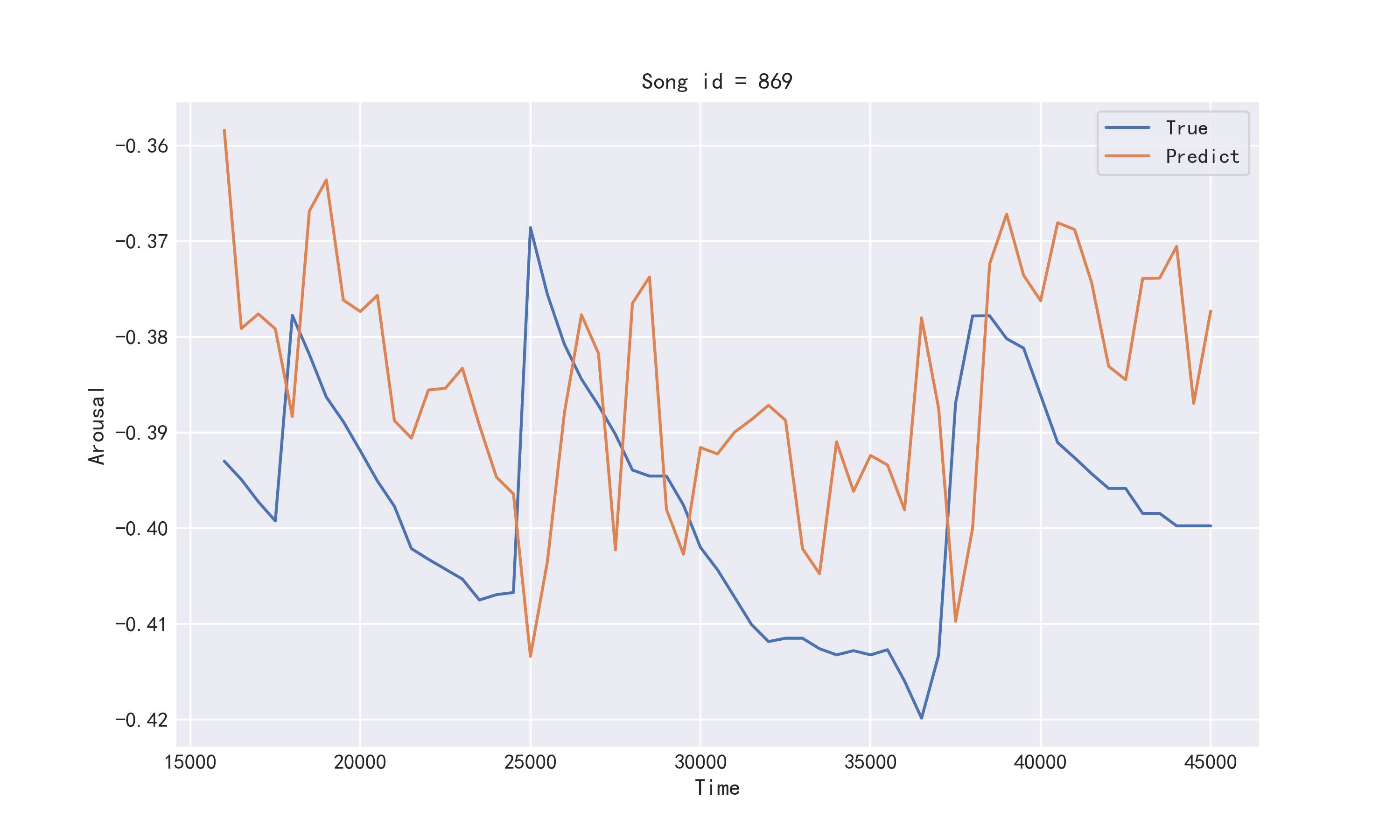

Supplement: S5 File — (ZIP) [file pone.0297712.s005.zip › All prediction results/prediction picture results(Emomusic_75)/song_id_869.jpg]

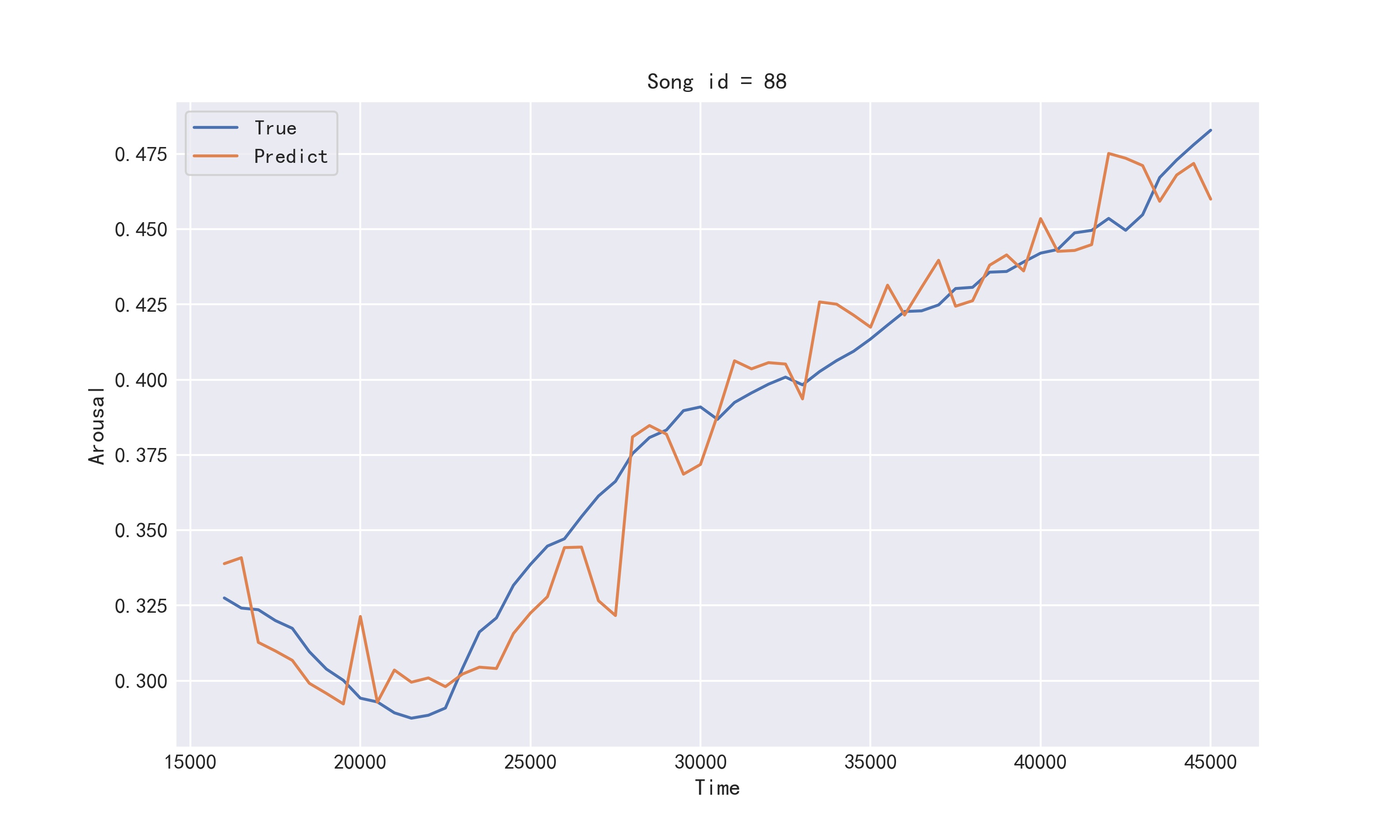

Supplement: S5 File — (ZIP) [file pone.0297712.s005.zip › All prediction results/prediction picture results(Emomusic_75)/song_id_88.jpg]

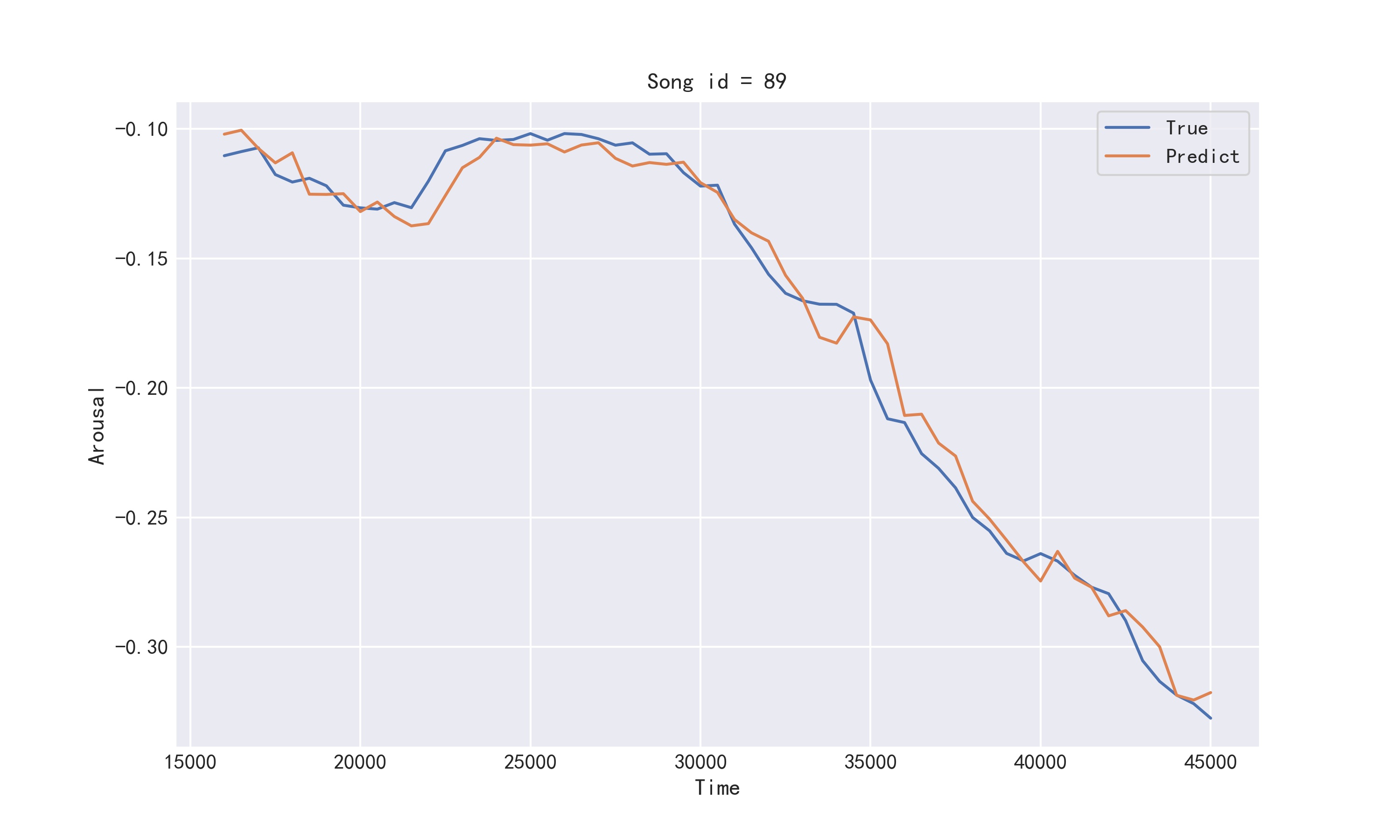

Supplement: S5 File — (ZIP) [file pone.0297712.s005.zip › All prediction results/prediction picture results(Emomusic_75)/song_id_89.jpg]

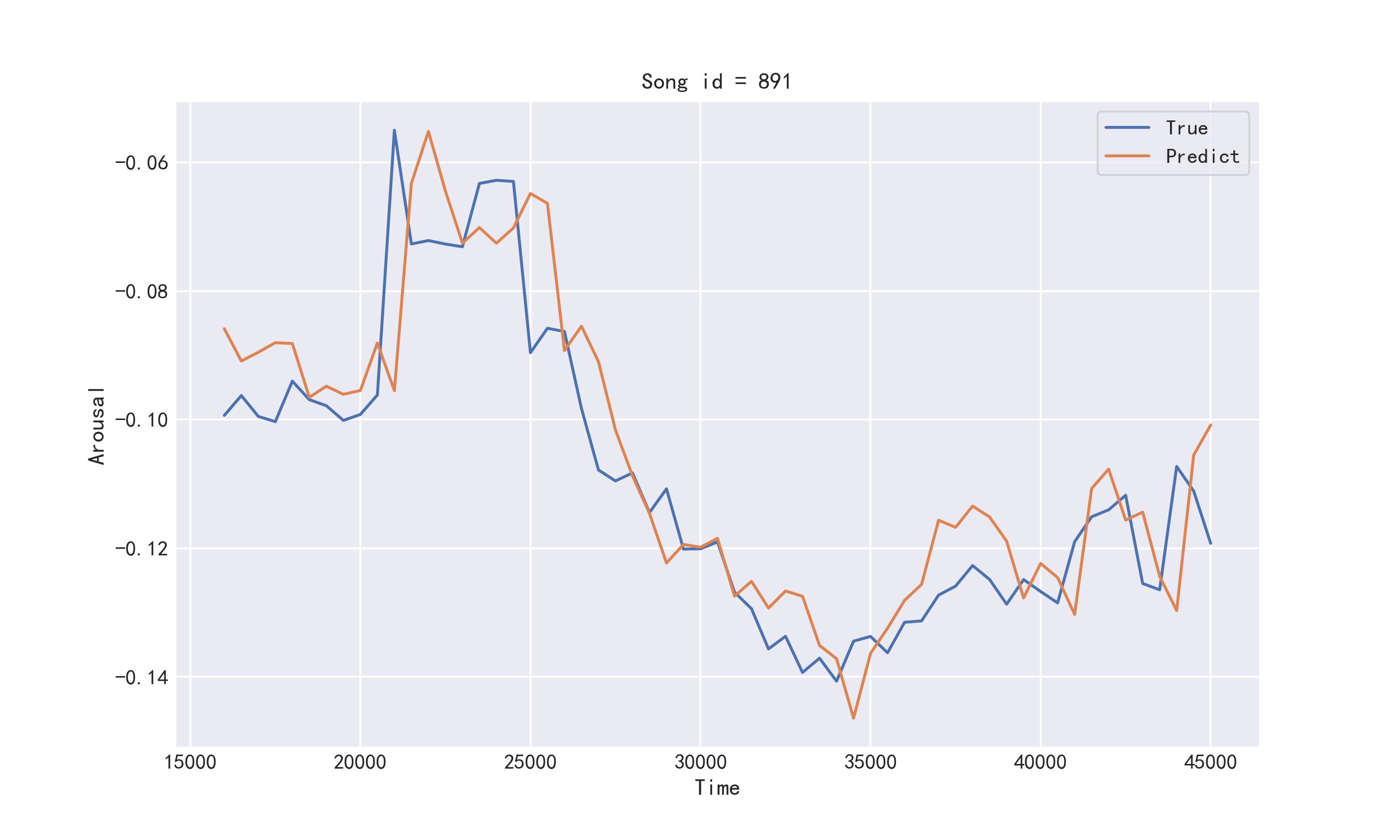

Supplement: S5 File — (ZIP) [file pone.0297712.s005.zip › All prediction results/prediction picture results(Emomusic_75)/song_id_891.jpg]

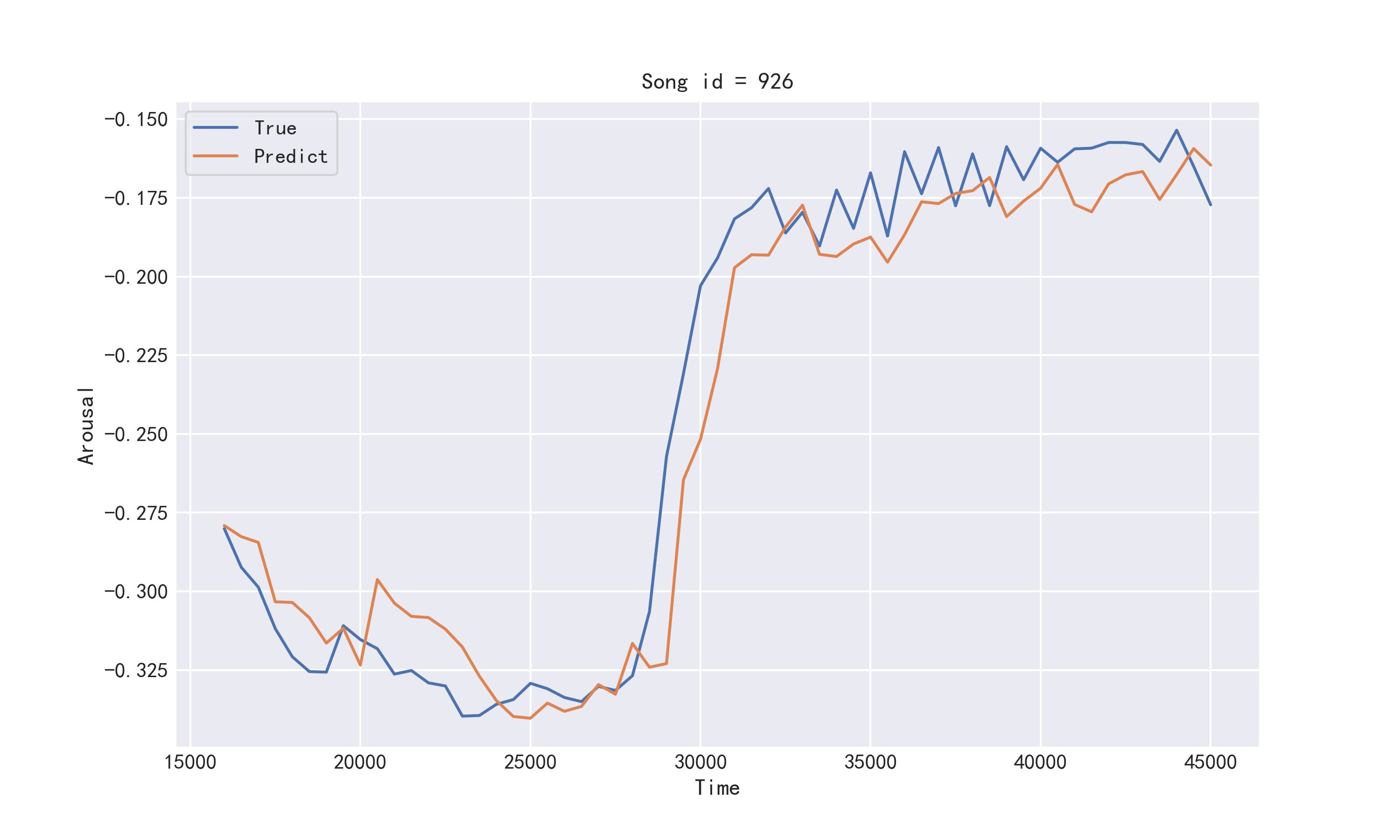

Supplement: S5 File — (ZIP) [file pone.0297712.s005.zip › All prediction results/prediction picture results(Emomusic_75)/song_id_926.jpg]

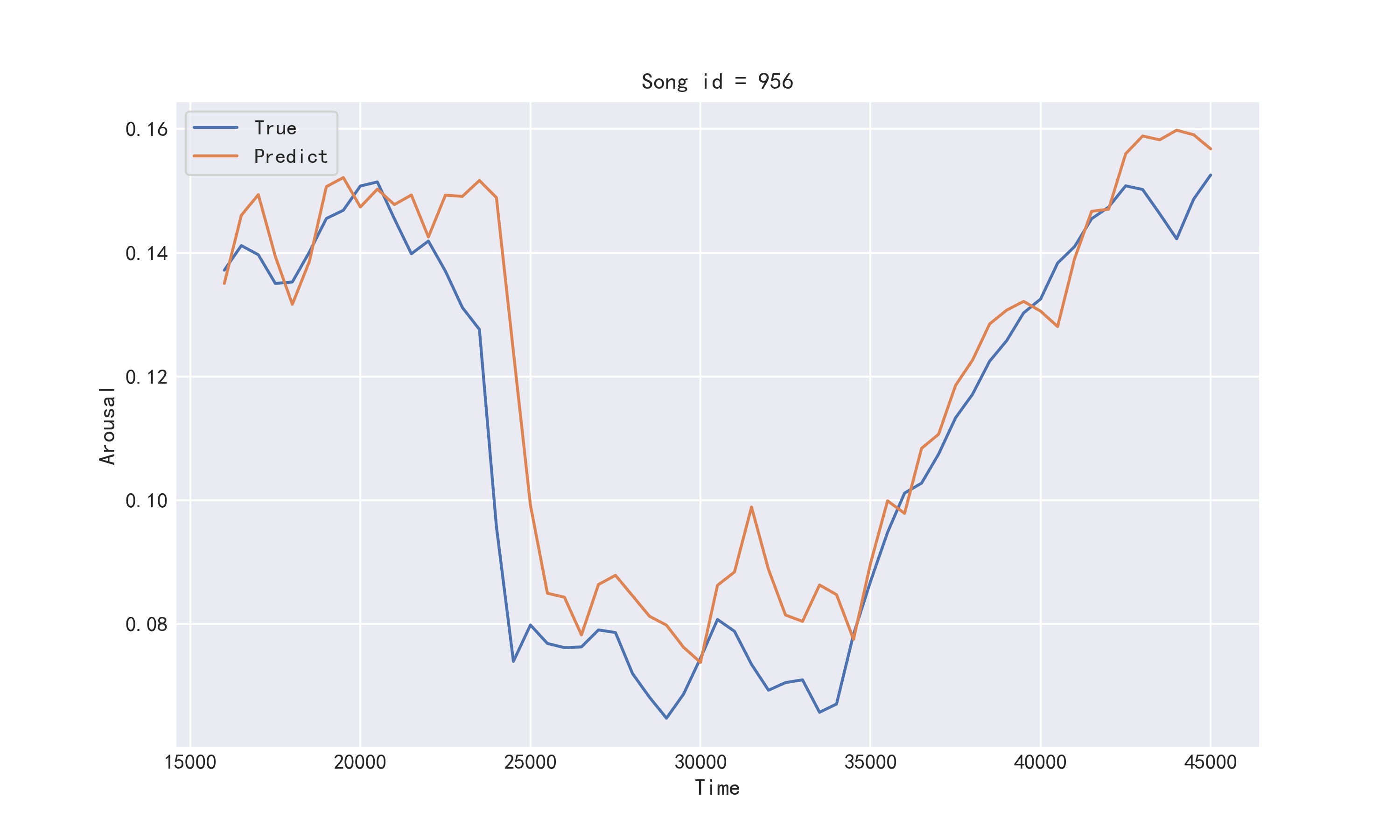

Supplement: S5 File — (ZIP) [file pone.0297712.s005.zip › All prediction results/prediction picture results(Emomusic_75)/song_id_956.jpg]

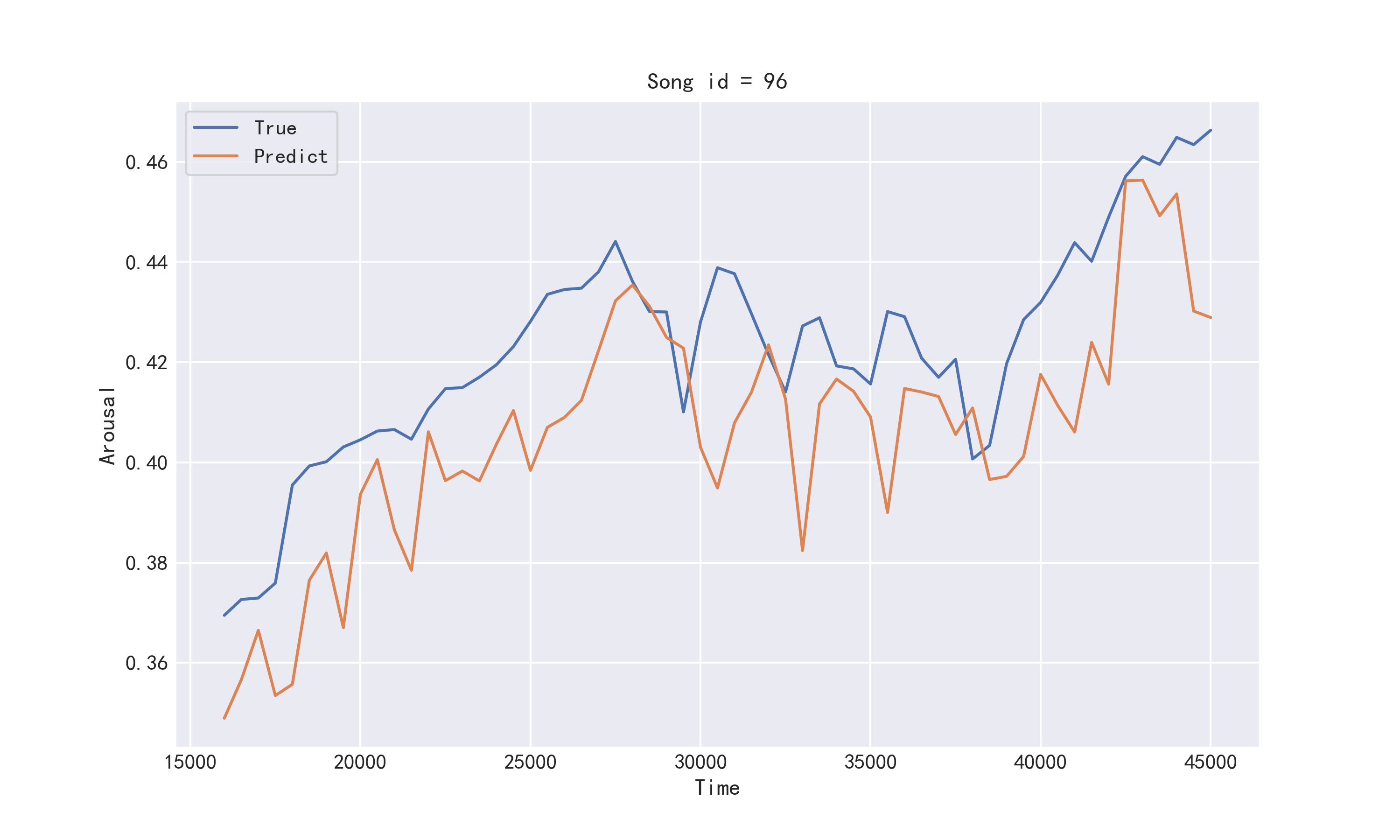

Supplement: S5 File — (ZIP) [file pone.0297712.s005.zip › All prediction results/prediction picture results(Emomusic_75)/song_id_96.jpg]

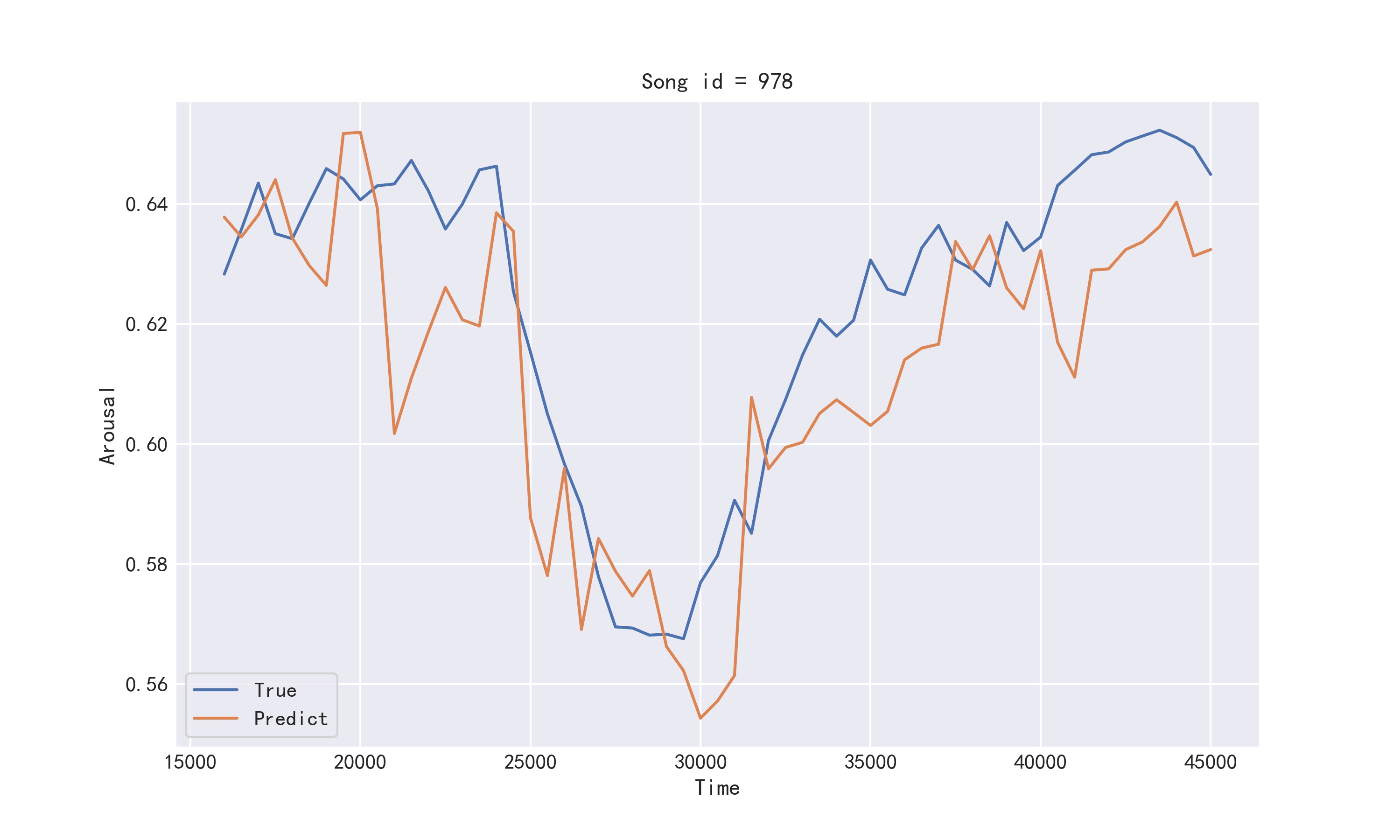

Supplement: S5 File — (ZIP) [file pone.0297712.s005.zip › All prediction results/prediction picture results(Emomusic_75)/song_id_978.jpg]

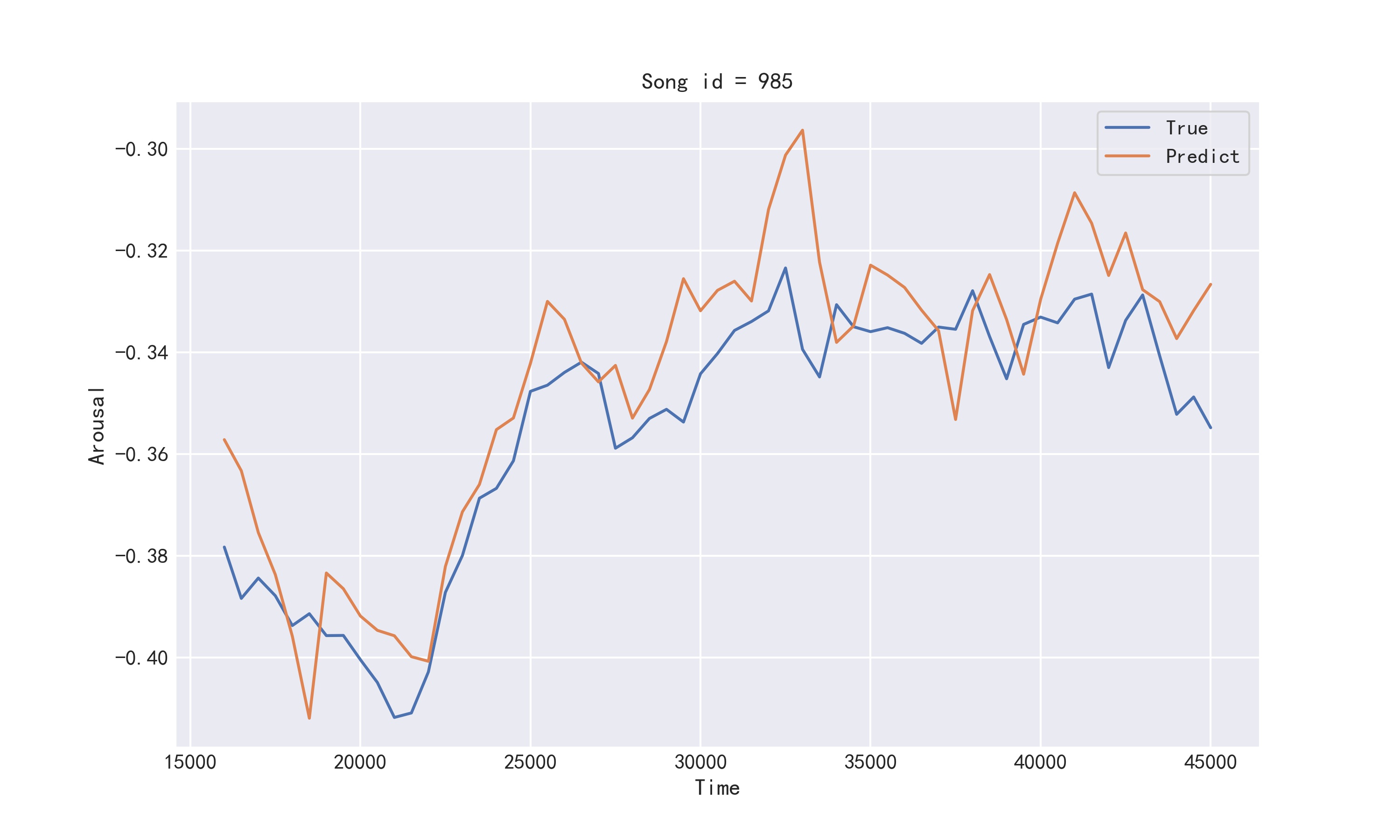

Supplement: S5 File — (ZIP) [file pone.0297712.s005.zip › All prediction results/prediction picture results(Emomusic_75)/song_id_985.jpg]

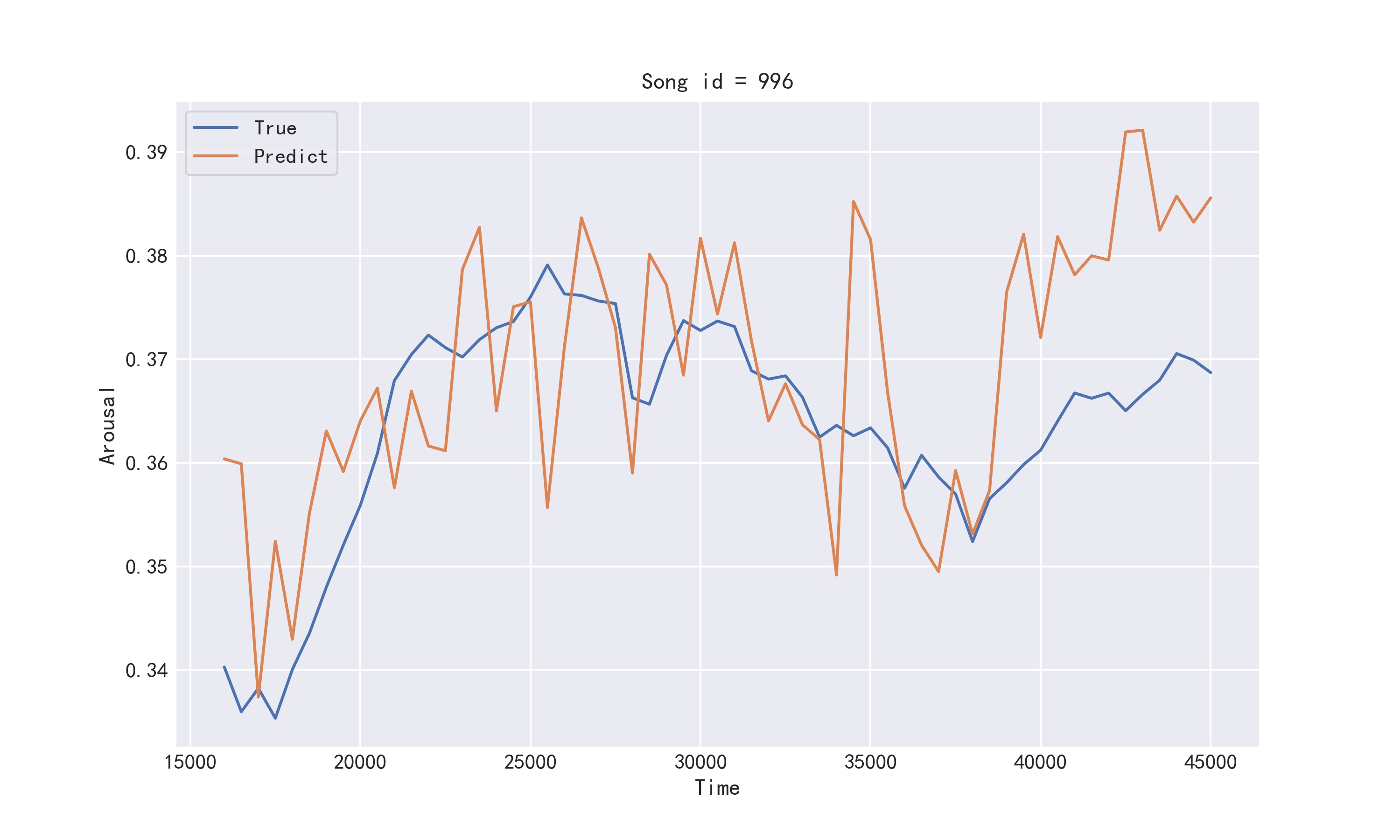

Supplement: S5 File — (ZIP) [file pone.0297712.s005.zip › All prediction results/prediction picture results(Emomusic_75)/song_id_996.jpg]
