# Supplementary figures and images for: Music-induced emotion flow modeling by ENMI Network (part 1 of 2)
Source: PLoS One. 2024 Oct 21;19(10):e0297712. doi: 10.1371/journal.pone.0297712 (PMC11493256; doi:10.1371/journal.pone.0297712)

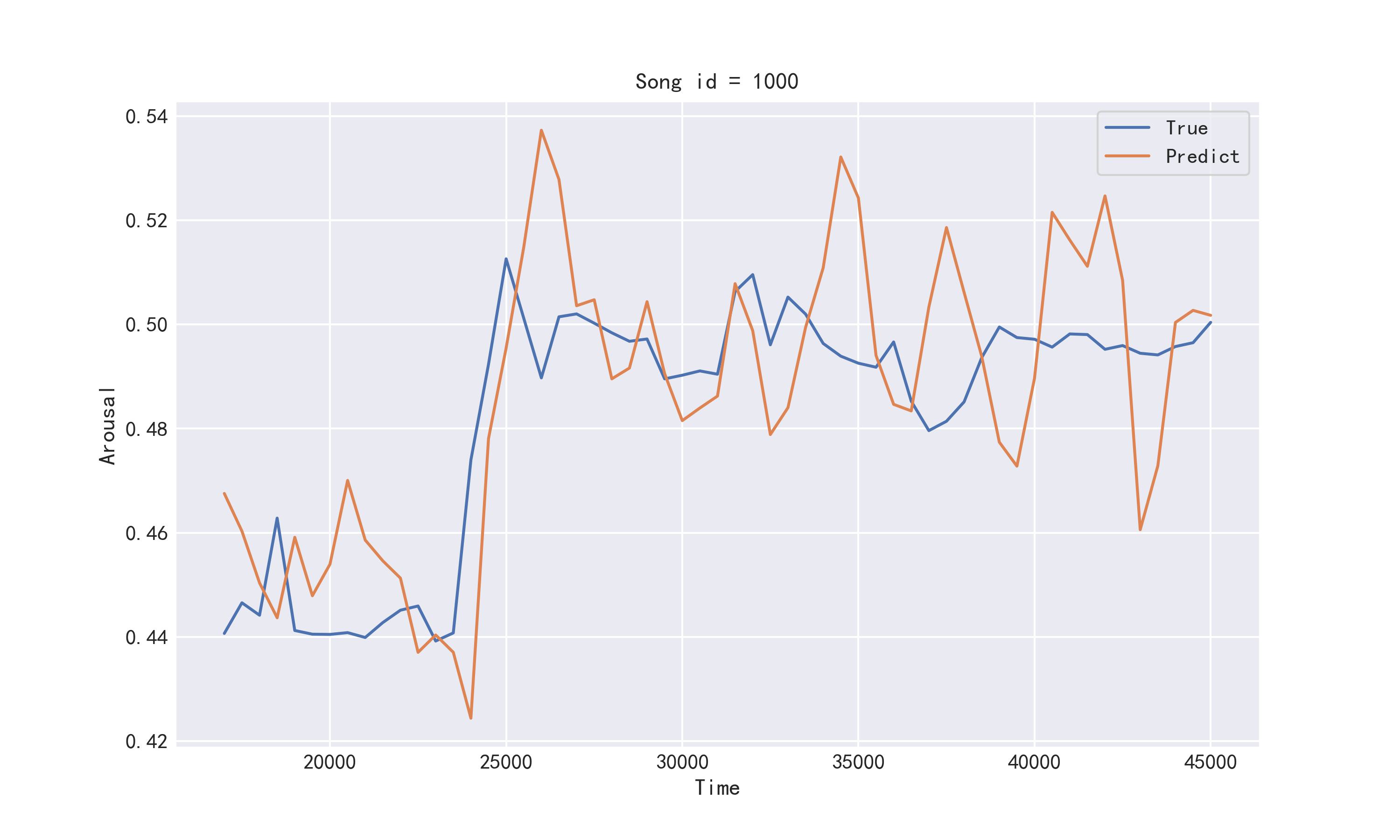

Supplement: S5 File — (ZIP) [file pone.0297712.s005.zip › All prediction results/prediction picture results(DEAM_100)/song_id_1000.jpg]

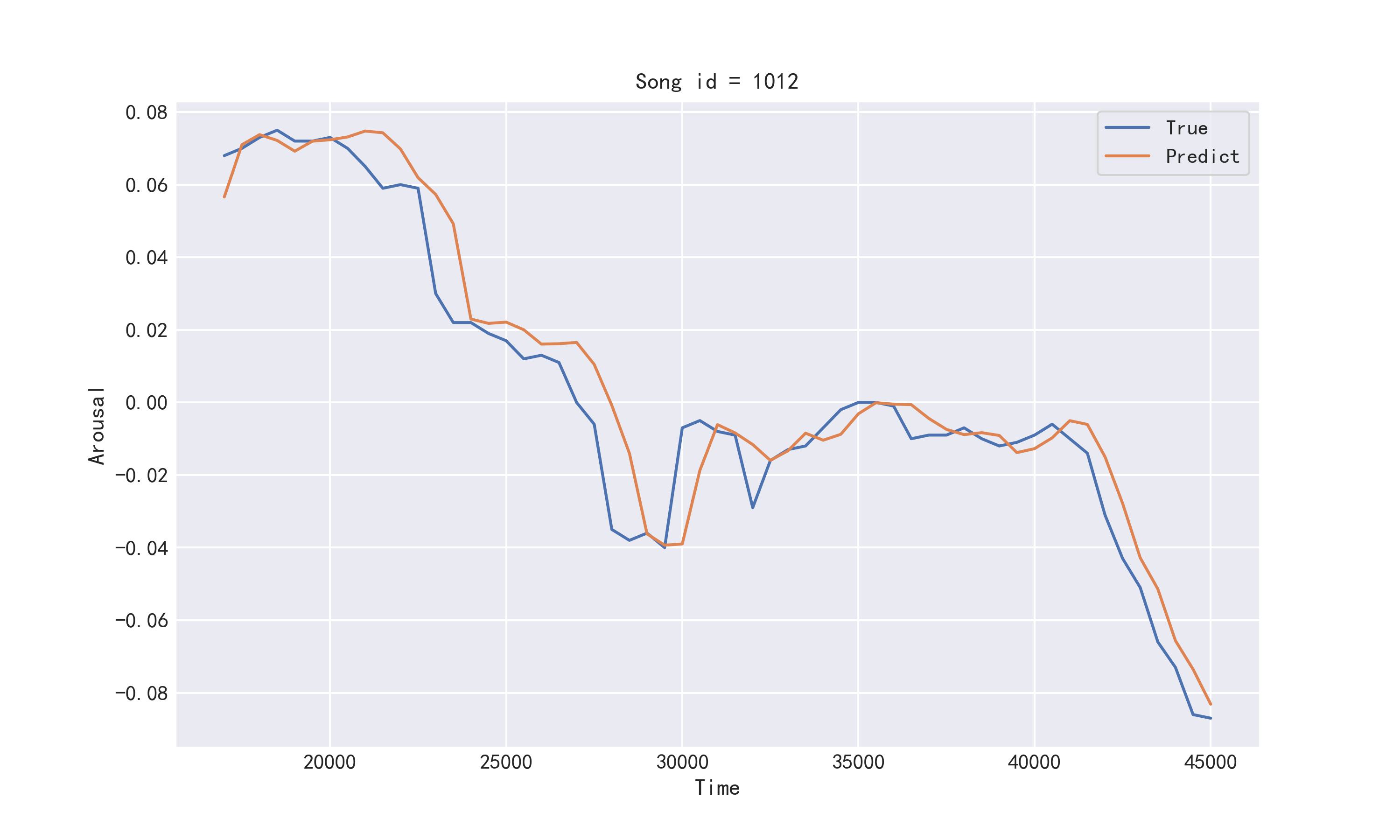

Supplement: S5 File — (ZIP) [file pone.0297712.s005.zip › All prediction results/prediction picture results(DEAM_100)/song_id_1012.jpg]

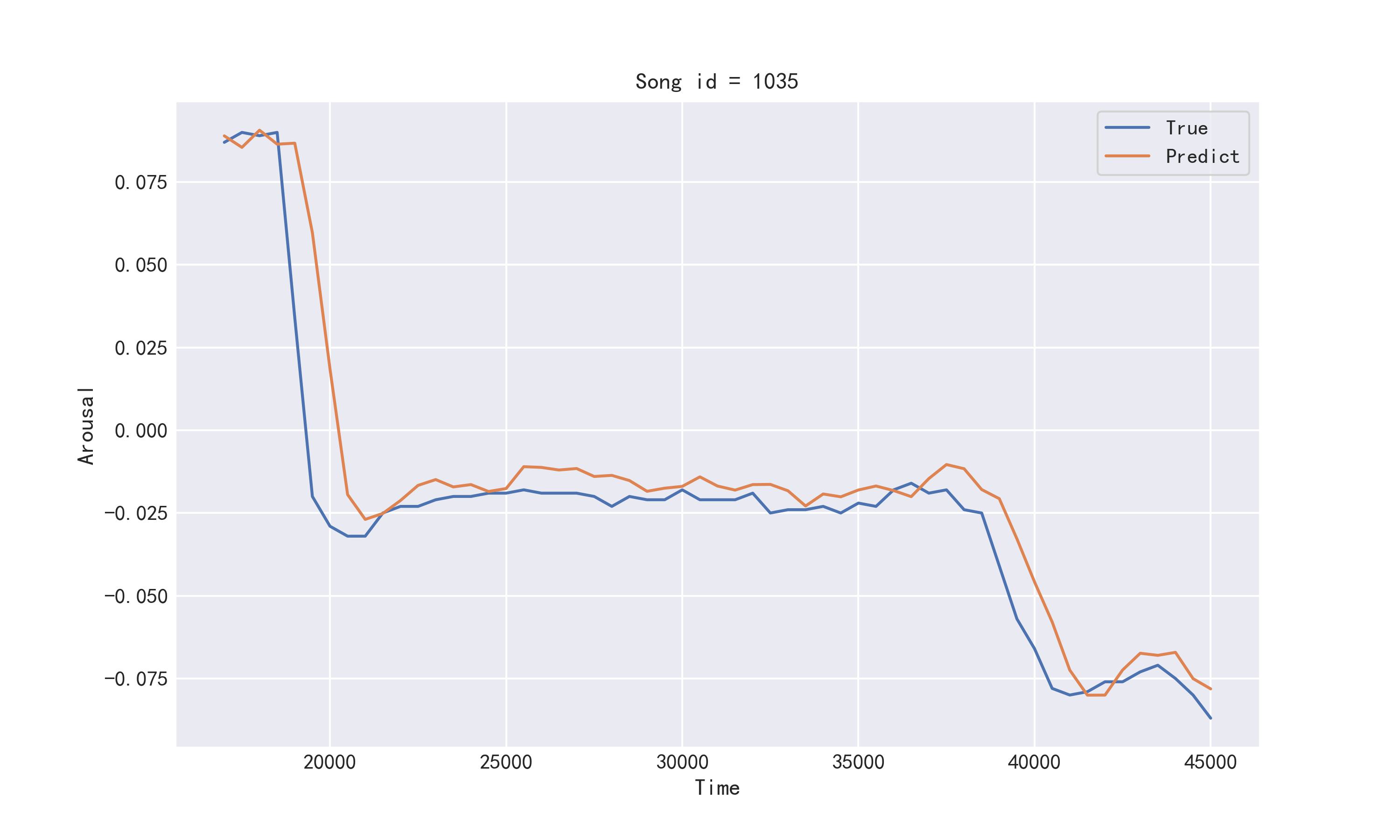

Supplement: S5 File — (ZIP) [file pone.0297712.s005.zip › All prediction results/prediction picture results(DEAM_100)/song_id_1035.jpg]

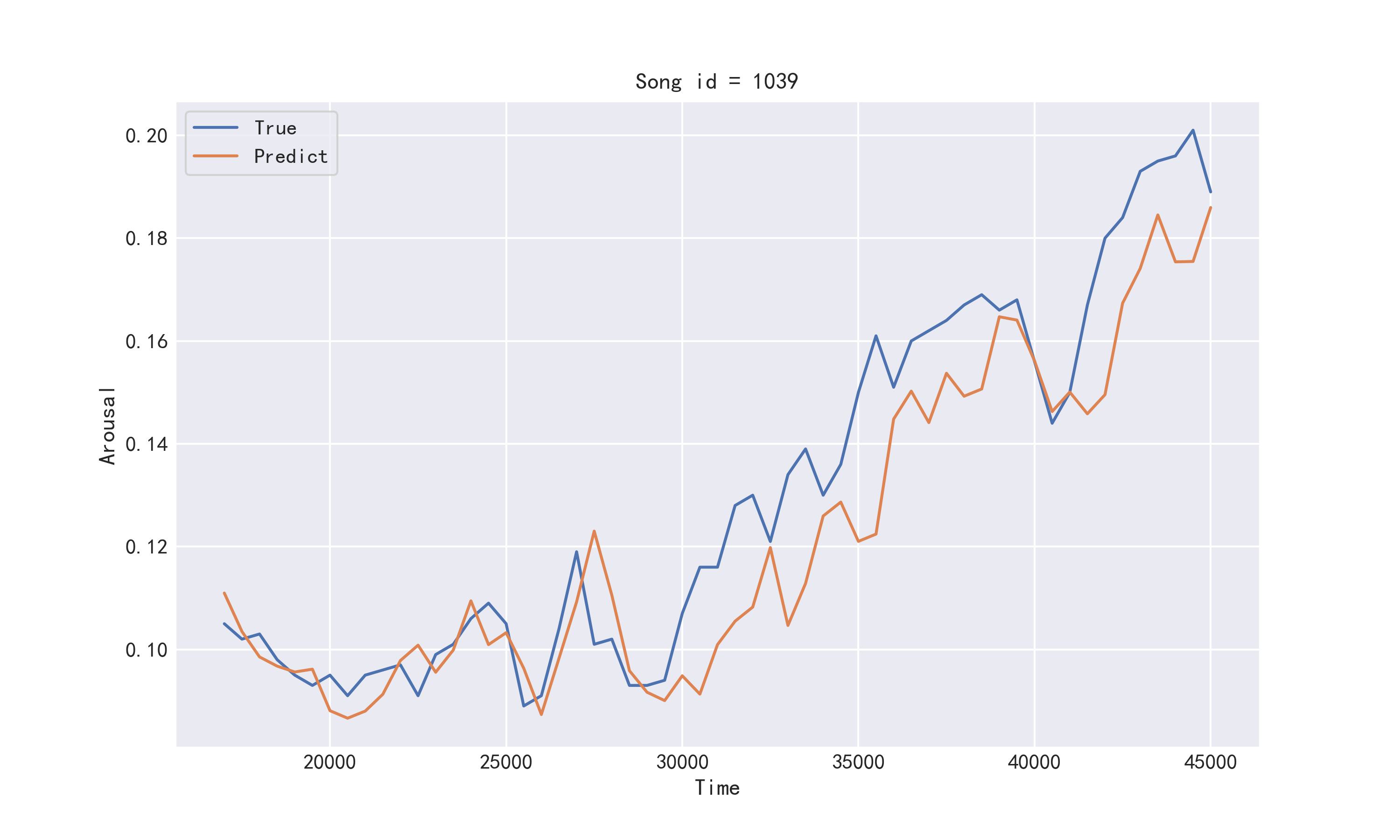

Supplement: S5 File — (ZIP) [file pone.0297712.s005.zip › All prediction results/prediction picture results(DEAM_100)/song_id_1039.jpg]

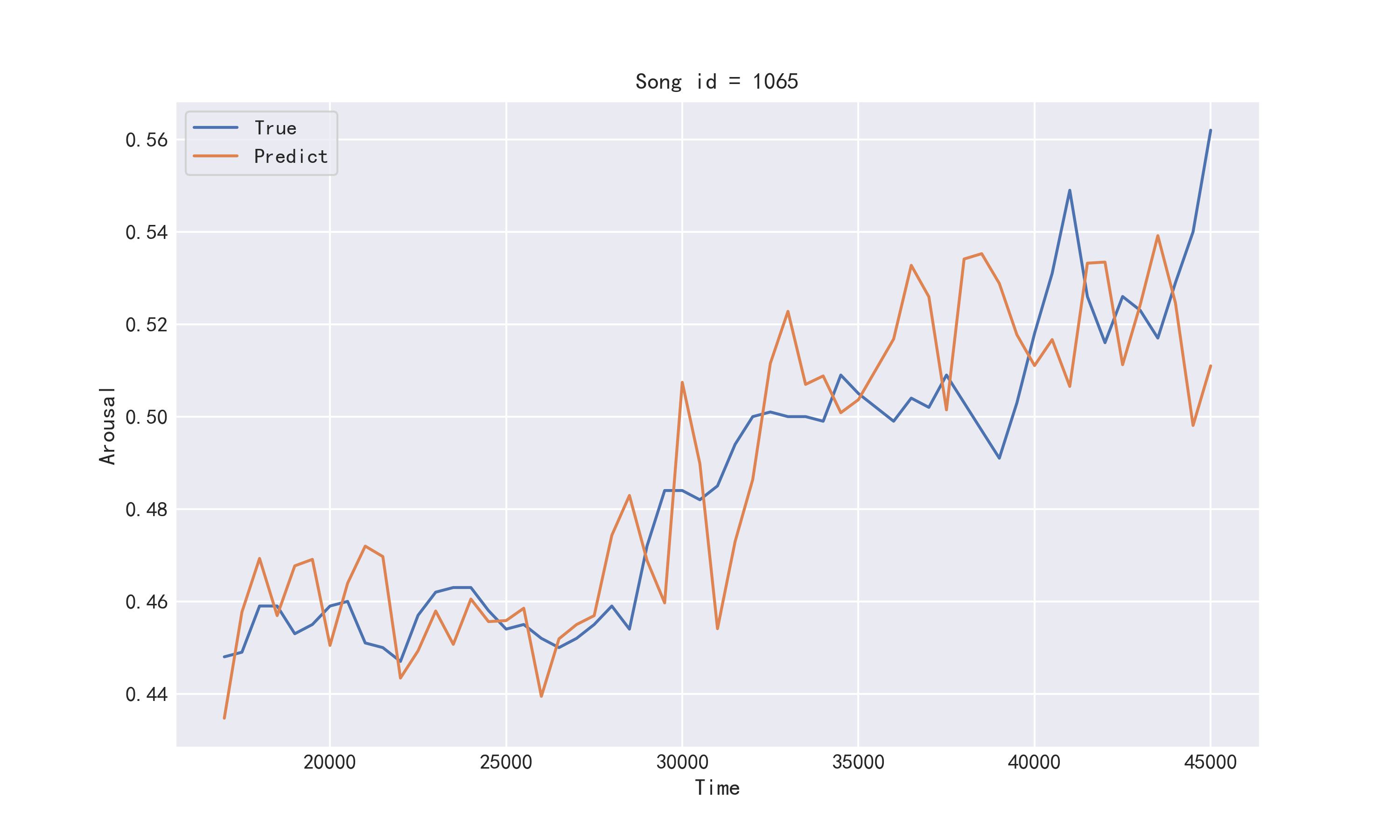

Supplement: S5 File — (ZIP) [file pone.0297712.s005.zip › All prediction results/prediction picture results(DEAM_100)/song_id_1065.jpg]

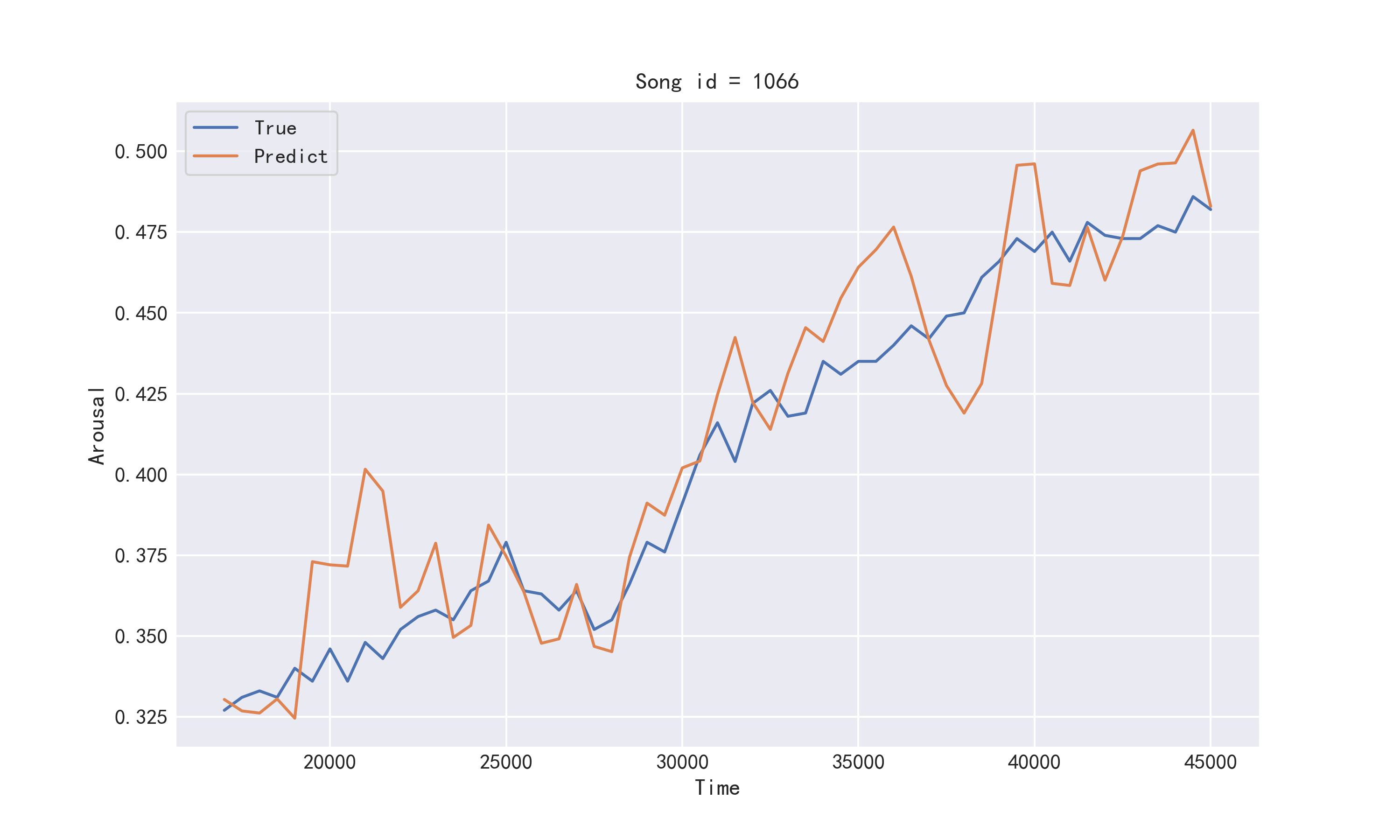

Supplement: S5 File — (ZIP) [file pone.0297712.s005.zip › All prediction results/prediction picture results(DEAM_100)/song_id_1066.jpg]

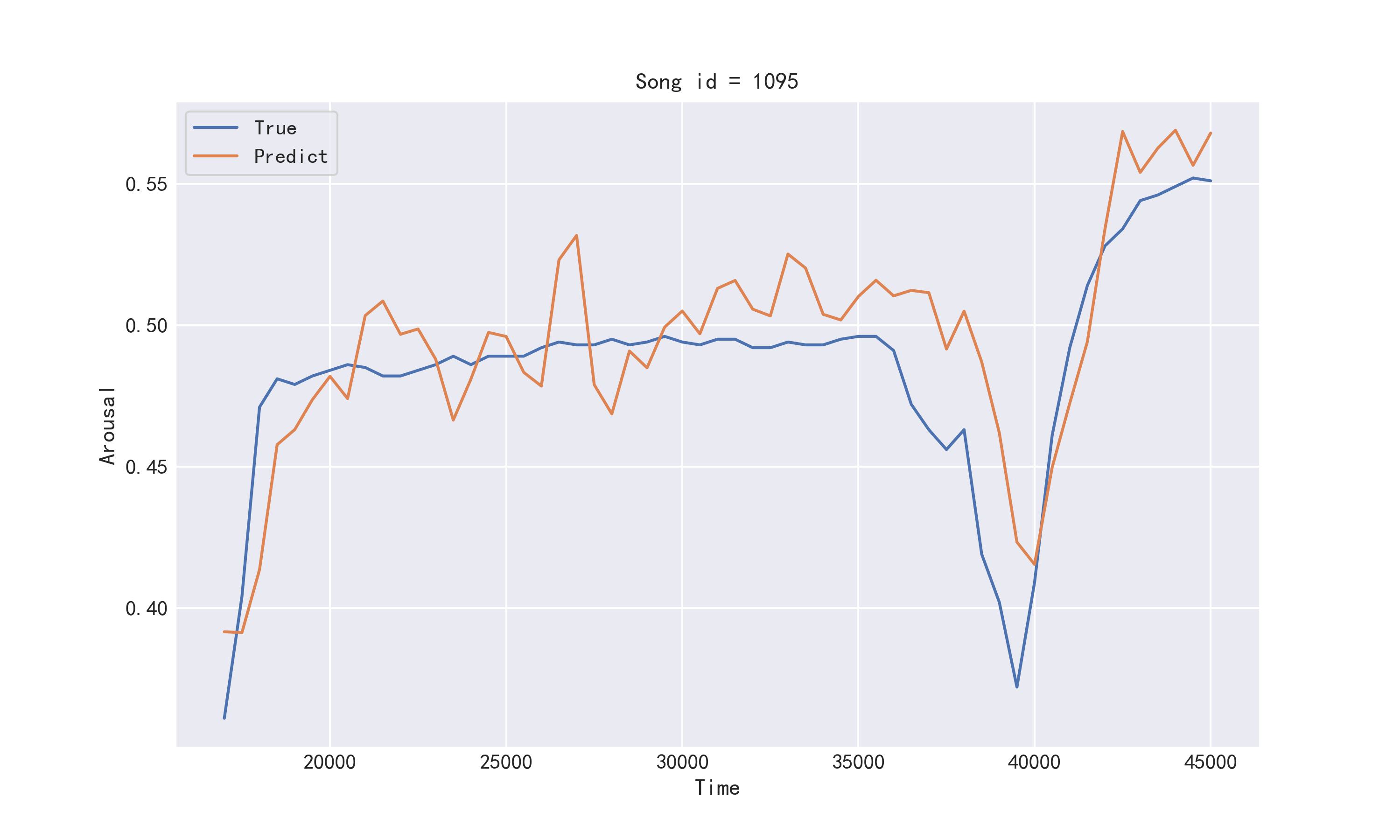

Supplement: S5 File — (ZIP) [file pone.0297712.s005.zip › All prediction results/prediction picture results(DEAM_100)/song_id_1095.jpg]

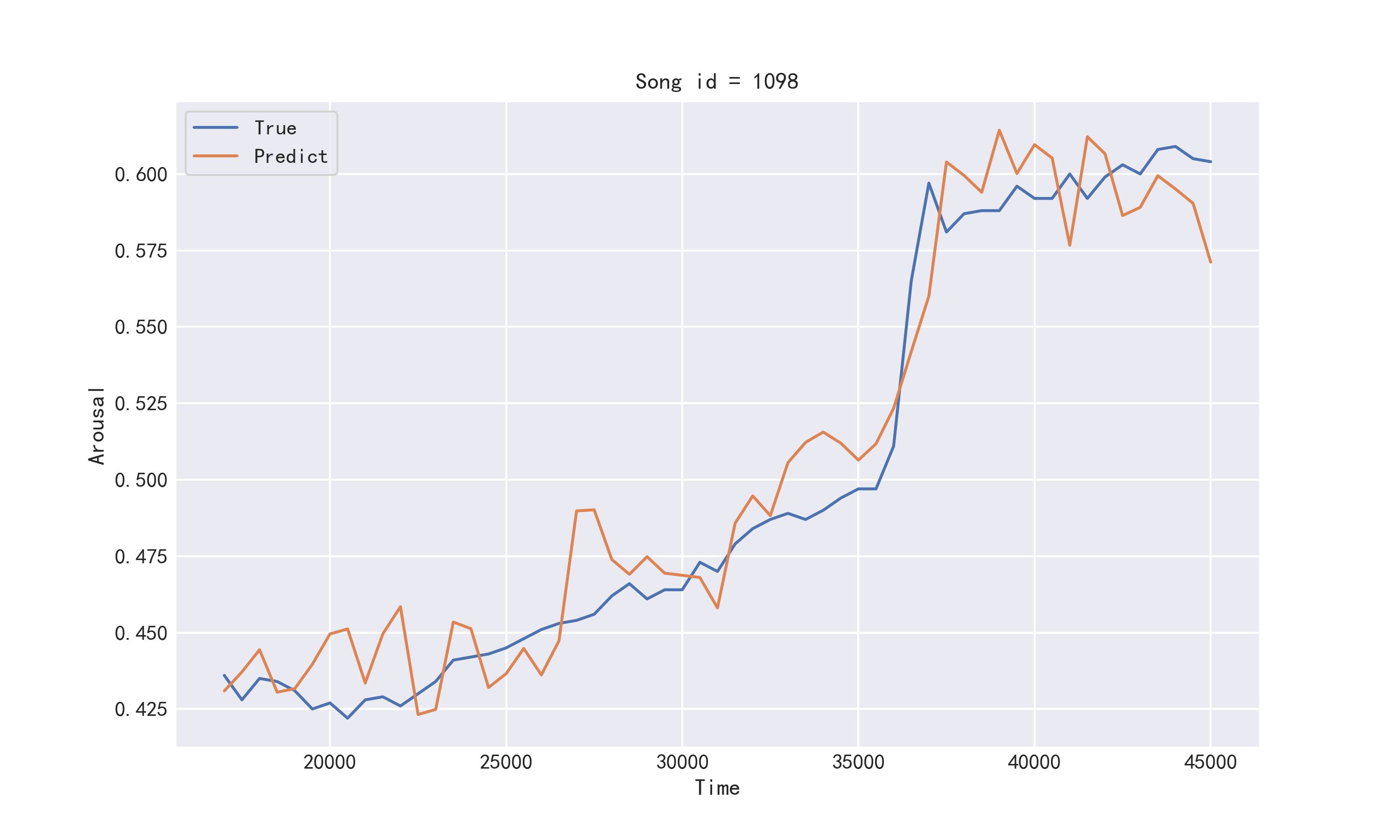

Supplement: S5 File — (ZIP) [file pone.0297712.s005.zip › All prediction results/prediction picture results(DEAM_100)/song_id_1098.jpg]

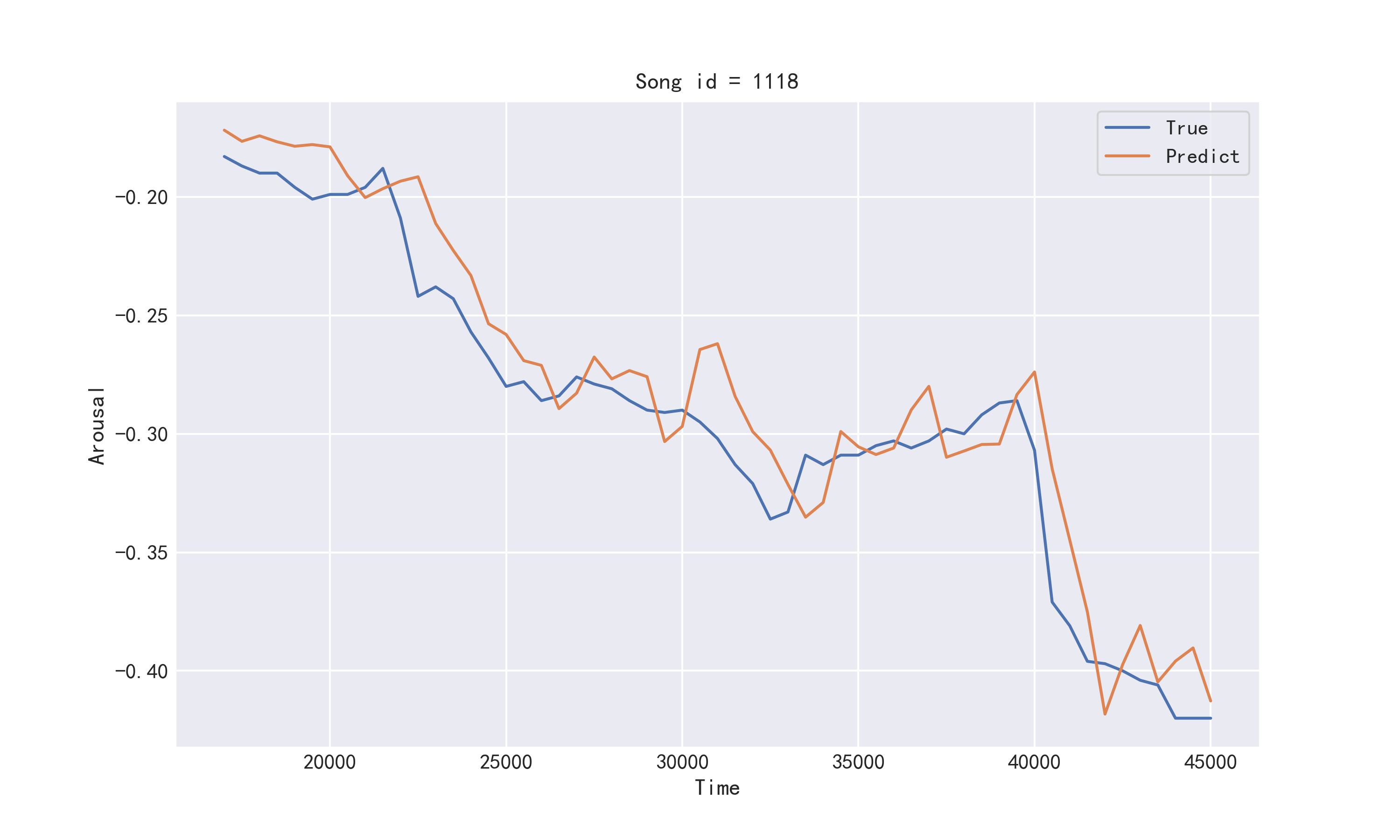

Supplement: S5 File — (ZIP) [file pone.0297712.s005.zip › All prediction results/prediction picture results(DEAM_100)/song_id_1118.jpg]

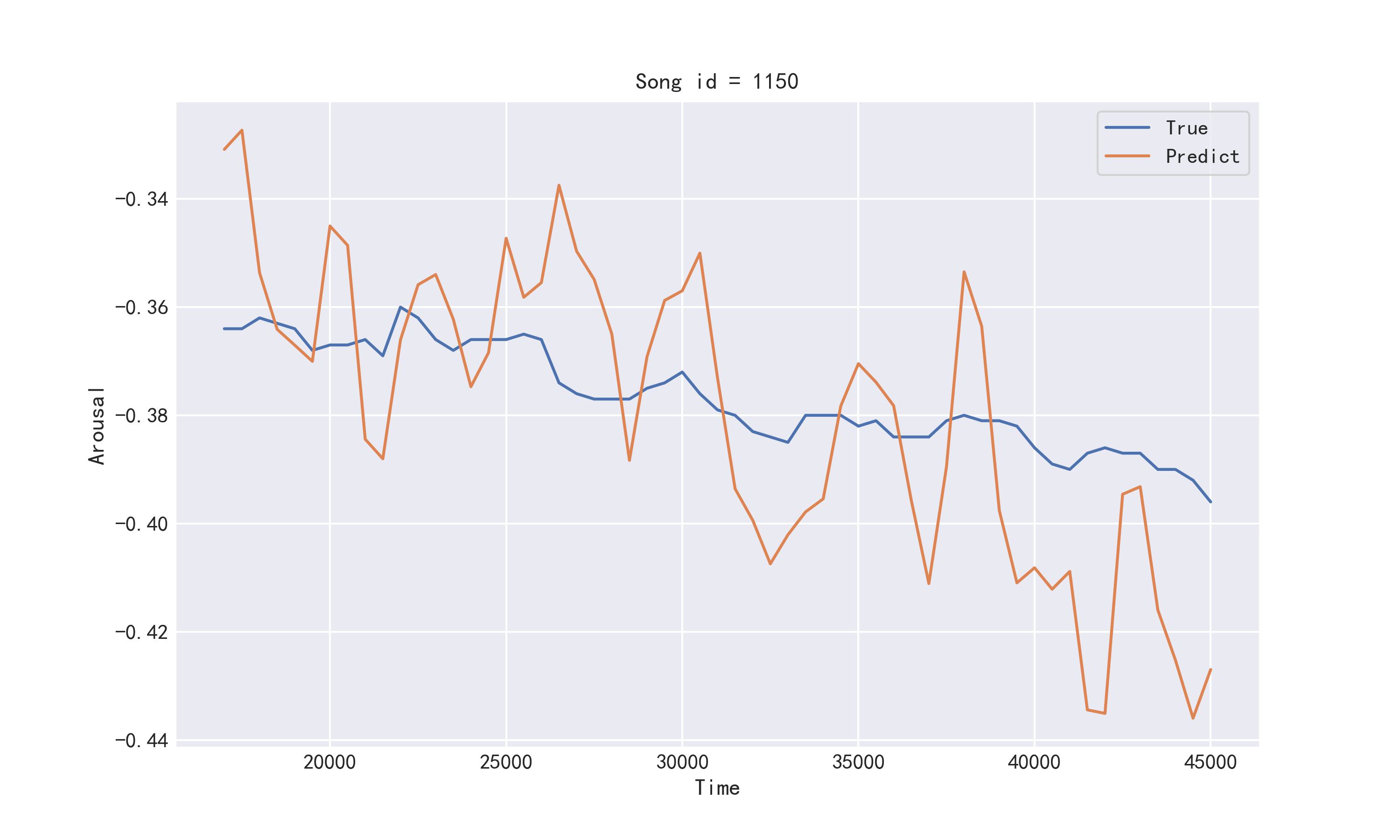

Supplement: S5 File — (ZIP) [file pone.0297712.s005.zip › All prediction results/prediction picture results(DEAM_100)/song_id_1150.jpg]

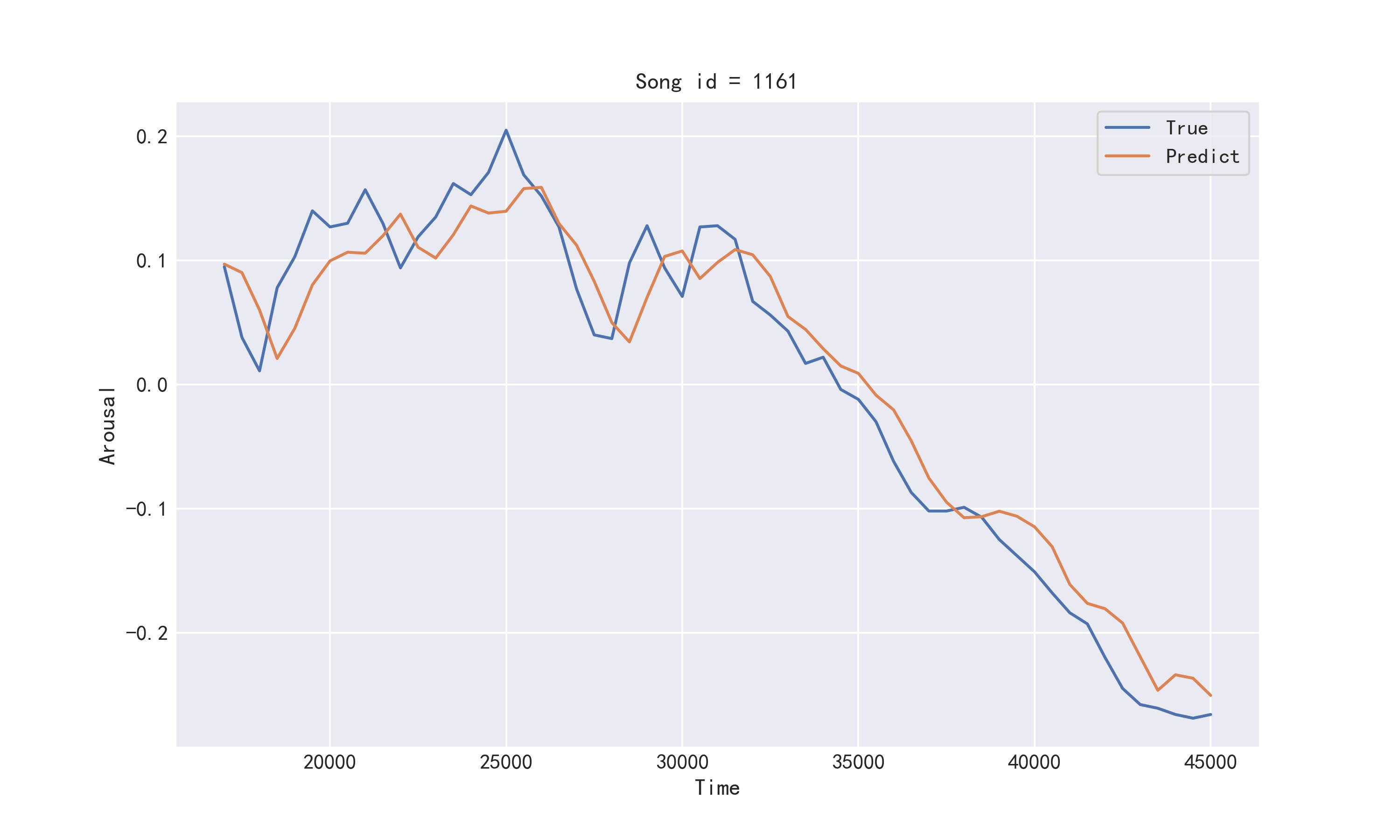

Supplement: S5 File — (ZIP) [file pone.0297712.s005.zip › All prediction results/prediction picture results(DEAM_100)/song_id_1161.jpg]

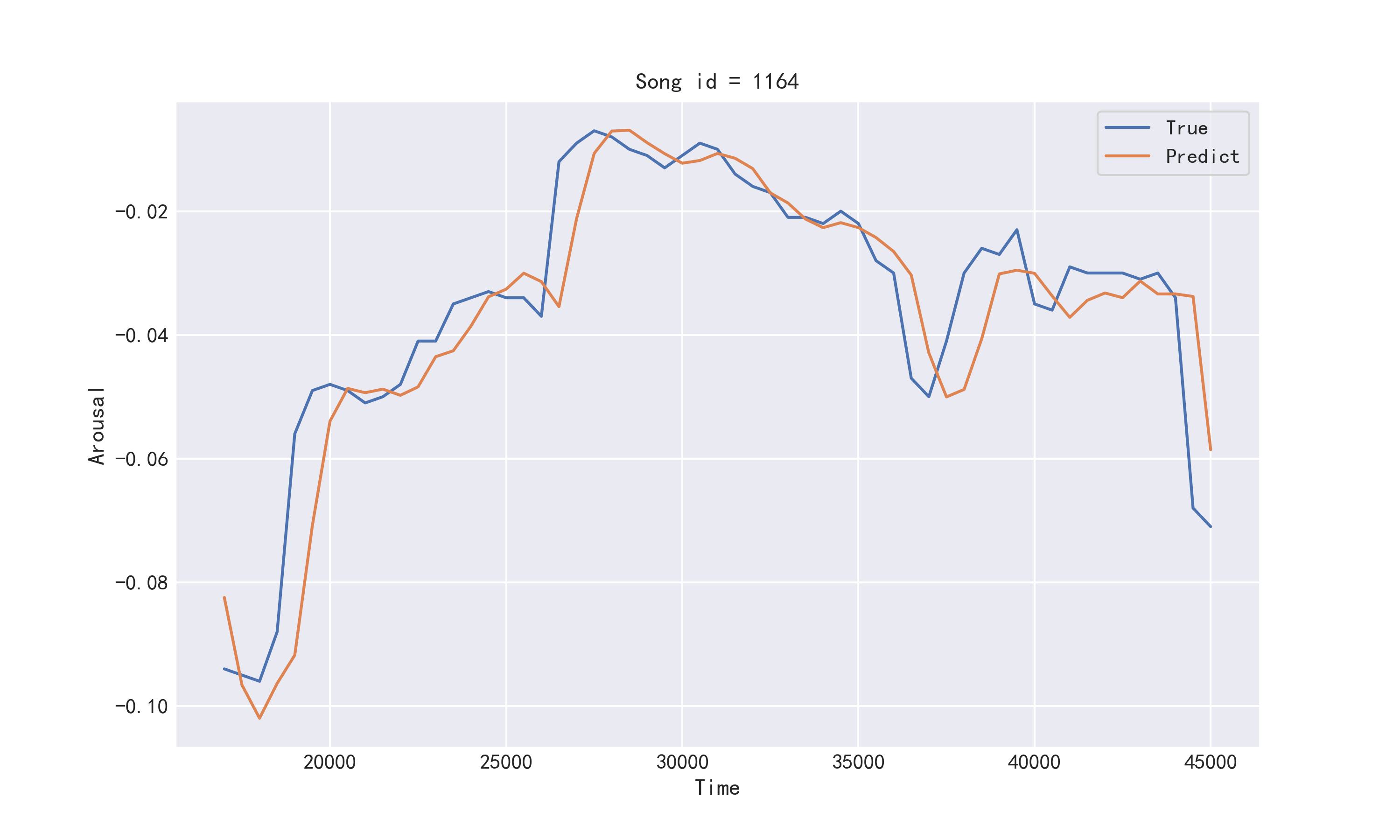

Supplement: S5 File — (ZIP) [file pone.0297712.s005.zip › All prediction results/prediction picture results(DEAM_100)/song_id_1164.jpg]

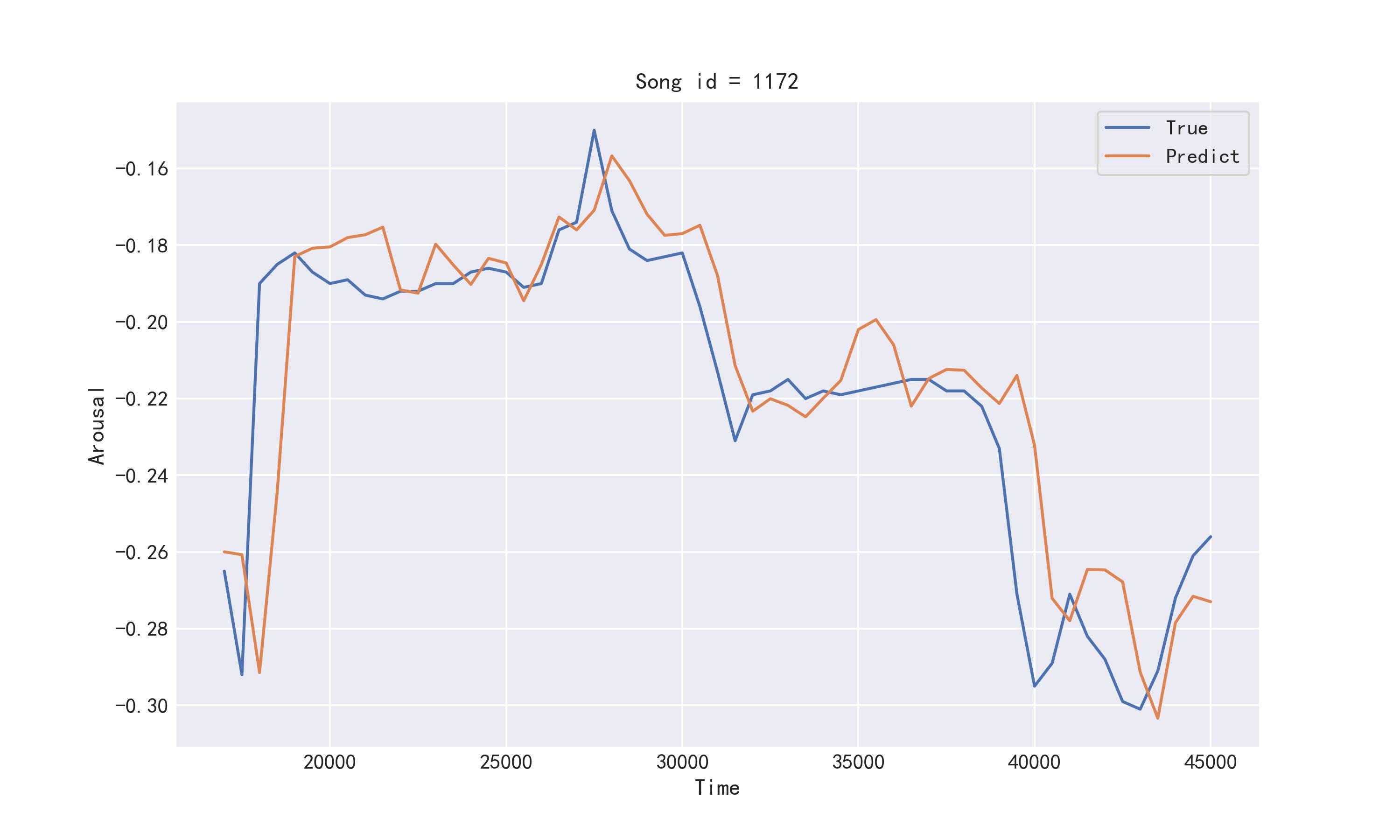

Supplement: S5 File — (ZIP) [file pone.0297712.s005.zip › All prediction results/prediction picture results(DEAM_100)/song_id_1172.jpg]

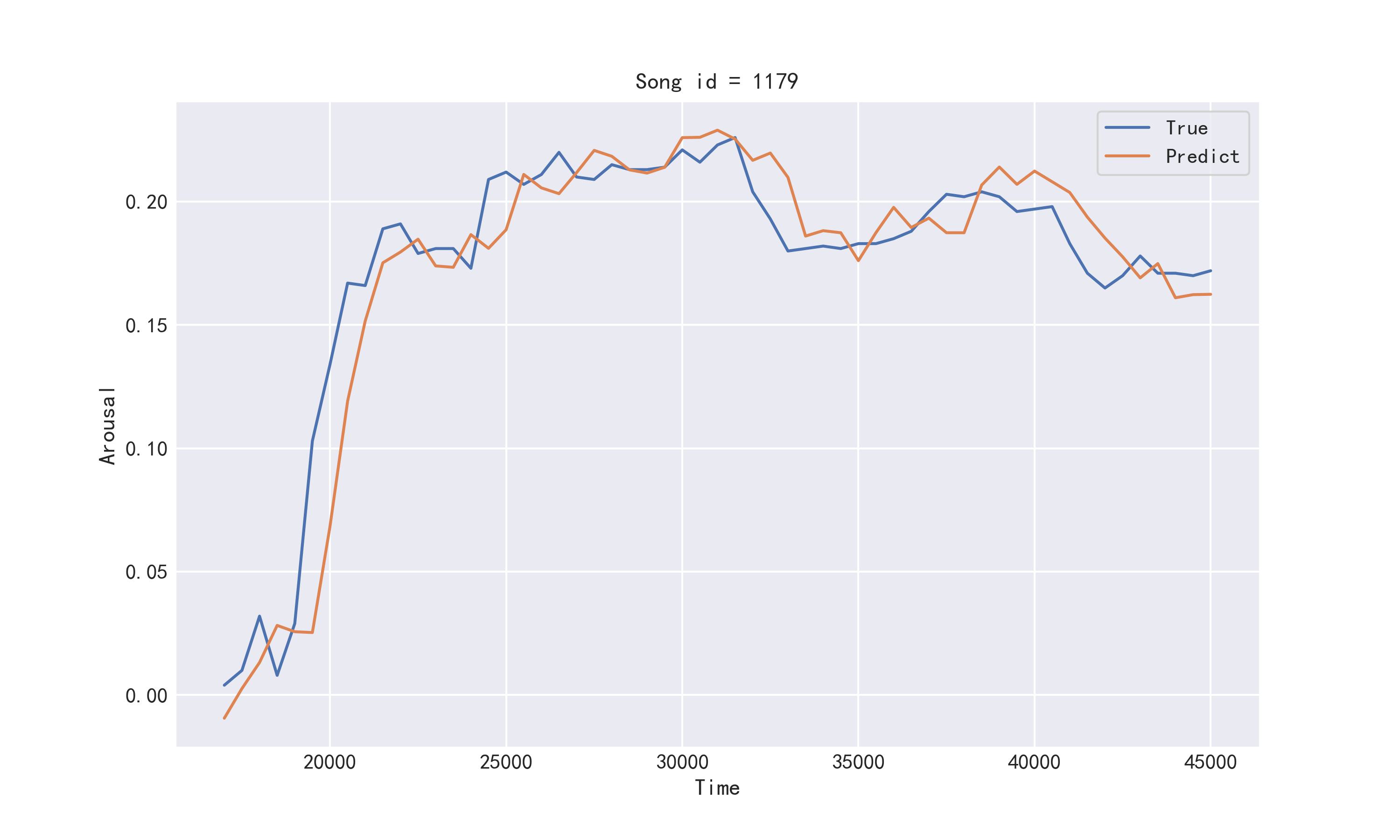

Supplement: S5 File — (ZIP) [file pone.0297712.s005.zip › All prediction results/prediction picture results(DEAM_100)/song_id_1179.jpg]

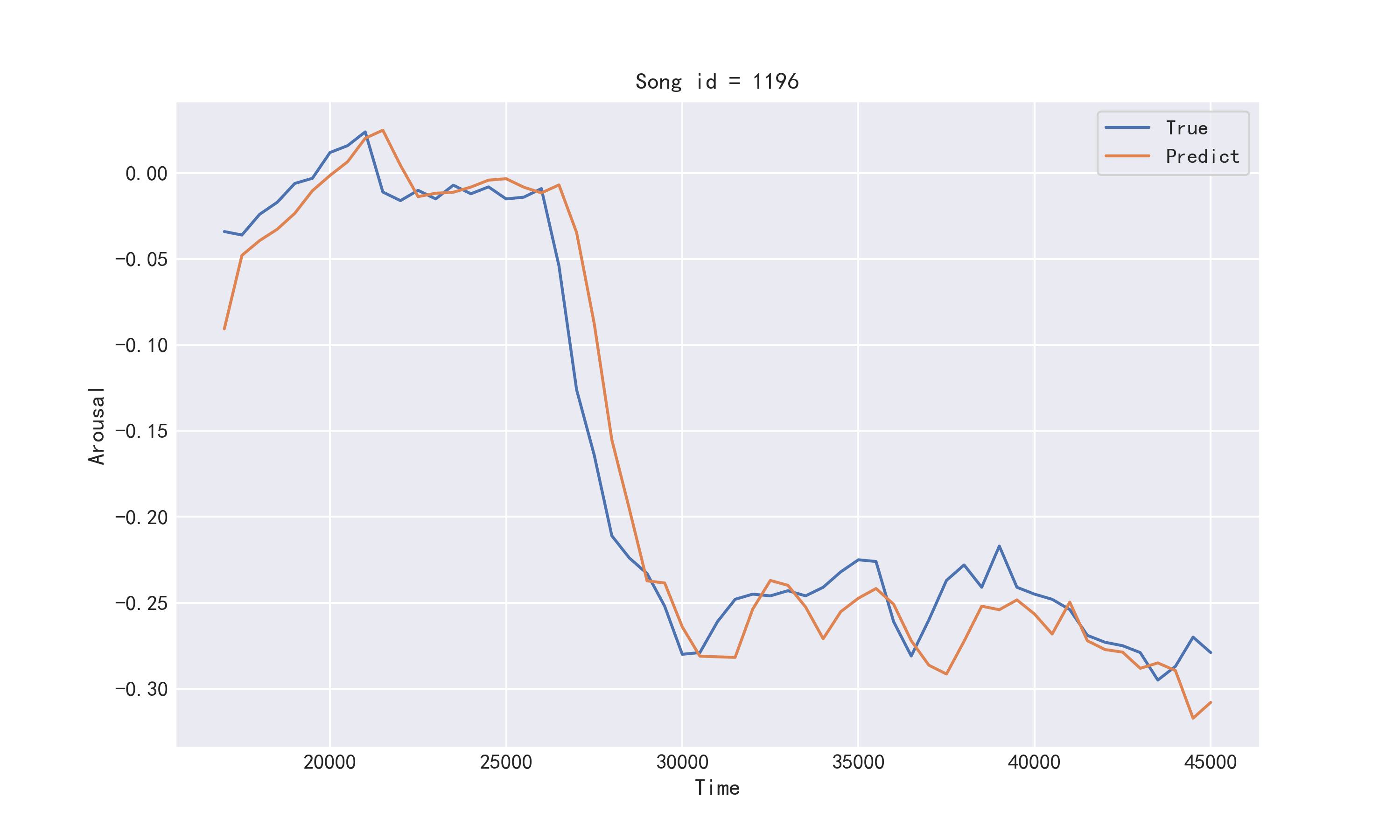

Supplement: S5 File — (ZIP) [file pone.0297712.s005.zip › All prediction results/prediction picture results(DEAM_100)/song_id_1196.jpg]

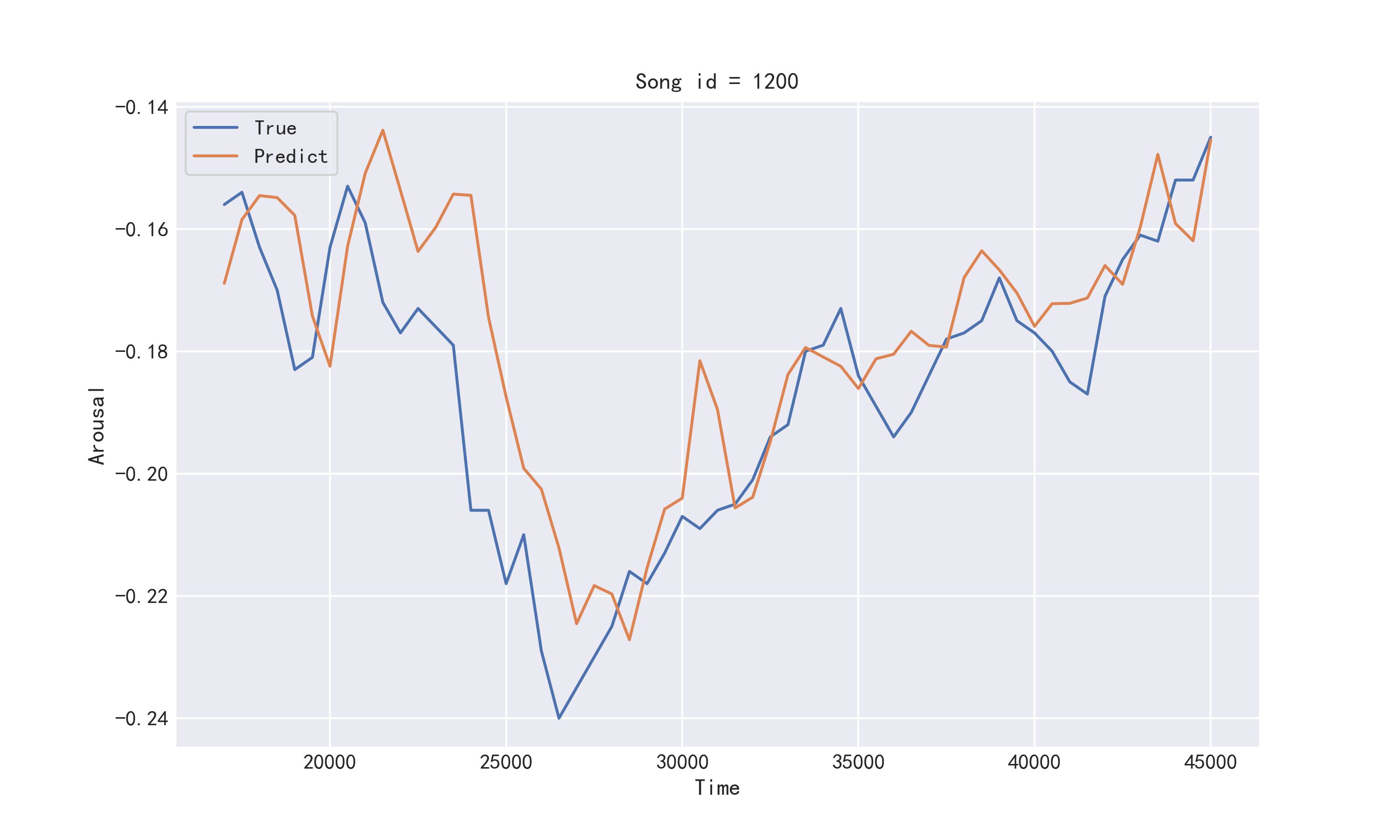

Supplement: S5 File — (ZIP) [file pone.0297712.s005.zip › All prediction results/prediction picture results(DEAM_100)/song_id_1200.jpg]

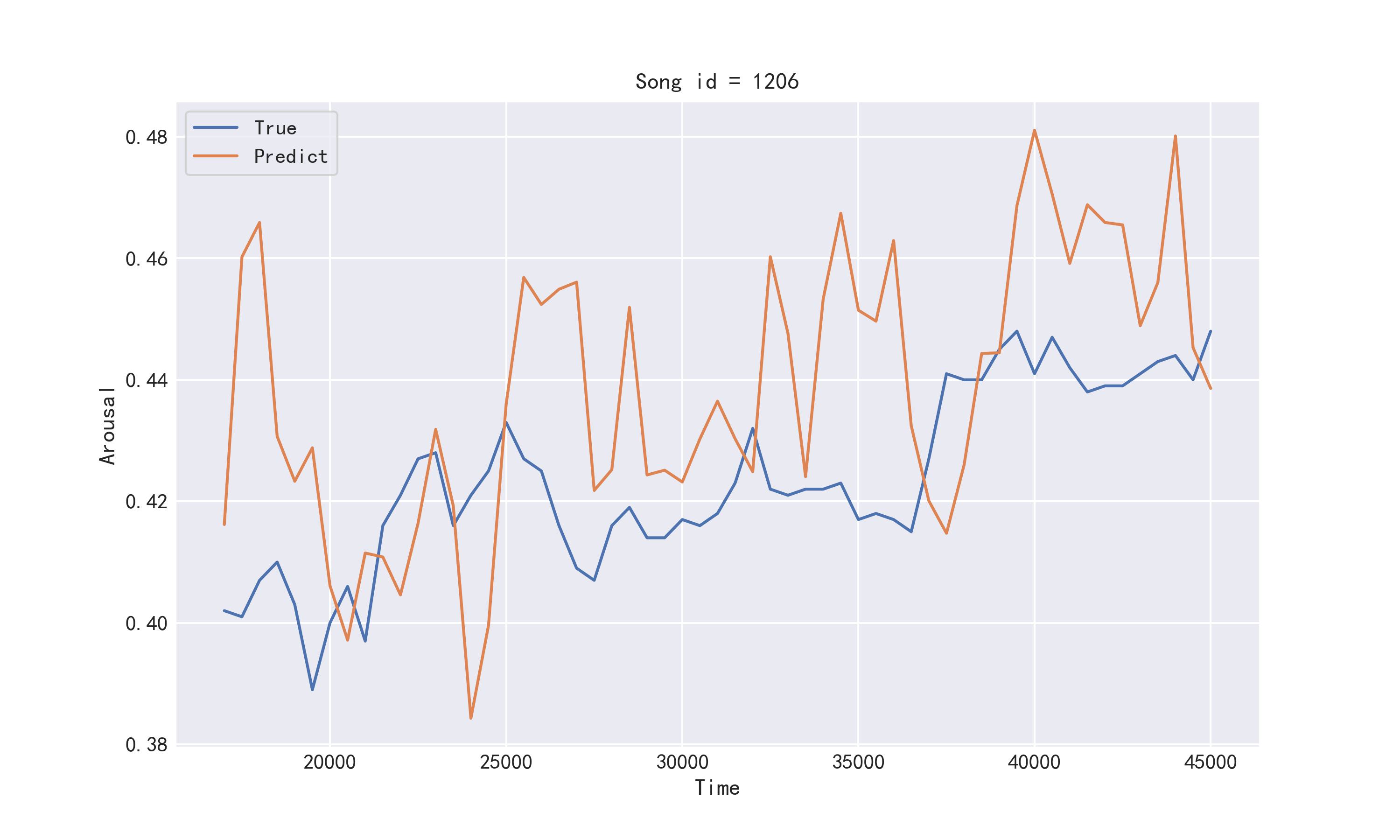

Supplement: S5 File — (ZIP) [file pone.0297712.s005.zip › All prediction results/prediction picture results(DEAM_100)/song_id_1206.jpg]

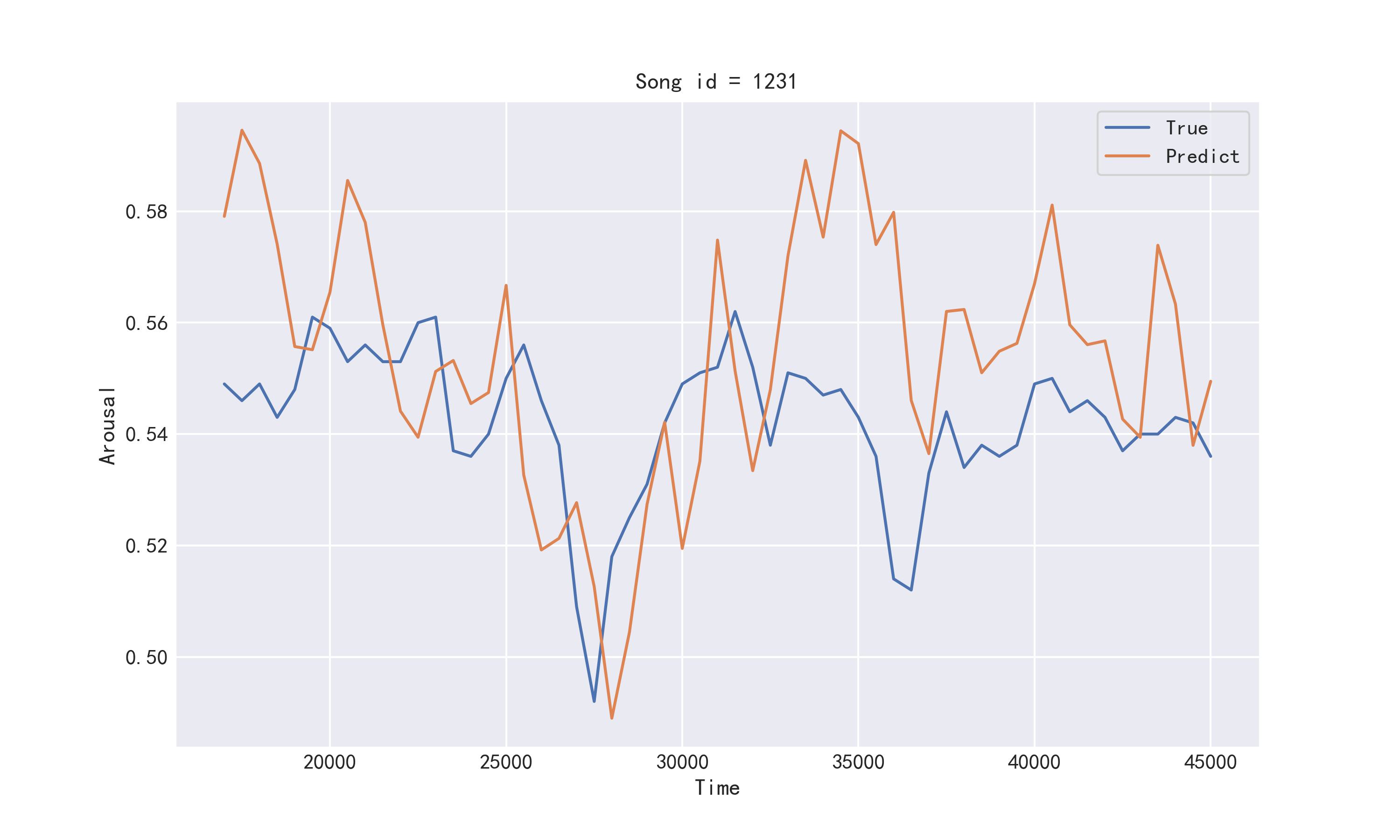

Supplement: S5 File — (ZIP) [file pone.0297712.s005.zip › All prediction results/prediction picture results(DEAM_100)/song_id_1231.jpg]

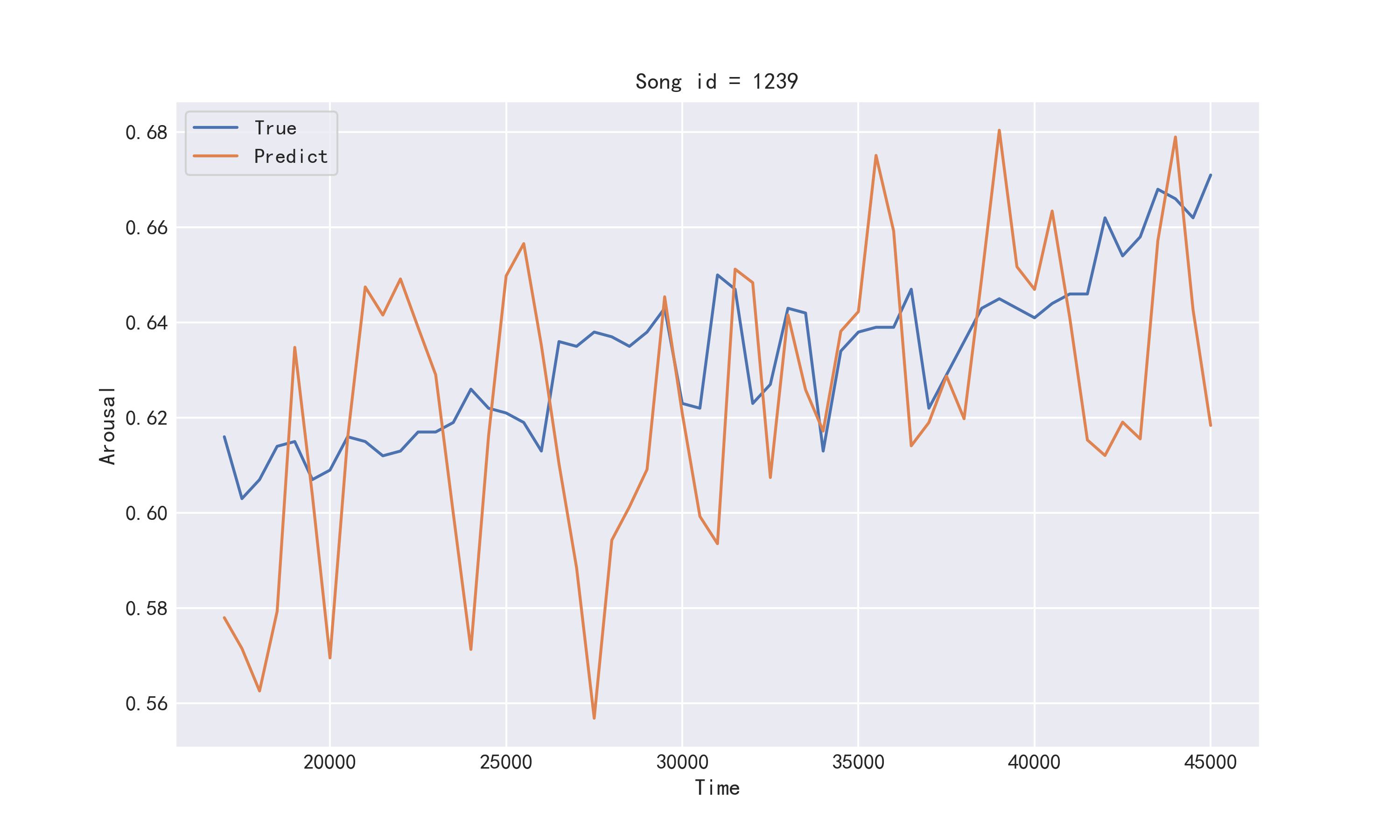

Supplement: S5 File — (ZIP) [file pone.0297712.s005.zip › All prediction results/prediction picture results(DEAM_100)/song_id_1239.jpg]

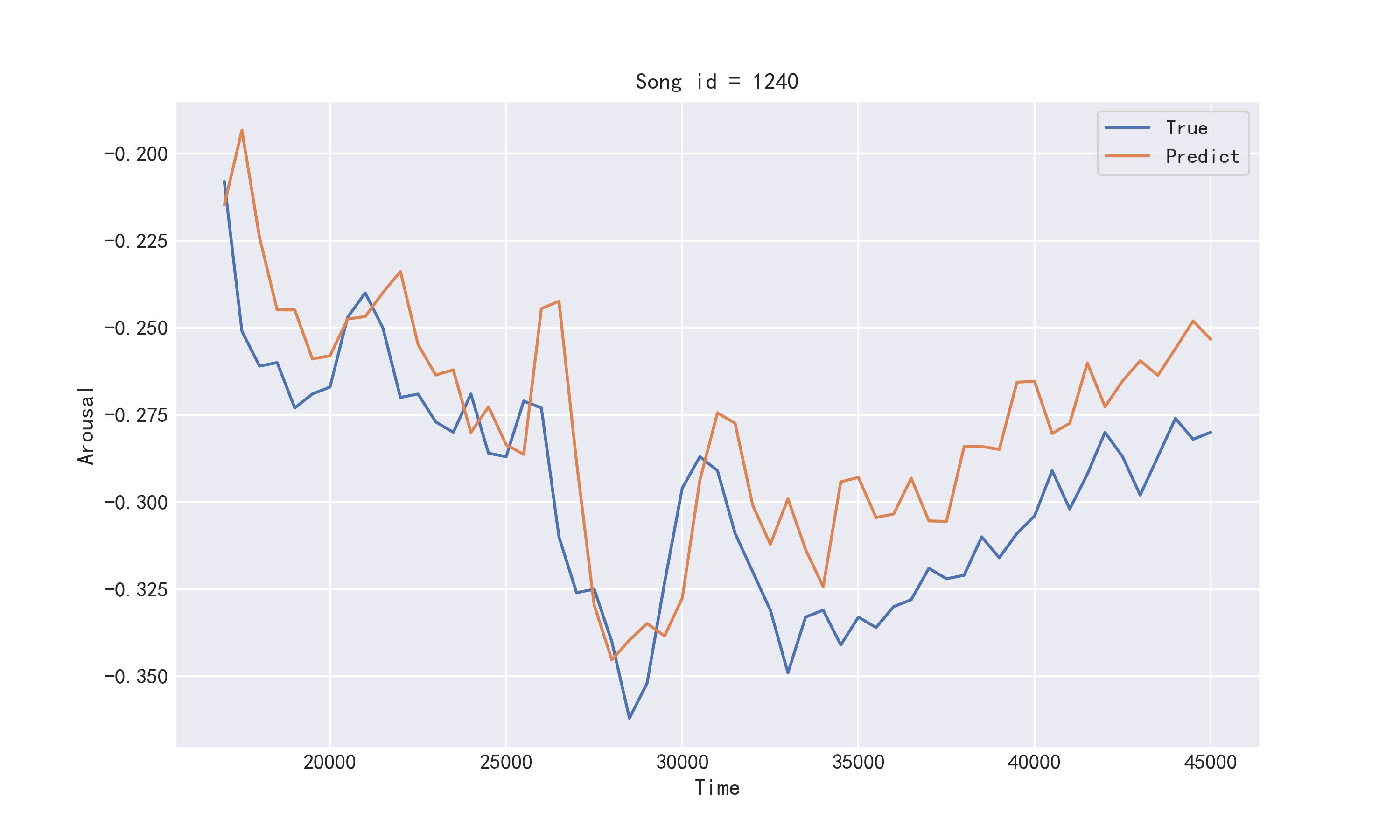

Supplement: S5 File — (ZIP) [file pone.0297712.s005.zip › All prediction results/prediction picture results(DEAM_100)/song_id_1240.jpg]

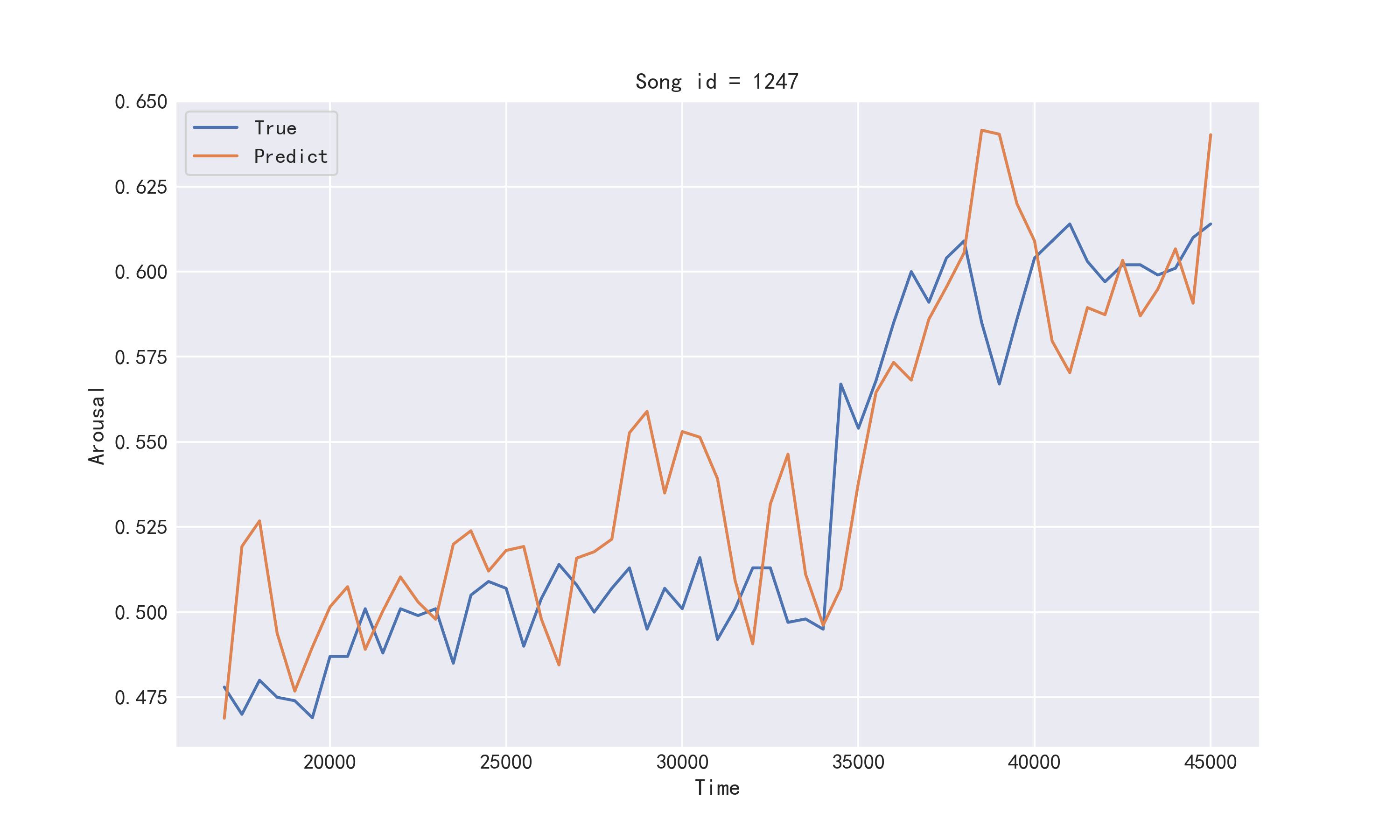

Supplement: S5 File — (ZIP) [file pone.0297712.s005.zip › All prediction results/prediction picture results(DEAM_100)/song_id_1247.jpg]

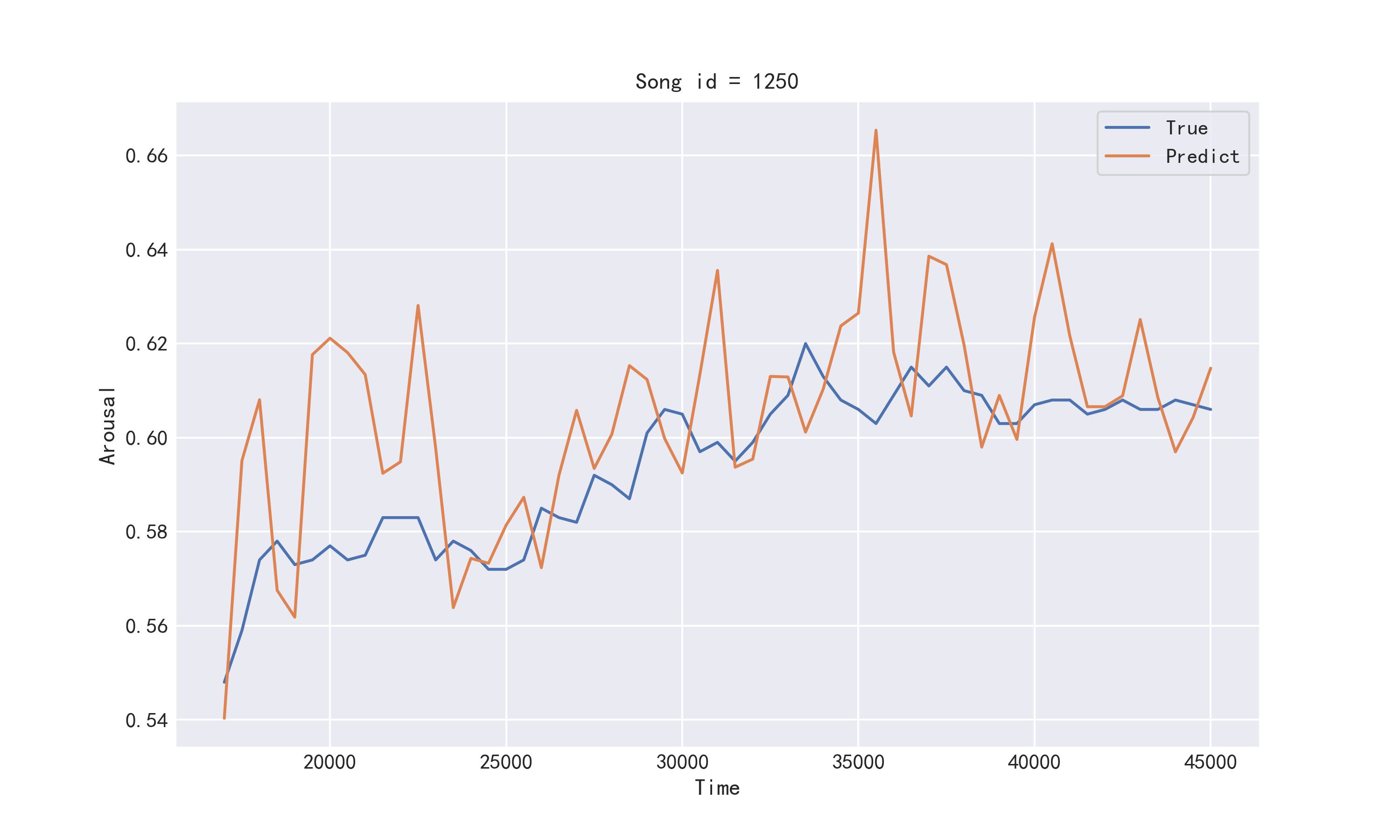

Supplement: S5 File — (ZIP) [file pone.0297712.s005.zip › All prediction results/prediction picture results(DEAM_100)/song_id_1250.jpg]

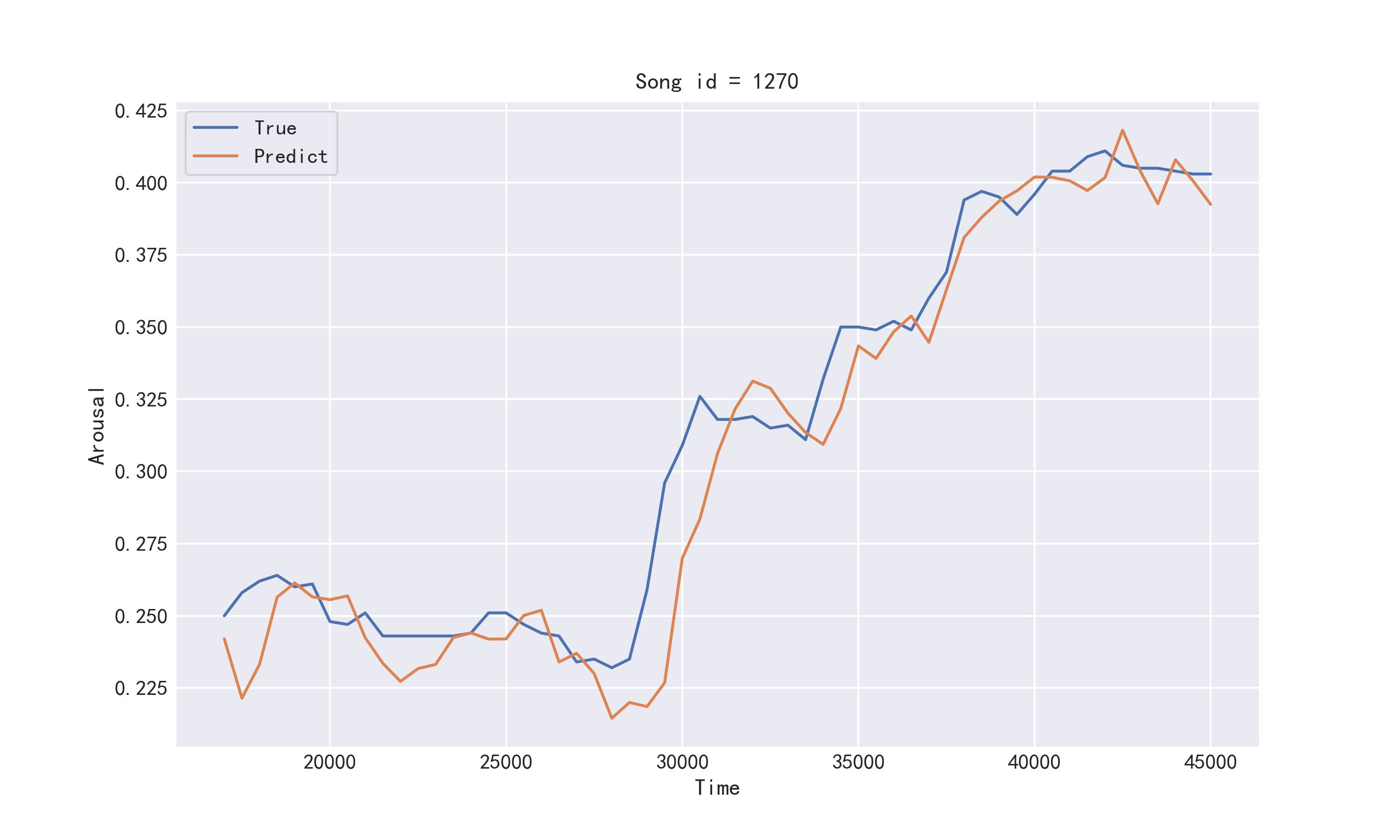

Supplement: S5 File — (ZIP) [file pone.0297712.s005.zip › All prediction results/prediction picture results(DEAM_100)/song_id_1270.jpg]

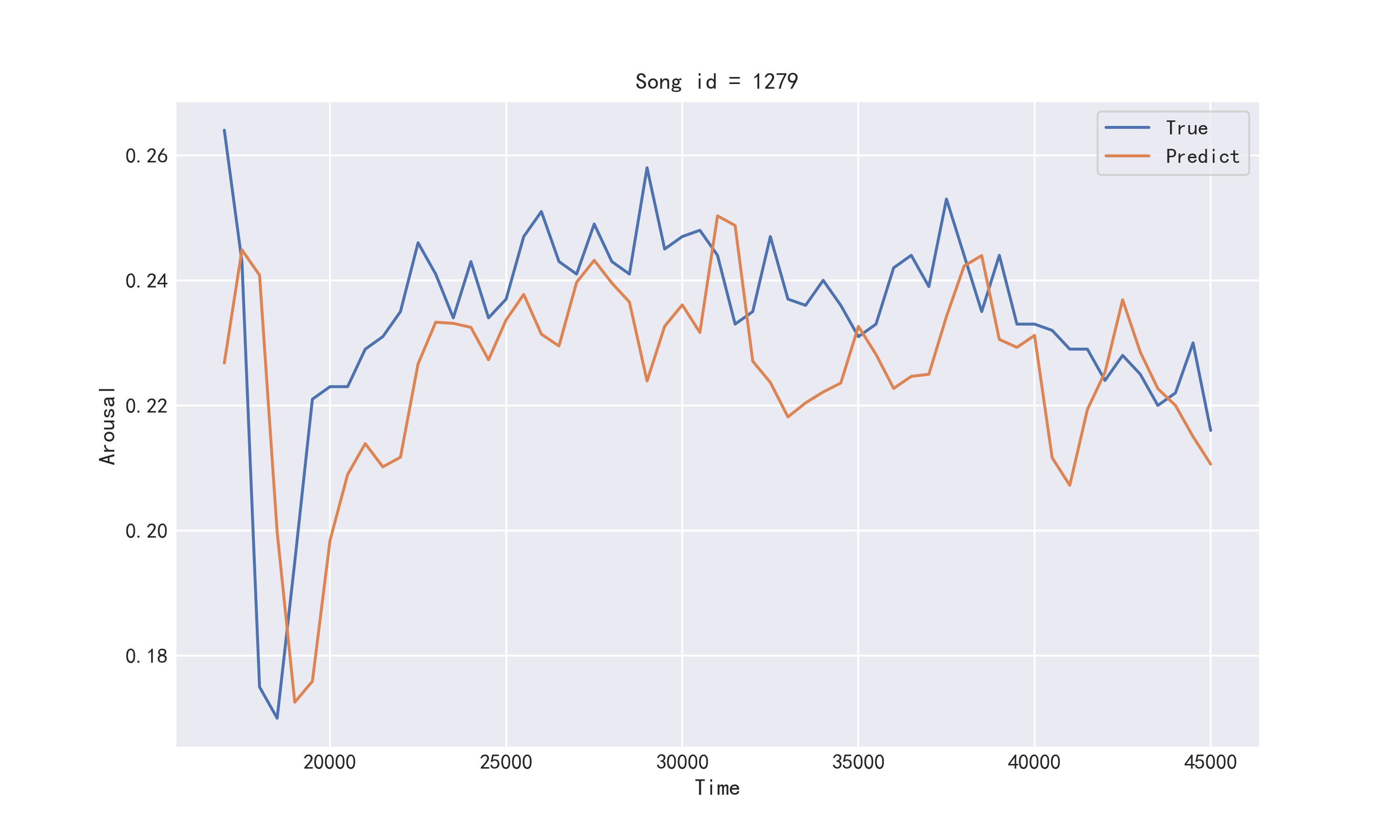

Supplement: S5 File — (ZIP) [file pone.0297712.s005.zip › All prediction results/prediction picture results(DEAM_100)/song_id_1279.jpg]

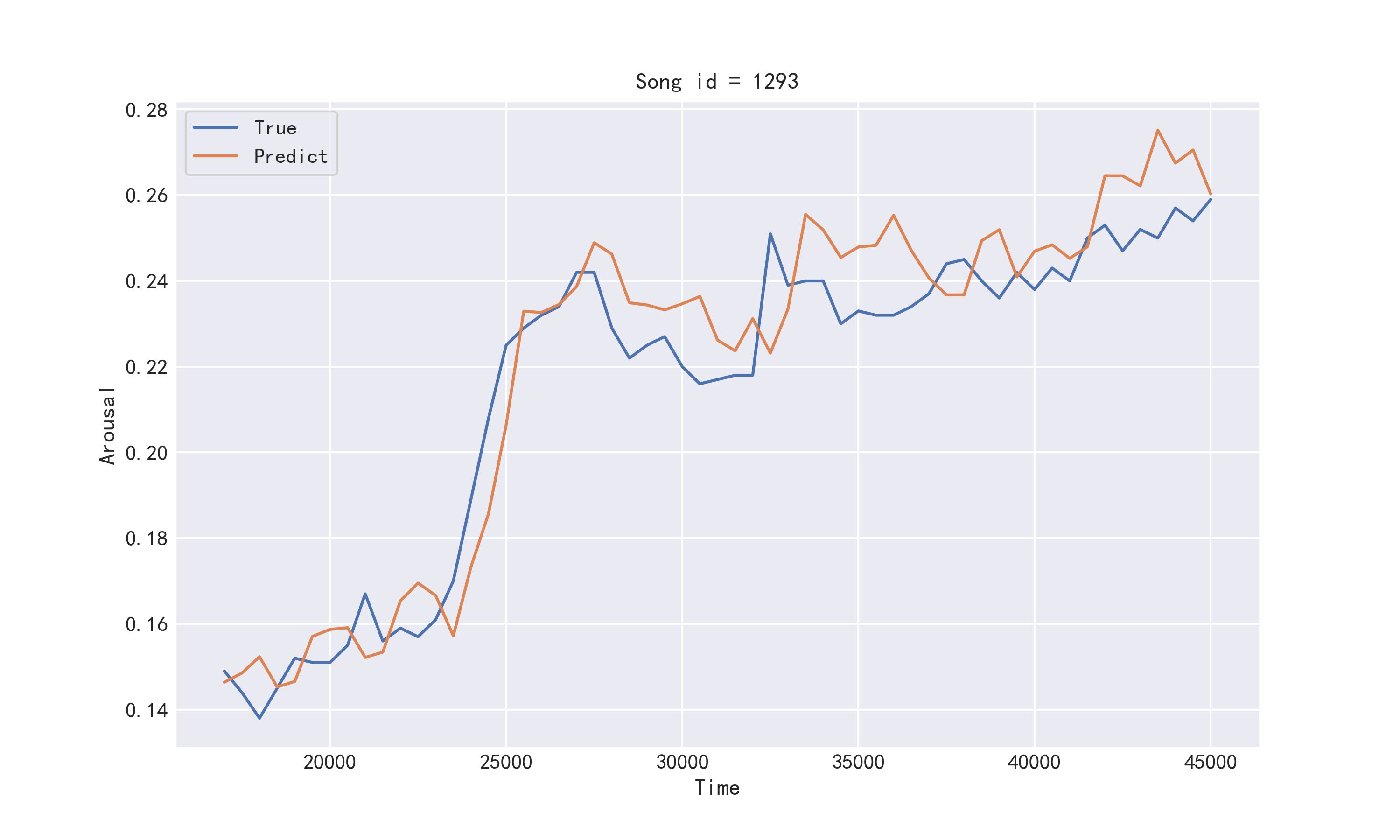

Supplement: S5 File — (ZIP) [file pone.0297712.s005.zip › All prediction results/prediction picture results(DEAM_100)/song_id_1293.jpg]

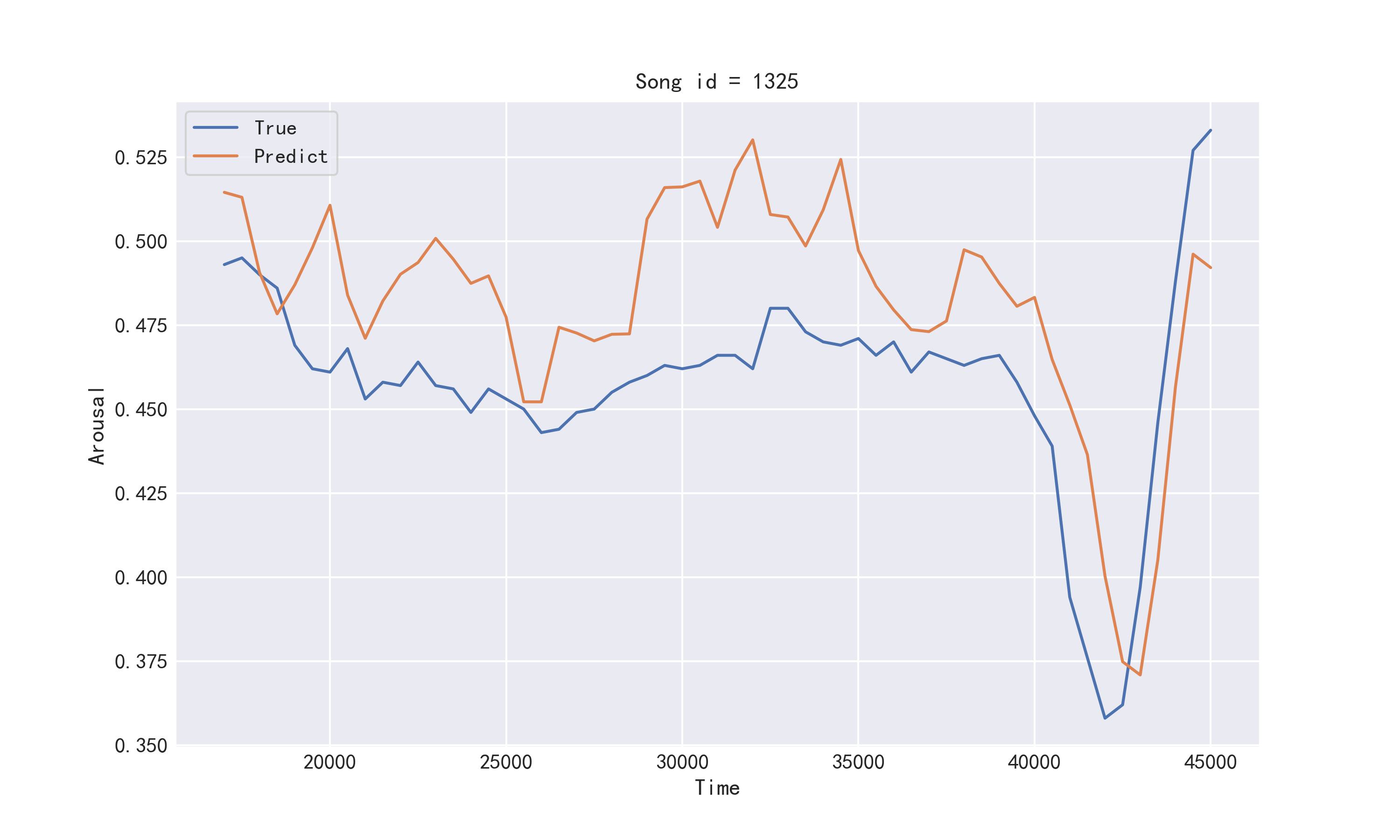

Supplement: S5 File — (ZIP) [file pone.0297712.s005.zip › All prediction results/prediction picture results(DEAM_100)/song_id_1325.jpg]

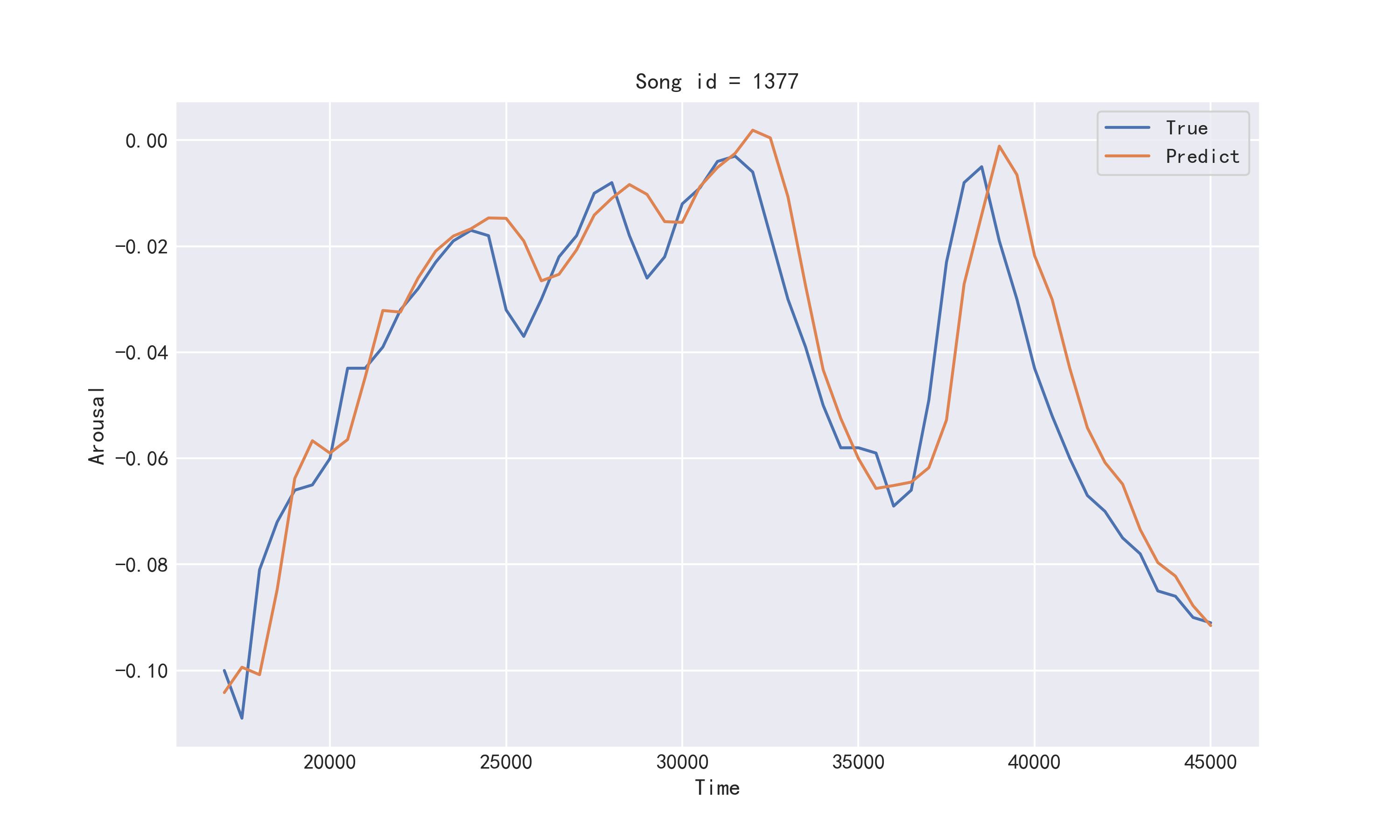

Supplement: S5 File — (ZIP) [file pone.0297712.s005.zip › All prediction results/prediction picture results(DEAM_100)/song_id_1377.jpg]

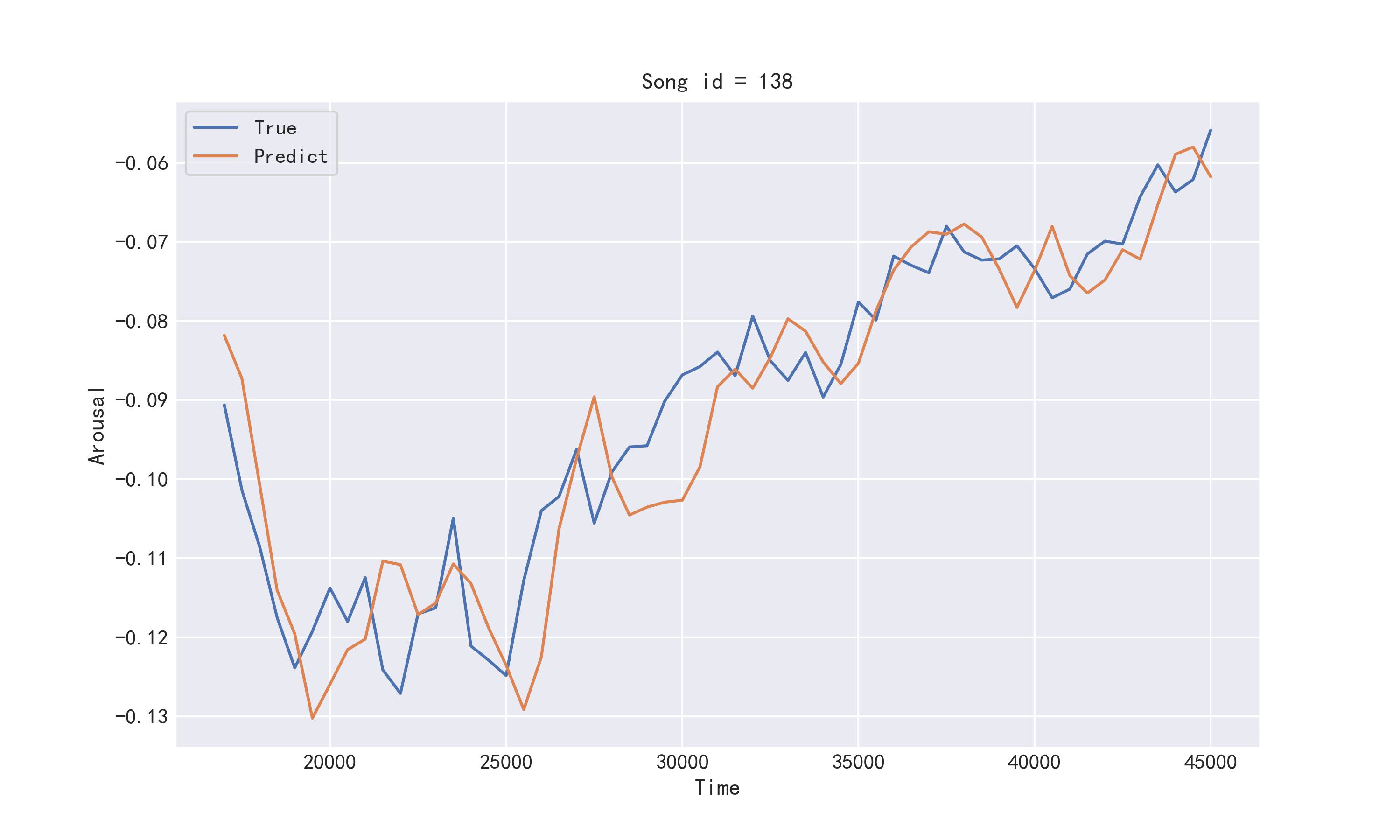

Supplement: S5 File — (ZIP) [file pone.0297712.s005.zip › All prediction results/prediction picture results(DEAM_100)/song_id_138.jpg]

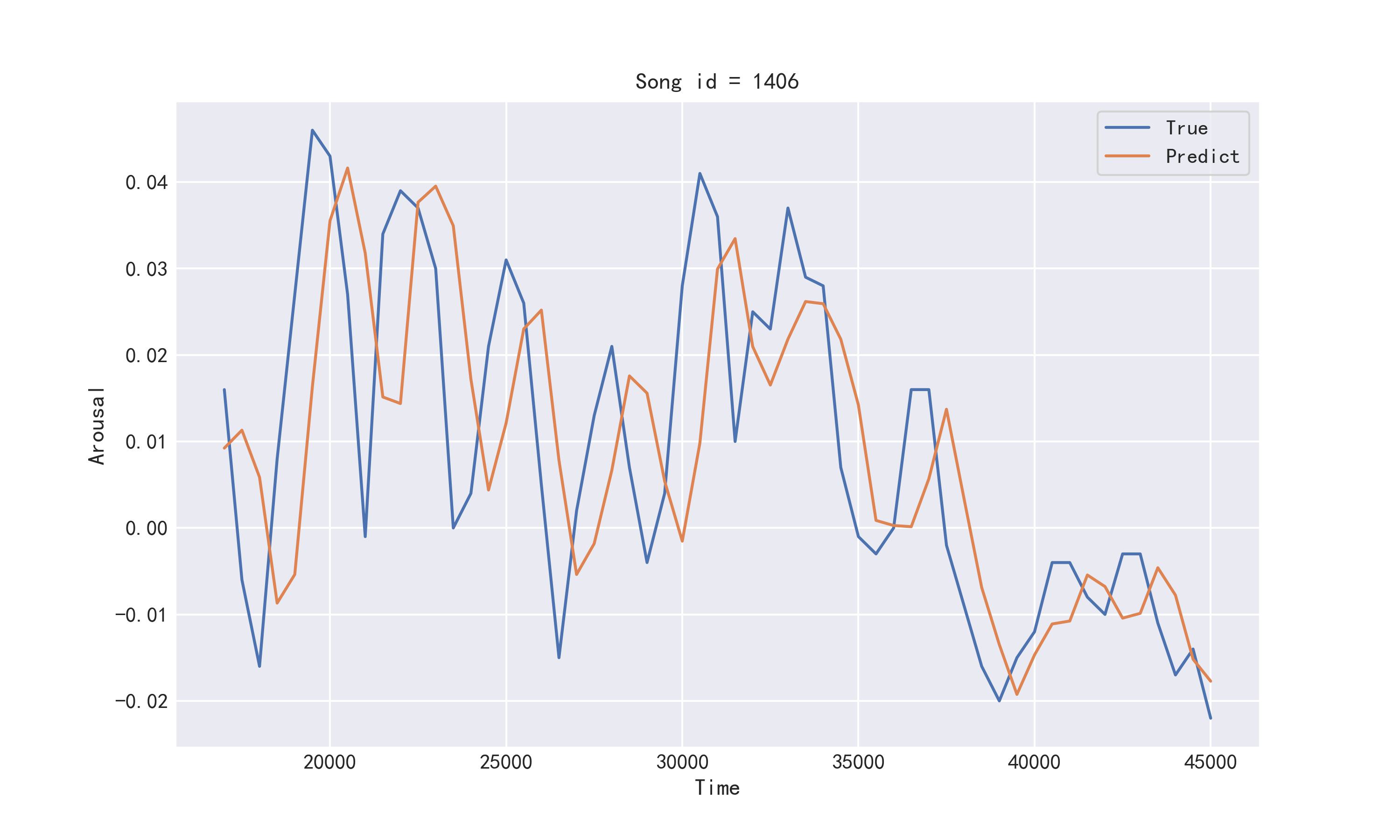

Supplement: S5 File — (ZIP) [file pone.0297712.s005.zip › All prediction results/prediction picture results(DEAM_100)/song_id_1406.jpg]

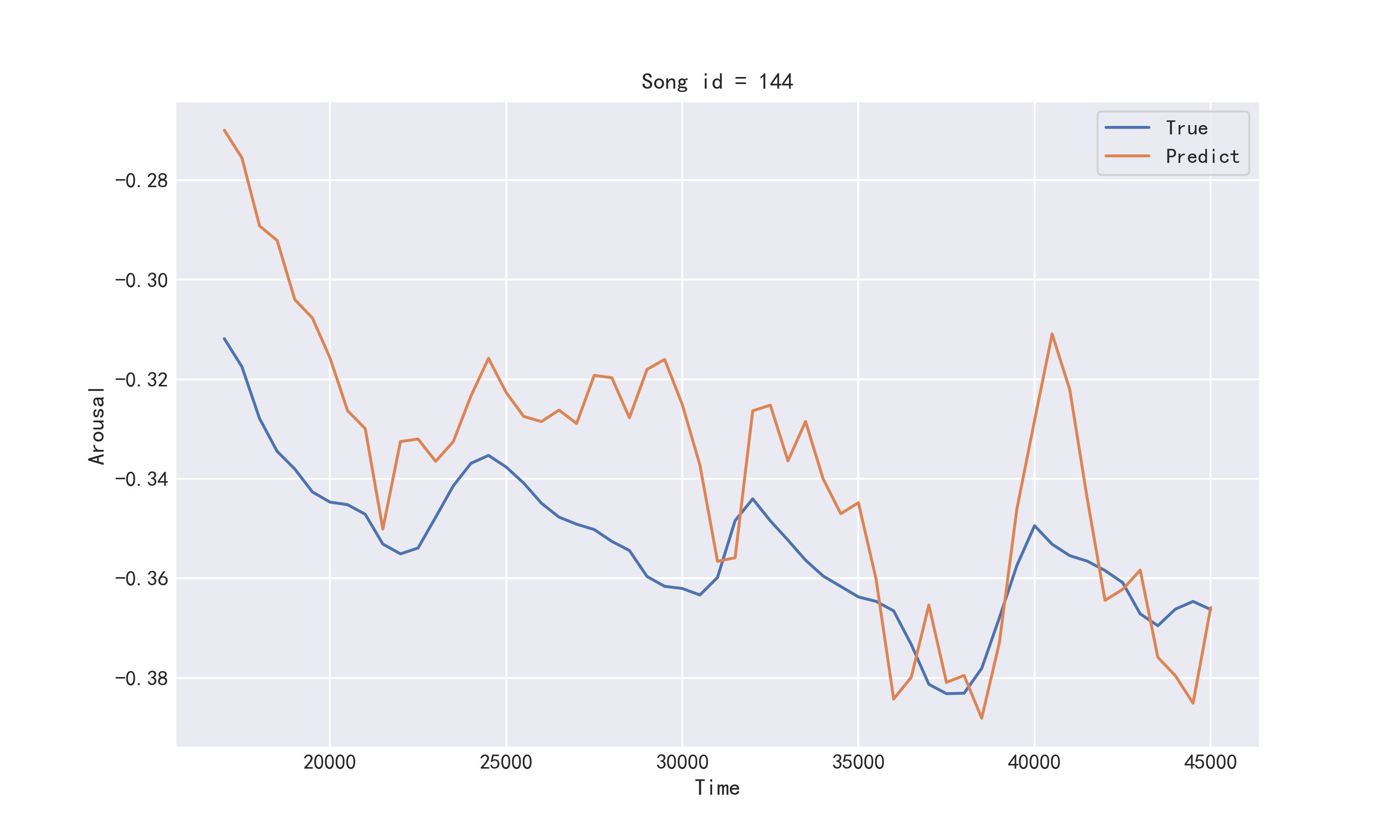

Supplement: S5 File — (ZIP) [file pone.0297712.s005.zip › All prediction results/prediction picture results(DEAM_100)/song_id_144.jpg]

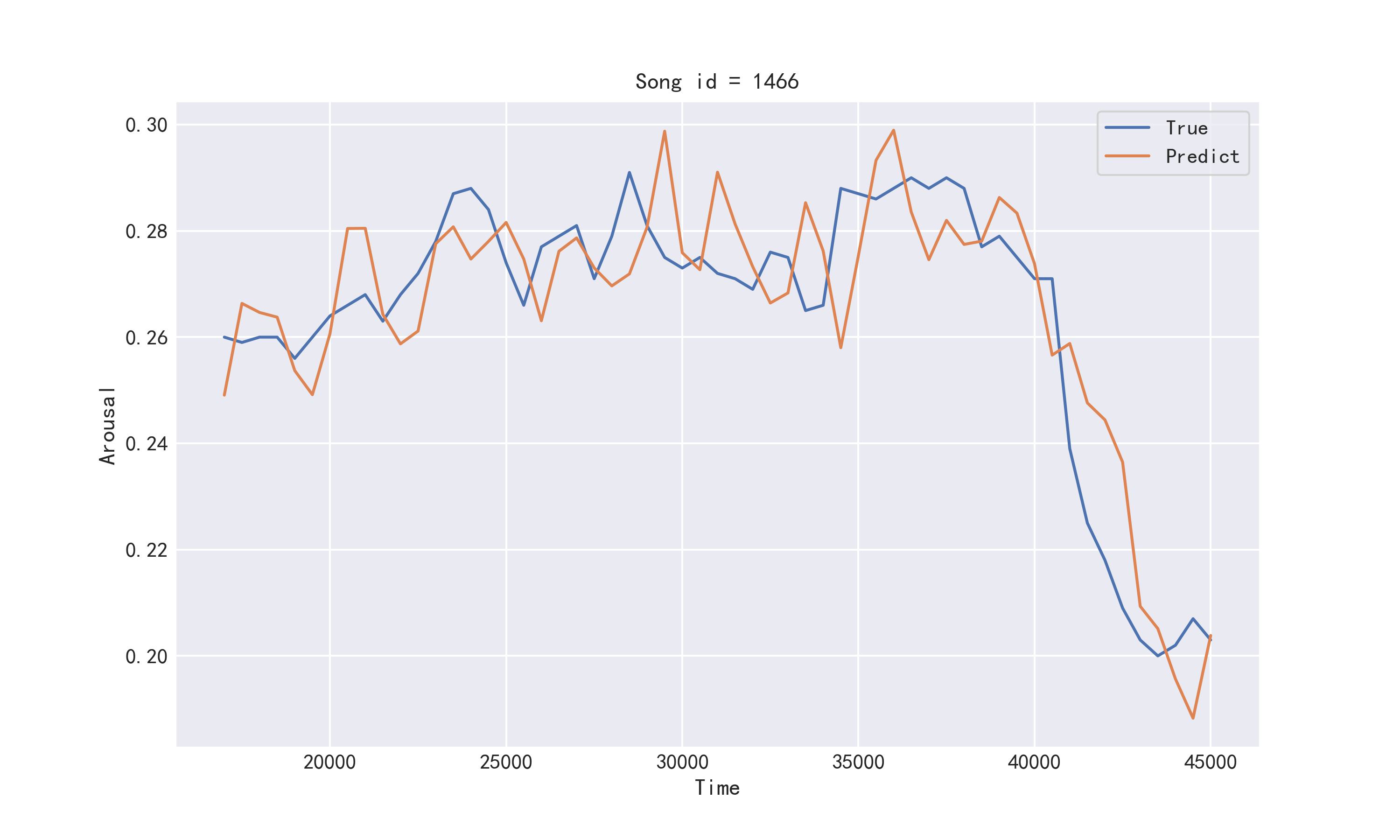

Supplement: S5 File — (ZIP) [file pone.0297712.s005.zip › All prediction results/prediction picture results(DEAM_100)/song_id_1466.jpg]

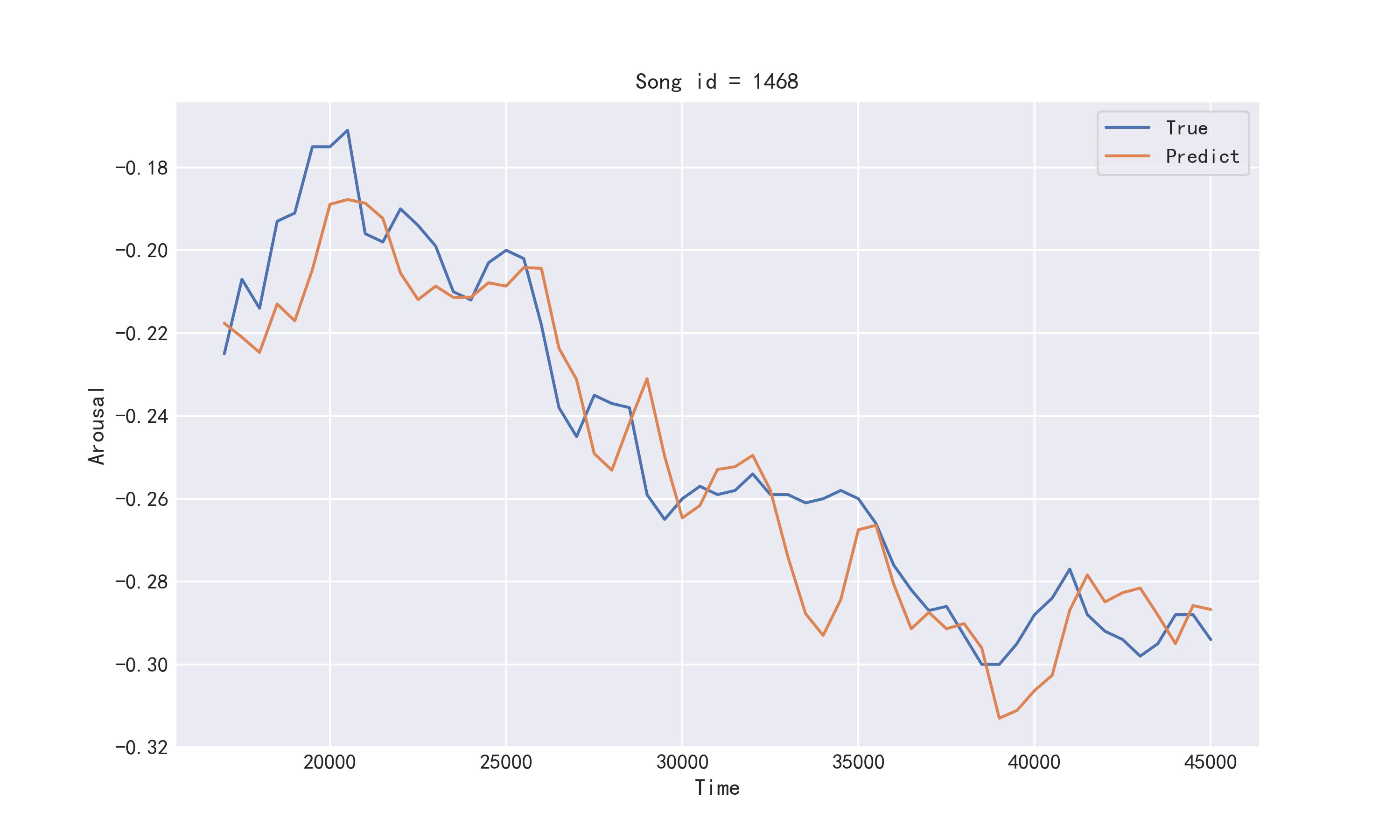

Supplement: S5 File — (ZIP) [file pone.0297712.s005.zip › All prediction results/prediction picture results(DEAM_100)/song_id_1468.jpg]

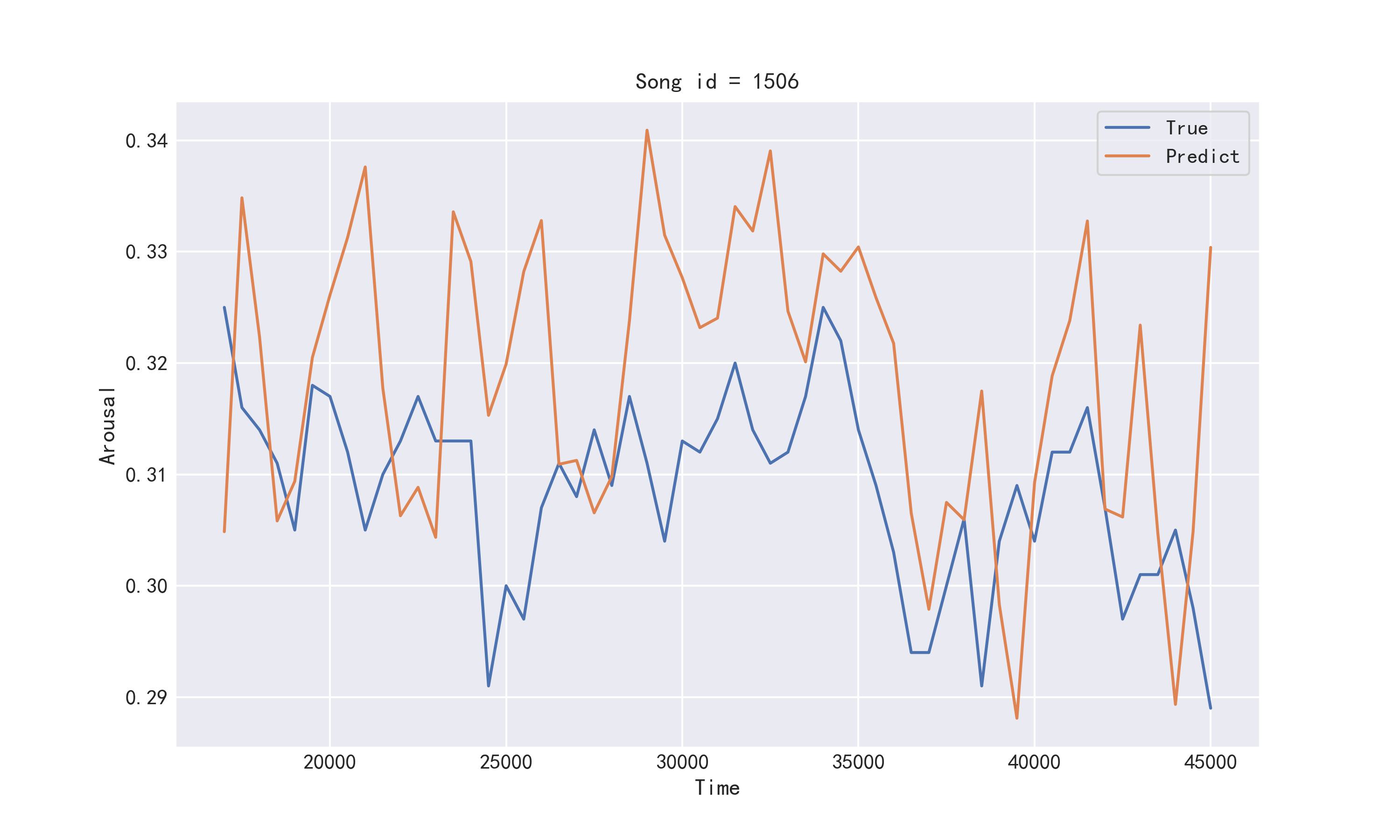

Supplement: S5 File — (ZIP) [file pone.0297712.s005.zip › All prediction results/prediction picture results(DEAM_100)/song_id_1506.jpg]

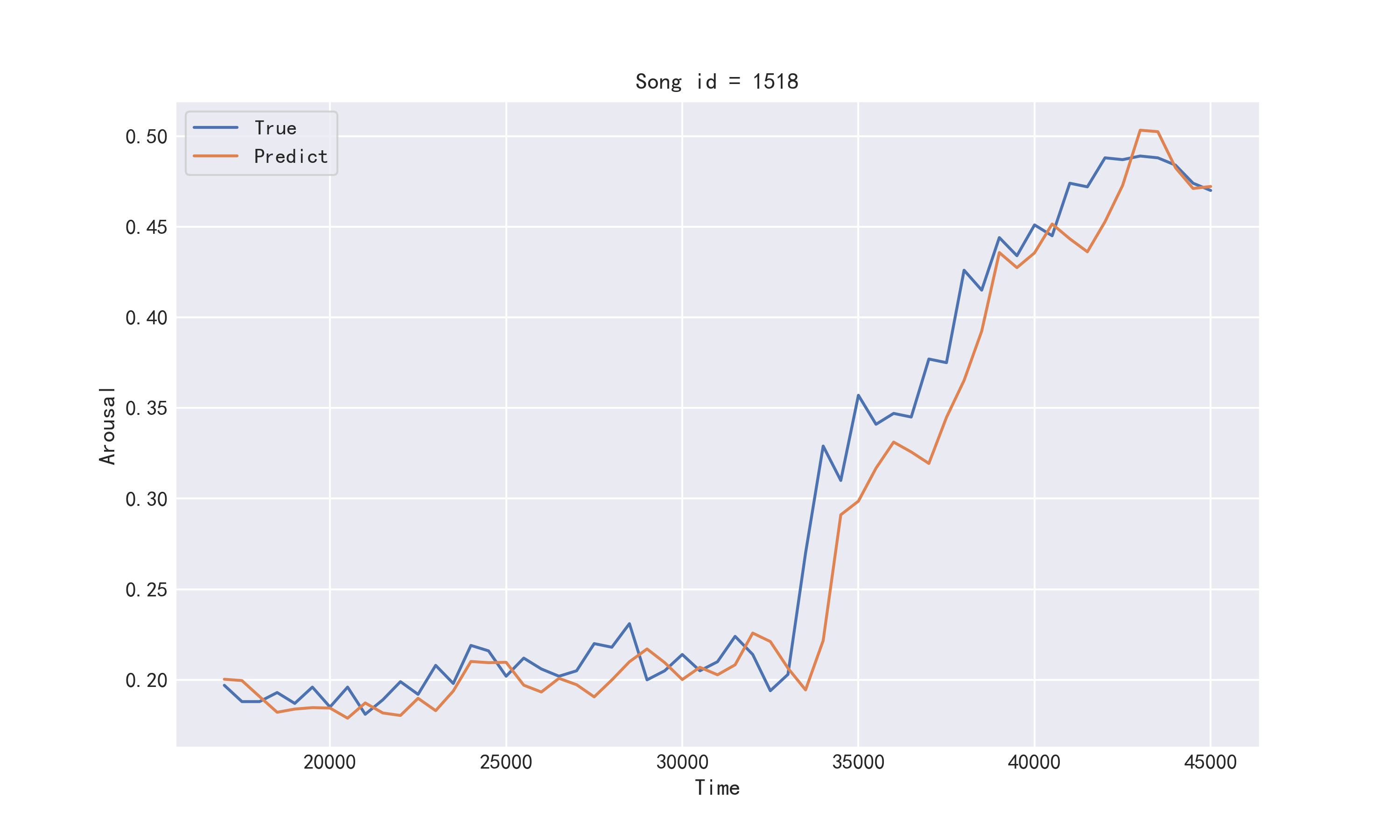

Supplement: S5 File — (ZIP) [file pone.0297712.s005.zip › All prediction results/prediction picture results(DEAM_100)/song_id_1518.jpg]

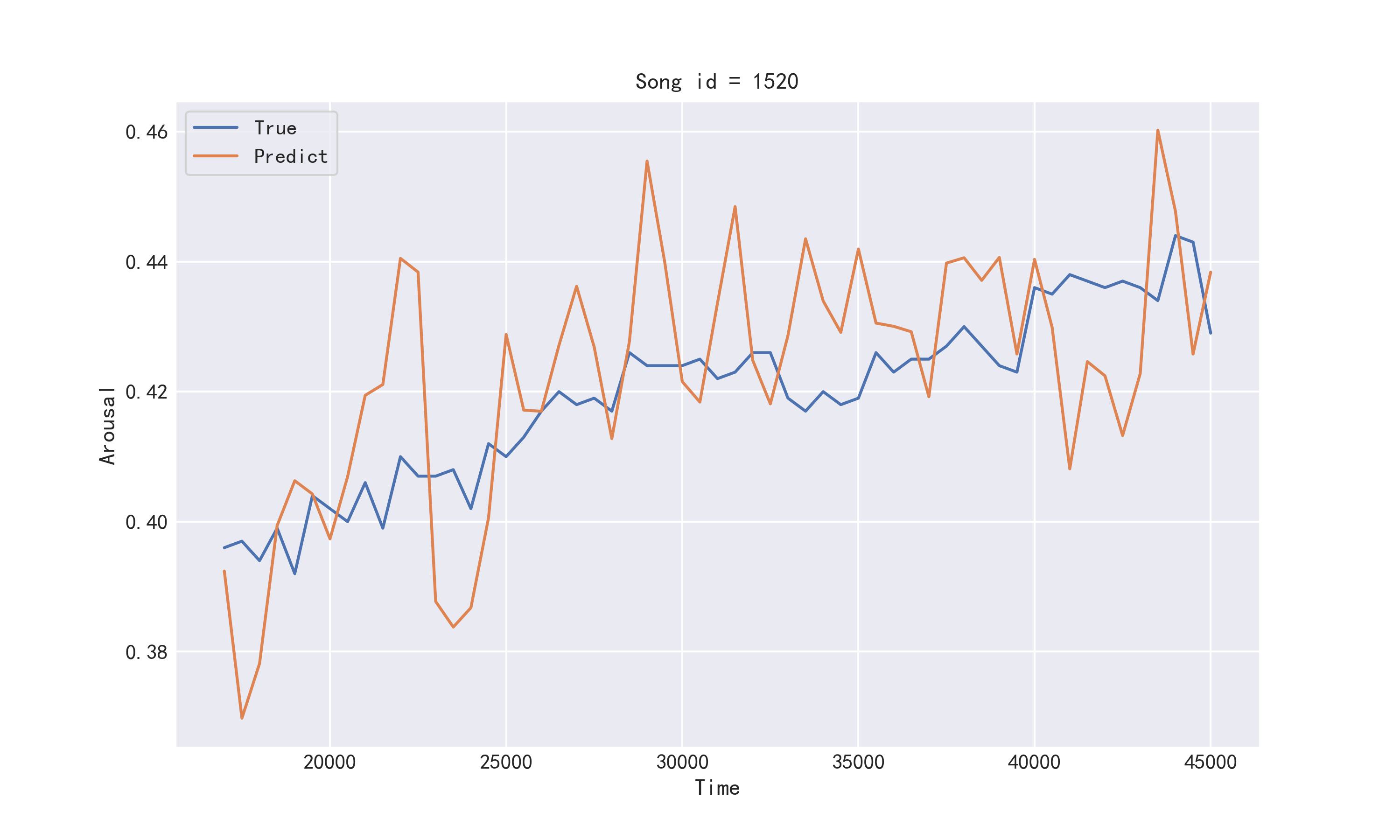

Supplement: S5 File — (ZIP) [file pone.0297712.s005.zip › All prediction results/prediction picture results(DEAM_100)/song_id_1520.jpg]

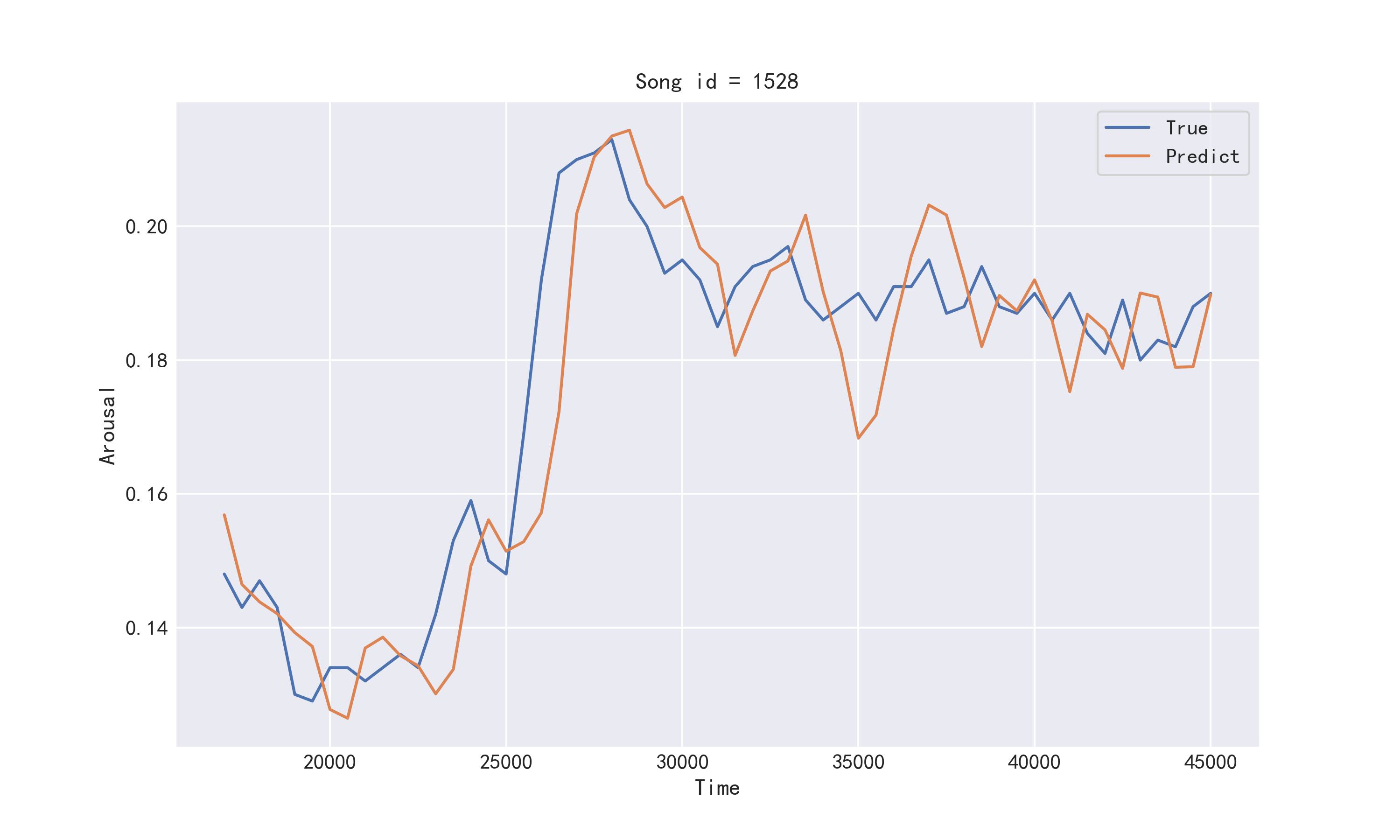

Supplement: S5 File — (ZIP) [file pone.0297712.s005.zip › All prediction results/prediction picture results(DEAM_100)/song_id_1528.jpg]

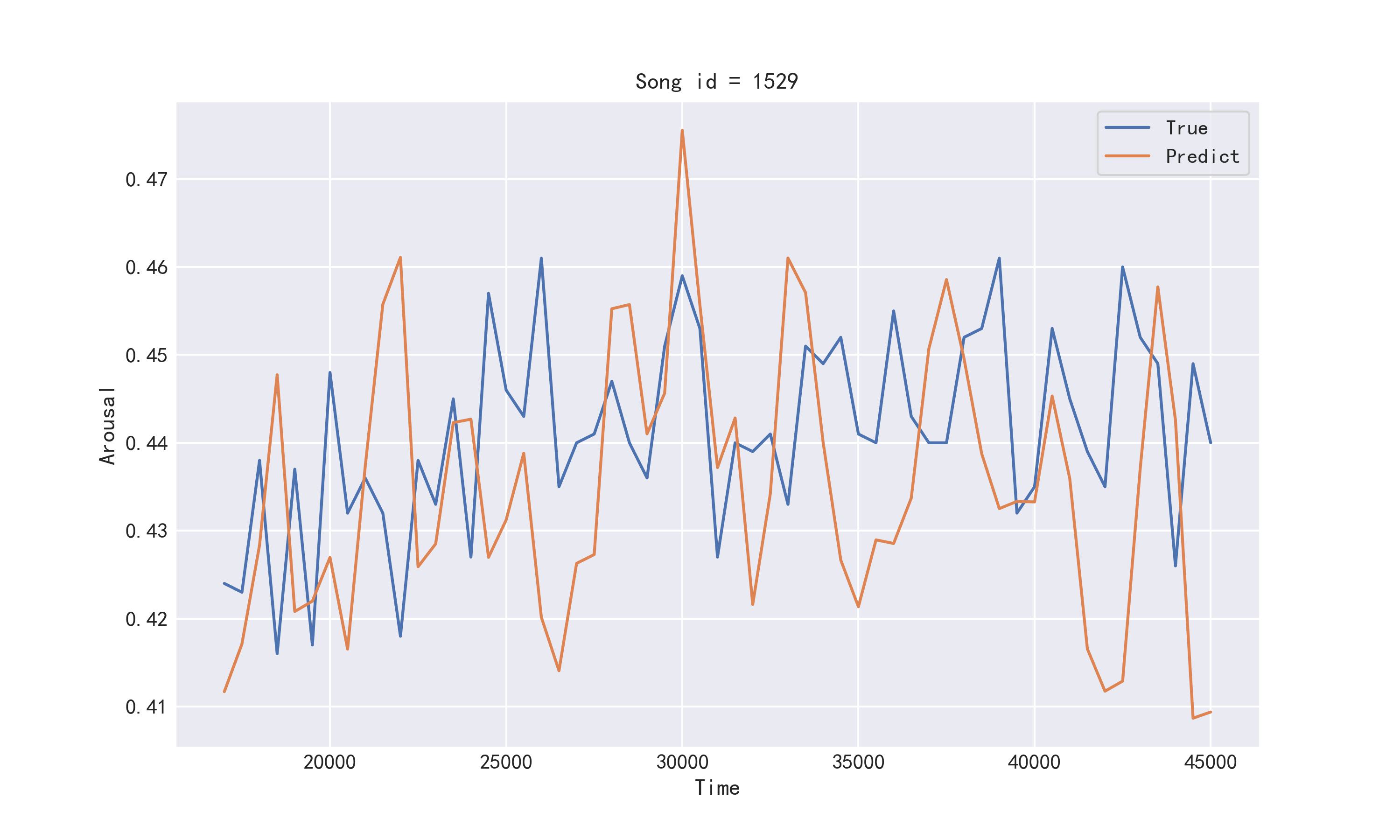

Supplement: S5 File — (ZIP) [file pone.0297712.s005.zip › All prediction results/prediction picture results(DEAM_100)/song_id_1529.jpg]

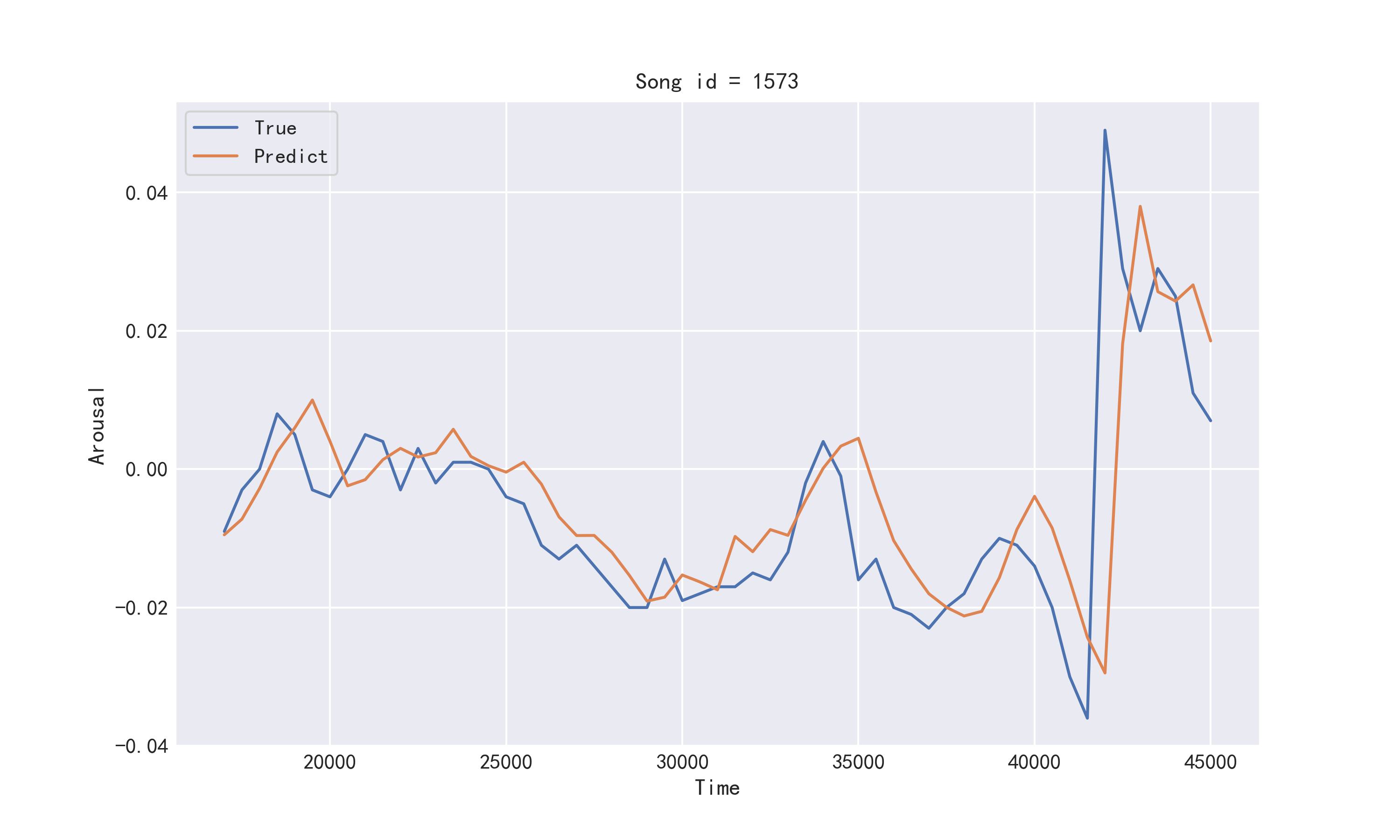

Supplement: S5 File — (ZIP) [file pone.0297712.s005.zip › All prediction results/prediction picture results(DEAM_100)/song_id_1573.jpg]

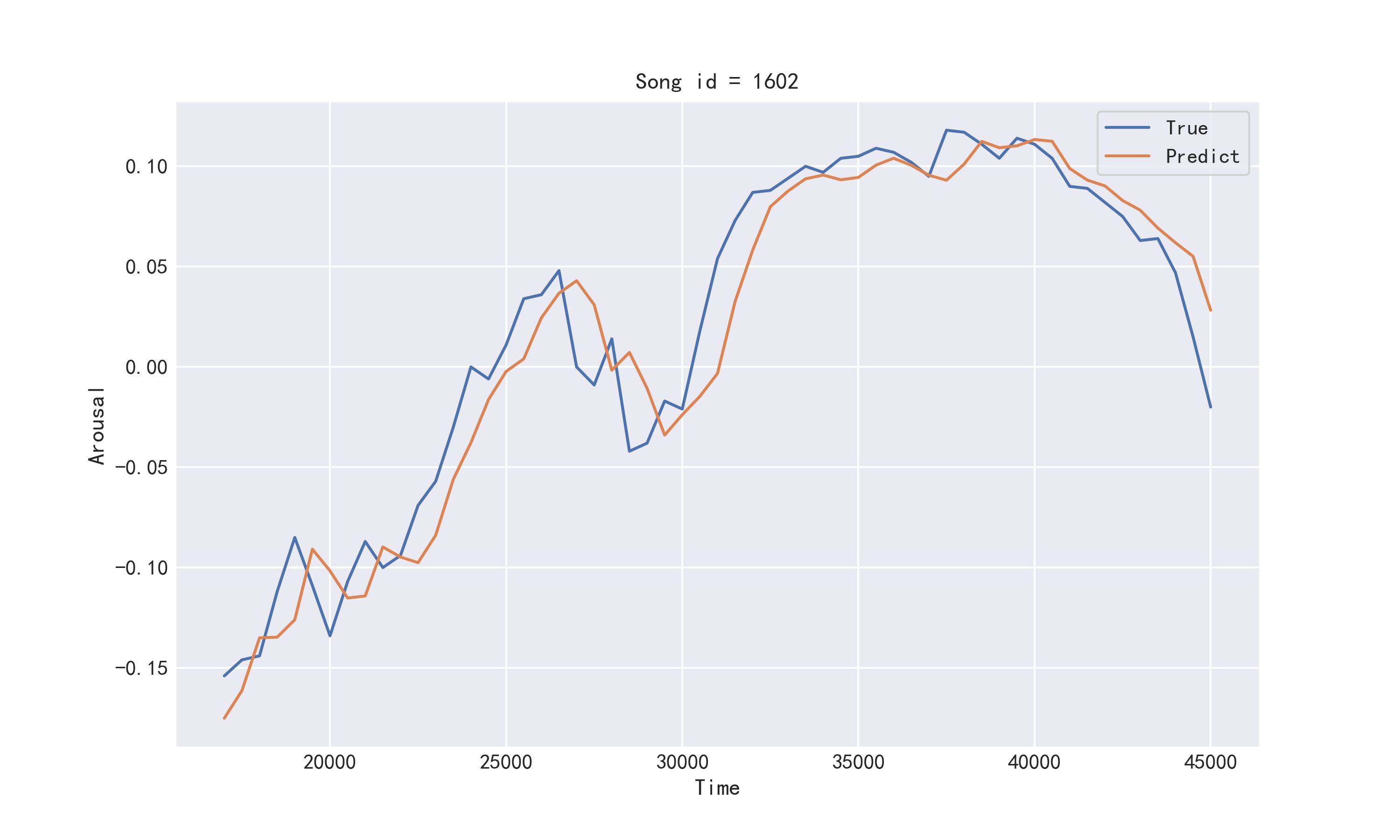

Supplement: S5 File — (ZIP) [file pone.0297712.s005.zip › All prediction results/prediction picture results(DEAM_100)/song_id_1602.jpg]

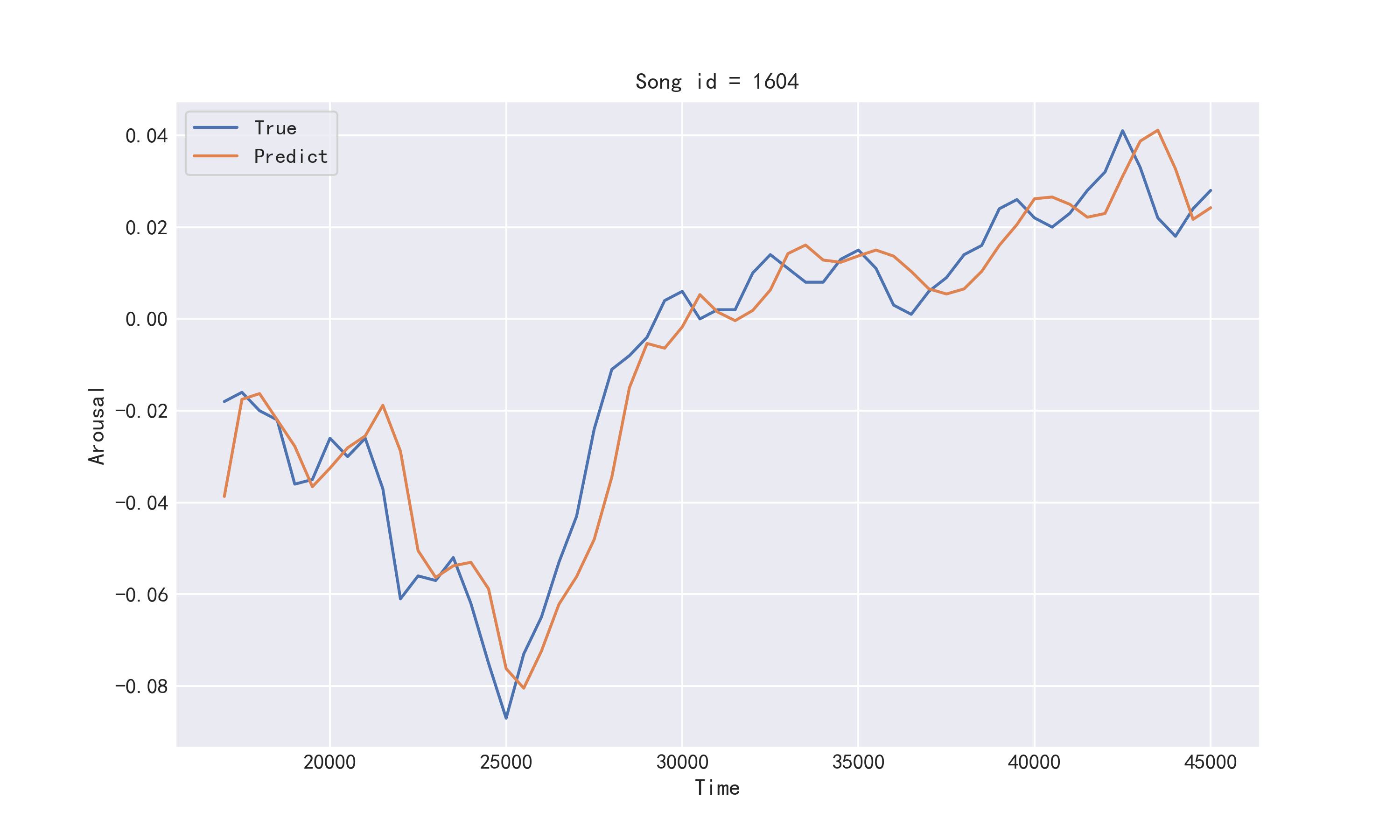

Supplement: S5 File — (ZIP) [file pone.0297712.s005.zip › All prediction results/prediction picture results(DEAM_100)/song_id_1604.jpg]

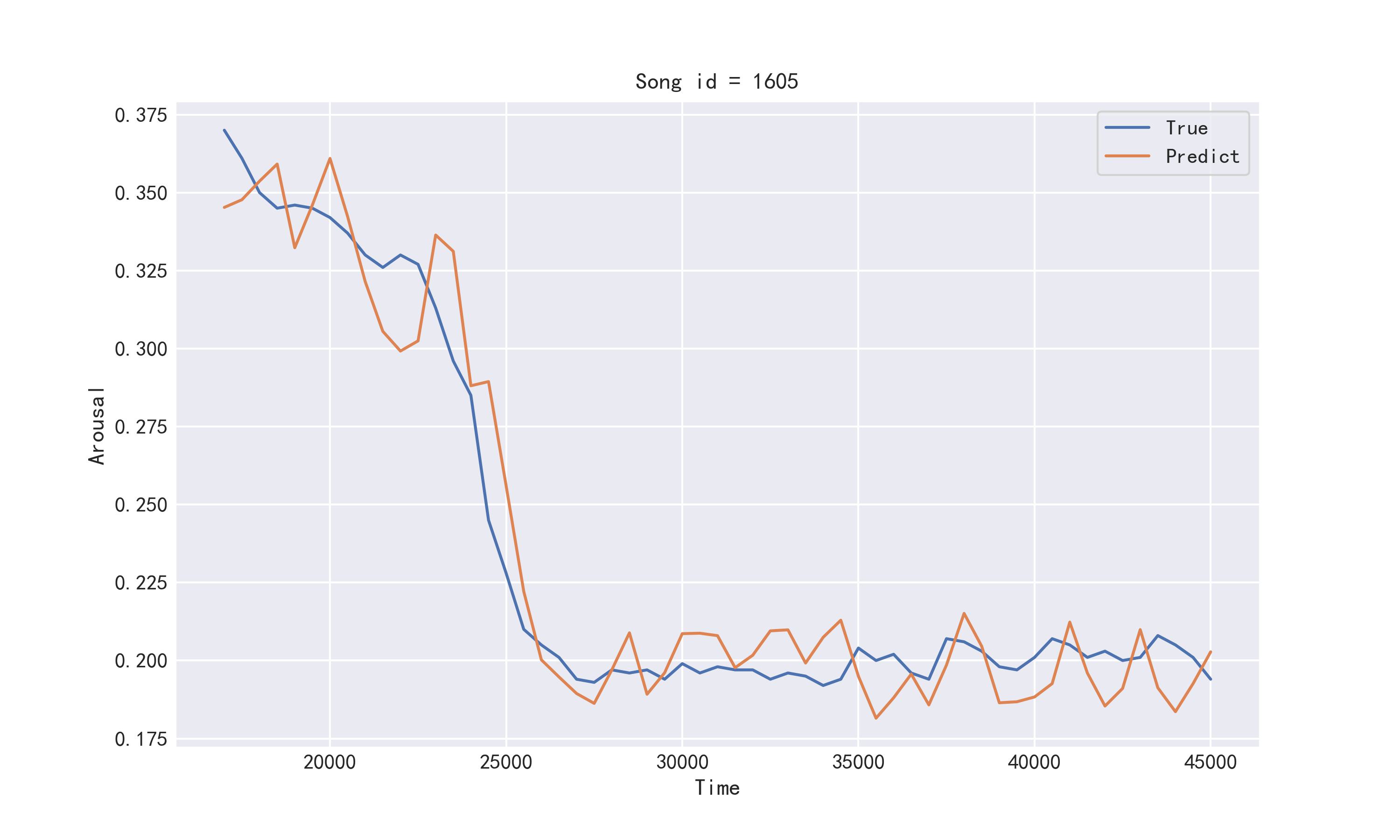

Supplement: S5 File — (ZIP) [file pone.0297712.s005.zip › All prediction results/prediction picture results(DEAM_100)/song_id_1605.jpg]

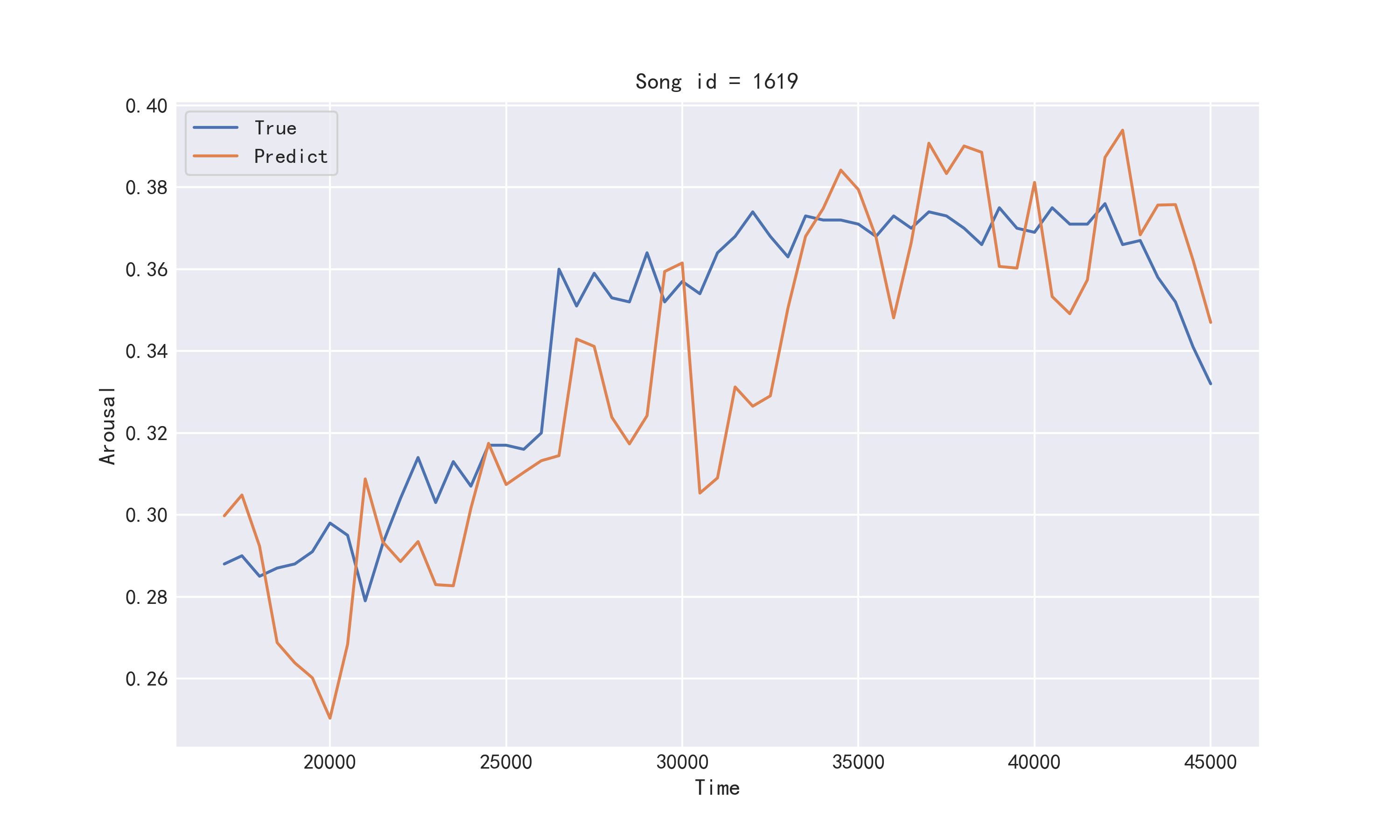

Supplement: S5 File — (ZIP) [file pone.0297712.s005.zip › All prediction results/prediction picture results(DEAM_100)/song_id_1619.jpg]

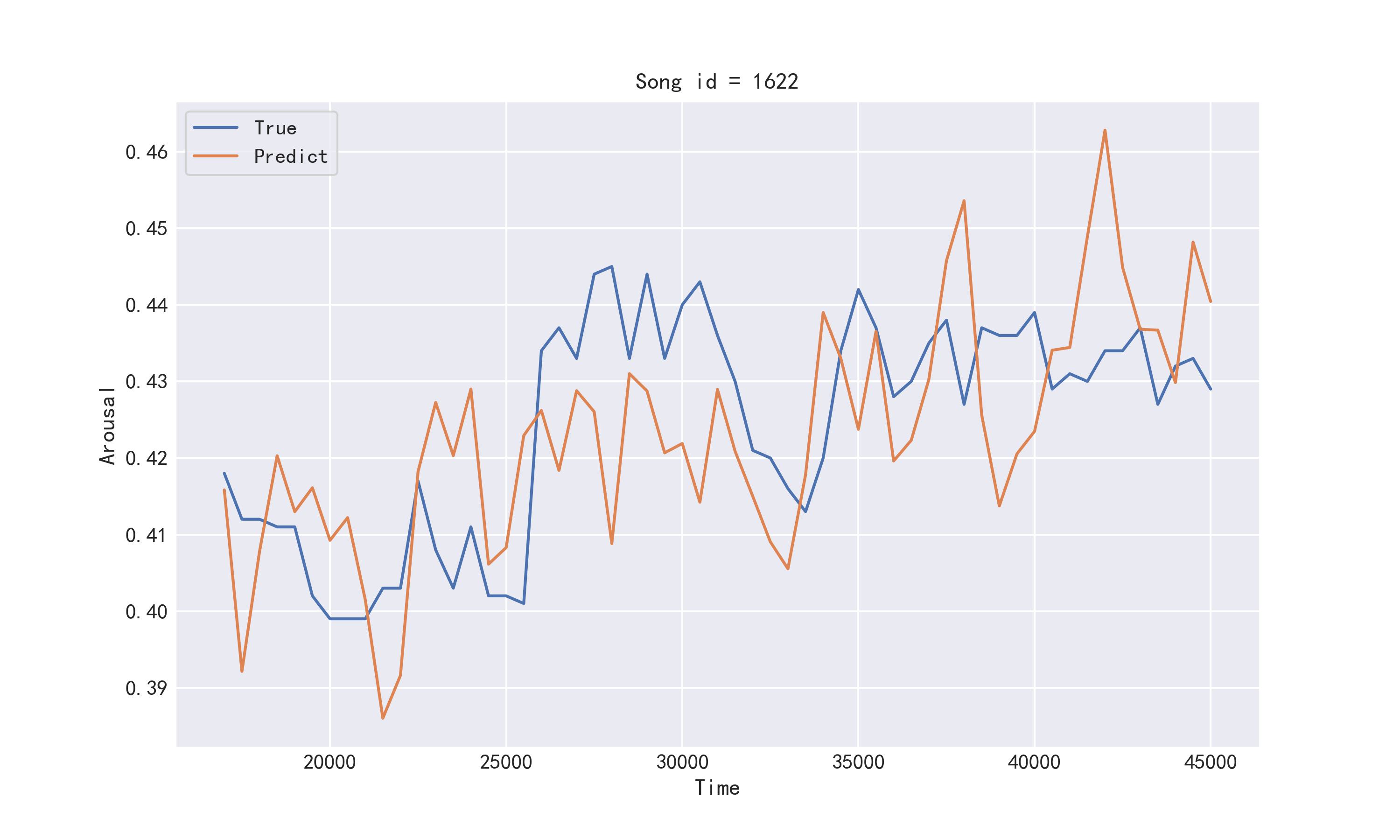

Supplement: S5 File — (ZIP) [file pone.0297712.s005.zip › All prediction results/prediction picture results(DEAM_100)/song_id_1622.jpg]

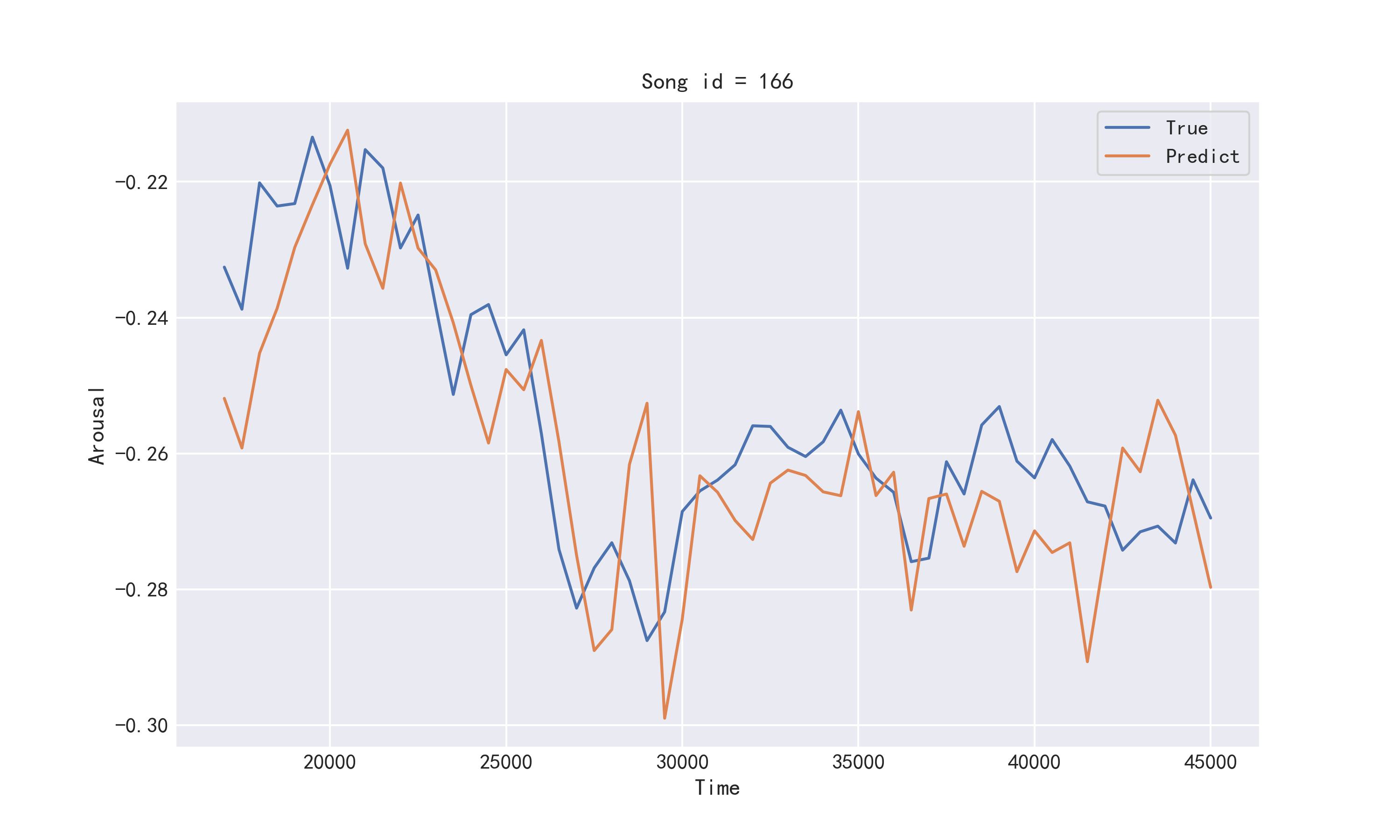

Supplement: S5 File — (ZIP) [file pone.0297712.s005.zip › All prediction results/prediction picture results(DEAM_100)/song_id_166.jpg]

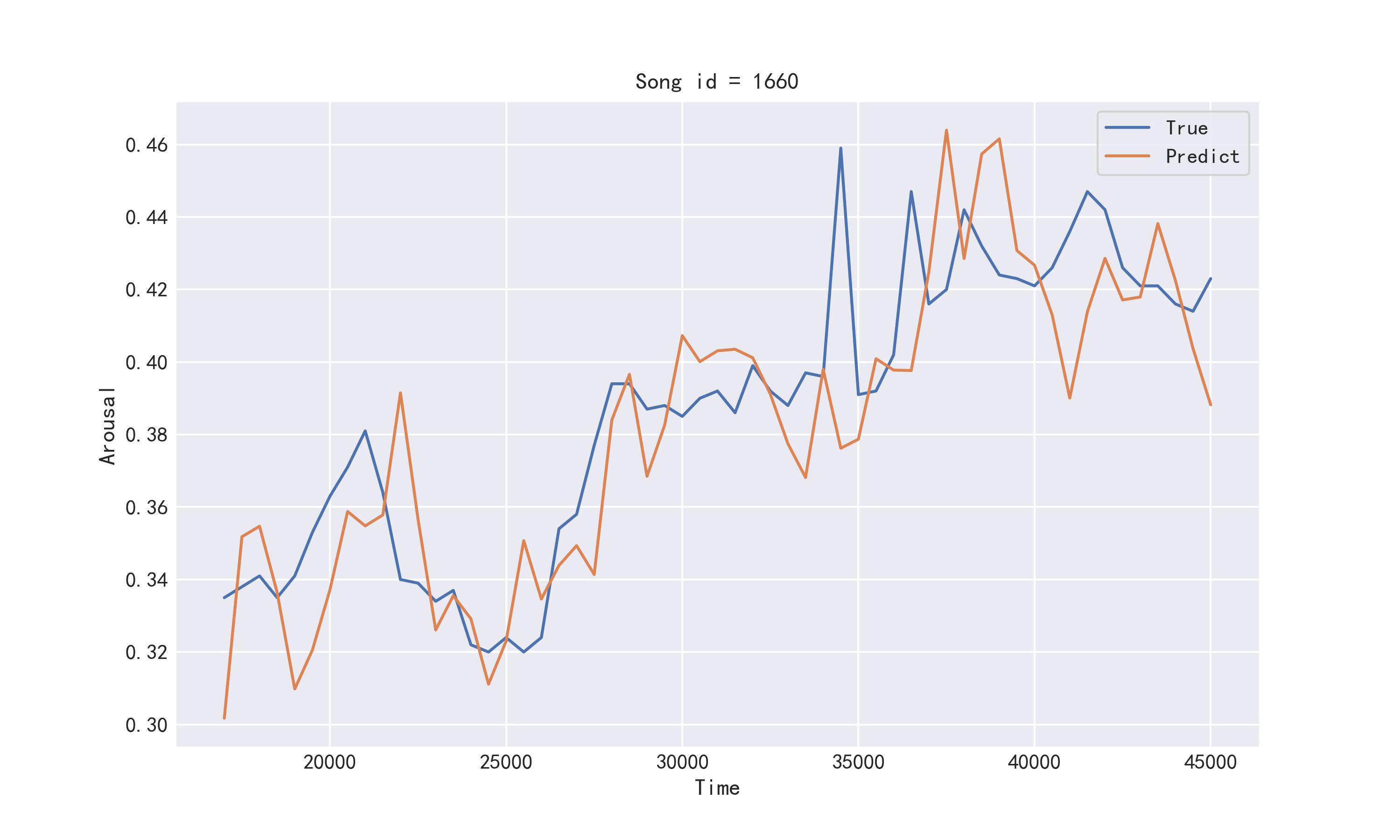

Supplement: S5 File — (ZIP) [file pone.0297712.s005.zip › All prediction results/prediction picture results(DEAM_100)/song_id_1660.jpg]

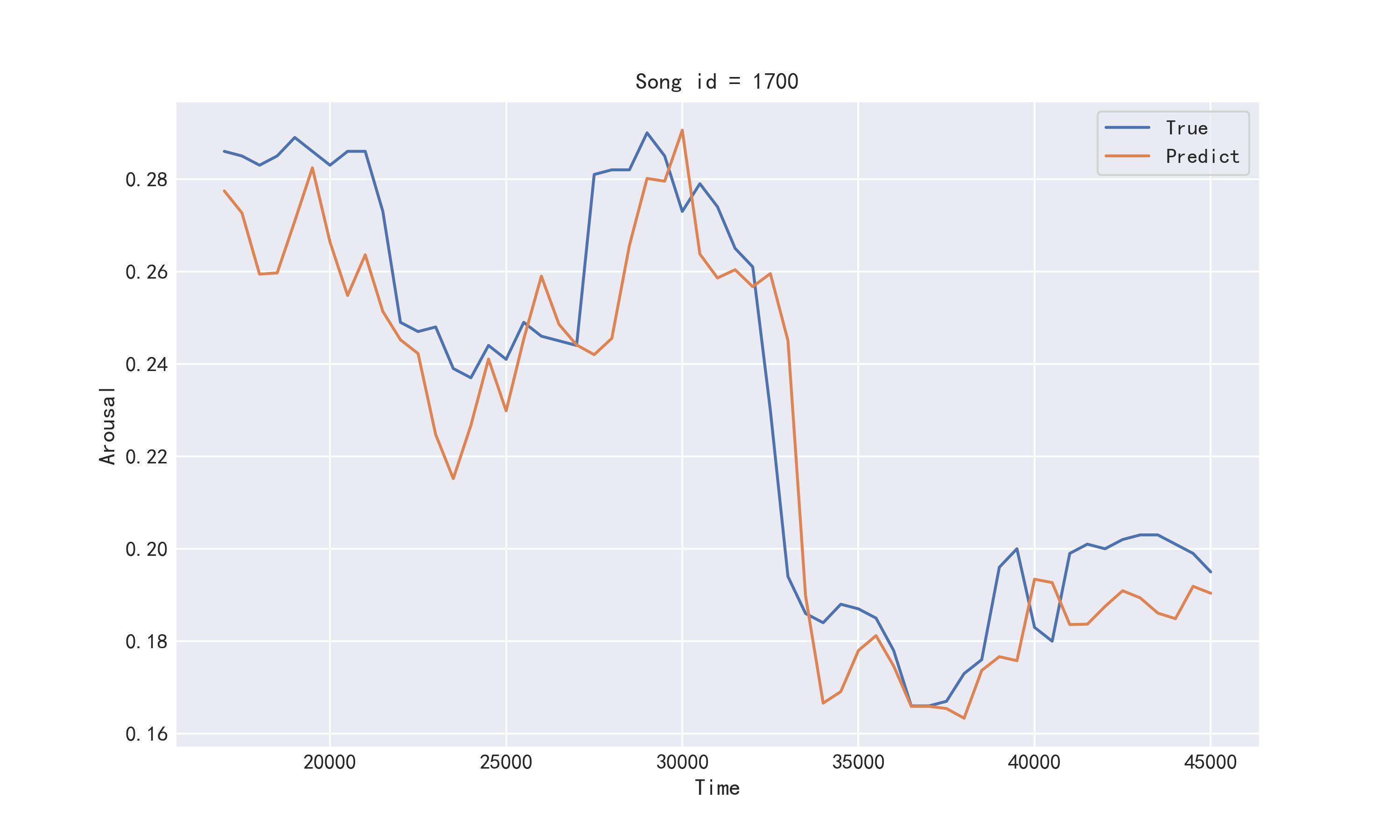

Supplement: S5 File — (ZIP) [file pone.0297712.s005.zip › All prediction results/prediction picture results(DEAM_100)/song_id_1700.jpg]

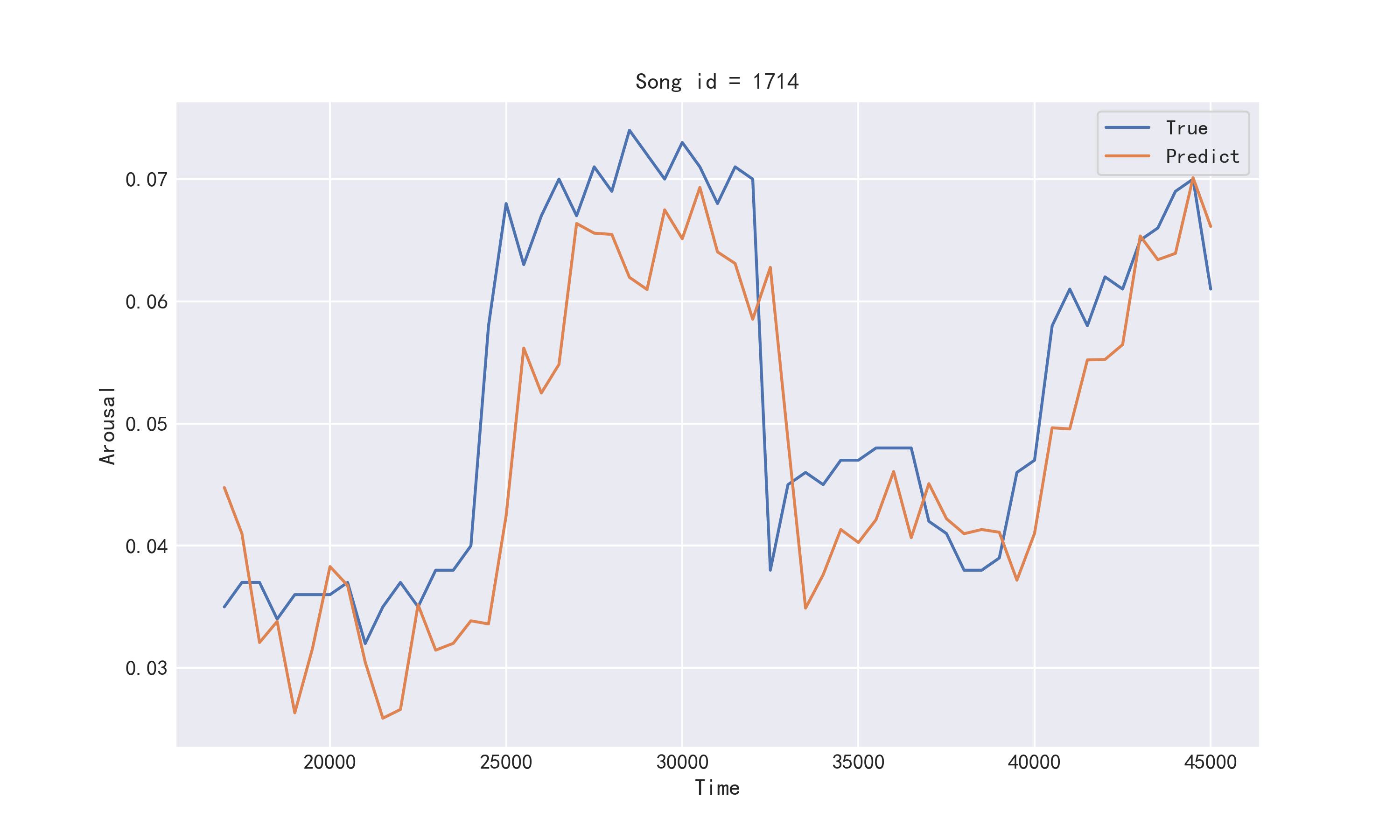

Supplement: S5 File — (ZIP) [file pone.0297712.s005.zip › All prediction results/prediction picture results(DEAM_100)/song_id_1714.jpg]

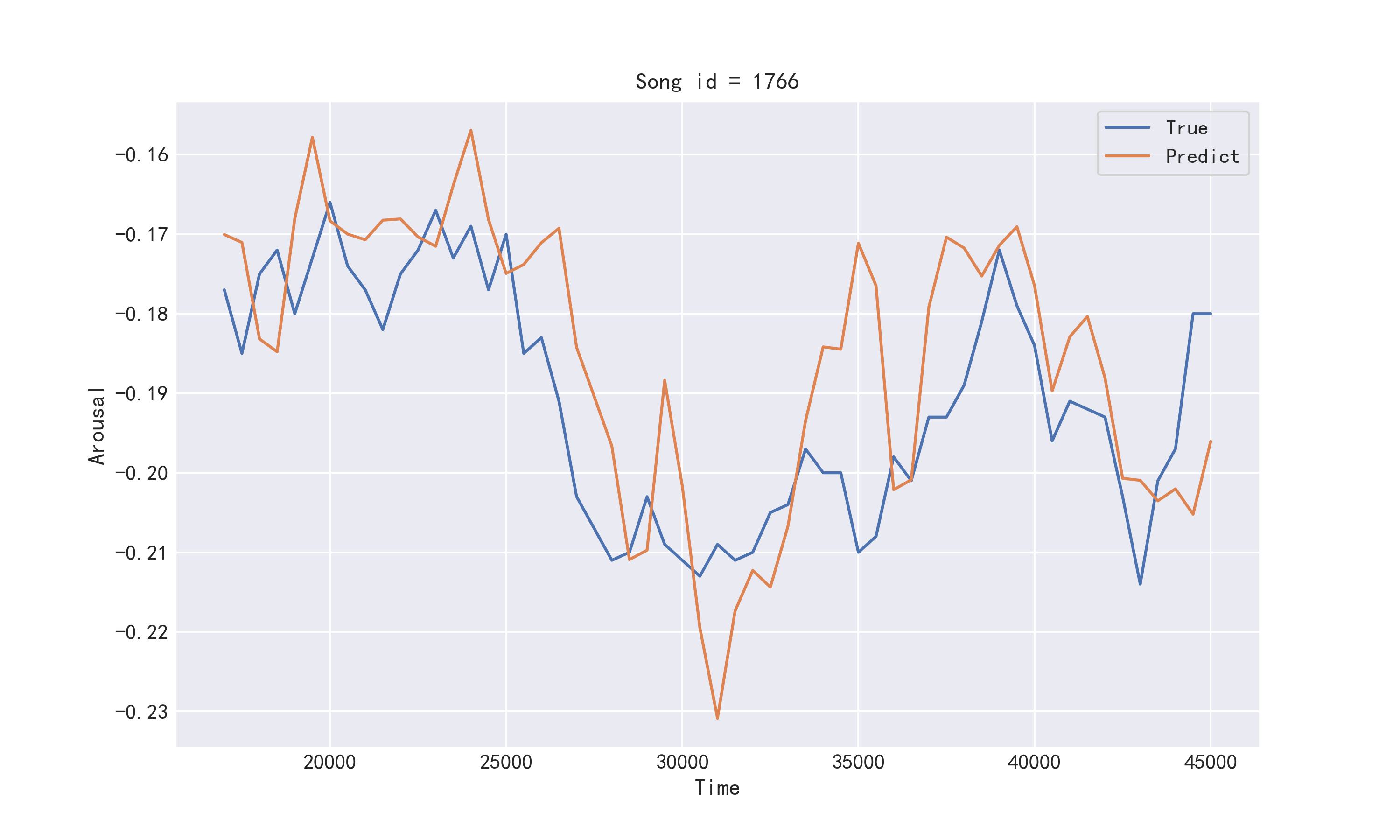

Supplement: S5 File — (ZIP) [file pone.0297712.s005.zip › All prediction results/prediction picture results(DEAM_100)/song_id_1766.jpg]

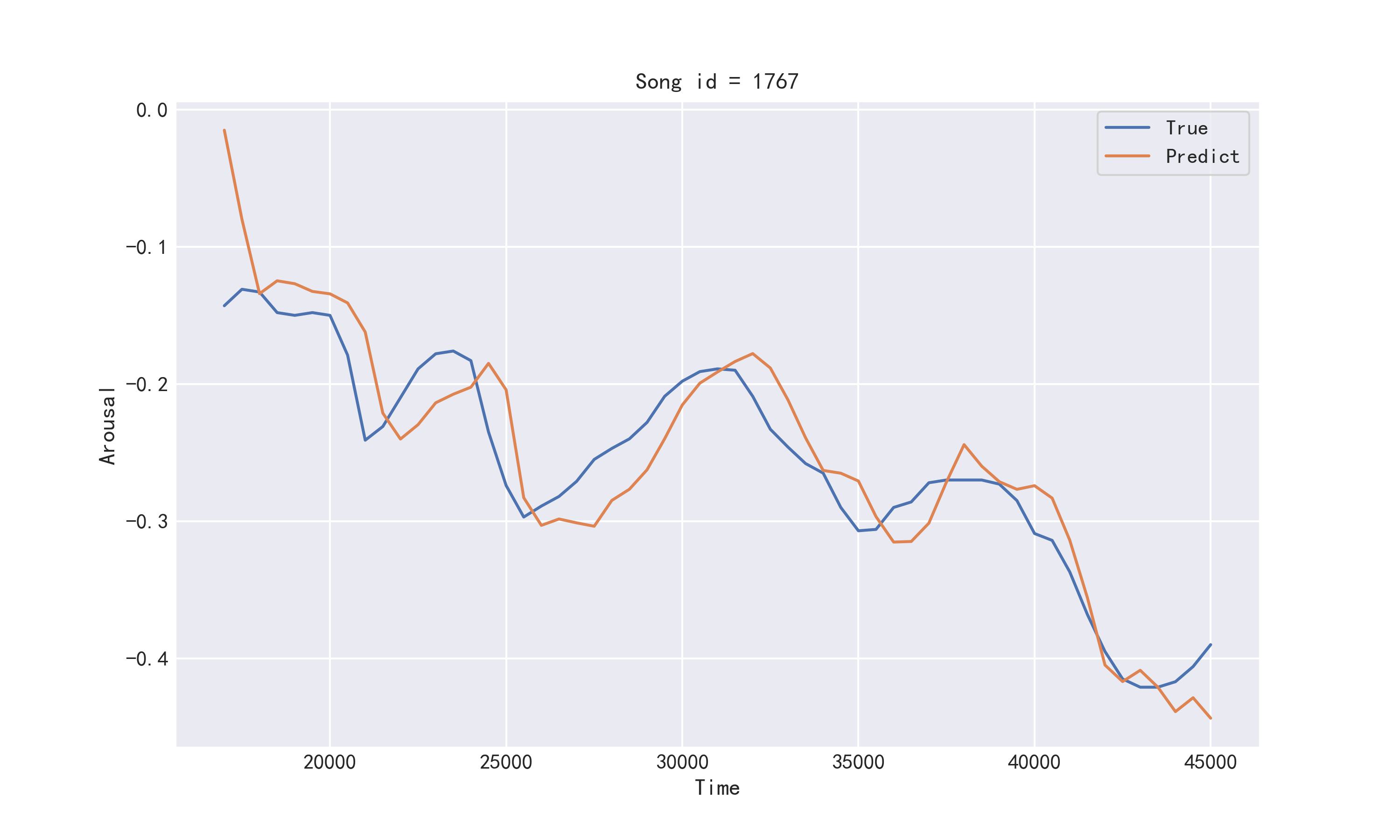

Supplement: S5 File — (ZIP) [file pone.0297712.s005.zip › All prediction results/prediction picture results(DEAM_100)/song_id_1767.jpg]

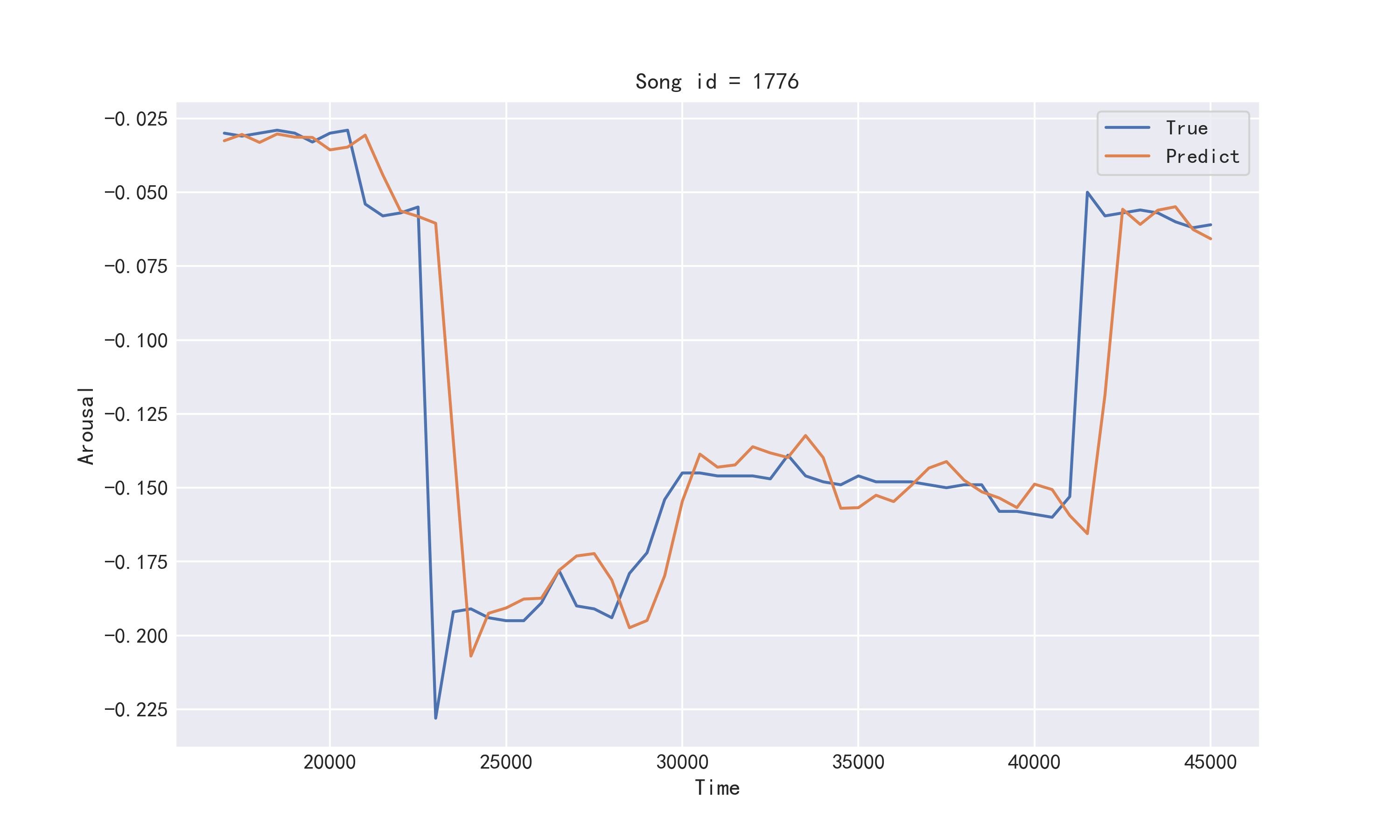

Supplement: S5 File — (ZIP) [file pone.0297712.s005.zip › All prediction results/prediction picture results(DEAM_100)/song_id_1776.jpg]

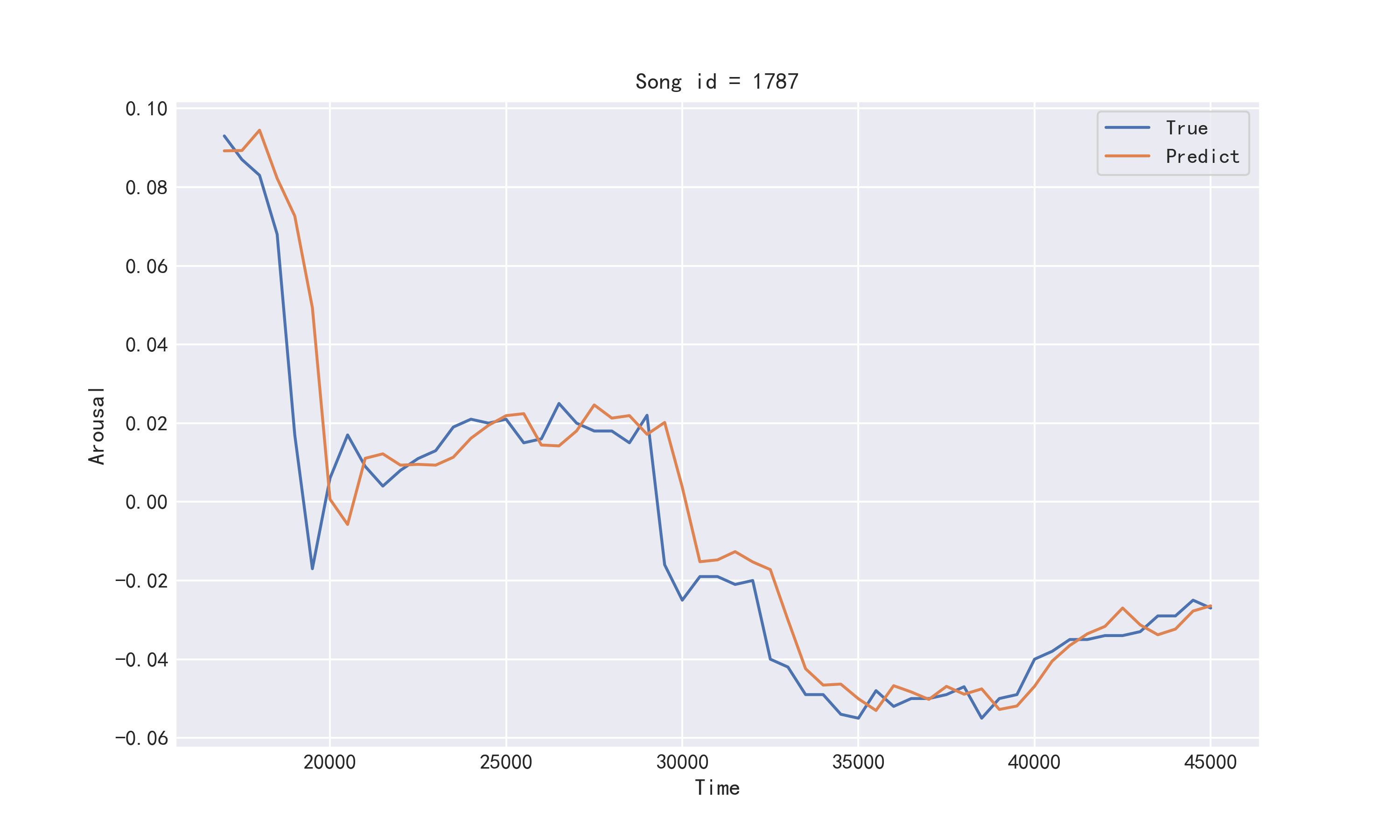

Supplement: S5 File — (ZIP) [file pone.0297712.s005.zip › All prediction results/prediction picture results(DEAM_100)/song_id_1787.jpg]

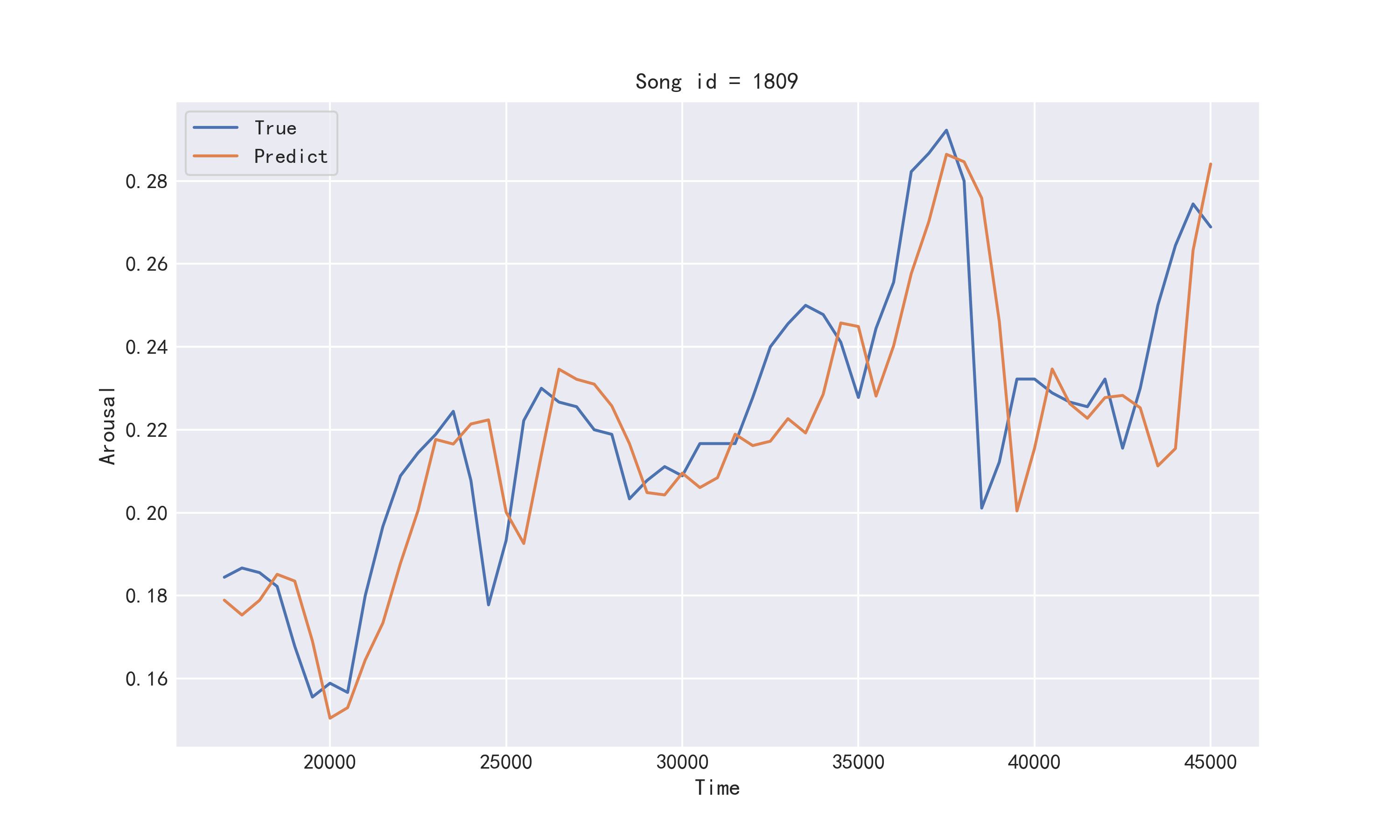

Supplement: S5 File — (ZIP) [file pone.0297712.s005.zip › All prediction results/prediction picture results(DEAM_100)/song_id_1809.jpg]

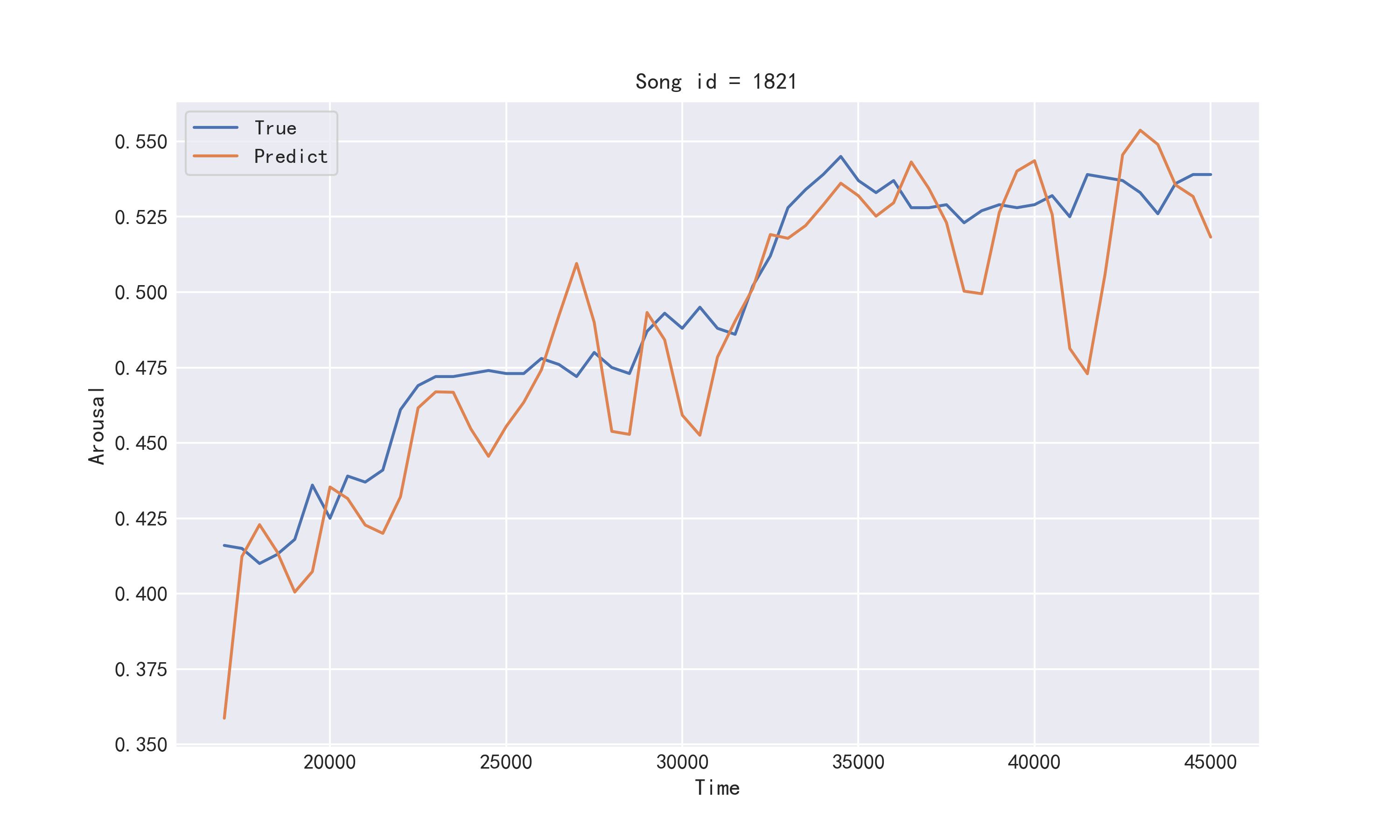

Supplement: S5 File — (ZIP) [file pone.0297712.s005.zip › All prediction results/prediction picture results(DEAM_100)/song_id_1821.jpg]

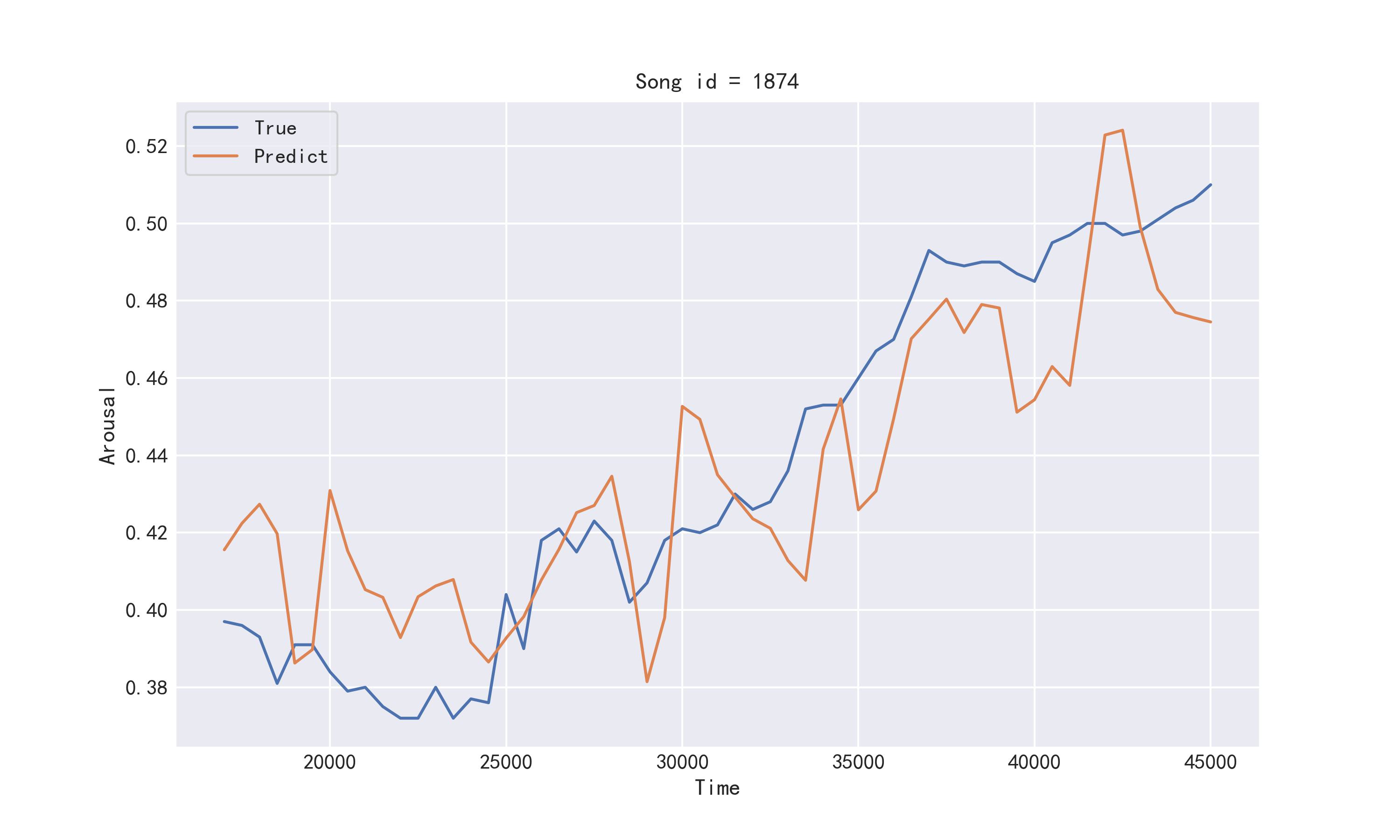

Supplement: S5 File — (ZIP) [file pone.0297712.s005.zip › All prediction results/prediction picture results(DEAM_100)/song_id_1874.jpg]

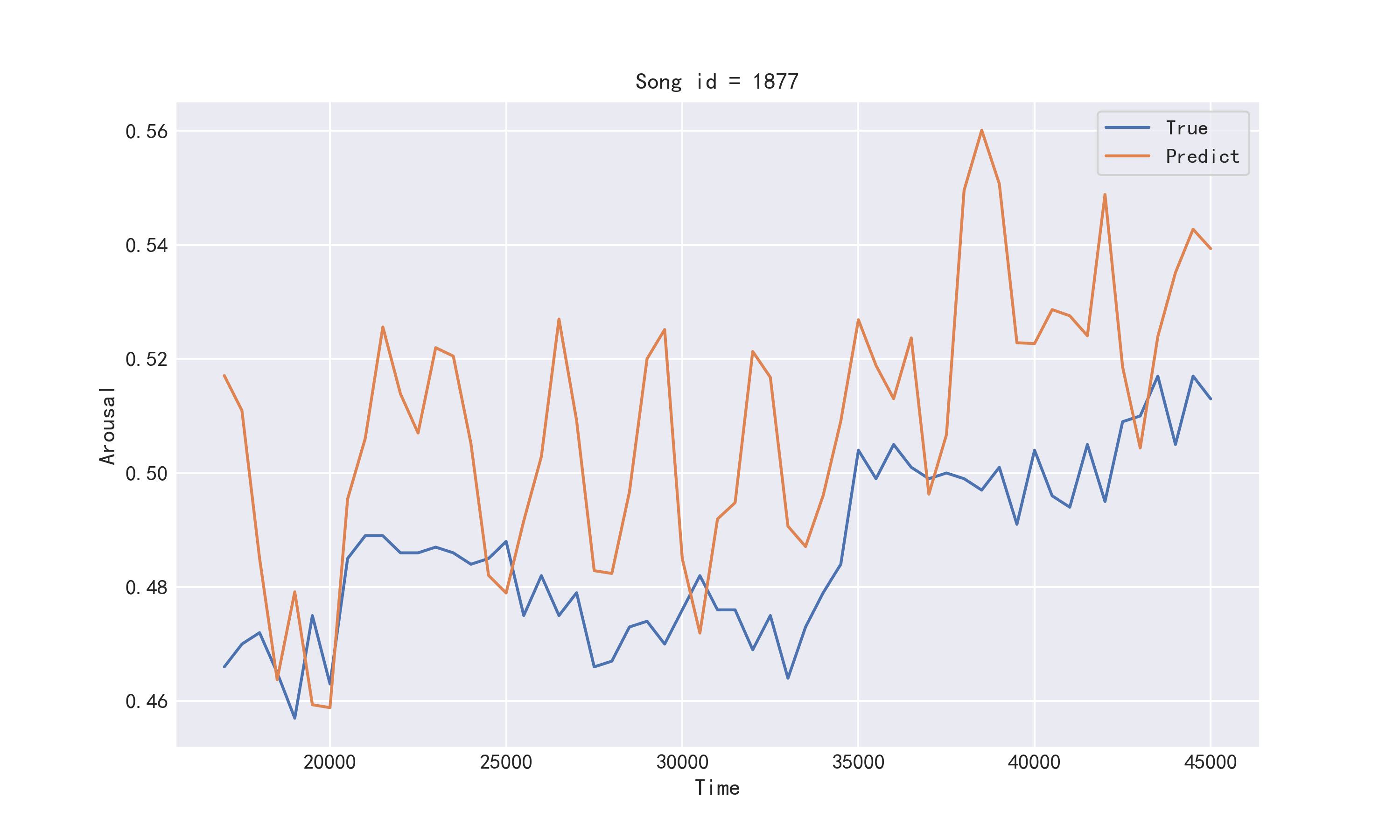

Supplement: S5 File — (ZIP) [file pone.0297712.s005.zip › All prediction results/prediction picture results(DEAM_100)/song_id_1877.jpg]

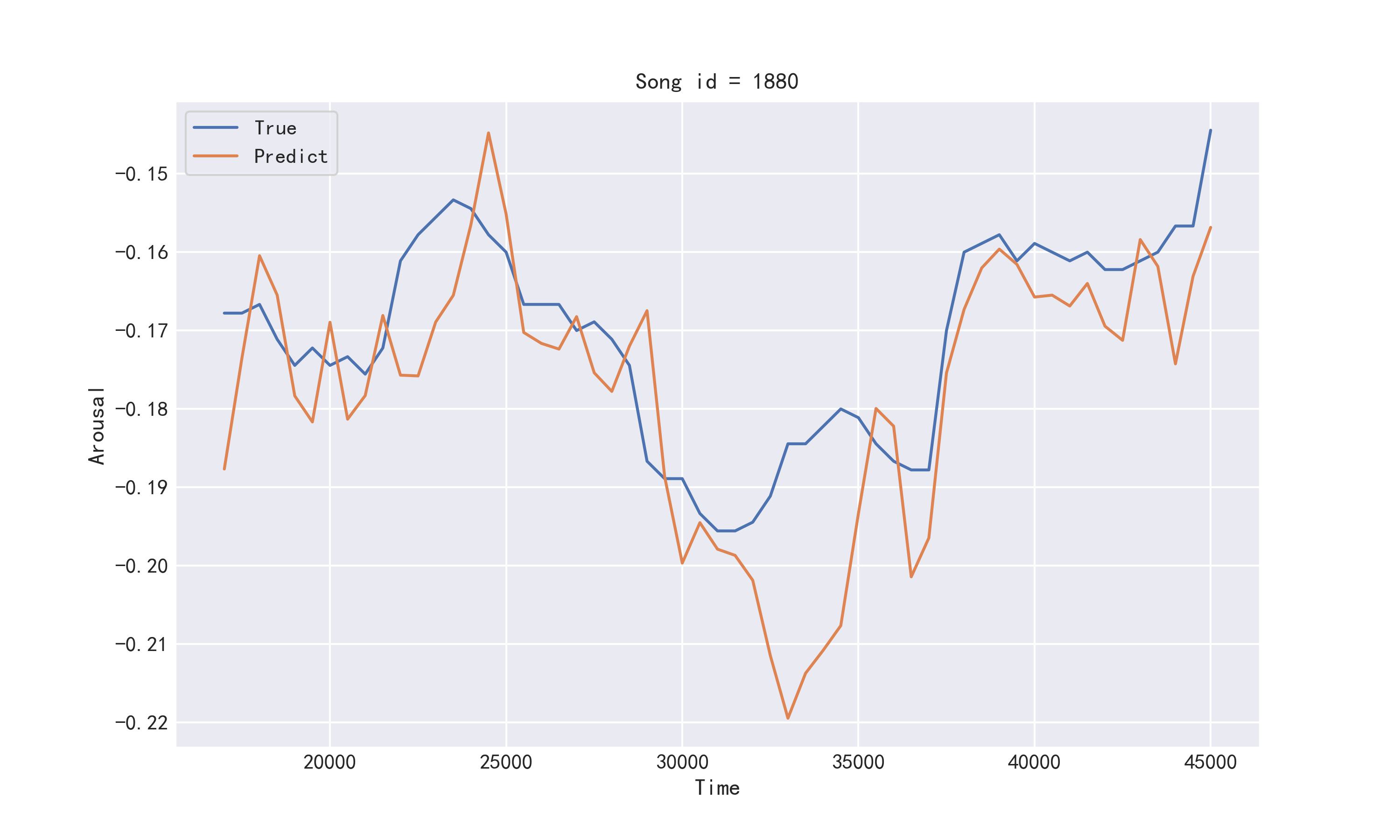

Supplement: S5 File — (ZIP) [file pone.0297712.s005.zip › All prediction results/prediction picture results(DEAM_100)/song_id_1880.jpg]

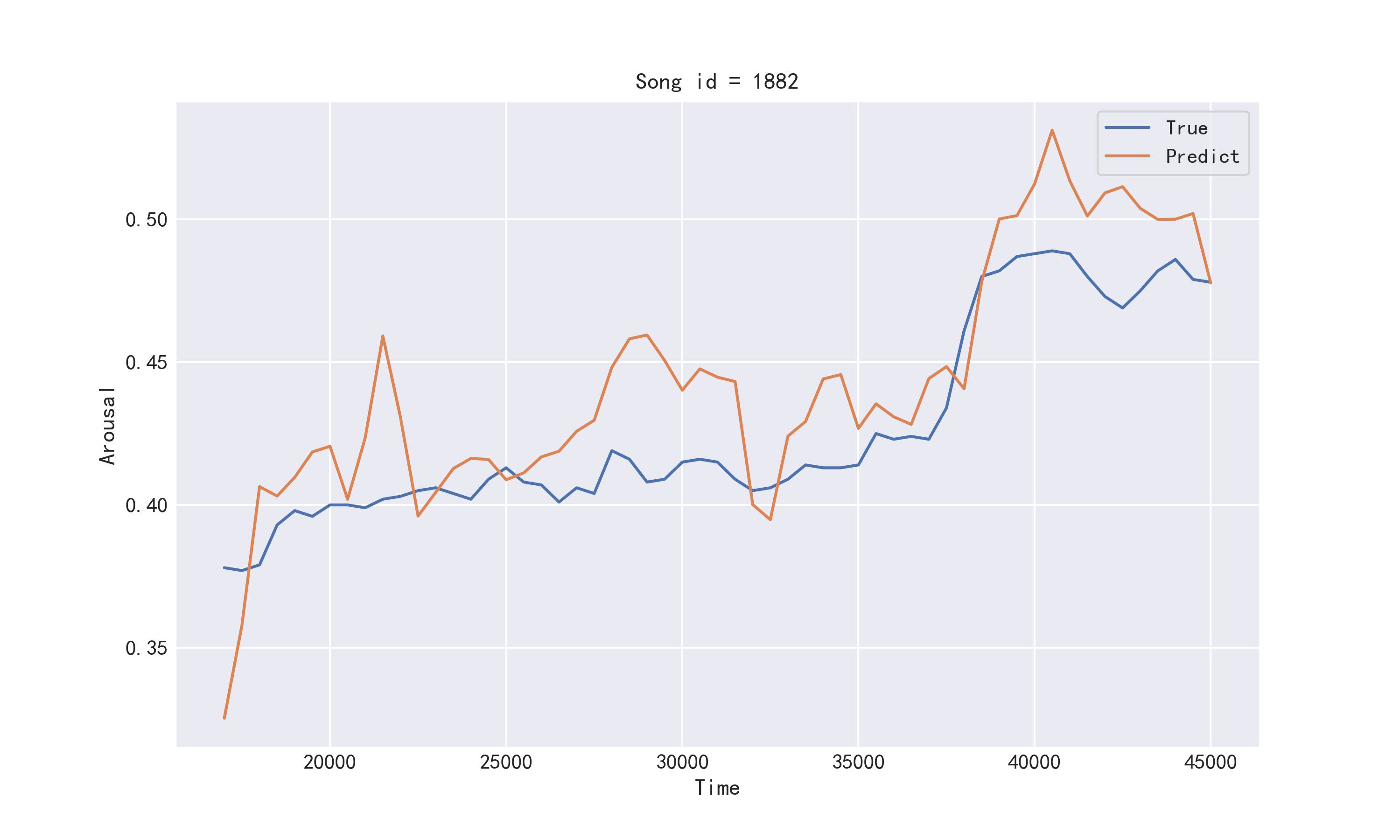

Supplement: S5 File — (ZIP) [file pone.0297712.s005.zip › All prediction results/prediction picture results(DEAM_100)/song_id_1882.jpg]

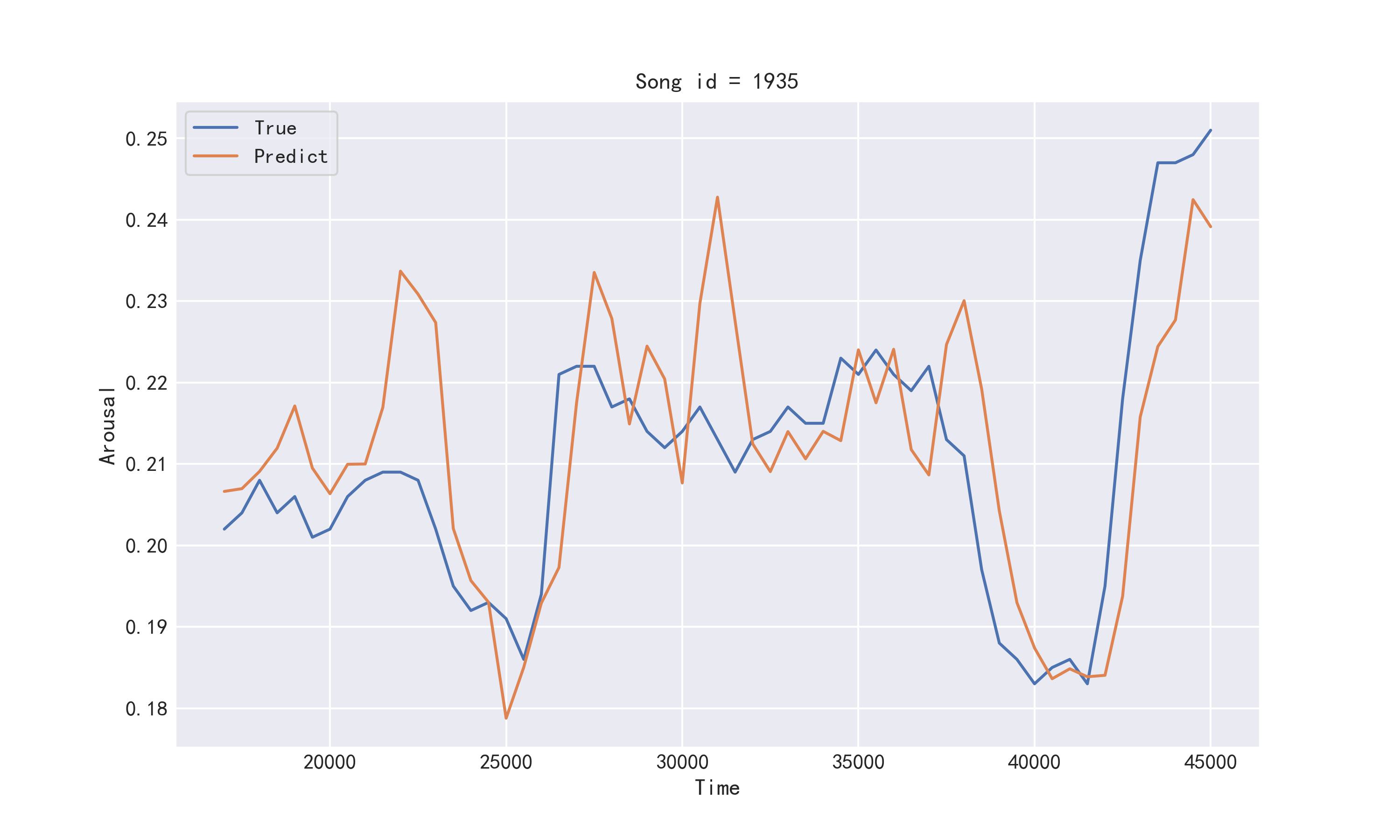

Supplement: S5 File — (ZIP) [file pone.0297712.s005.zip › All prediction results/prediction picture results(DEAM_100)/song_id_1935.jpg]

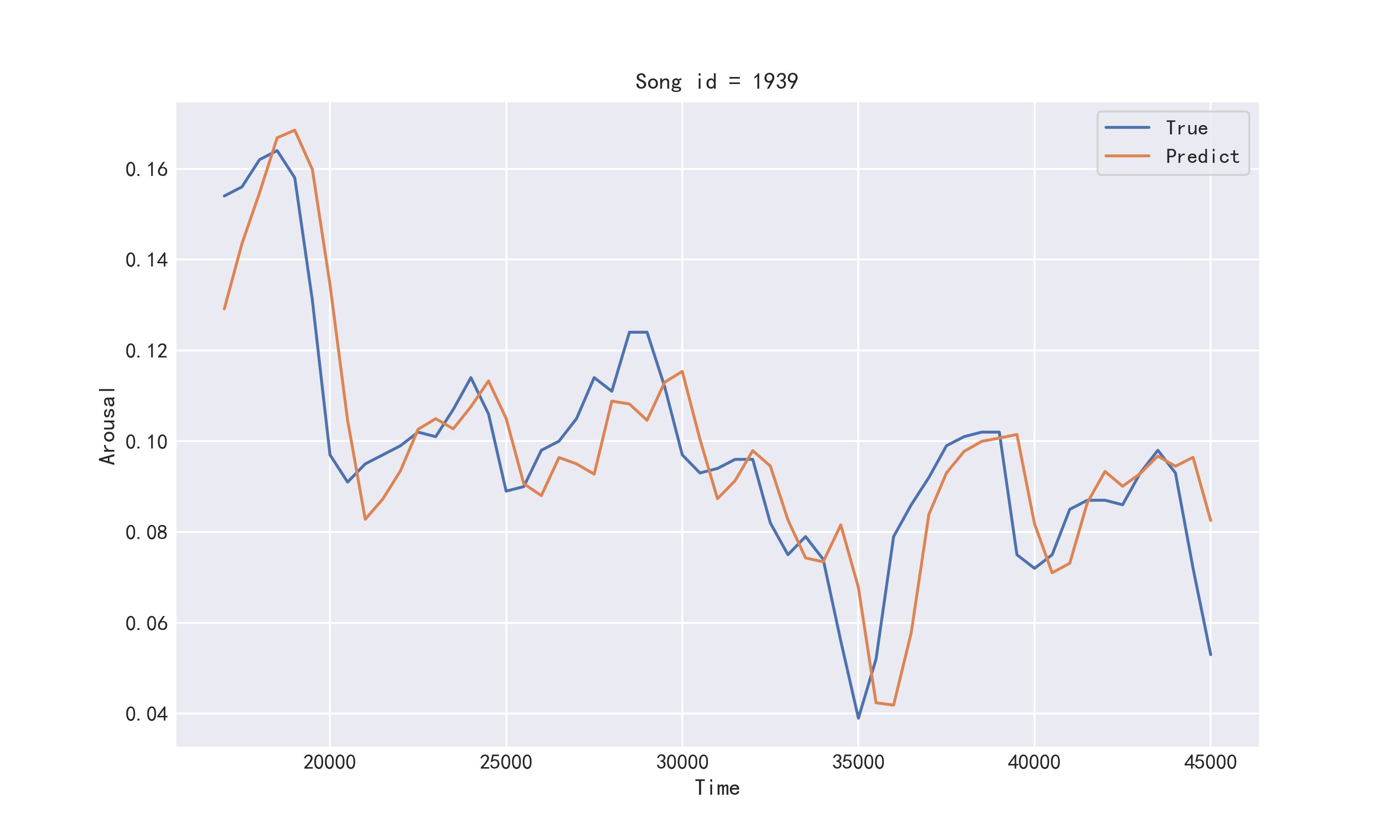

Supplement: S5 File — (ZIP) [file pone.0297712.s005.zip › All prediction results/prediction picture results(DEAM_100)/song_id_1939.jpg]

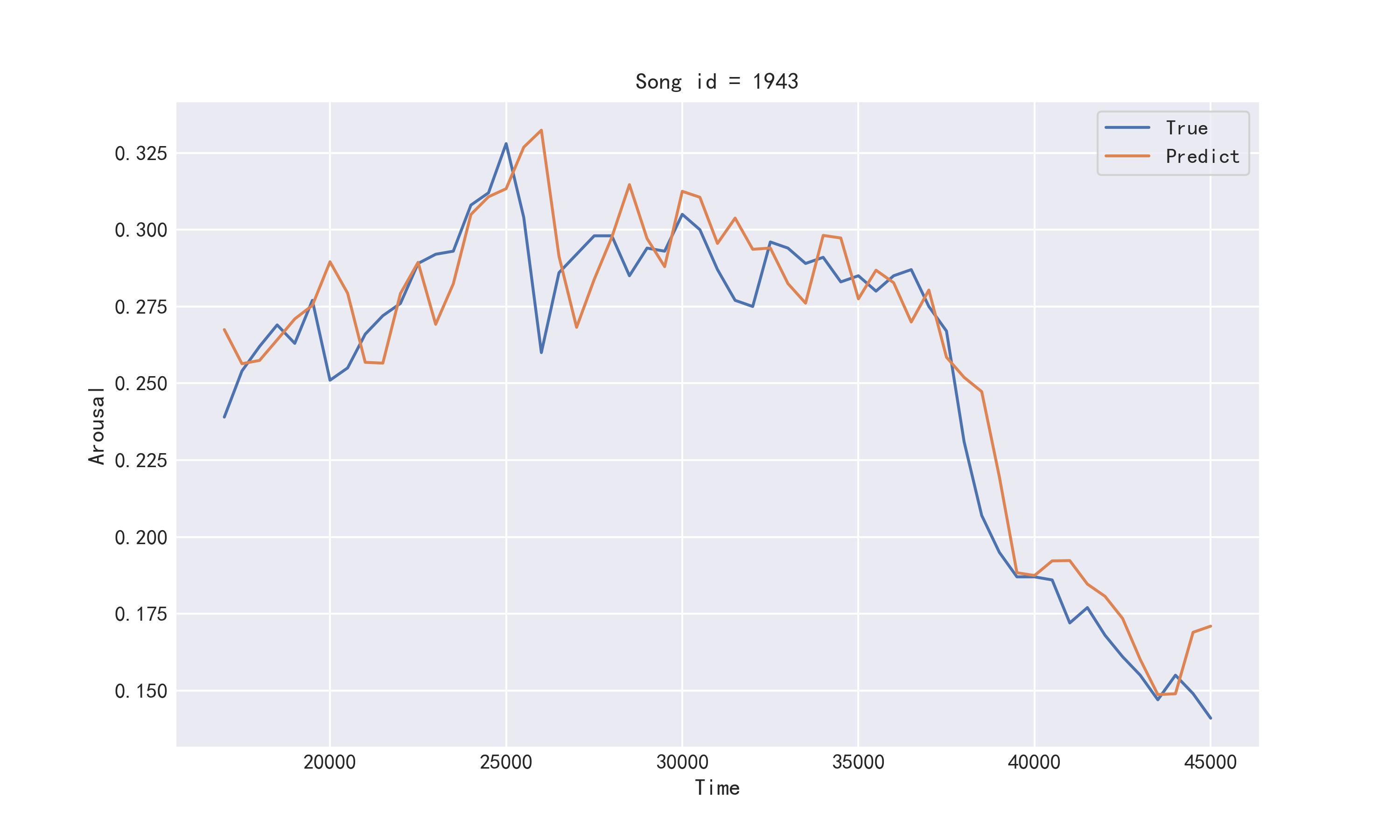

Supplement: S5 File — (ZIP) [file pone.0297712.s005.zip › All prediction results/prediction picture results(DEAM_100)/song_id_1943.jpg]

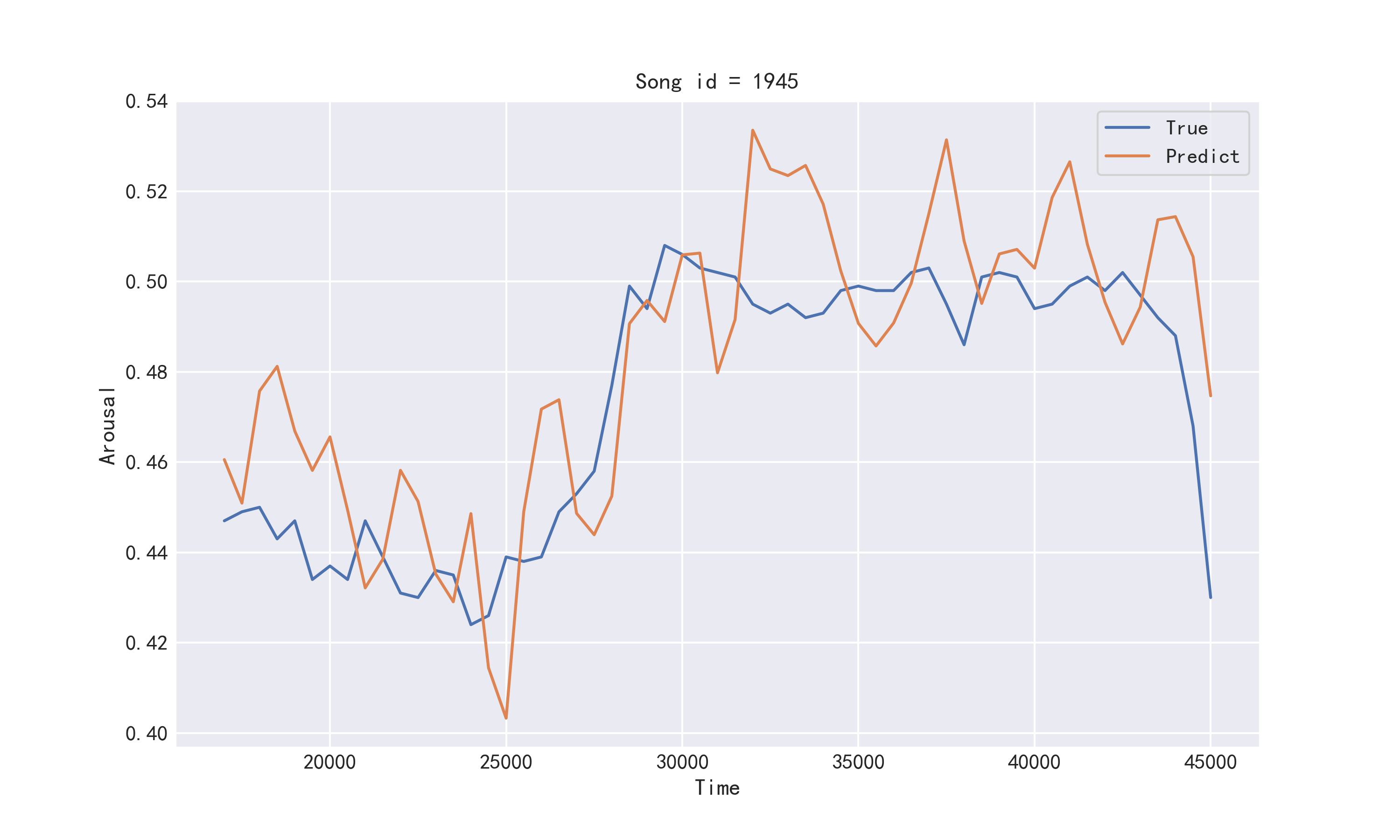

Supplement: S5 File — (ZIP) [file pone.0297712.s005.zip › All prediction results/prediction picture results(DEAM_100)/song_id_1945.jpg]

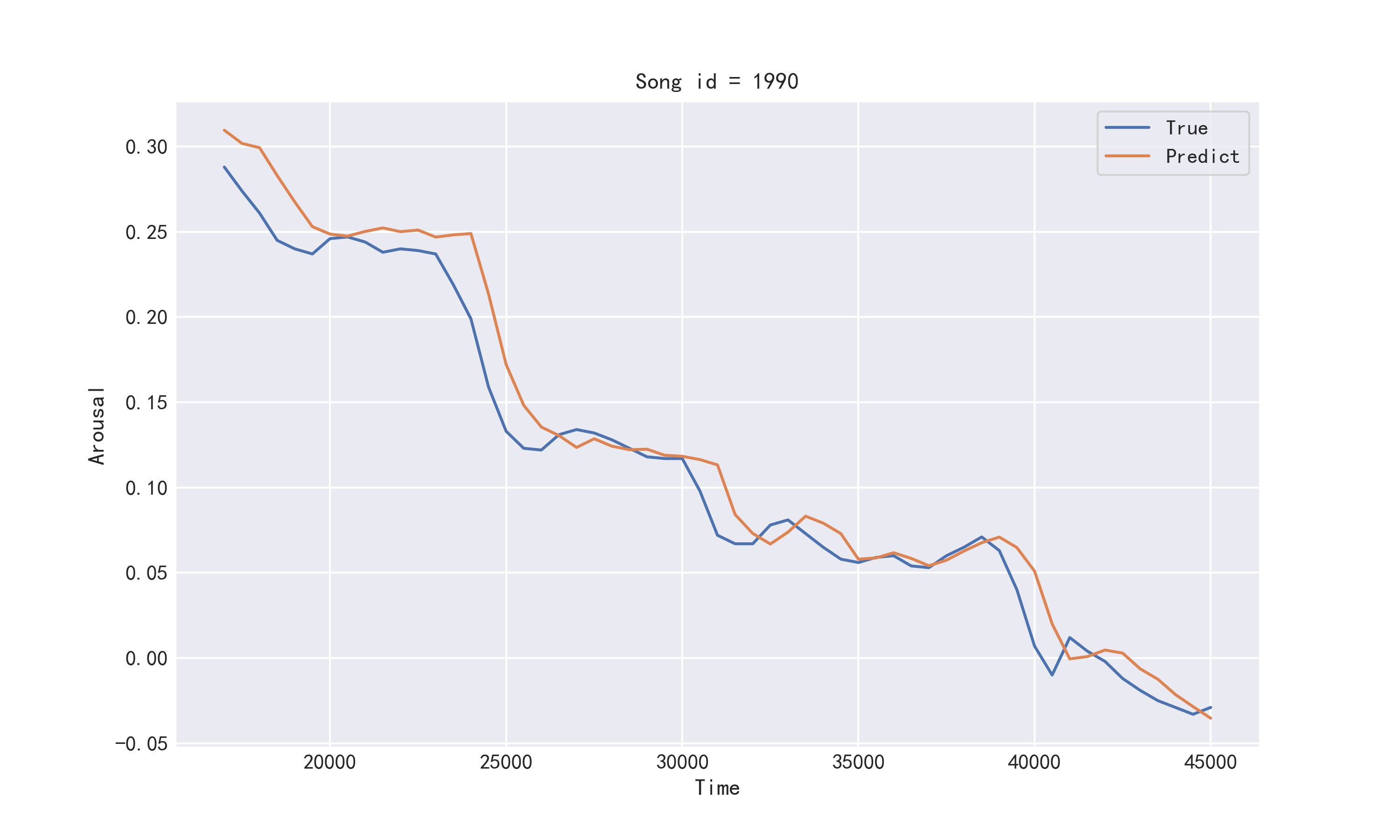

Supplement: S5 File — (ZIP) [file pone.0297712.s005.zip › All prediction results/prediction picture results(DEAM_100)/song_id_1990.jpg]

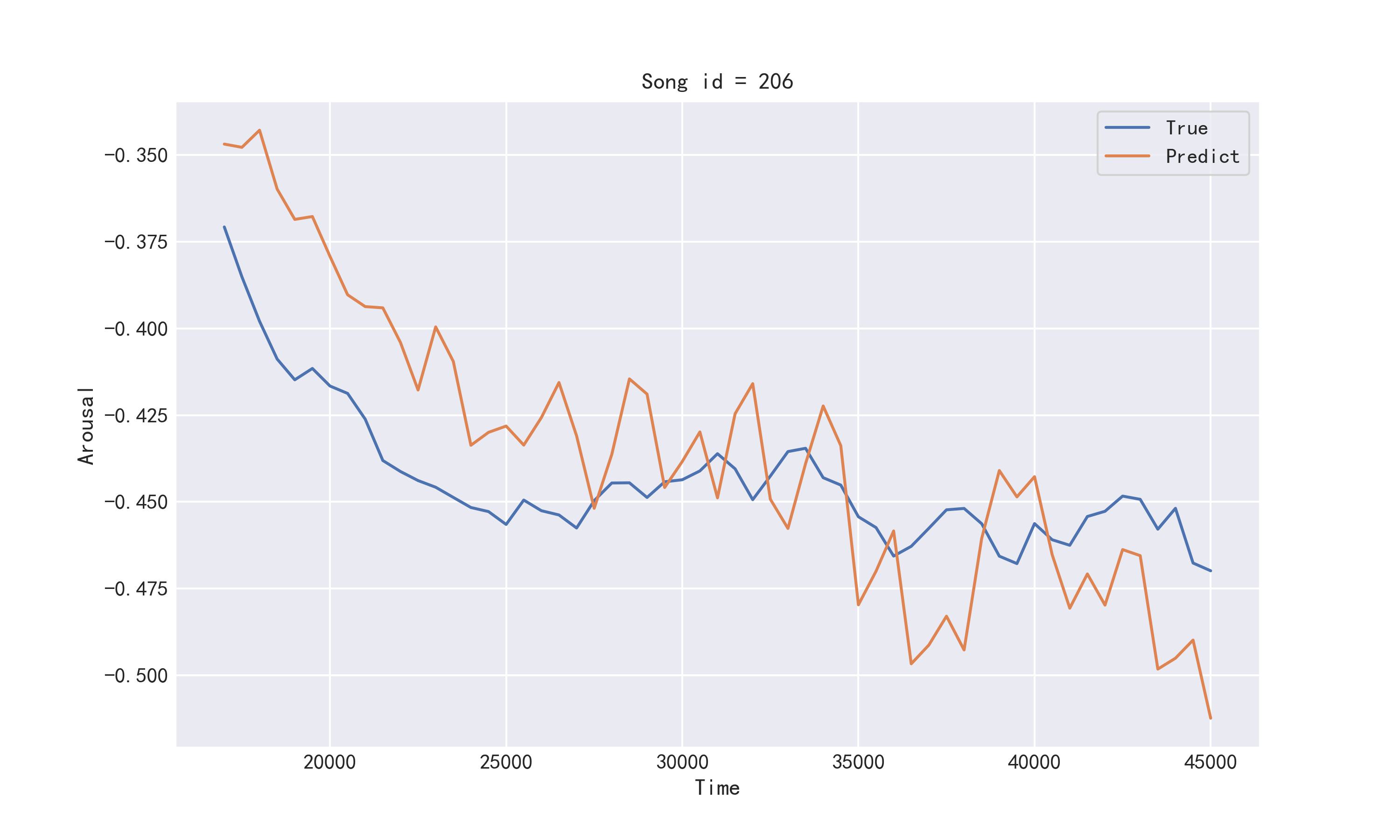

Supplement: S5 File — (ZIP) [file pone.0297712.s005.zip › All prediction results/prediction picture results(DEAM_100)/song_id_206.jpg]

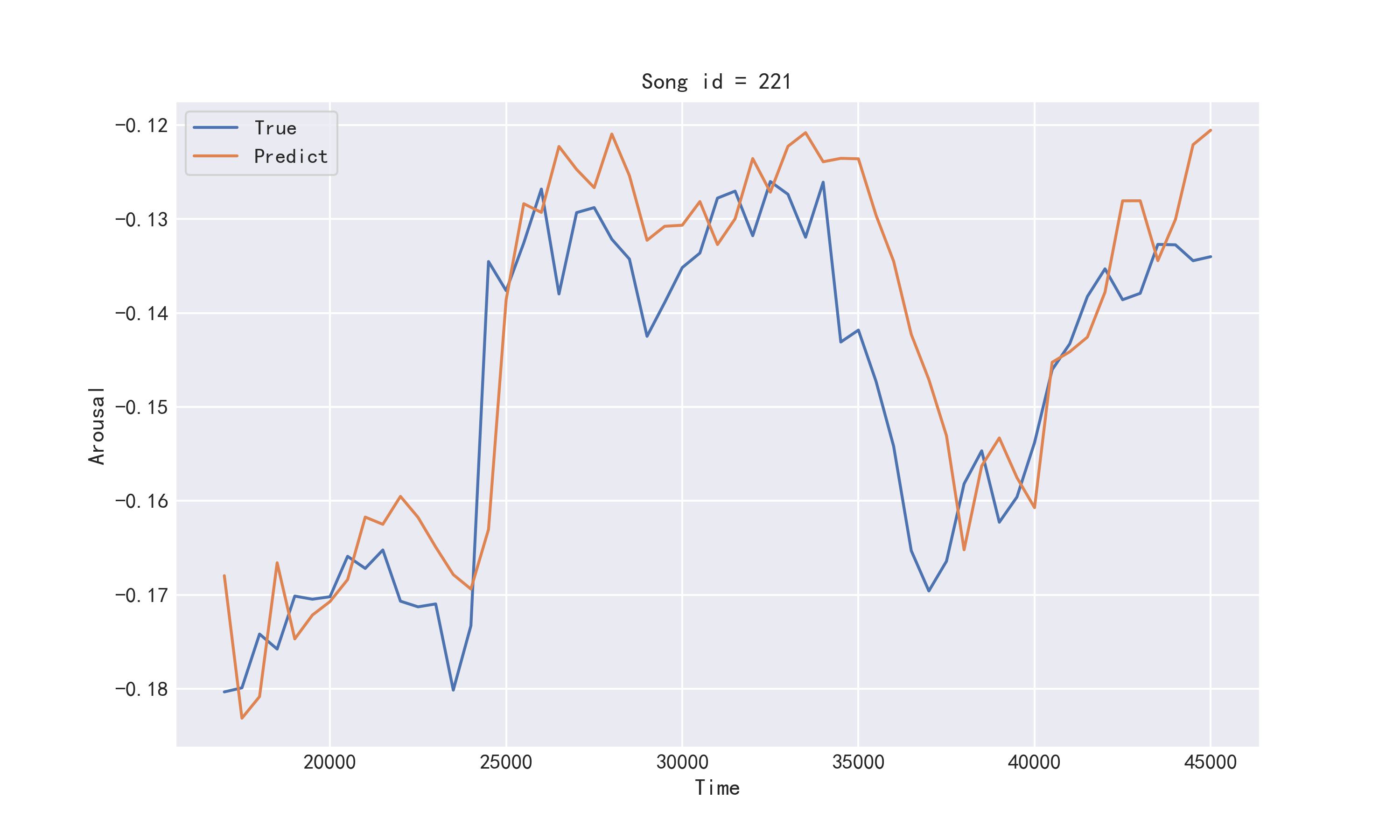

Supplement: S5 File — (ZIP) [file pone.0297712.s005.zip › All prediction results/prediction picture results(DEAM_100)/song_id_221.jpg]

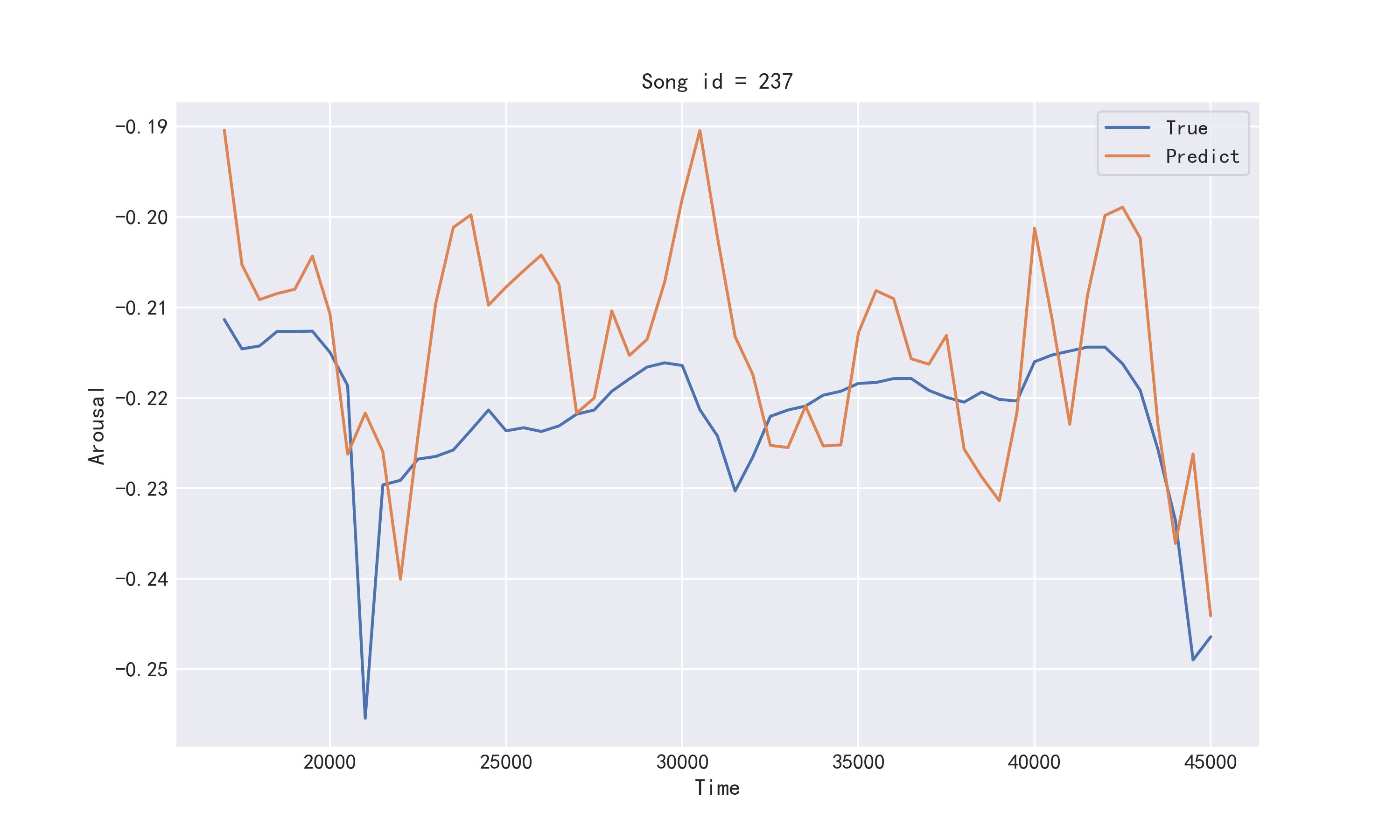

Supplement: S5 File — (ZIP) [file pone.0297712.s005.zip › All prediction results/prediction picture results(DEAM_100)/song_id_237.jpg]

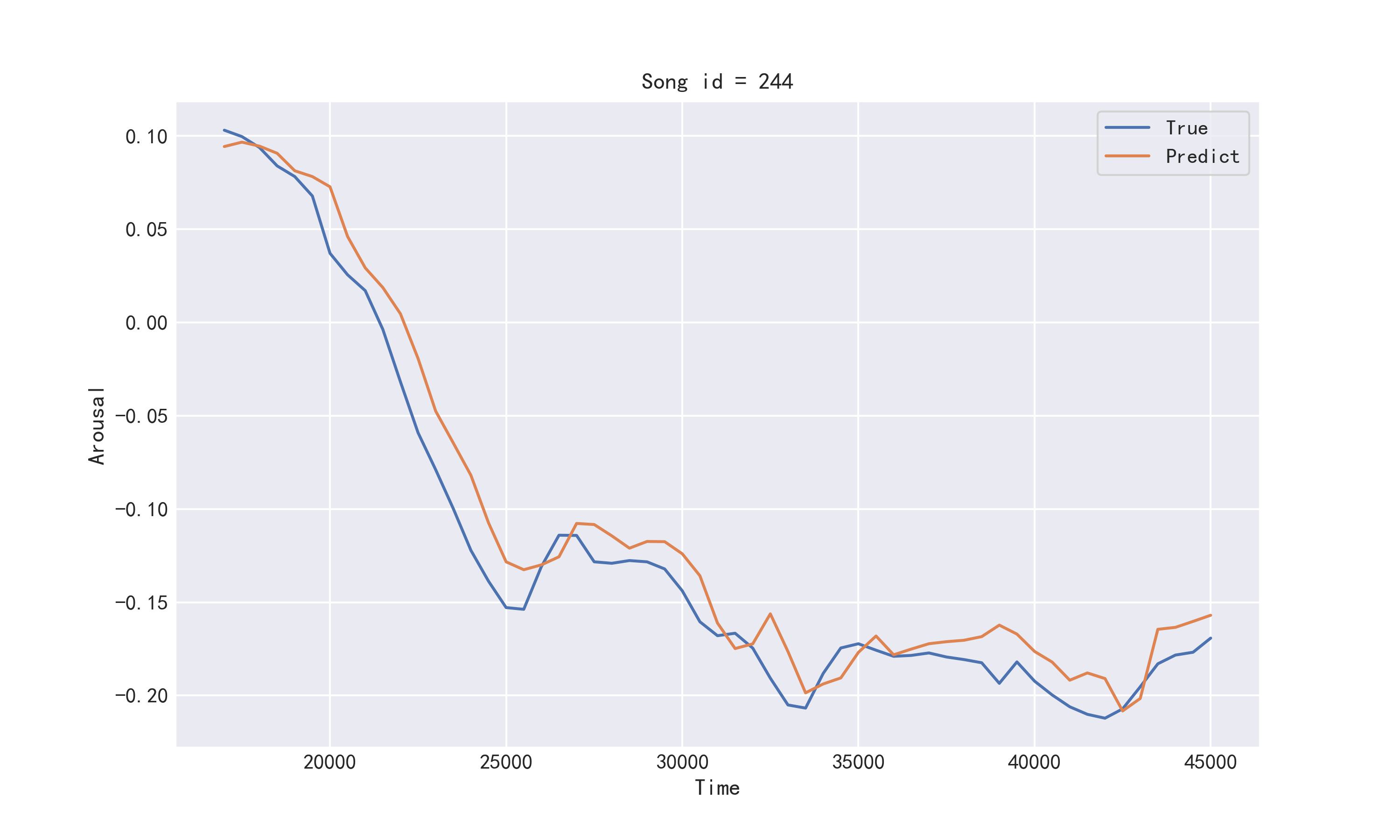

Supplement: S5 File — (ZIP) [file pone.0297712.s005.zip › All prediction results/prediction picture results(DEAM_100)/song_id_244.jpg]

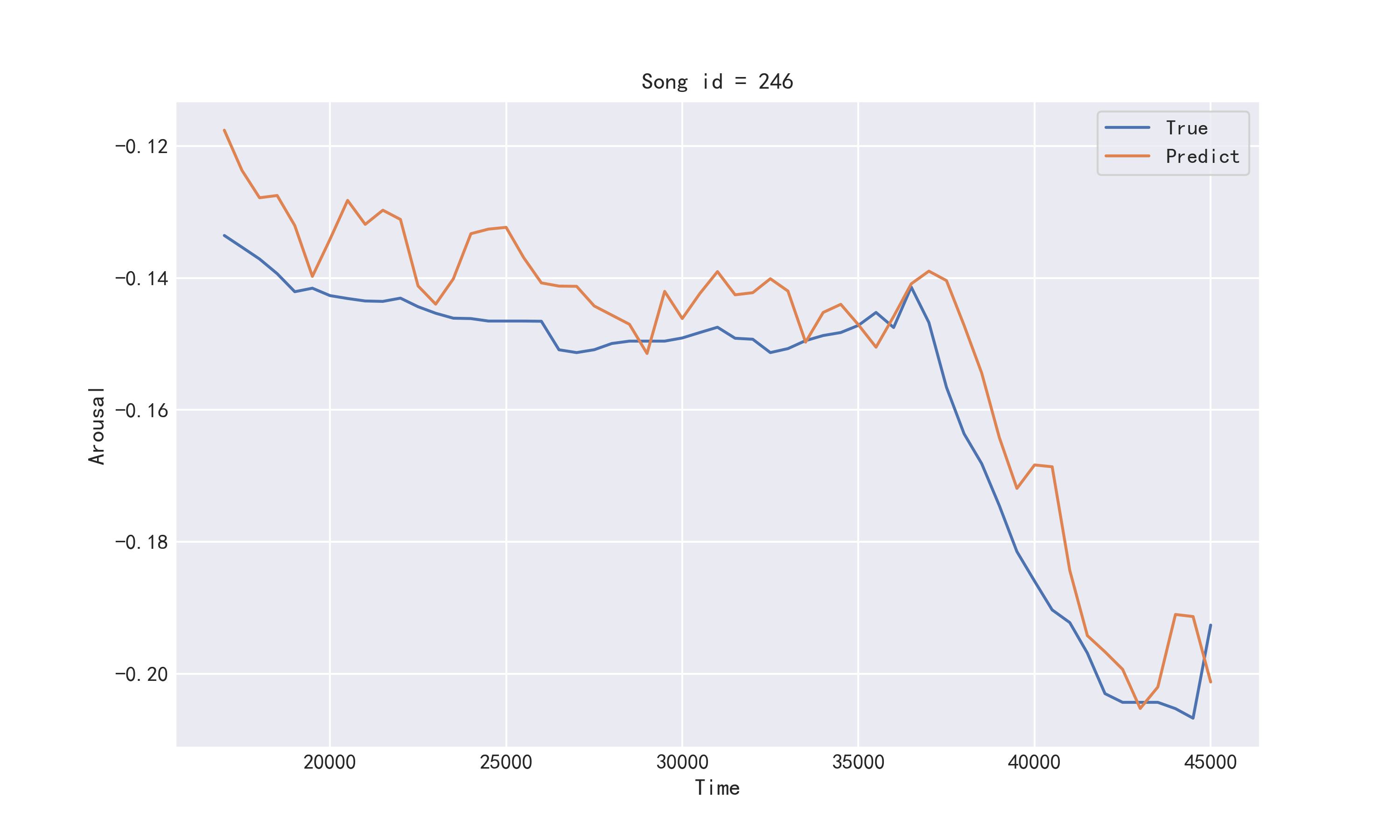

Supplement: S5 File — (ZIP) [file pone.0297712.s005.zip › All prediction results/prediction picture results(DEAM_100)/song_id_246.jpg]

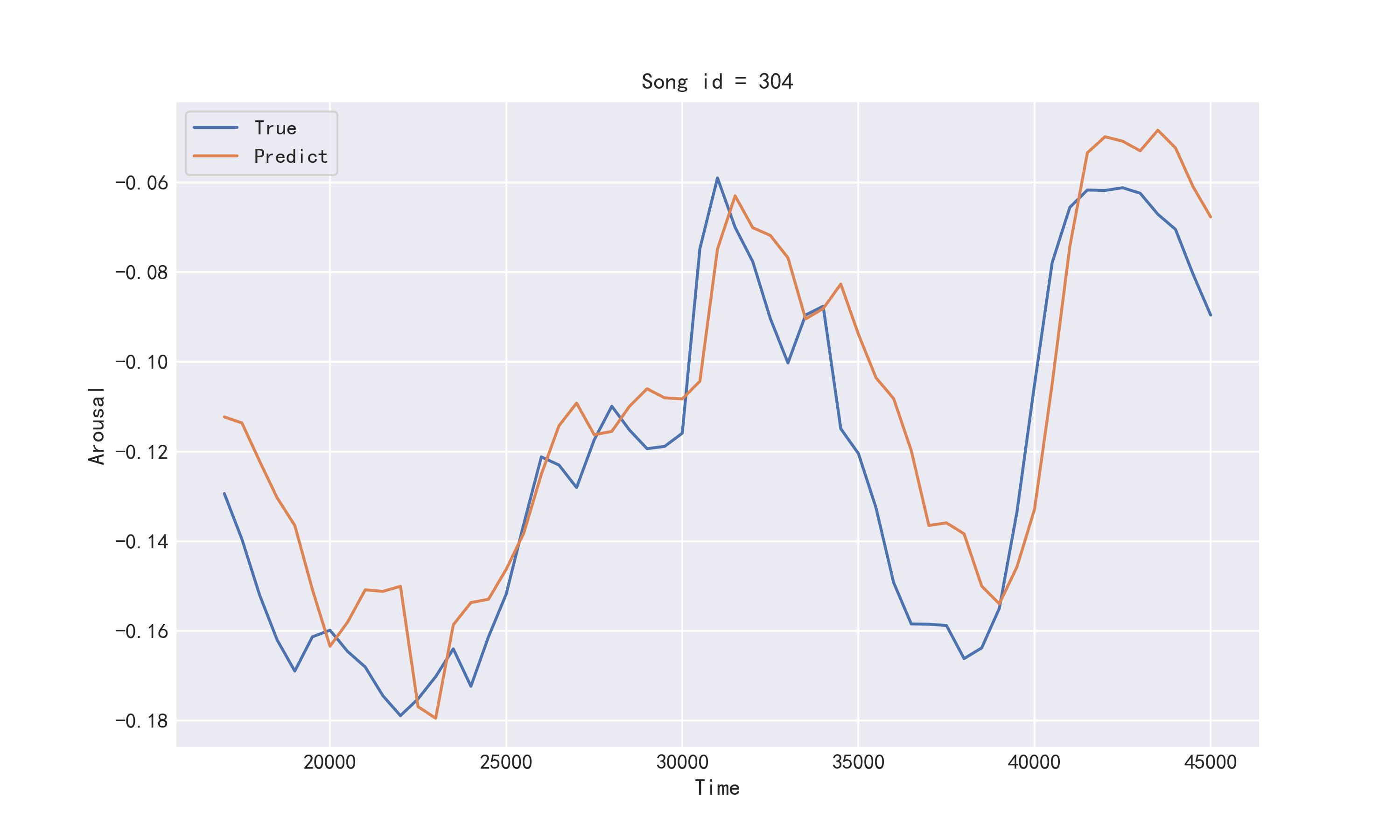

Supplement: S5 File — (ZIP) [file pone.0297712.s005.zip › All prediction results/prediction picture results(DEAM_100)/song_id_304.jpg]

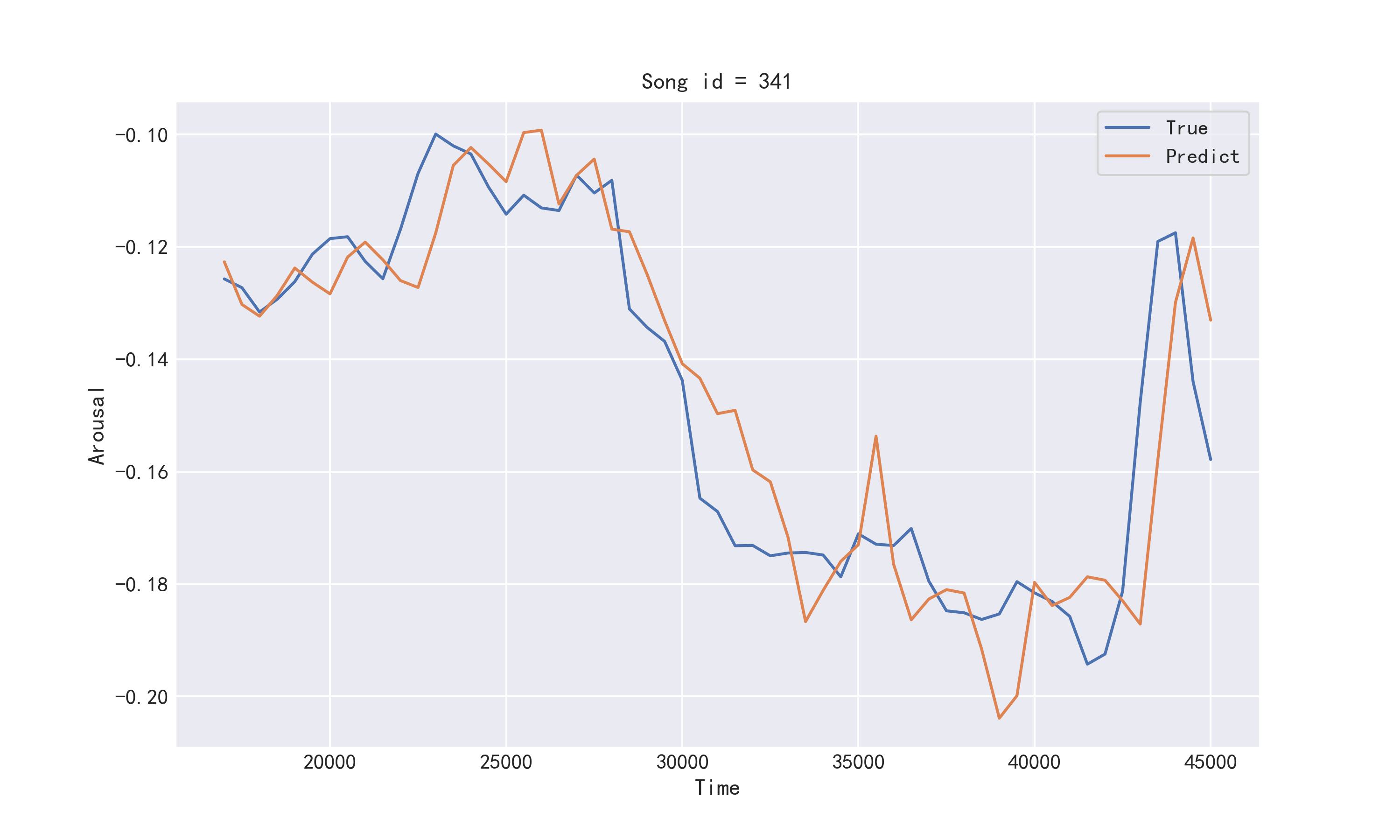

Supplement: S5 File — (ZIP) [file pone.0297712.s005.zip › All prediction results/prediction picture results(DEAM_100)/song_id_341.jpg]

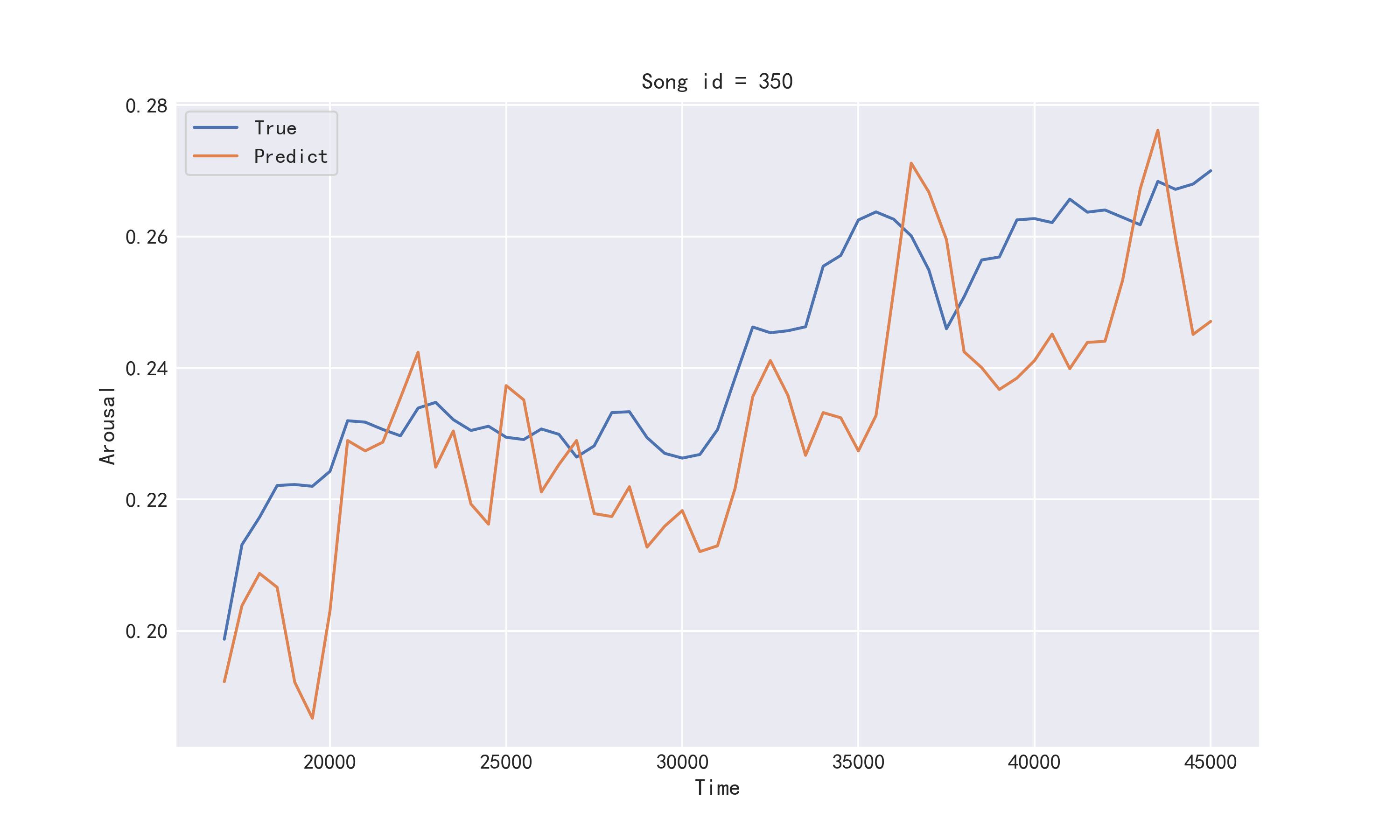

Supplement: S5 File — (ZIP) [file pone.0297712.s005.zip › All prediction results/prediction picture results(DEAM_100)/song_id_350.jpg]

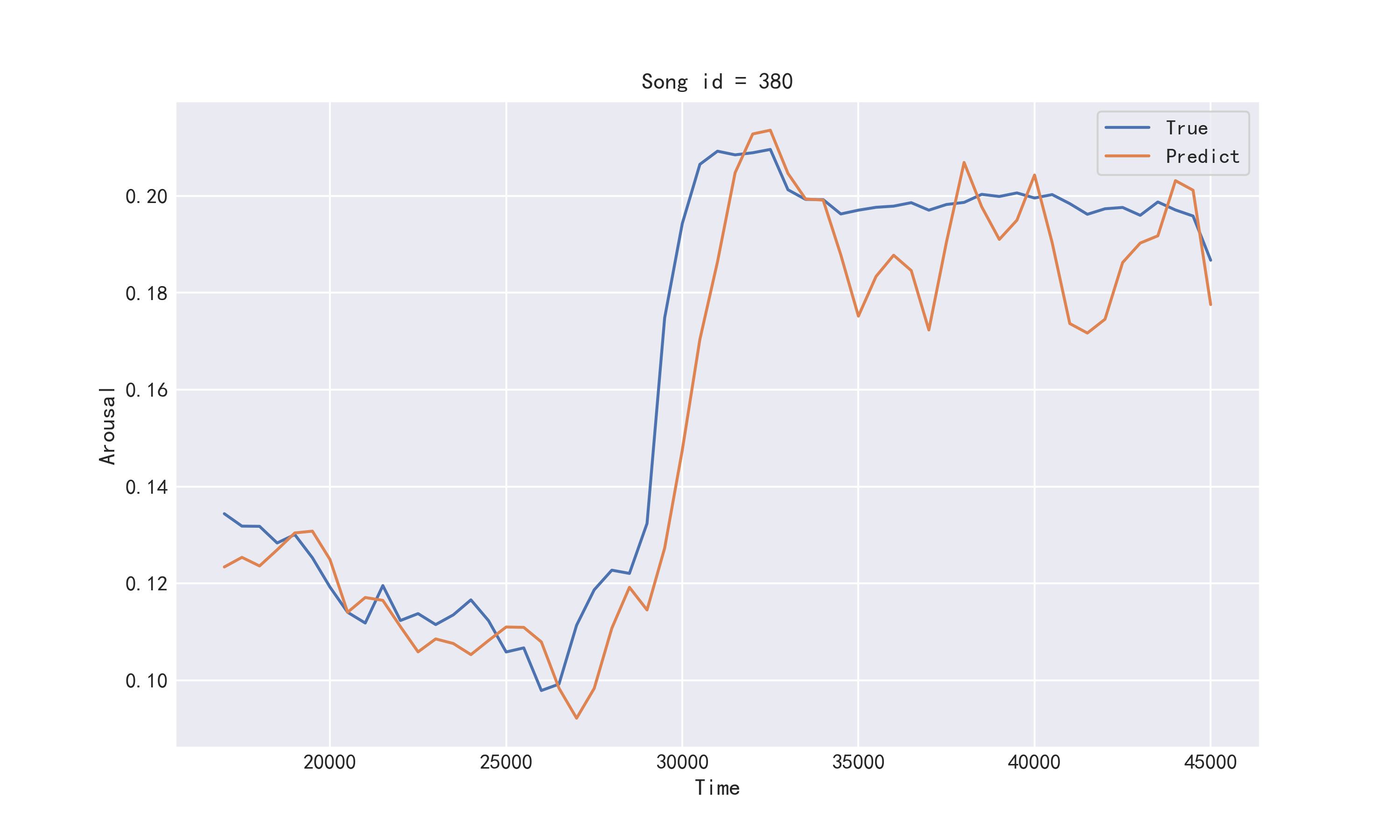

Supplement: S5 File — (ZIP) [file pone.0297712.s005.zip › All prediction results/prediction picture results(DEAM_100)/song_id_380.jpg]

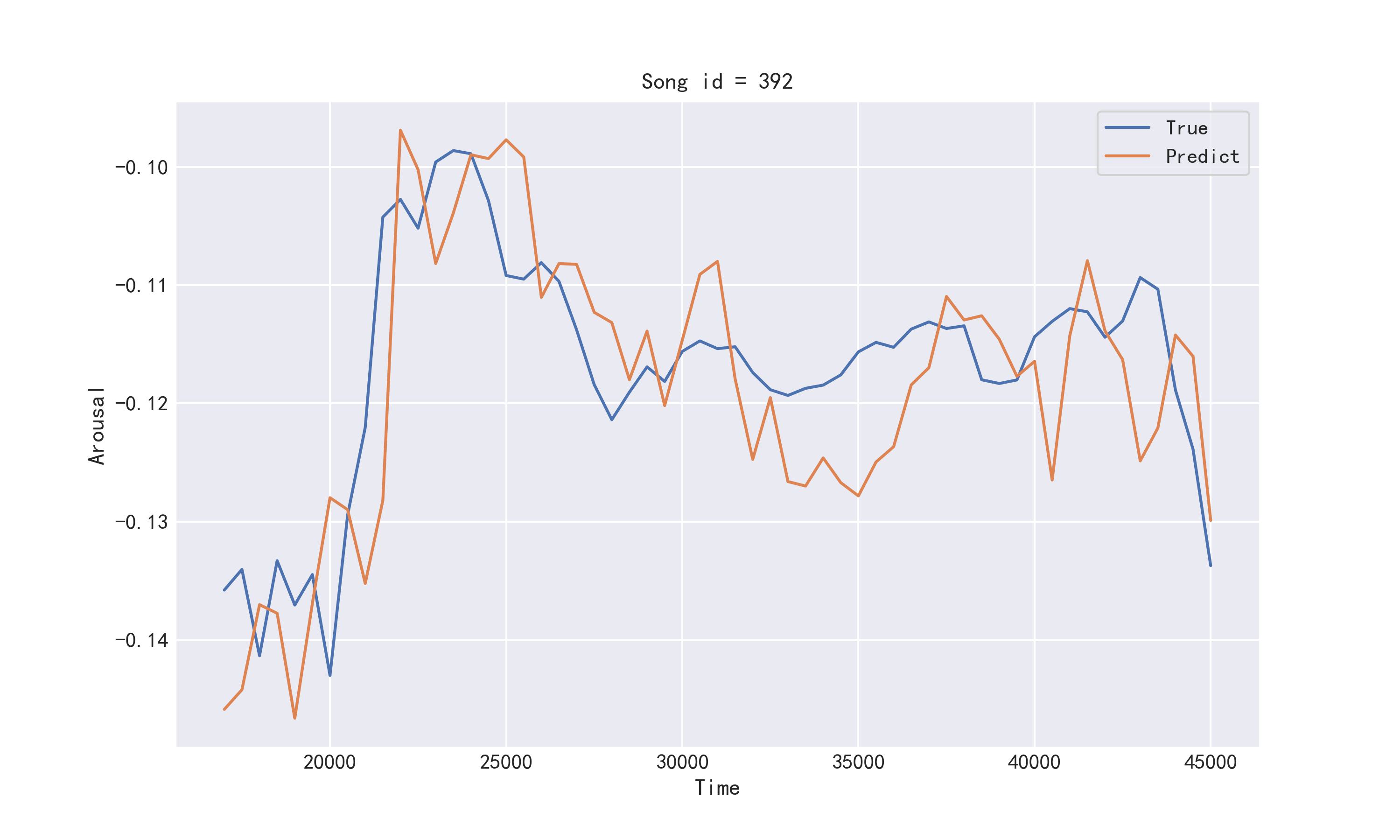

Supplement: S5 File — (ZIP) [file pone.0297712.s005.zip › All prediction results/prediction picture results(DEAM_100)/song_id_392.jpg]

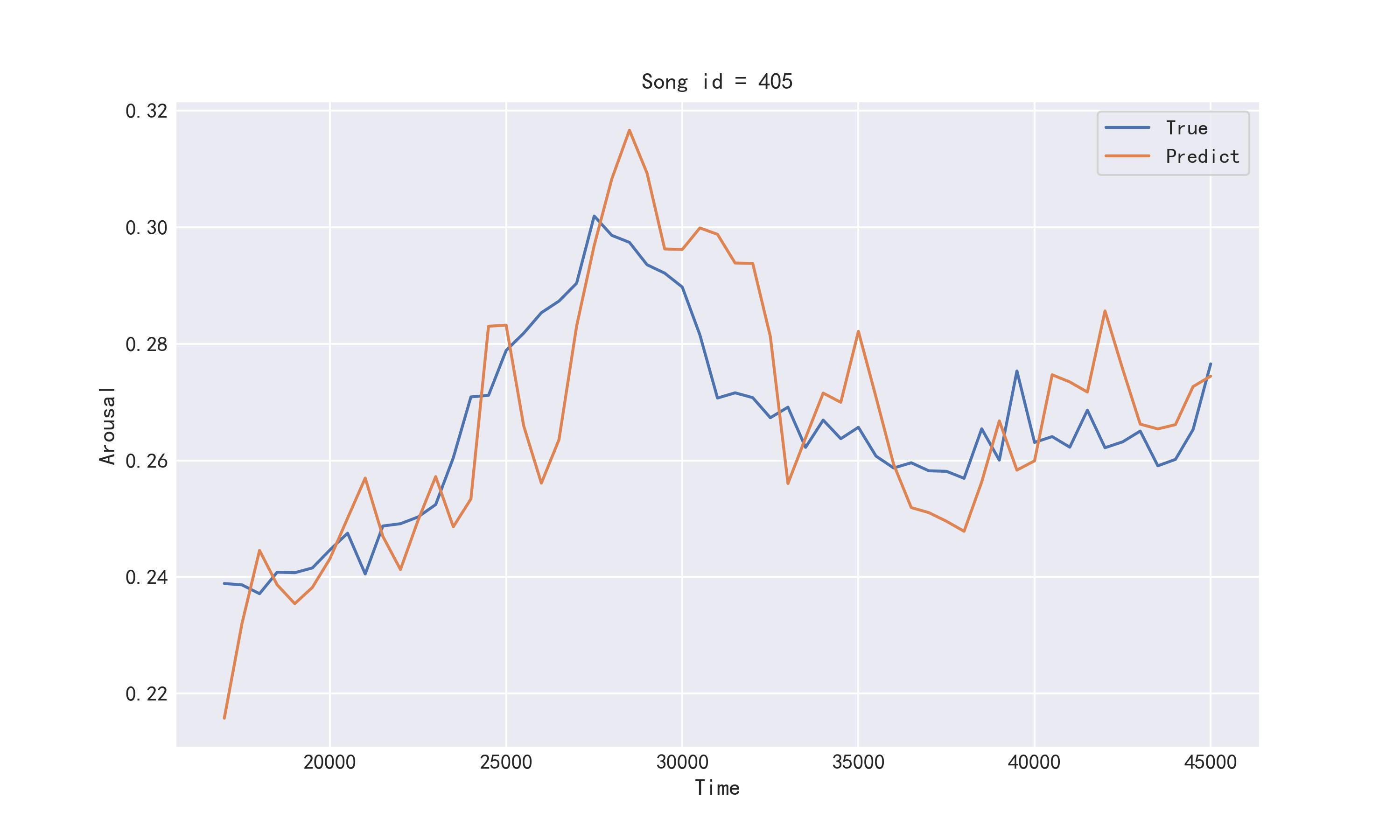

Supplement: S5 File — (ZIP) [file pone.0297712.s005.zip › All prediction results/prediction picture results(DEAM_100)/song_id_405.jpg]

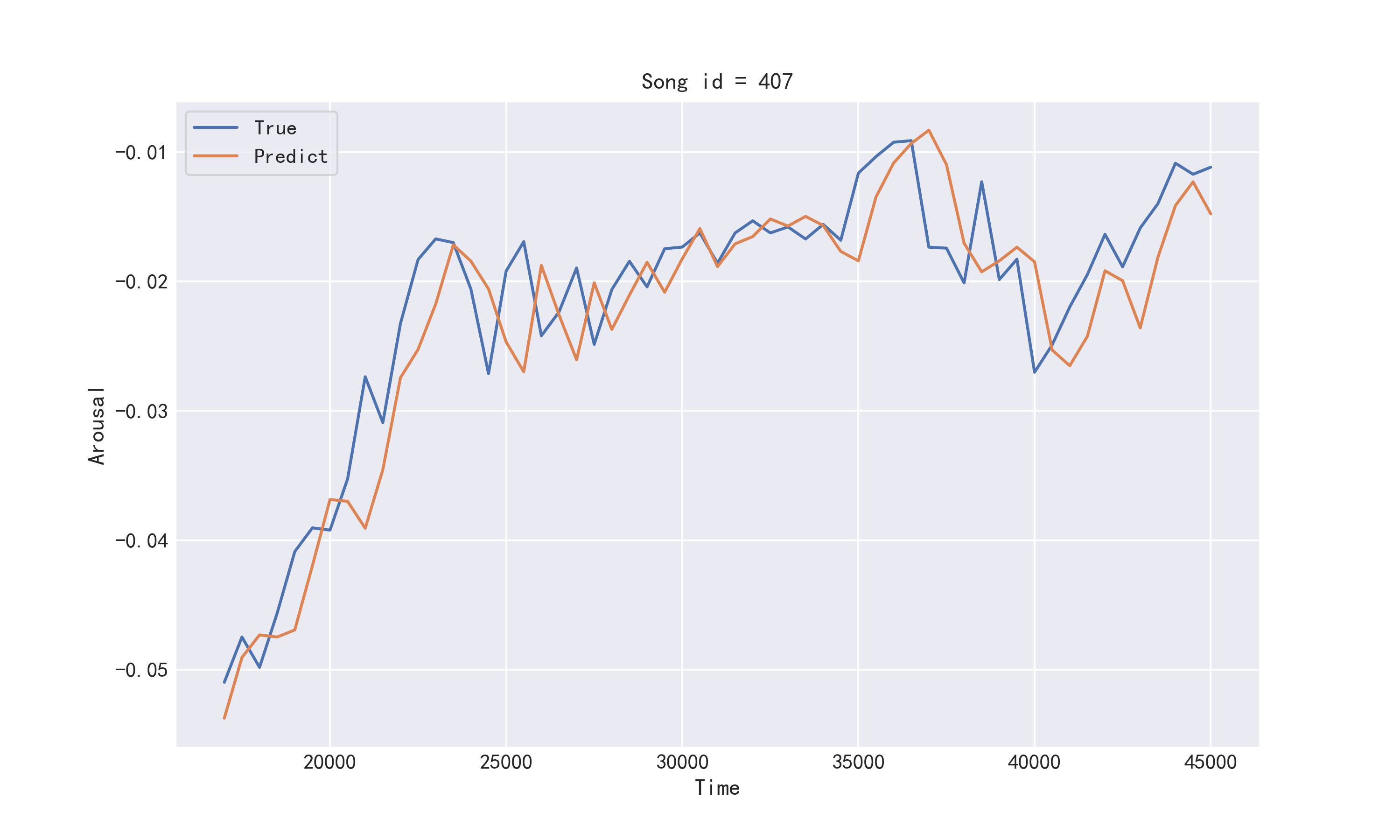

Supplement: S5 File — (ZIP) [file pone.0297712.s005.zip › All prediction results/prediction picture results(DEAM_100)/song_id_407.jpg]

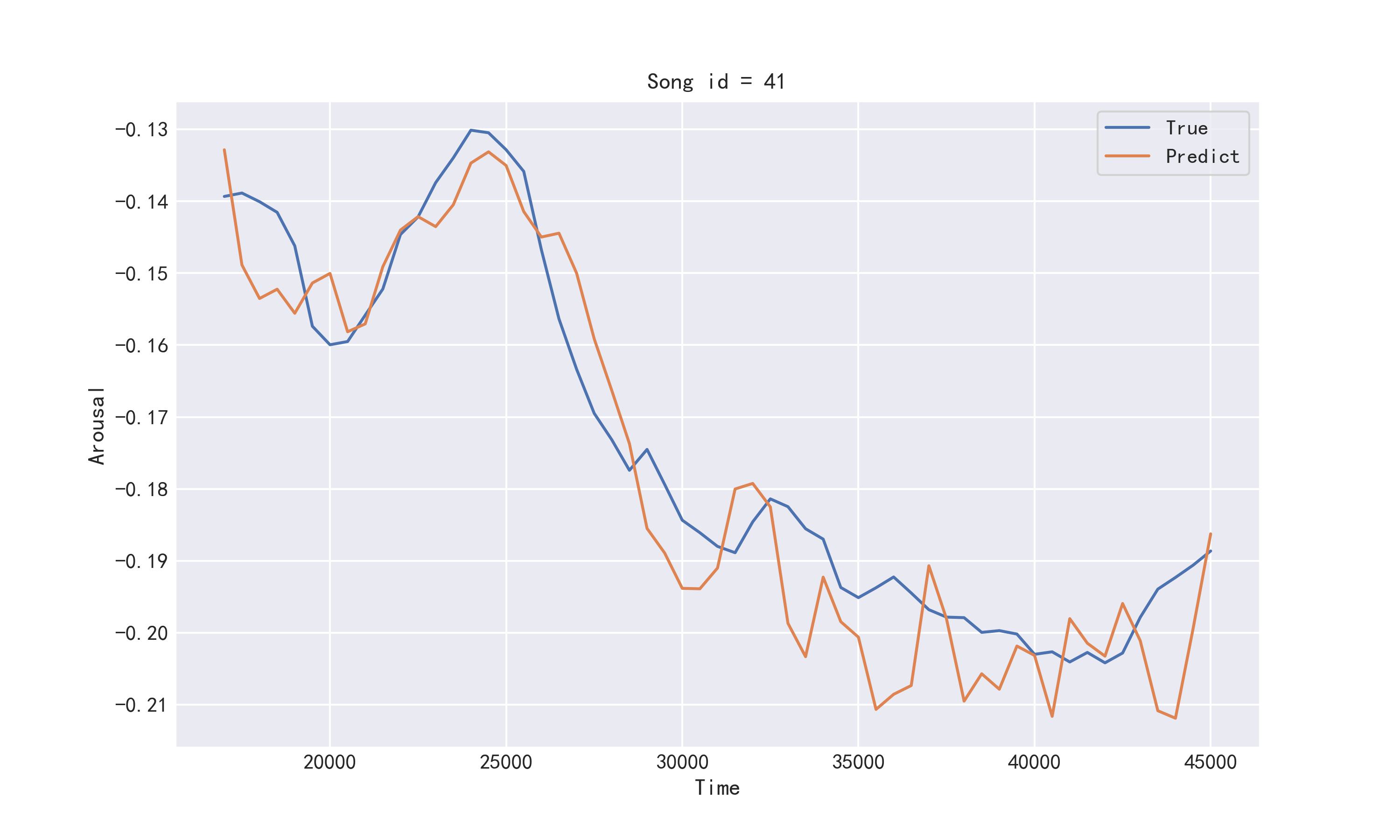

Supplement: S5 File — (ZIP) [file pone.0297712.s005.zip › All prediction results/prediction picture results(DEAM_100)/song_id_41.jpg]

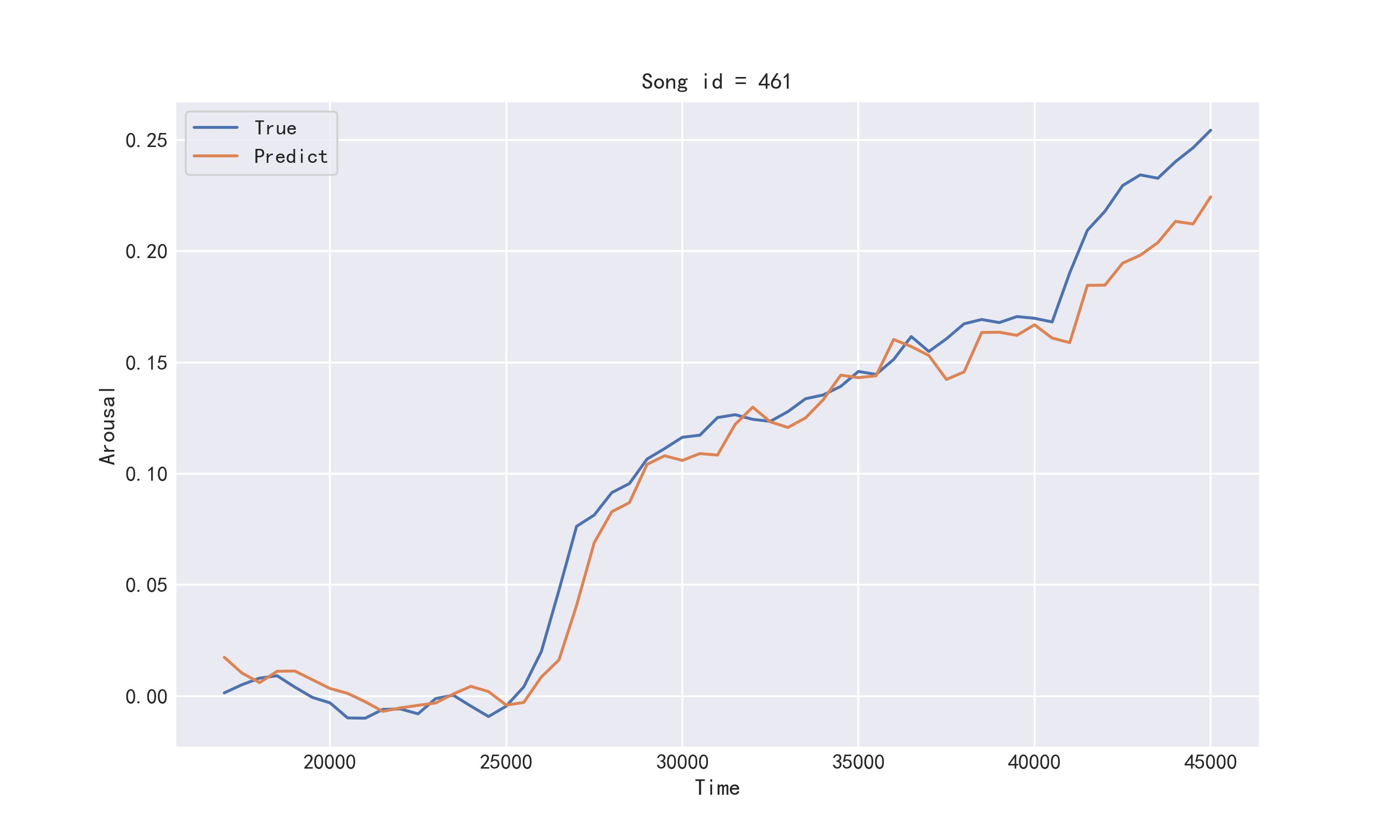

Supplement: S5 File — (ZIP) [file pone.0297712.s005.zip › All prediction results/prediction picture results(DEAM_100)/song_id_461.jpg]

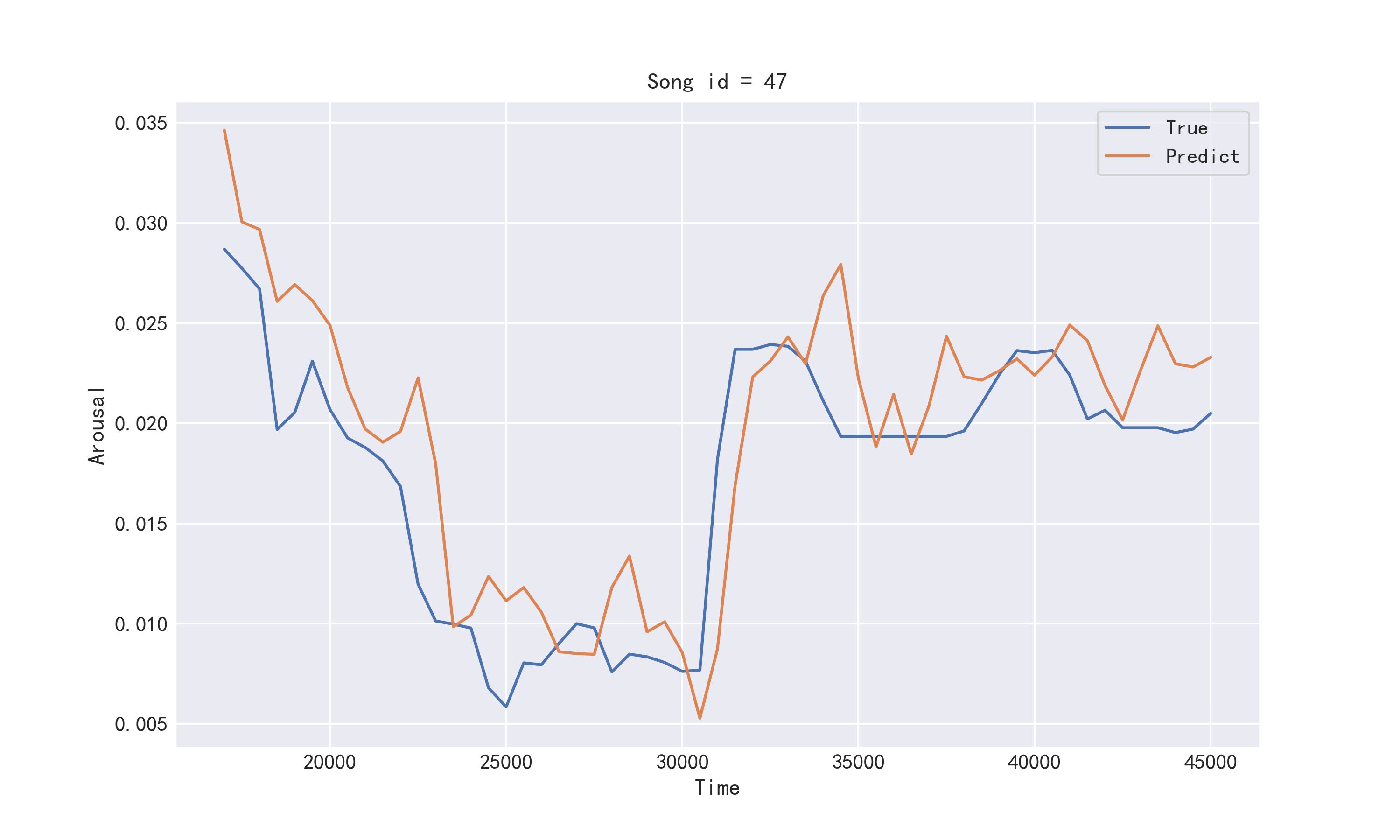

Supplement: S5 File — (ZIP) [file pone.0297712.s005.zip › All prediction results/prediction picture results(DEAM_100)/song_id_47.jpg]

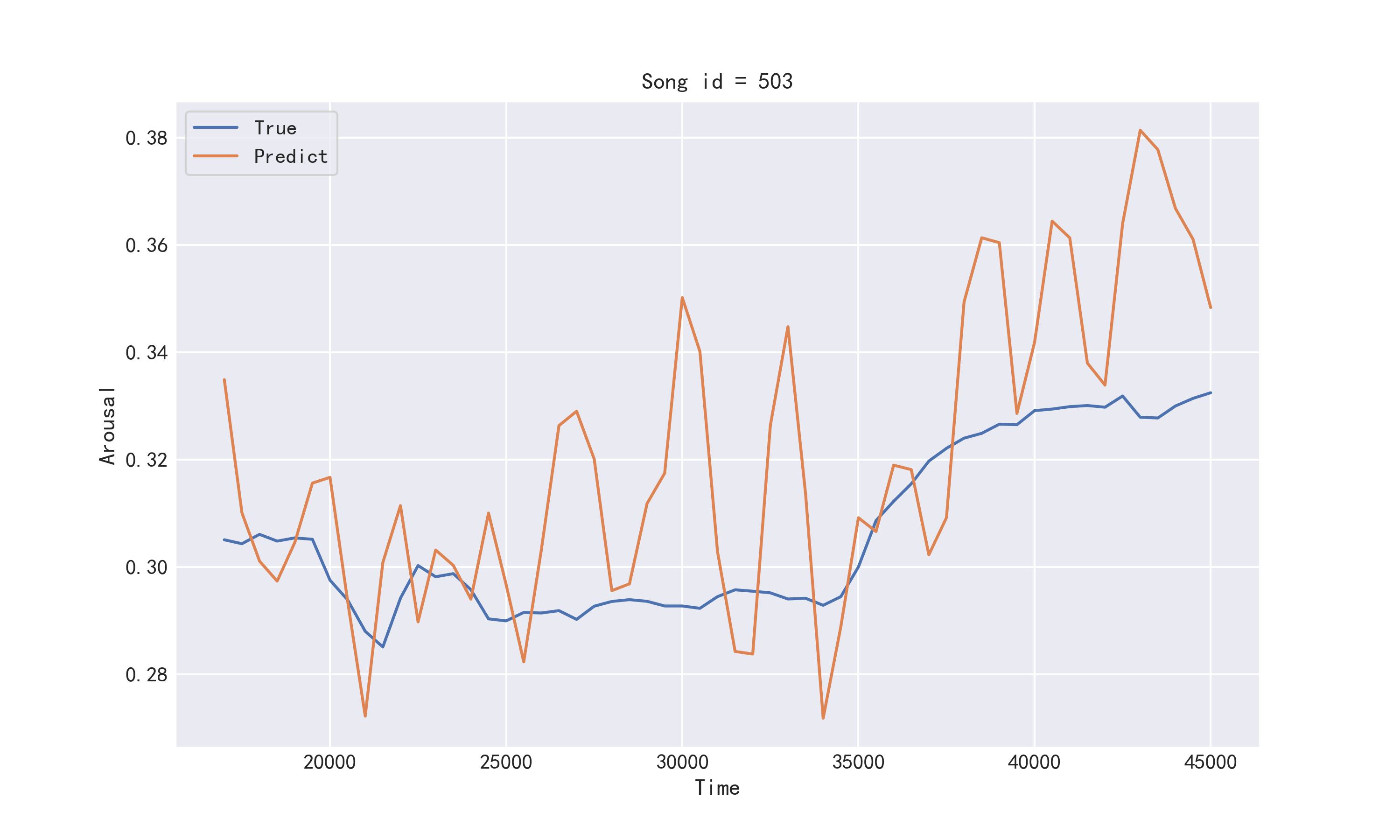

Supplement: S5 File — (ZIP) [file pone.0297712.s005.zip › All prediction results/prediction picture results(DEAM_100)/song_id_503.jpg]

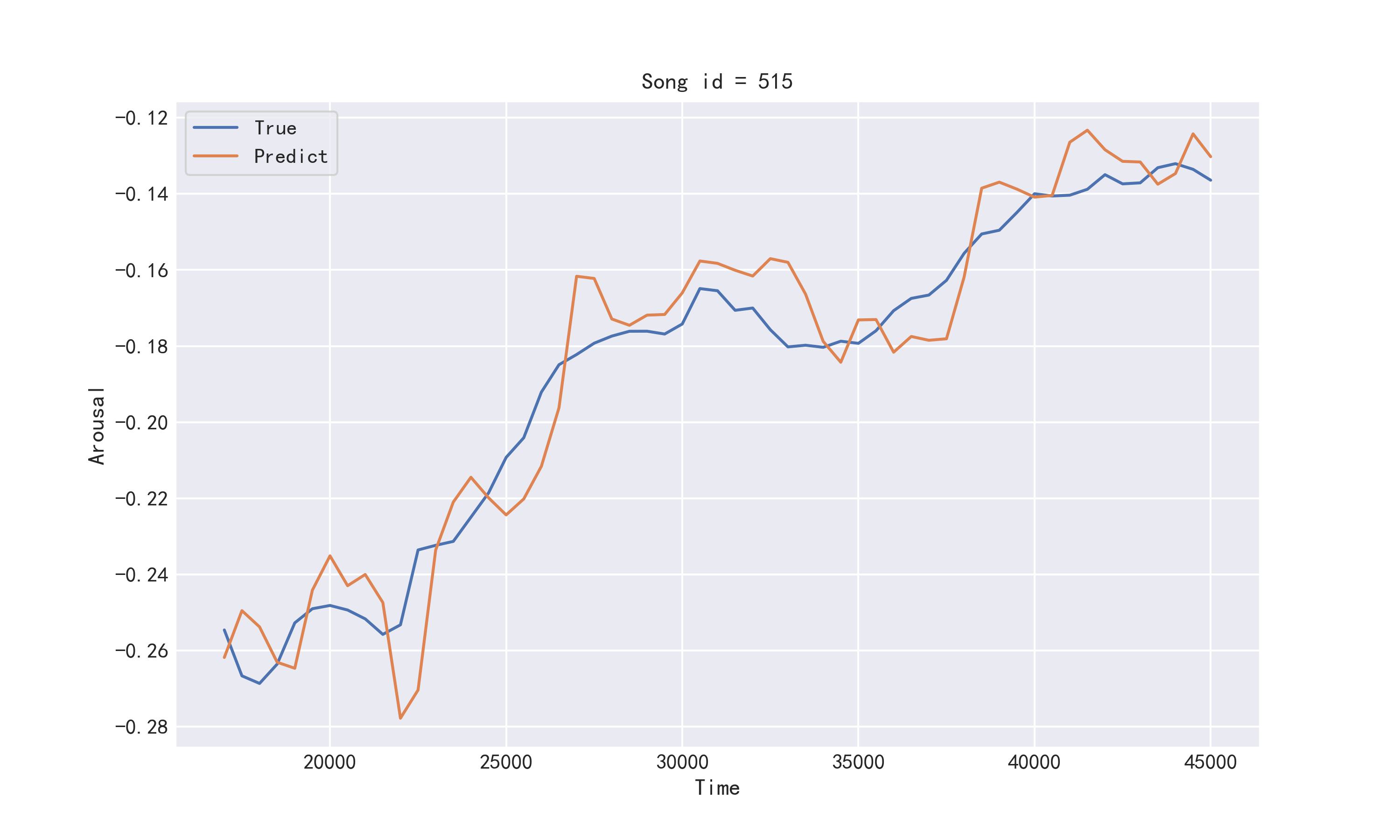

Supplement: S5 File — (ZIP) [file pone.0297712.s005.zip › All prediction results/prediction picture results(DEAM_100)/song_id_515.jpg]

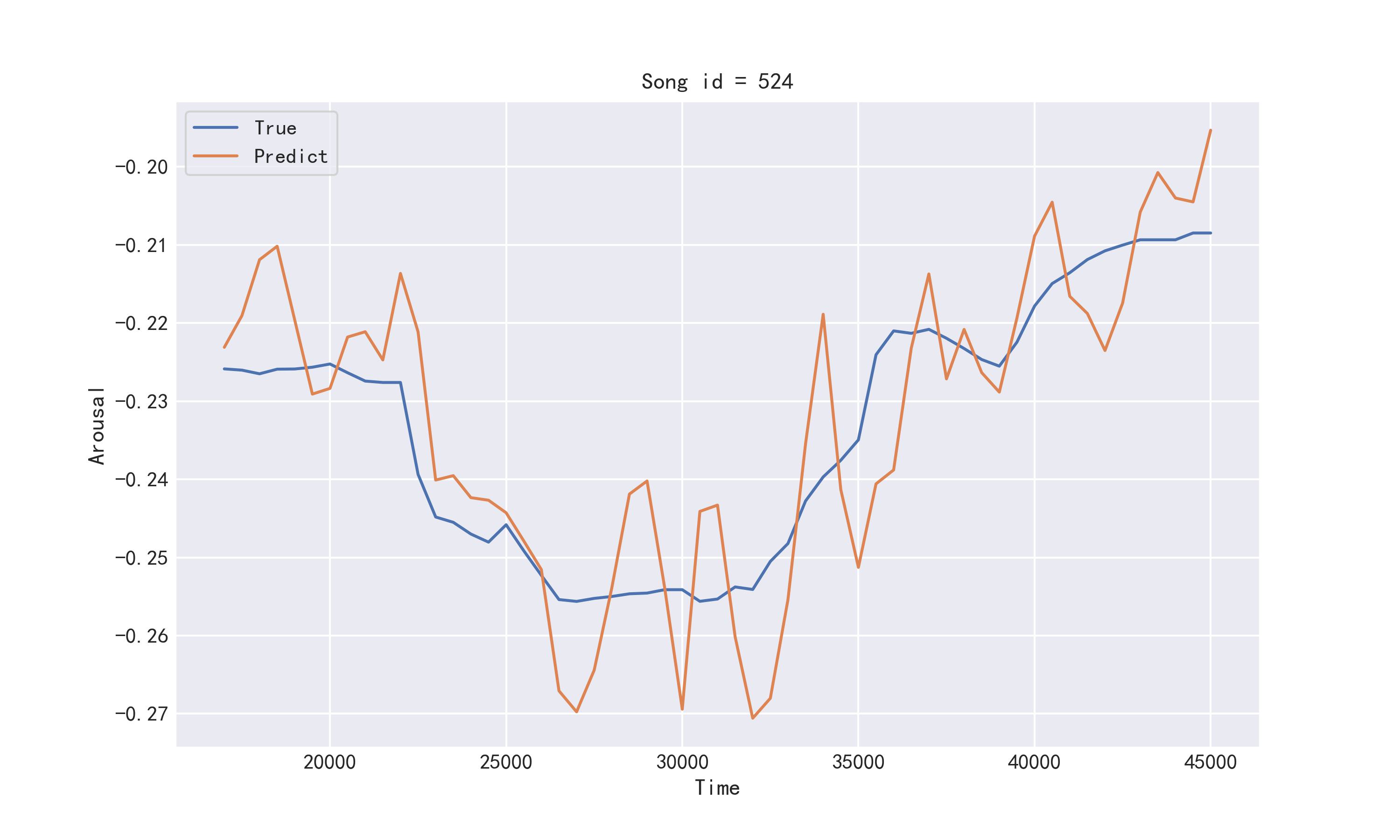

Supplement: S5 File — (ZIP) [file pone.0297712.s005.zip › All prediction results/prediction picture results(DEAM_100)/song_id_524.jpg]

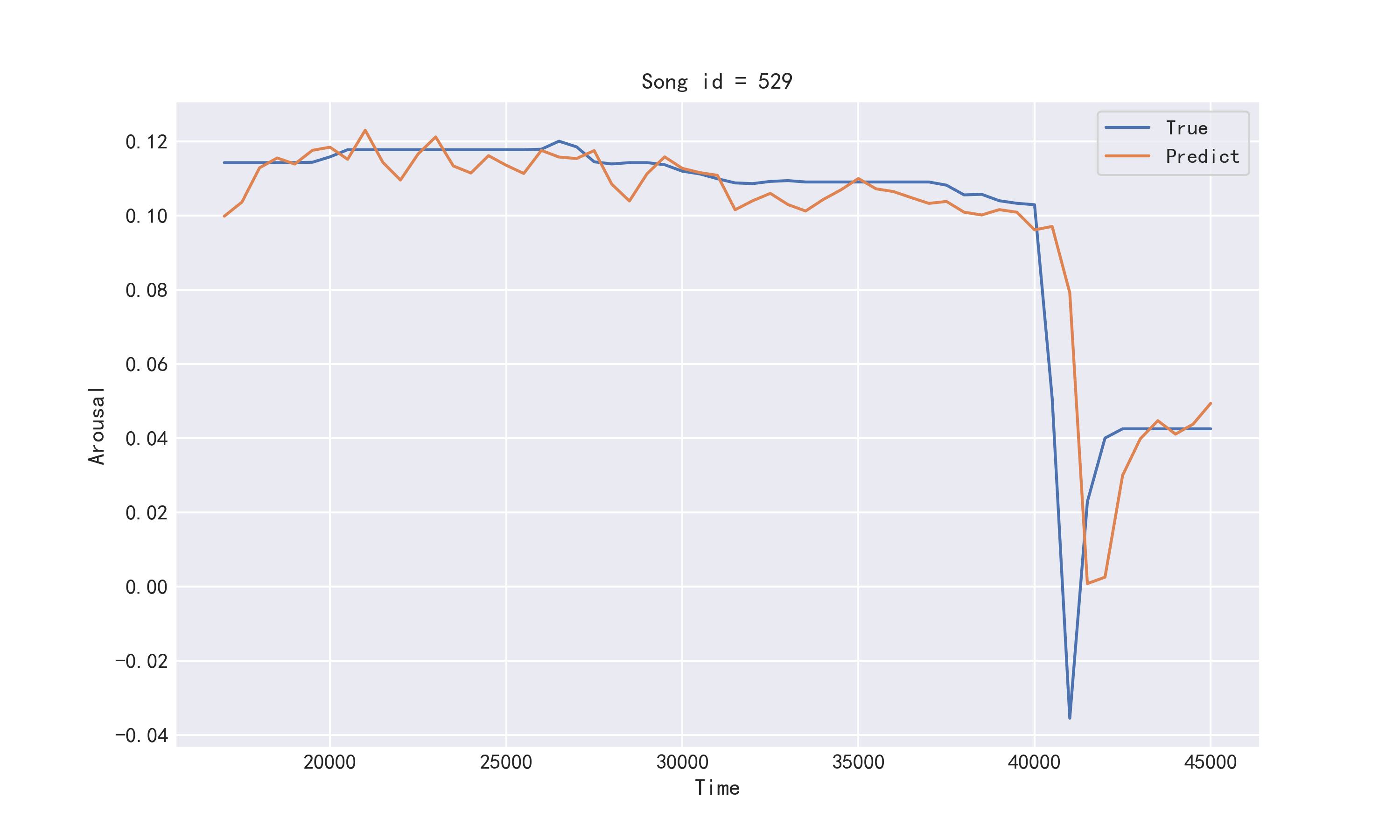

Supplement: S5 File — (ZIP) [file pone.0297712.s005.zip › All prediction results/prediction picture results(DEAM_100)/song_id_529.jpg]

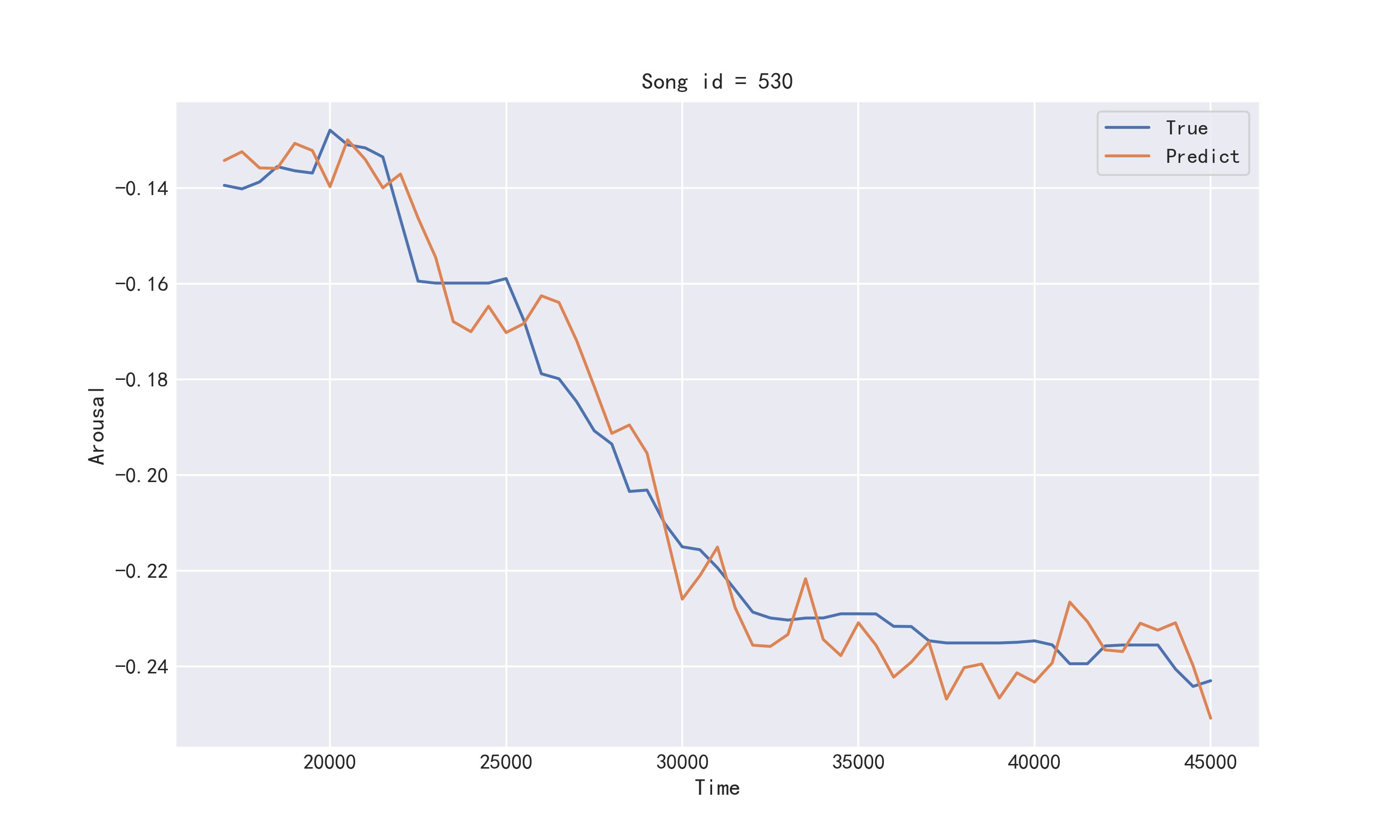

Supplement: S5 File — (ZIP) [file pone.0297712.s005.zip › All prediction results/prediction picture results(DEAM_100)/song_id_530.jpg]

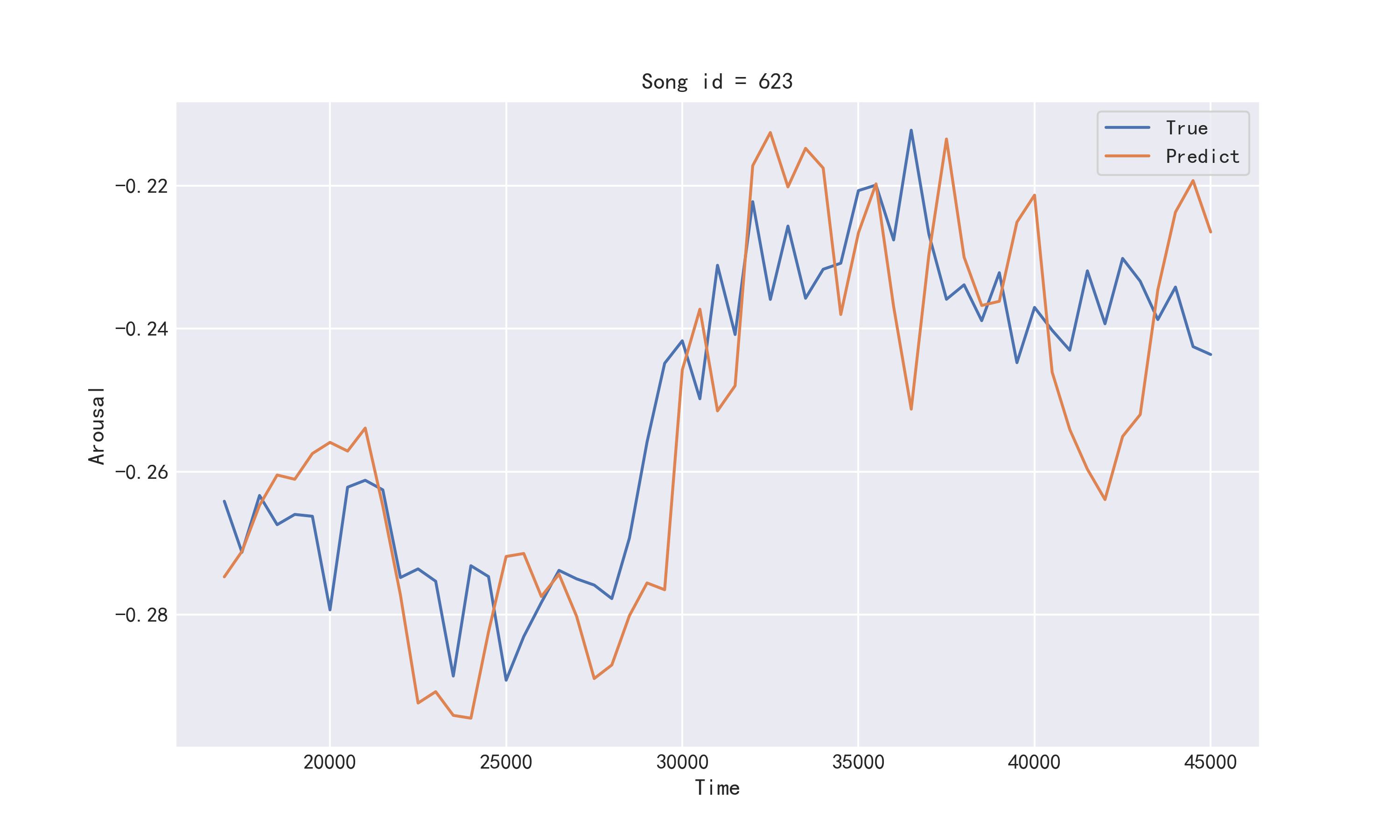

Supplement: S5 File — (ZIP) [file pone.0297712.s005.zip › All prediction results/prediction picture results(DEAM_100)/song_id_623.jpg]

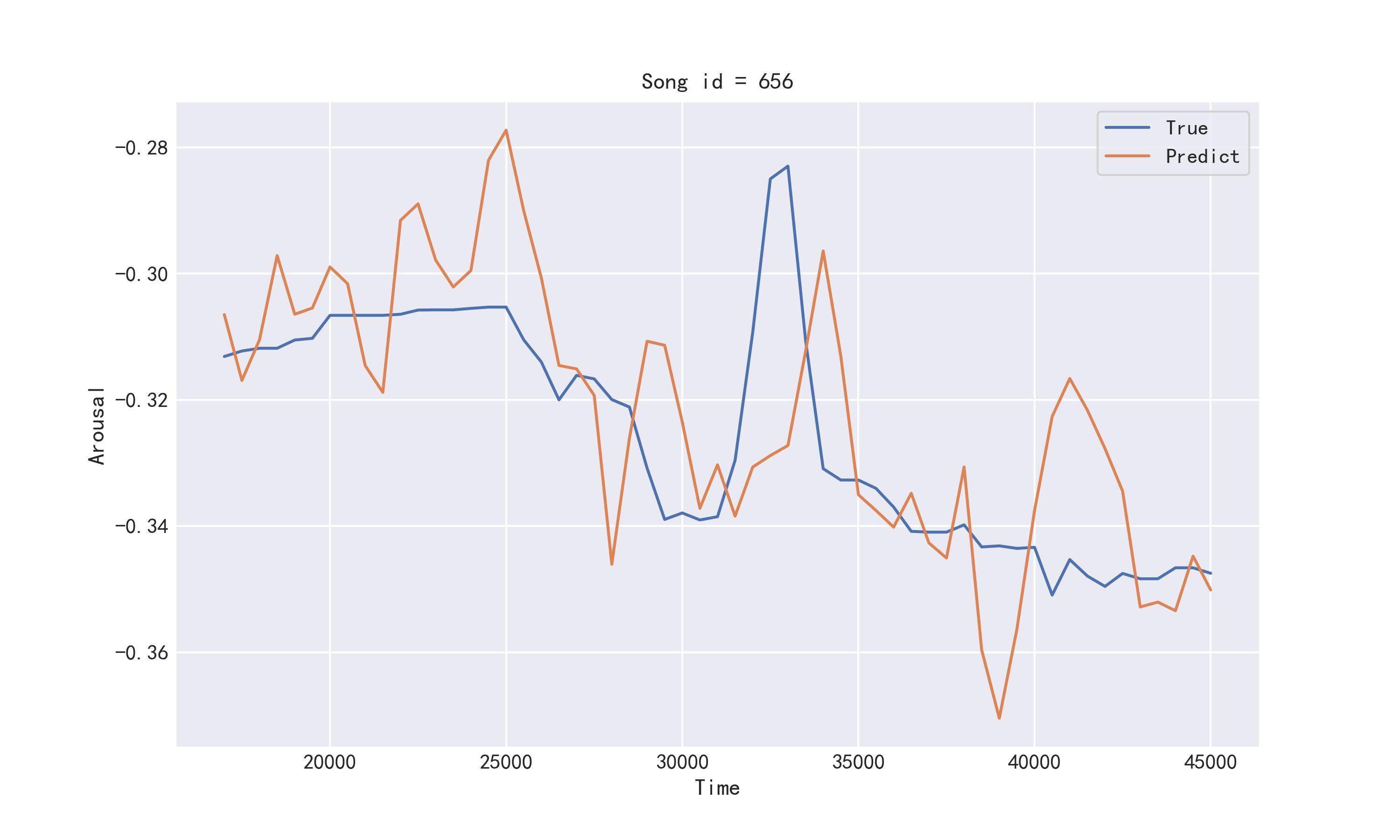

Supplement: S5 File — (ZIP) [file pone.0297712.s005.zip › All prediction results/prediction picture results(DEAM_100)/song_id_656.jpg]

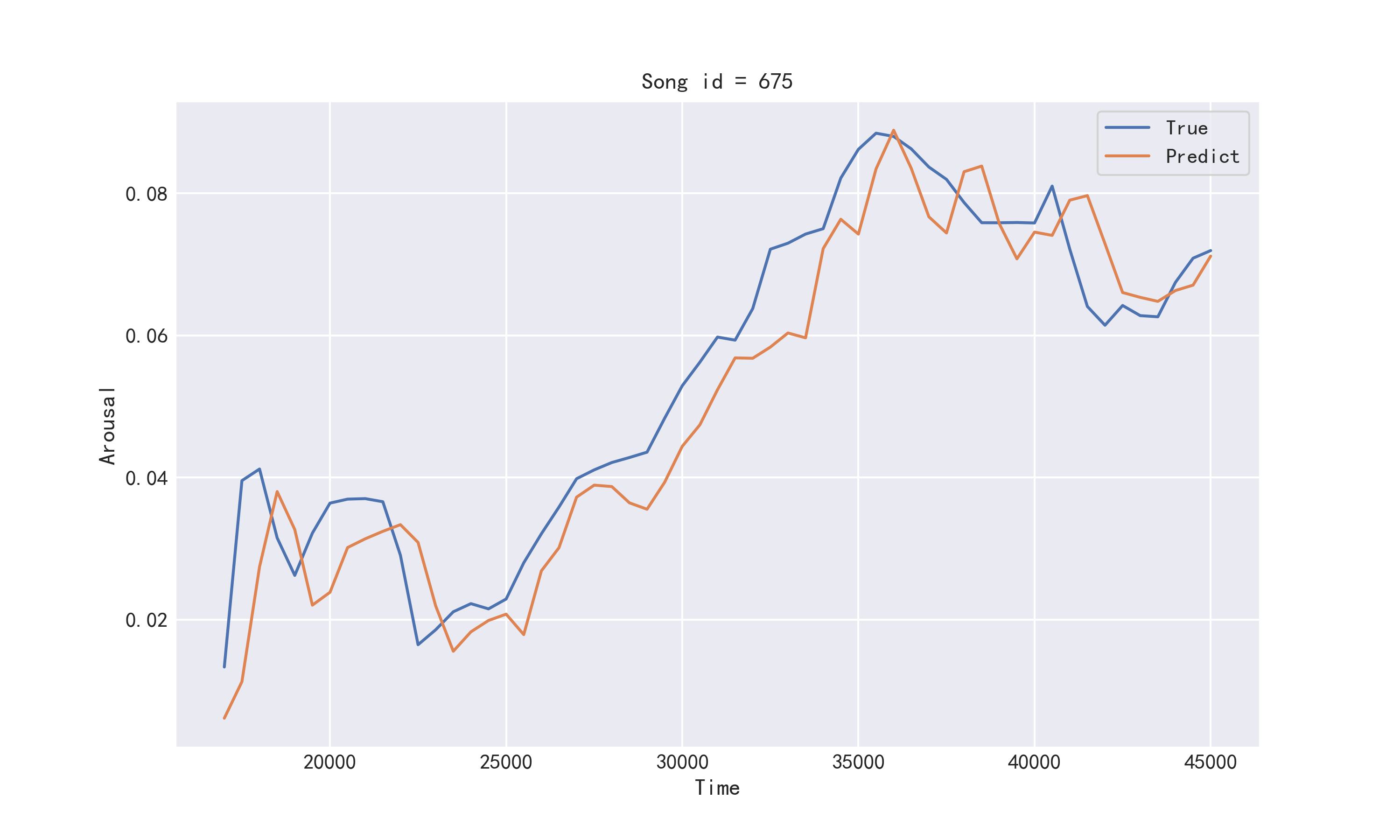

Supplement: S5 File — (ZIP) [file pone.0297712.s005.zip › All prediction results/prediction picture results(DEAM_100)/song_id_675.jpg]

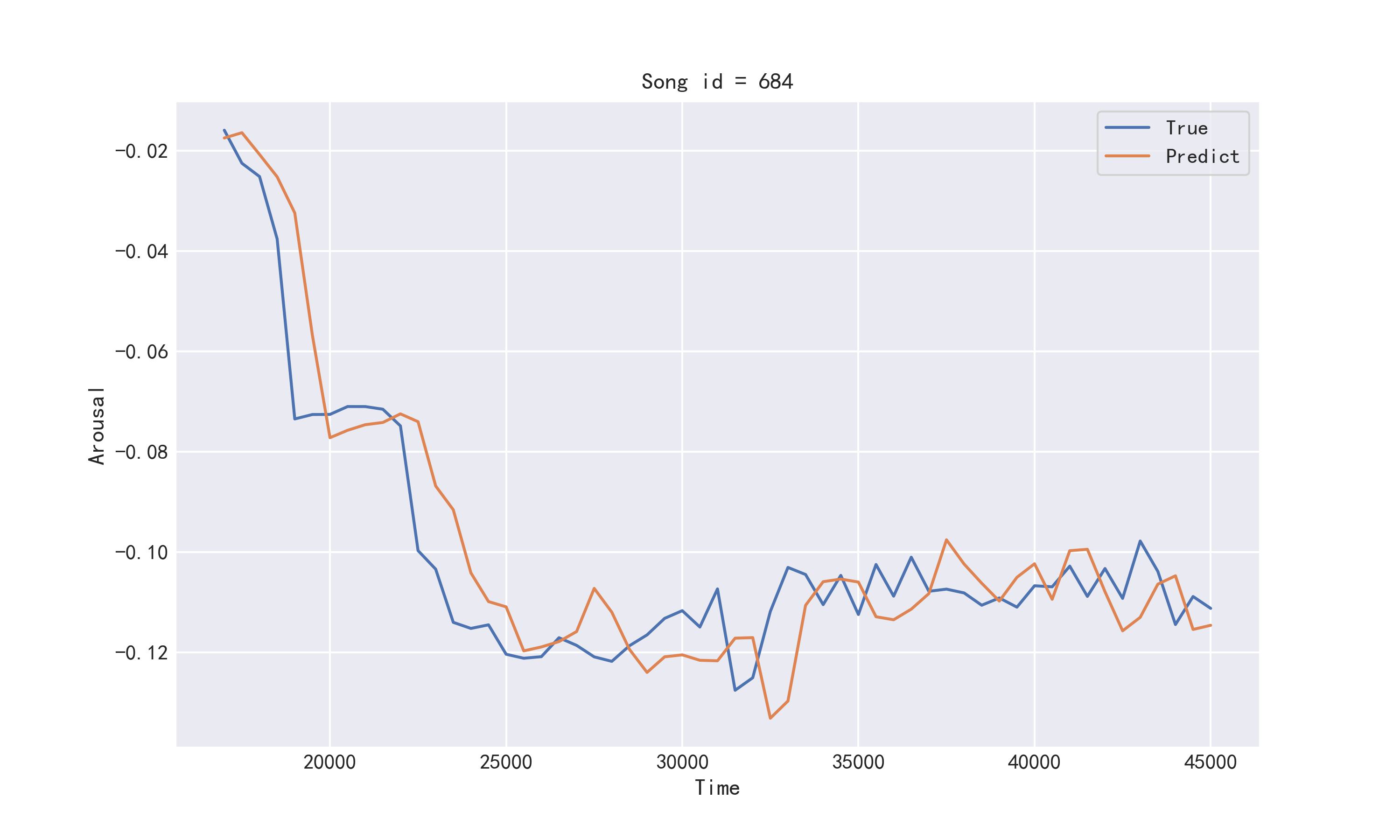

Supplement: S5 File — (ZIP) [file pone.0297712.s005.zip › All prediction results/prediction picture results(DEAM_100)/song_id_684.jpg]

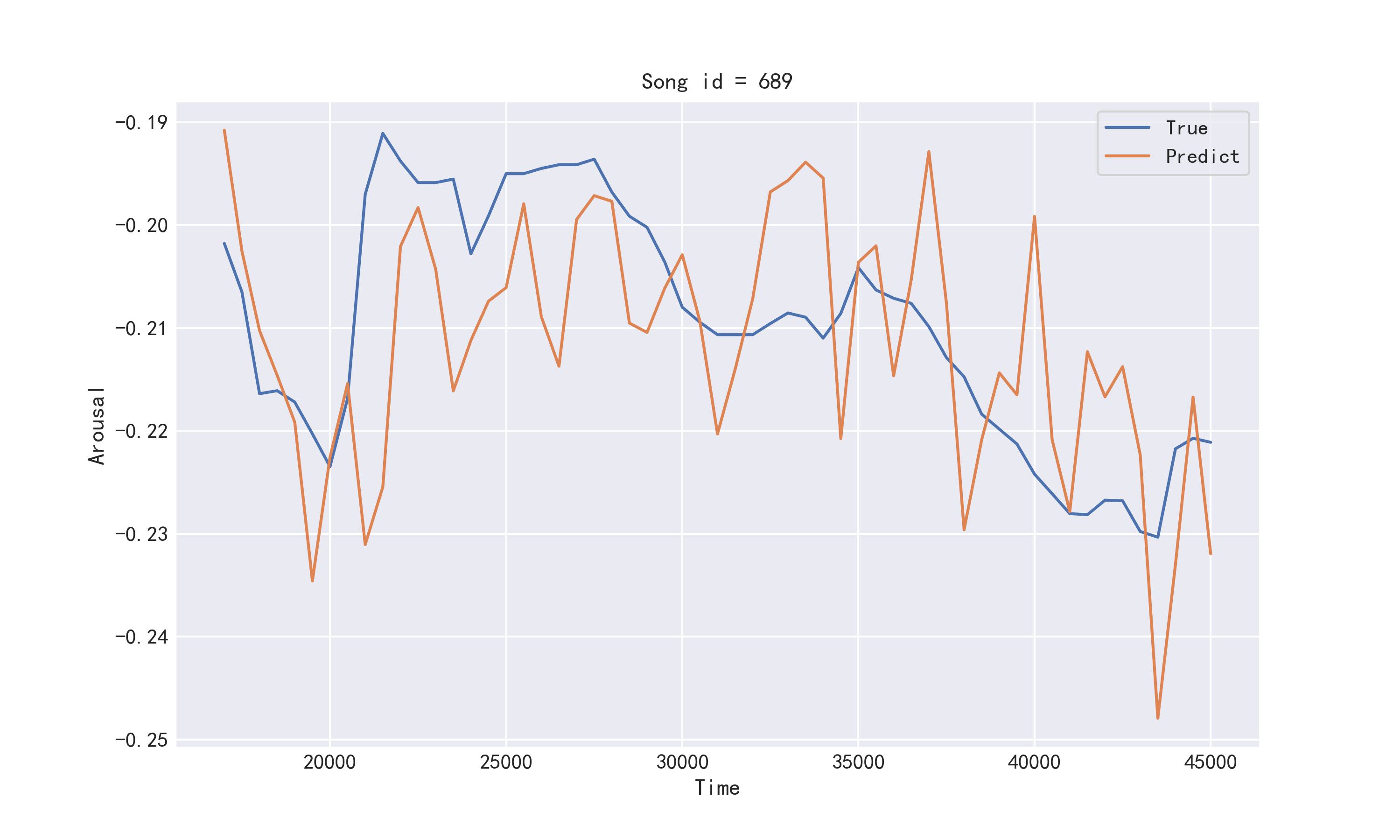

Supplement: S5 File — (ZIP) [file pone.0297712.s005.zip › All prediction results/prediction picture results(DEAM_100)/song_id_689.jpg]

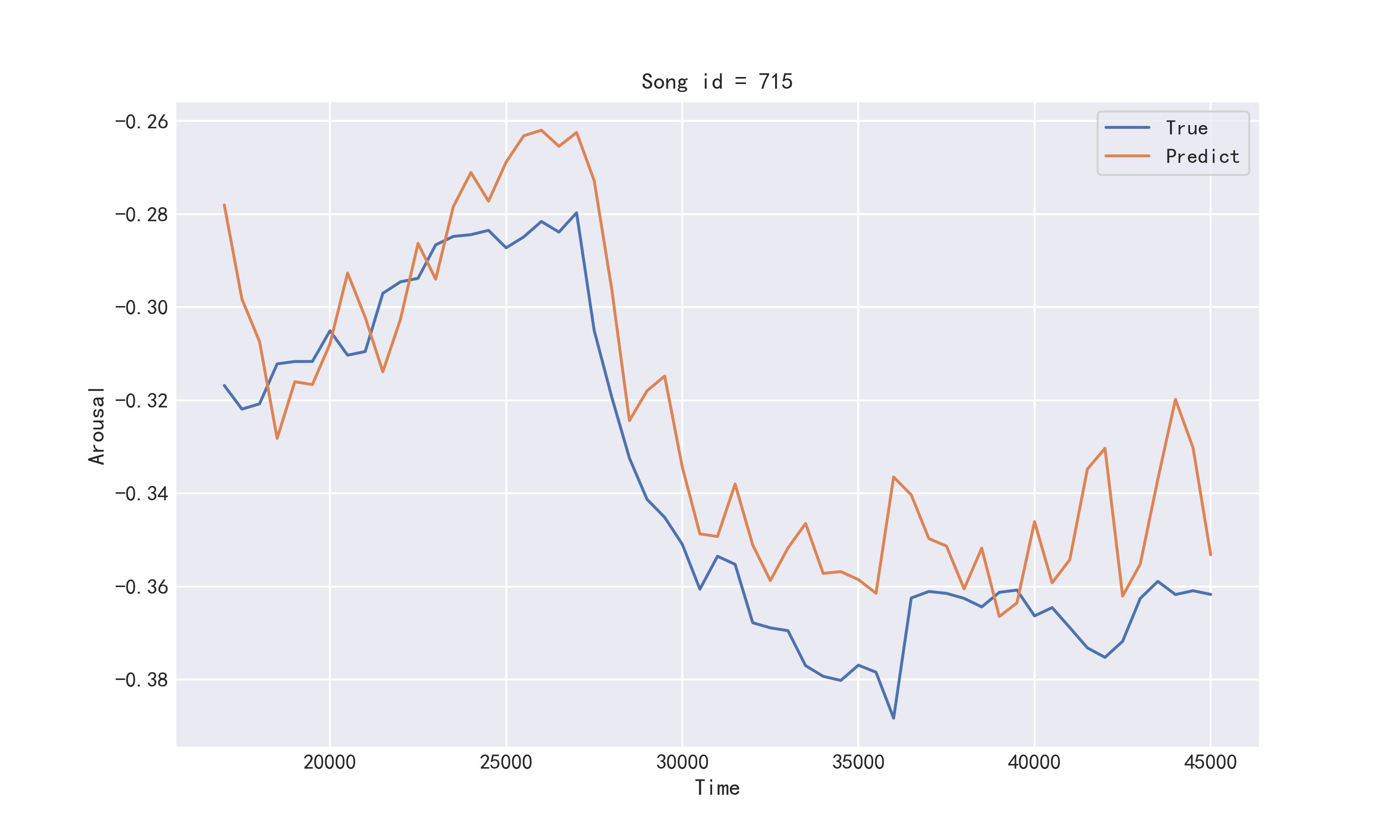

Supplement: S5 File — (ZIP) [file pone.0297712.s005.zip › All prediction results/prediction picture results(DEAM_100)/song_id_715.jpg]

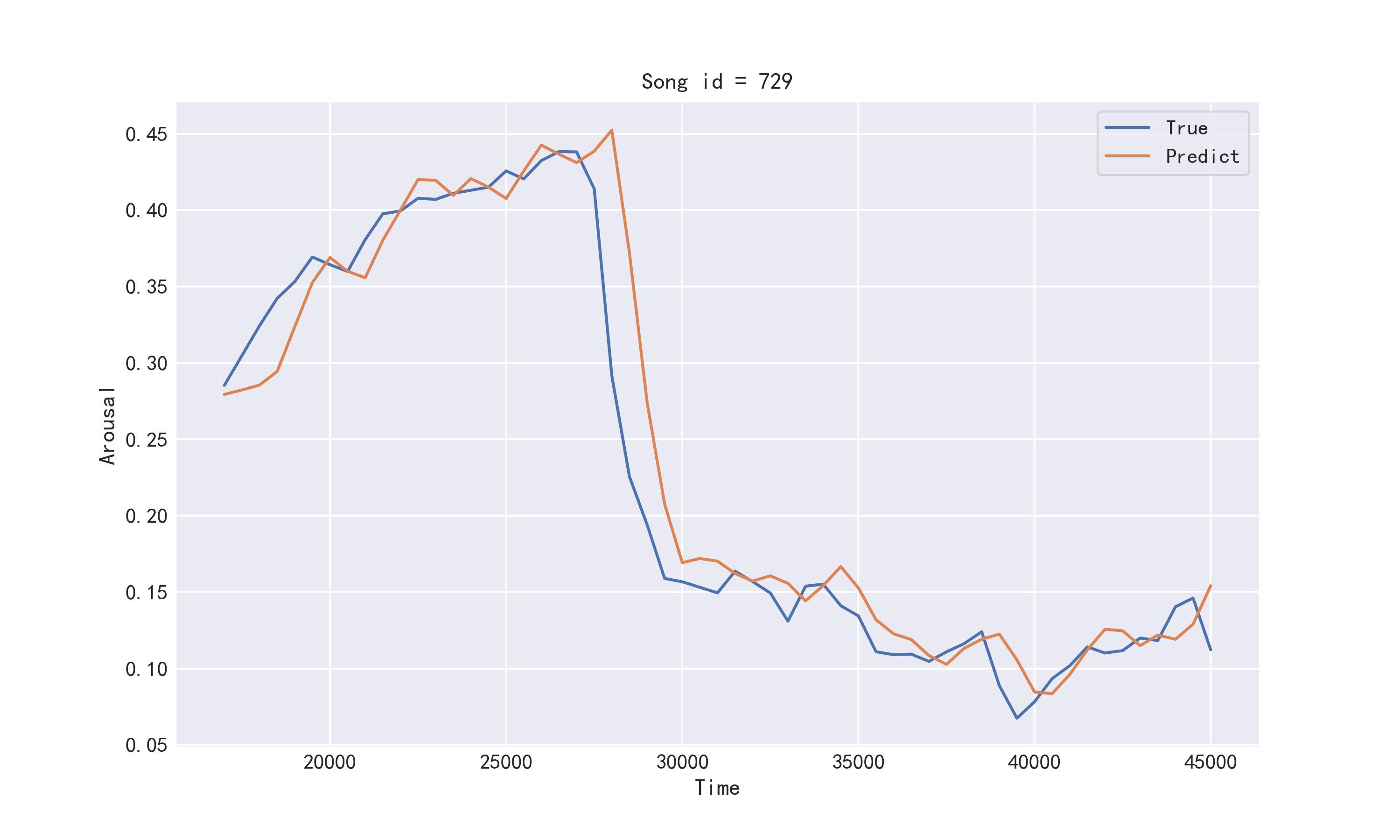

Supplement: S5 File — (ZIP) [file pone.0297712.s005.zip › All prediction results/prediction picture results(DEAM_100)/song_id_729.jpg]

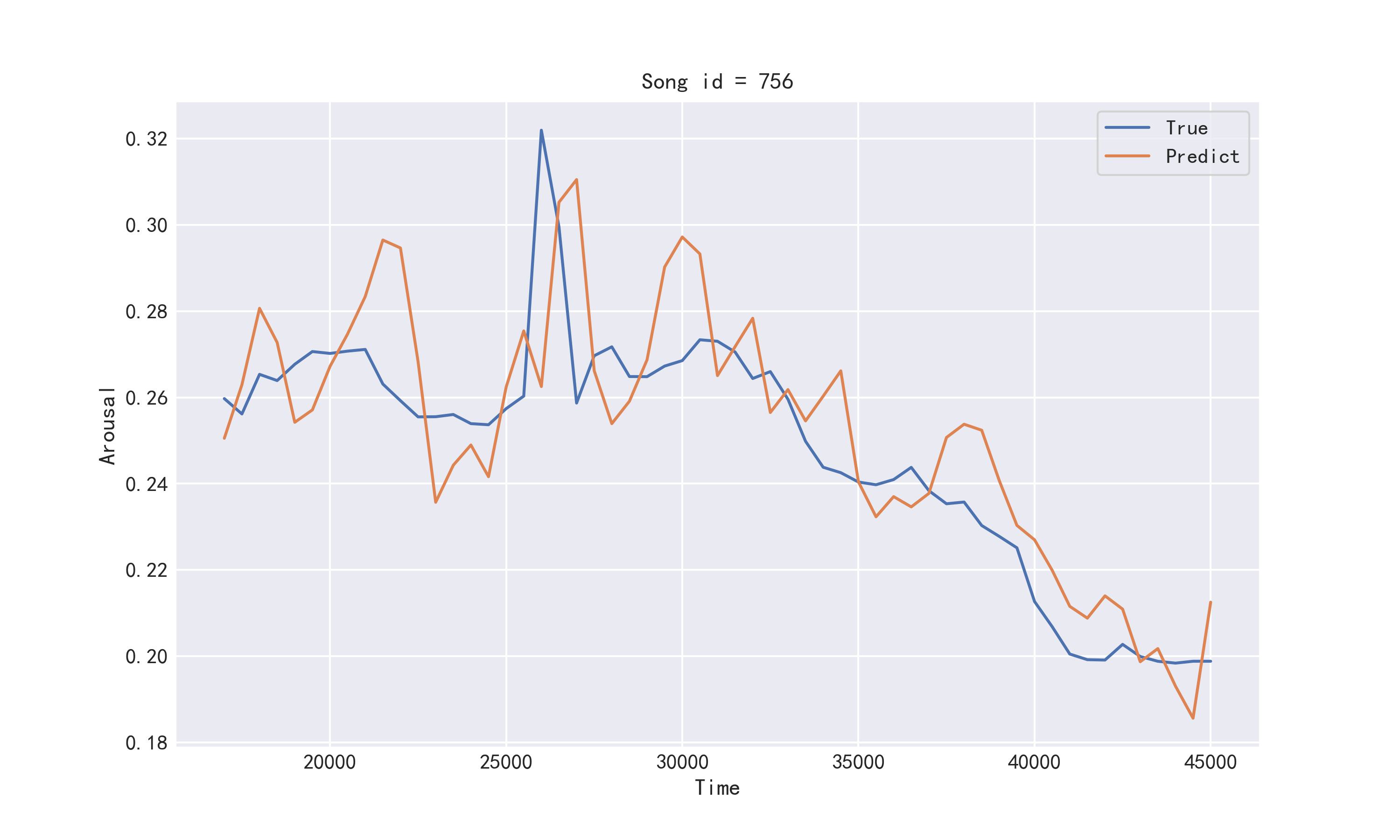

Supplement: S5 File — (ZIP) [file pone.0297712.s005.zip › All prediction results/prediction picture results(DEAM_100)/song_id_756.jpg]

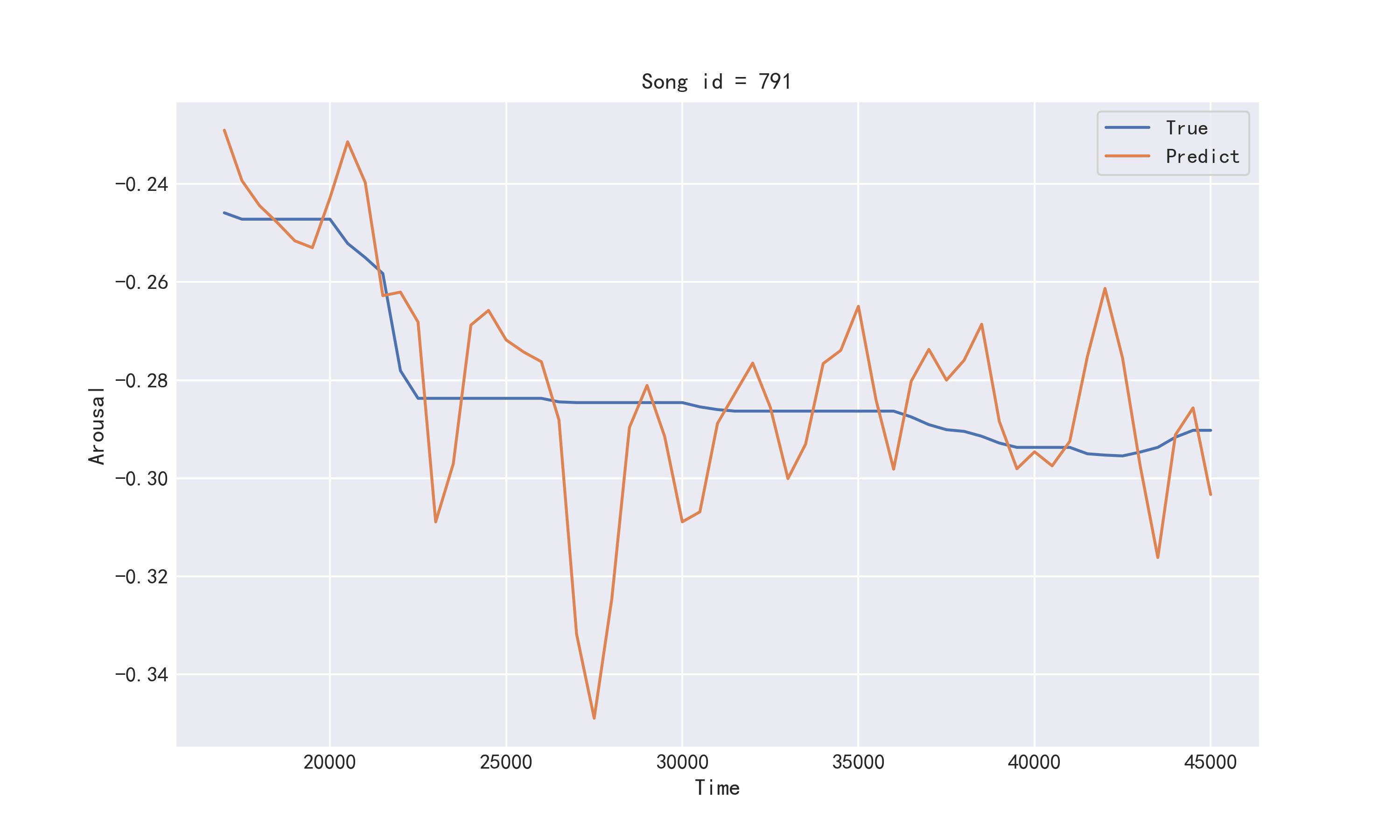

Supplement: S5 File — (ZIP) [file pone.0297712.s005.zip › All prediction results/prediction picture results(DEAM_100)/song_id_791.jpg]

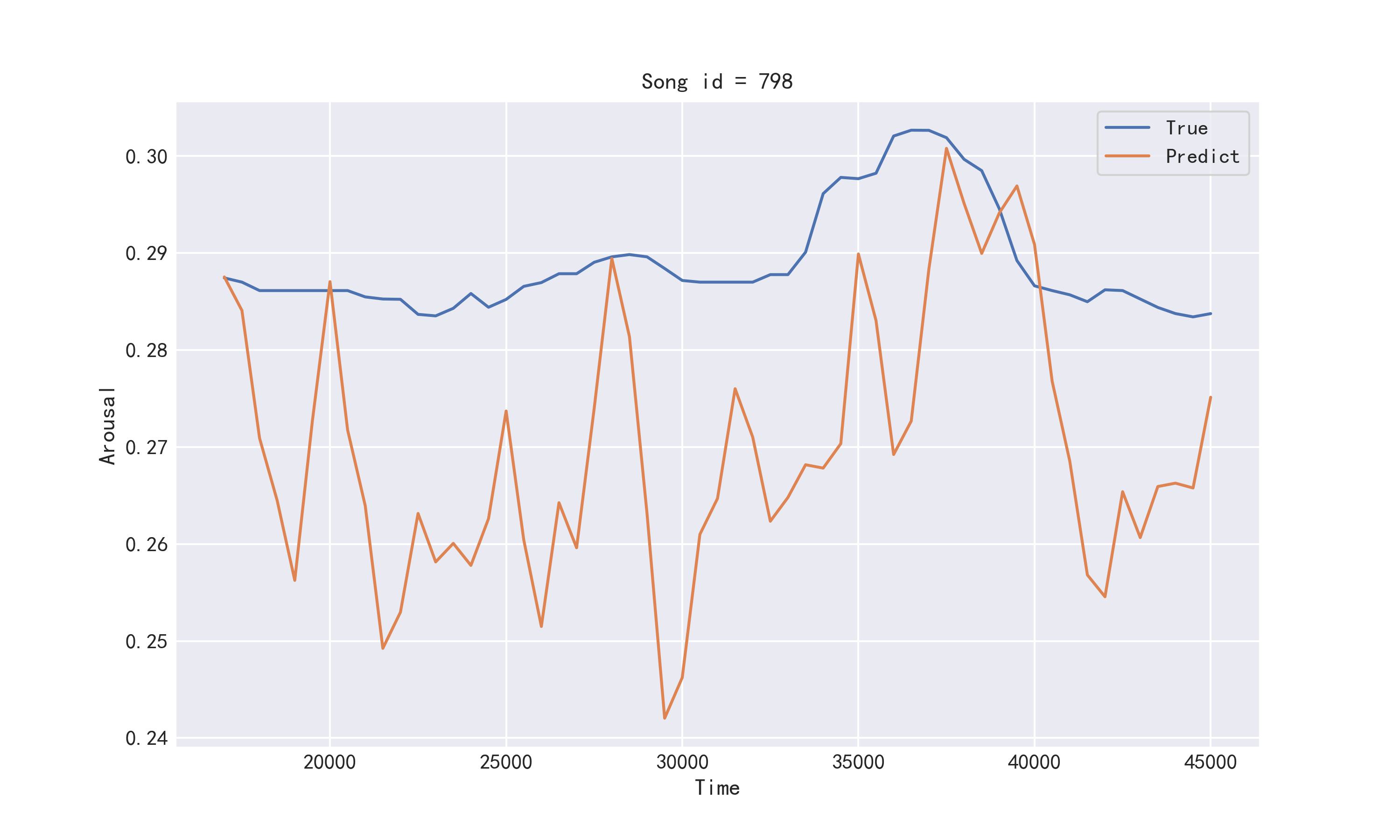

Supplement: S5 File — (ZIP) [file pone.0297712.s005.zip › All prediction results/prediction picture results(DEAM_100)/song_id_798.jpg]

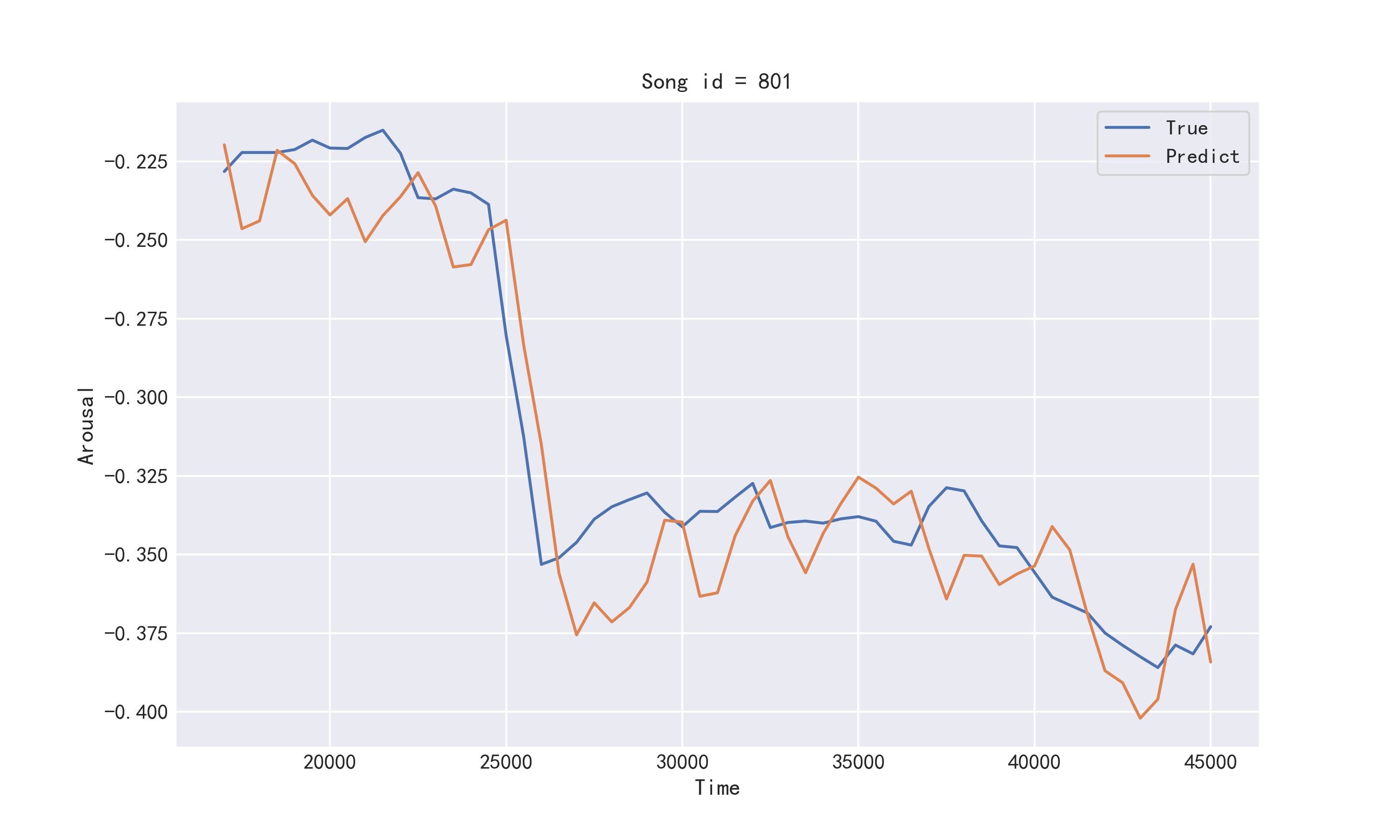

Supplement: S5 File — (ZIP) [file pone.0297712.s005.zip › All prediction results/prediction picture results(DEAM_100)/song_id_801.jpg]

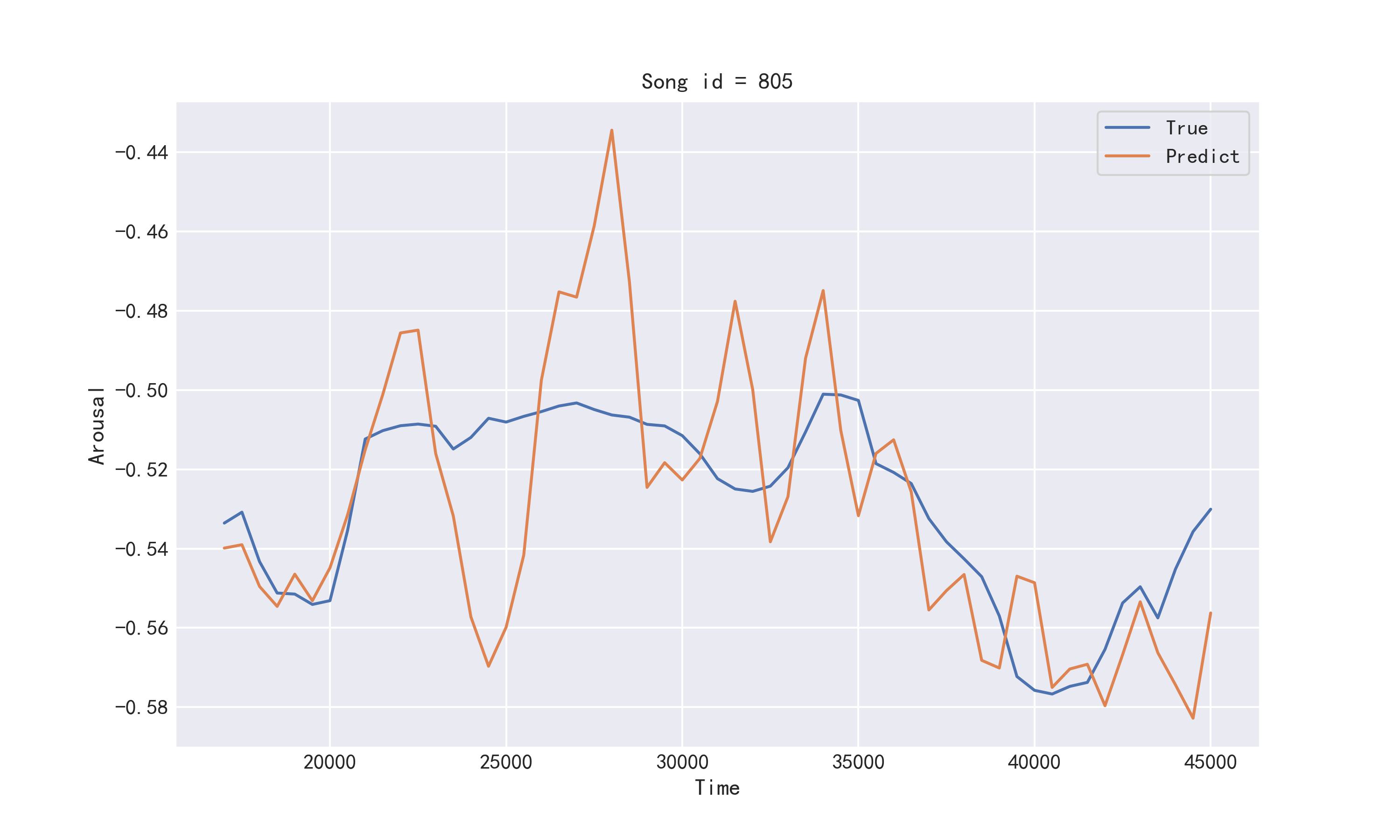

Supplement: S5 File — (ZIP) [file pone.0297712.s005.zip › All prediction results/prediction picture results(DEAM_100)/song_id_805.jpg]

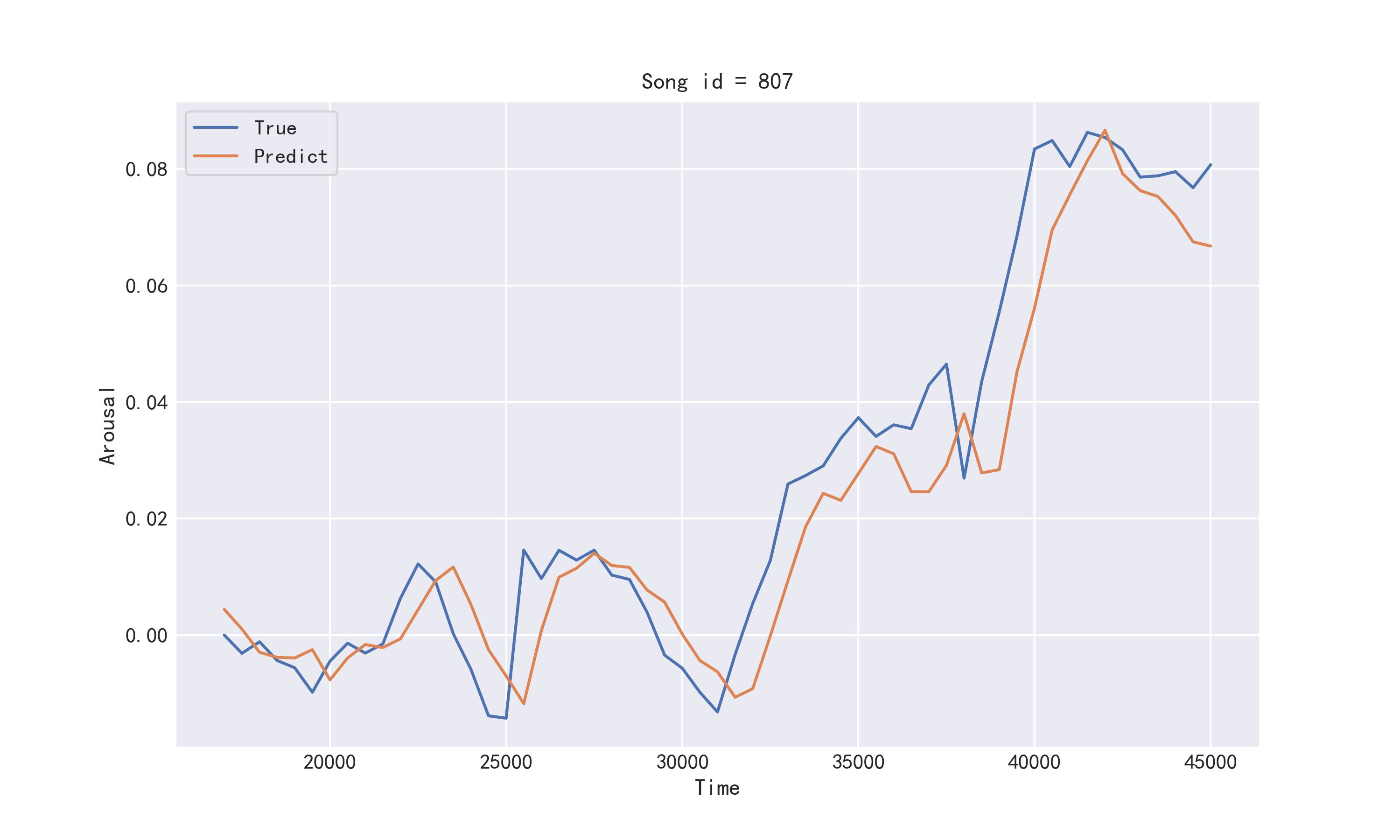

Supplement: S5 File — (ZIP) [file pone.0297712.s005.zip › All prediction results/prediction picture results(DEAM_100)/song_id_807.jpg]

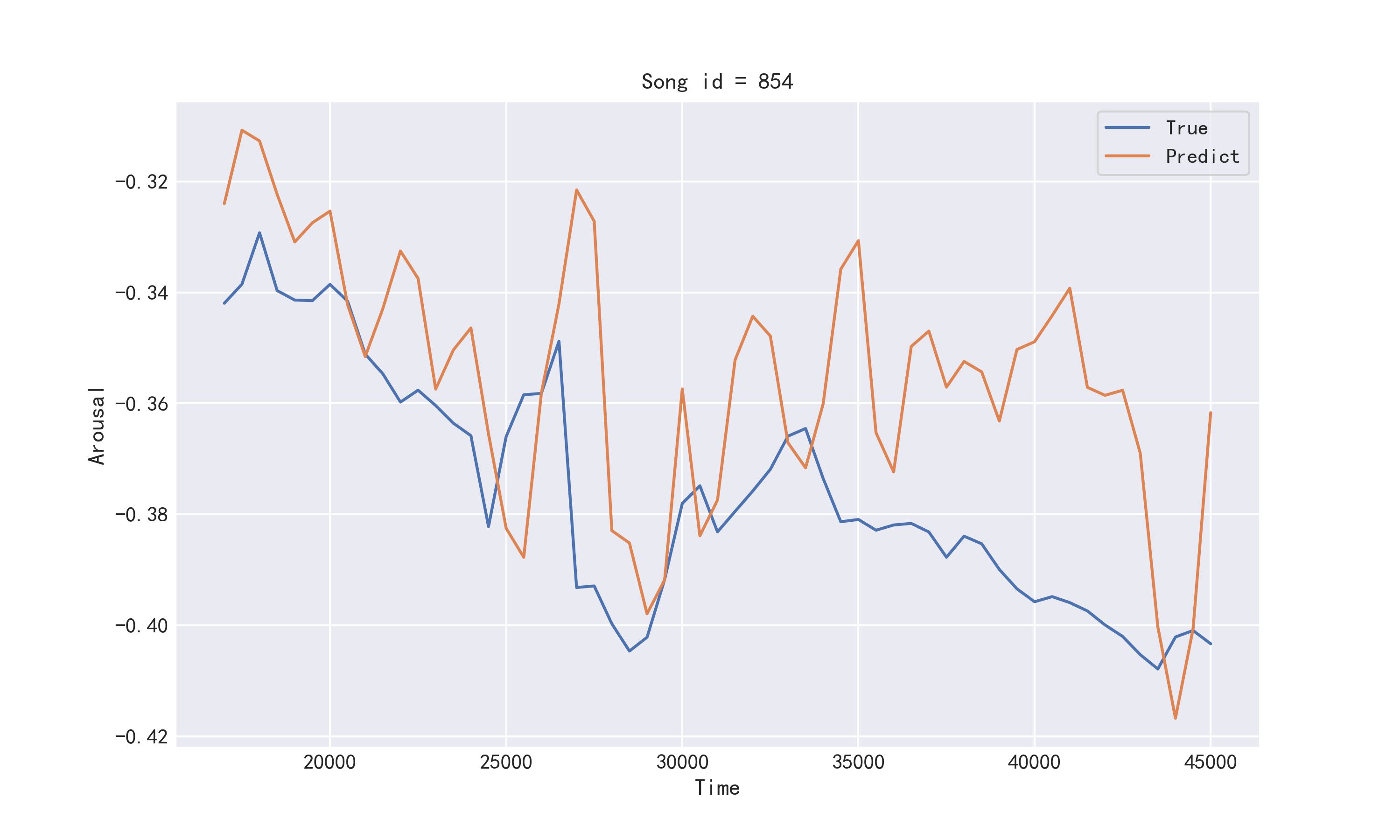

Supplement: S5 File — (ZIP) [file pone.0297712.s005.zip › All prediction results/prediction picture results(DEAM_100)/song_id_854.jpg]

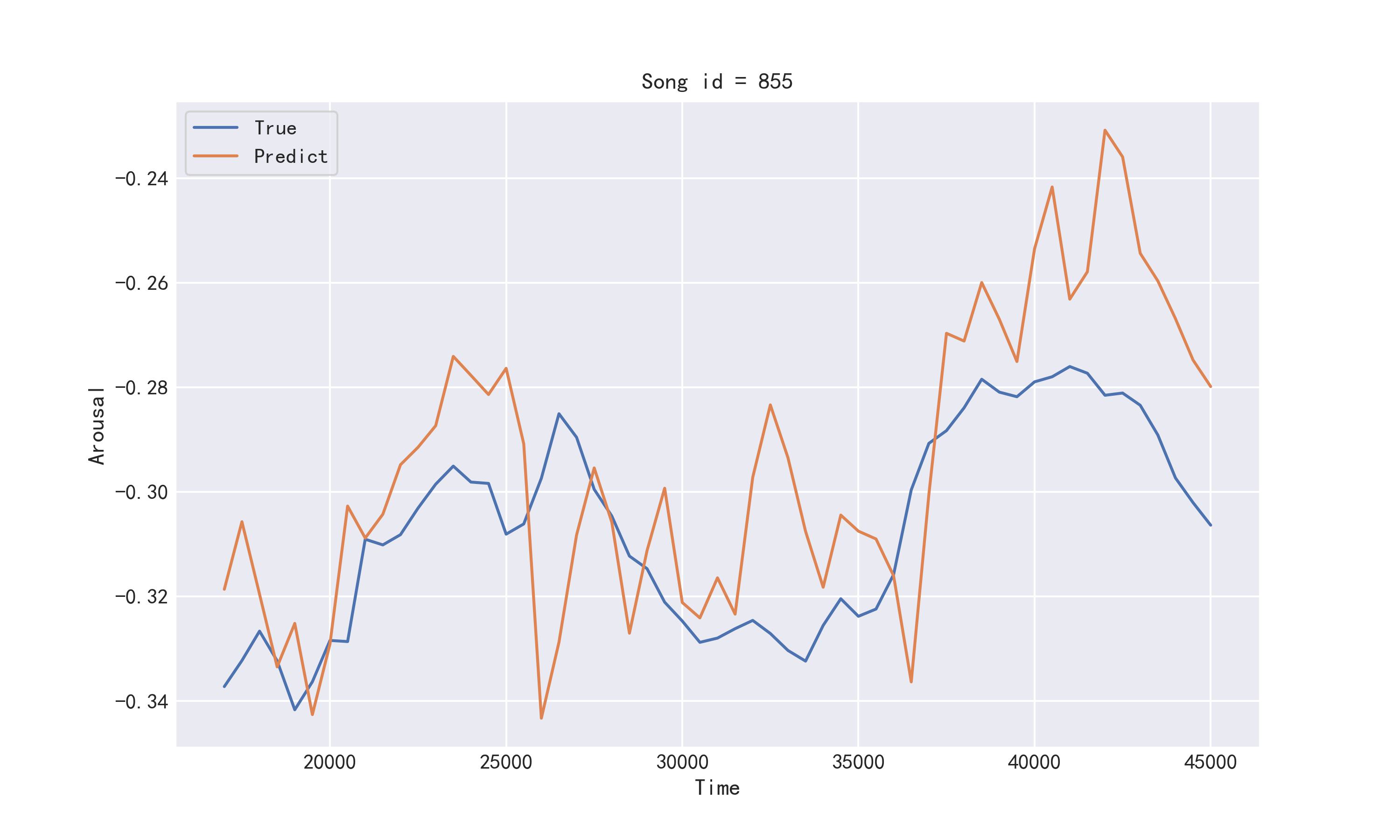

Supplement: S5 File — (ZIP) [file pone.0297712.s005.zip › All prediction results/prediction picture results(DEAM_100)/song_id_855.jpg]

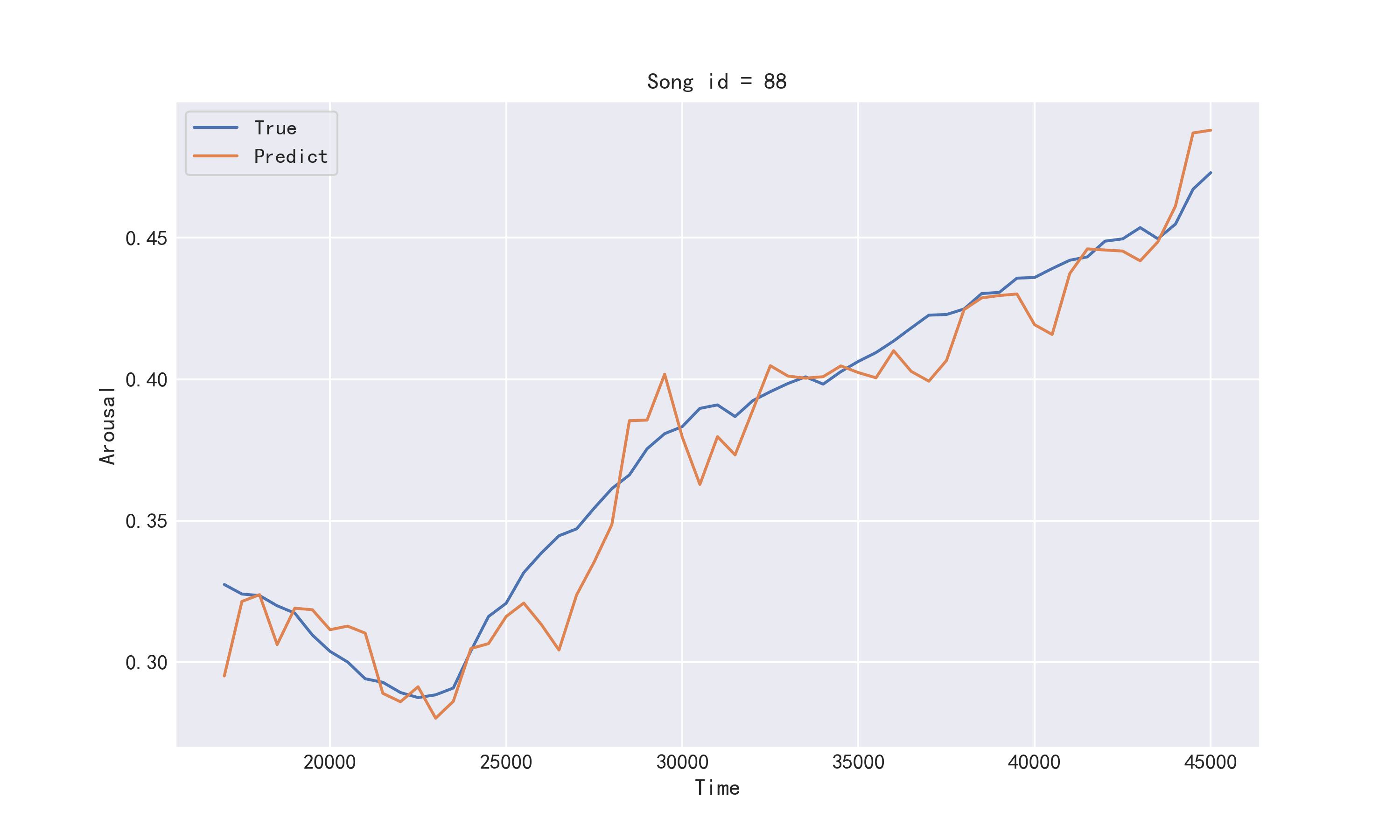

Supplement: S5 File — (ZIP) [file pone.0297712.s005.zip › All prediction results/prediction picture results(DEAM_100)/song_id_88.jpg]

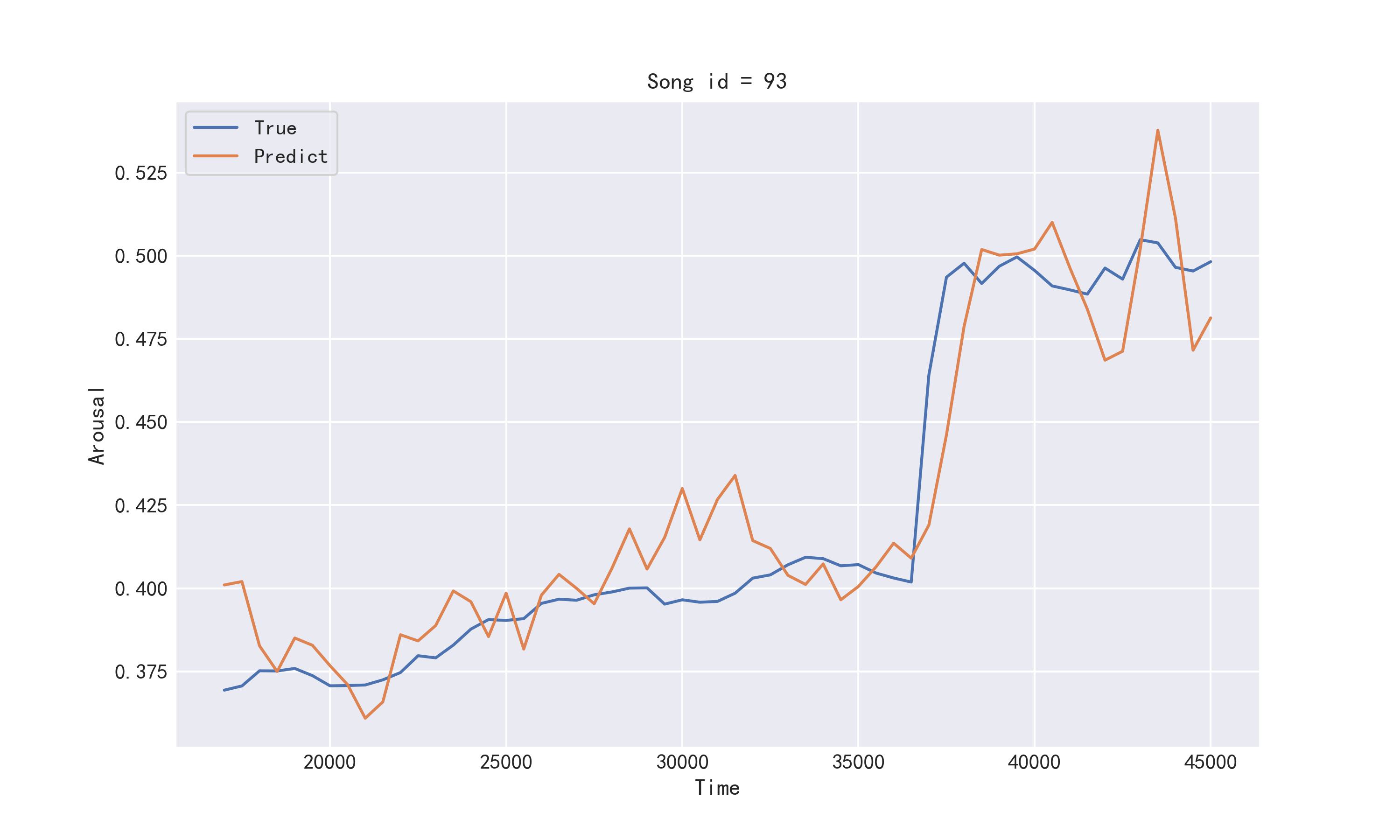

Supplement: S5 File — (ZIP) [file pone.0297712.s005.zip › All prediction results/prediction picture results(DEAM_100)/song_id_93.jpg]

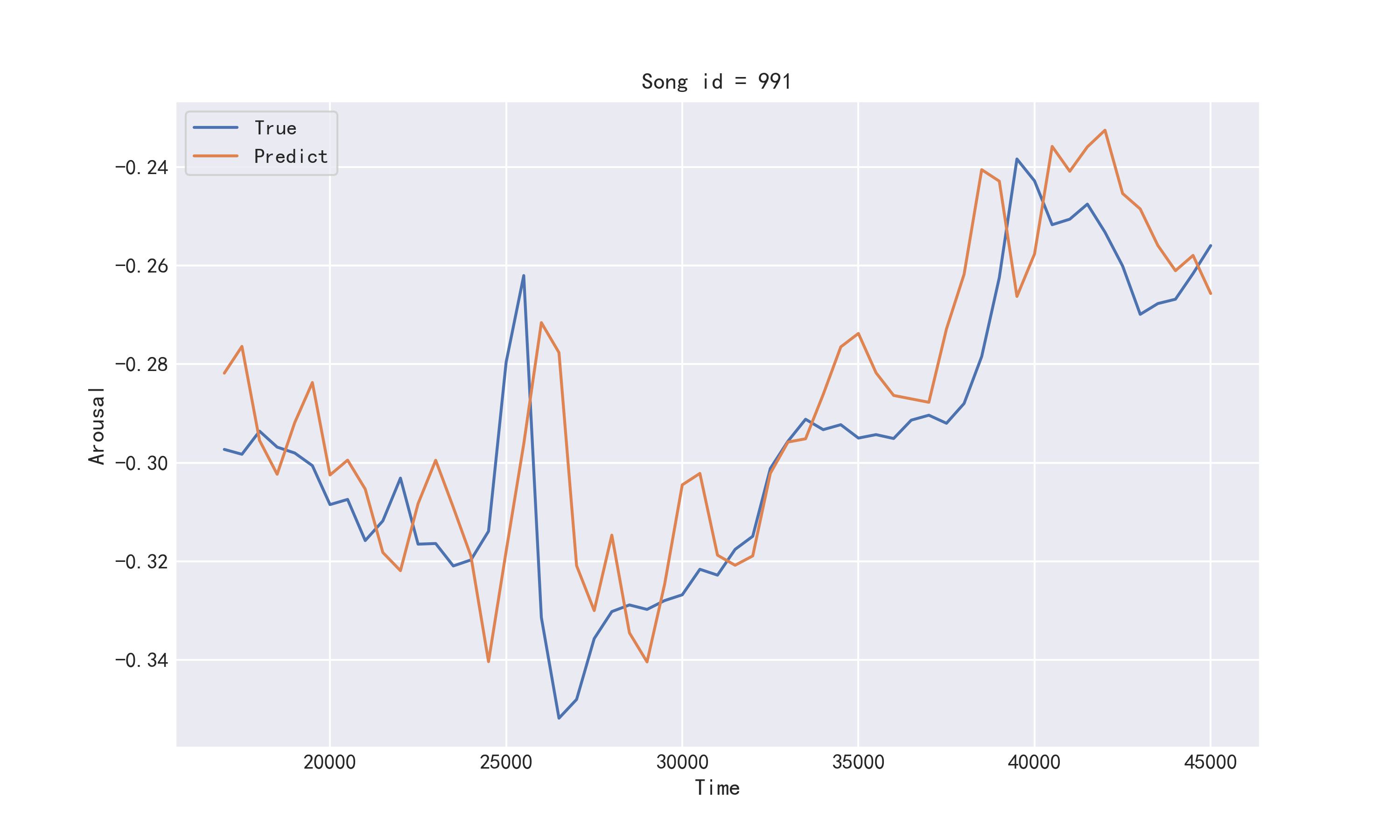

Supplement: S5 File — (ZIP) [file pone.0297712.s005.zip › All prediction results/prediction picture results(DEAM_100)/song_id_991.jpg]
