# Supplementary material for: Music-induced emotion flow modeling by ENMI Network
Source: PLoS One. 2024 Oct 21;19(10):e0297712. doi: 10.1371/journal.pone.0297712 (PMC11493256; doi:10.1371/journal.pone.0297712)
Supplement: Attachment — Submitted filename: Response to Reviewers.docx [file pone.0297712.s006.docx]

PONE-D-24-01232

Music-induced emotion flow modeling by ENMI Network

PLOS ONE

**Dear Editors and Reviewers:**

Thank you very much for giving us an opportunity to revise our manuscript. We appreciate the editors and reviewers very much for your constructive comments and suggestions on our manuscript entitled “**Music-induced emotion flow modeling by ENMI Network**”

Upon receiving your notification, we promptly delved into the reviewer's comments and suggestions, recognizing the depth of their insights into both the specific sections and the overall structure of the paper. The constructive feedback not only offers a fresh perspective but also provides valuable directions for refining the manuscript. Understanding the stringent academic standards set by PLOS ONE, we are dedicated to revising the paper to meet the highest standards and ensure it attains optimal academic excellence.

I sincerely appreciate your and the reviewer's recognition of my work, and I look forward to aligning the revised manuscript more closely with the expectations of the journal. I will work diligently to address the reviewer's comments comprehensively, aiming to make a positive contribution towards the paper's ultimate acceptance.

Best regards,

Lead author: Yunrui Shang

E-mail: [m15031528057@163.com](mailto:m15031528057@163.com)

Corresponding author: Yinhua Liu

E-mail: [liuyinhua@qdu.edu.cn](mailto:liuyinhua@qdu.edu.cn)

**Explanation: Comments	Response	Titles and location	Revision**

**Mark according to the file named “Revised Manuscript with Track Changes”**

**Response to reviewer #1：**

**Comment 1：The manuscript lacks literature review section. The authors must discuss existing studies in this field in a separate section and clearly mention the challenges of previous methods.**

Response 1: Thank you for your valuable suggestions. In the "Introduction" section, we have exemplified and discussed past studies on MER, with a particular focus on the last five years of research on dynamic musical emotion recognition using regression theory. Subsequently, we included two challenges to previous research in the manuscript. Finally, the advantages of our model are introduced, and we outline our work and innovations at the end of the "Introduction".

Revision 1: Introduction - P.3-4. In recent years, researchers have sought to incorporate attention mechanisms into the MER task to further enhance accuracy. ...... These studies highlight the importance of considering two conditions simultaneously to enhance the accuracy of dynamic music emotion recognition: a) A complete and effective set of musical features. ...... b) Models with perfect long sequence prediction capabilities. ......To our knowledge, there are limited studies in Music Emotion Recognition (MER) employing regression methods to simultaneously investigate dynamic emotional prediction. ......(See the details in the manuscript)

**Comment 2：Section 2 is hard to follow and understand. Authors have to provide more information and make the equations more comprehensible for authors.**

Response 2: I apologize for the trouble I caused you with the second part of my article. We have completely updated the logical structure of the textual narrative in Section II and added more information about the A-V model. In order to clearly present the process of music-induced emotion flow, we have cut the redundant parts in Fig 1 and adjusted the color and font size of the figure to make it more concise. Corresponding text descriptions have been added to the formulas in the hope of helping readers' understanding.

Revision 2: Music-induced emotion - Emotion flow on Arousal-Valence emotional space - P.5-6 : ------ Correct is "The Arousal-Valence model (A-V model) is currently considered to be the most effective model in the field of emotion computing ... Because the two dimensions cannot be considered to be completely independent at the time of annotation, two cross terms are added to represent the interaction of the two dimensions."(See the details in the manuscript)

**Comment 3：Section 3 must be unified. One sentence cannot be considered as a separate paragraph.**

Response 3: We were really sorry for our careless mistakes. Thank you for your reminder. We've made revised to all non-standard formatting and unified similar paragraphs.

Revision 3: P.9 - para.2 "The network combines..." is unified with the previous paragraph. The first-level title "Model description" is followed by the second-level title "Overview" to describe the overall architecture of the model.

**Comment 4：Authors are suggested to provide more information about the implementation process and used hyper-parameters and datasets.**

Response 4: Thanks for your suggestions. In the new manuscript, we have added an introduction to the DEAM dataset and the Emomusic dataset, given a public availability statement for the dataset, and created a link to access the dataset. In addition, we have added a parameter table to the Experiment Section to show the distribution of the model layers from layer to layer, the size of the parameter quantity, and the output tensor size. A description has been added to the end of the table to provide more hyperparameter information. Finally, we expanded the Model Description Section to include a description of the Time Distributed Layer, and rearranged the composition of Fig 4 to make it more aesthetically pleasing.

Revision 4: Modifications to the dataset ------ Experiment and process - Datasets - paras 1-3:
MediaEval Emotion in Music is a dynamic MER algorithm evaluation. The data set
used in this evaluation comes from the research and development results of Mohammad Soleymani and others ...... In order to unify the size of the model, 58 longer music clips were removed from the DEAM dataset, and we only focused on music clips from 15s to 45s. (Access to the Emomusic dataset: <https://cvml.unige.ch/databases/emoMusic/)> (Access to the DEAM dataset: <https://cvml.unige.ch/databases/DEAM/)> (See the details in the manuscript)

Provide more information about the implementation process and used hyper-parameters ------ Experiment and process - Model architecture - P.18-20(See the details in the manuscript)

Provide more information about the implementation process and used hyper-parameters ------ Model description - Music Informer Layer - Time Distributed Layer - P.13: The Time Distributed Layer is commonly employed for processing time series data and is typically utilized following the CNN Layer, RNN Layer, or LSTM Layer......(See the details in the manuscript)

**Comment 5：There are couple of grammatical and verbal mistakes; the authors have to carefully reread the manuscript.**

Response 5: Thanks for your careful checks. We are sorry for our carelessness. Based on your comments, we invited professional professors to polish the article. We believe that our writing is clear and free of any spelling, grammar, or punctuation errors now.

Revision 5: Every sentence in the manuscript has been polished. (See the details in the manuscript)

**Comment 6：Please discuss the motivation behind the proposed model clearly. Mention what is the priority of the proposed model compared to previous studies**

Response 6: Thanks for your questions. We find that related studies using Attention Mechanisms significantly improved regression accuracy, but the model is not lightweight enough due to the excessive redundancy of features in many musical feature sets. We extend this study from two aspects: 1) Dimensionality reduction features: We use a hierarchical classification approach proposed by Zhou et al. to divide music features into low-level features (physical level) and intermediate features (music theory level). In addition, we integrate the field of computer vision, use the Mel spectrogram to extract features using the Efficient Net Network, and then combine the Librosa library to extract the corresponding features at each time step. The fused features are highly representative, with a dimension of only 45 dimensions, and the experiments in the later stage prove that this extraction method achieves excellent regression accuracy. 2) Upgrade the model: Create an Encoder-decoder architecture, use the Multi-head Attention Mechanism and LSTM Layer to build the Informer model, and add the Layer Normalization Layer, Dropout Layer and Time Distributed Layer to improve the accuracy and generalization ability of the model.

Revision 6: In order to illustrate the superiority of the ENMI model, we added a comparative experiment at the end of the paper, and compared with the RMSE index of the previous MER study, our model performed the best and had better regression performance. We have added it at this location: Discussion - Contrast experiment - P27-28：To further demonstrate the effectiveness of the ENMI model, we compared it with other state-of-the-art dynamic music emotion recognition models using the same evaluation metrics......(See the details in the manuscript)

**Comment 7：The authors are suggested to provide some comments on future directions.**

Response 7: Thanks for your suggestions. We have updated the second paragraph in the "Conclusion and future work" section about the future direction of MER, and I hope that my revision will make the article more complete. The revision includes the following three points: 1) MER needs to improve the accuracy of long time regression prediction; 2) The regression problem in MER mainly studies how the computer thinks ordinary people should have mood swings induced by music, which ignores the problem of differences between people; 3) Based on the solution of the first two points, we can use music to regulate the mood of human beings. For example: music therapy, music information retrieval (MIR).

Revision 7: The complete modification is:"When we evaluated the regression effect of the model, we found that the prediction" accuracy of long-term emotion flow was far less than that of second-level prediction. In the future, we will focus on exploring the deeper relationship between music and emotion on a long-term scale. In addition, MER focuses on the effect that computers think music samples have on people’s emotions, which does not take into account the differences between different individuals. In future work, we will try to build a dataset with more music samples and experimental subjects to study a wider range of patterns. We believe that this work will be meaningful, especially for the development of Music Information Retrieval (MIR) and the practical application of music therapy".

**Comment 8：The format of references is not compatible and the same. Try to carefully update the format of references and use more update references.**

Response 8: We feel sorry for our carelessness. In our latest submission, the manuscript has been completely revised in accordance with the official reference format given by "Plos one". Thanks for your correction.

Revision 8: (See the details in the manuscript)

**Comment 9：The following articles are recommended for use in your manuscript:**

**•Zeinab Khodaverdian, et.al. (2023) An energy aware resource allocation based on combination of CNN and GRU for virtual machine selection. Multimedia Tools and Applications Multimedia Tools and Applications, 2023.**

**https://doi.org/10.1007/s11042-023-16488-2**

**•Sadr H, Nazari Soleimandarabi M (2022) ACNN-TL: attention-based convolutional neural network coupling with transfer learning and contextualized word representation for enhancing the performance of sentiment classification. J Supercomputer 78(7):10149–10175.**

**https://doi.org/10.1007/s11227-021-04208-2**

**•Fatemeh Mohades Deilami, et.al. (2022) Contextualized Multidimensional Personality Recognition using Combination of Deep Neural Network and Ensemble Learning. Neural Process Letters, 54(5), pp. 3811–3828.**

**https://doi.org/10.1007/s11063-022-10787-9**

**• Kalashami, M.P., et.al. (2022) EEG Feature Extraction and Data Augmentation in Emotion Recognition. Computational Intelligence and Neuroscience, 2022, 7028517.**

**https://doi.org/10.1155/2022/7028517**

**•Hossein Sadr, et.al. (2019) Exploring the Efficiency of Topic-Based Models in Computing Semantic Relatedness of Geographic Terms, International journal of web research, Volume 2, Issue 2, Autumn-Winter, December 2019, Pages 23-35.**

**https://doi.org/10.22133/ijwr.2020.225866.1056**

Response 9: We sincerely thank you for providing us with these references. Through reading, we found that these literature are valuable for this research. We have added all the references you have provided to the article and cited them.

Revision 9: These references are cited below: [4][10][33][38][43]

[4] Introduction - para.1: Therefore, it must be noted that building a general system for this aim is very challenging due to the uncertain delineation of human behaviors in the diverse situation [4].

[10]Introduction - para.3: Convolutional Neural Networks (CNNs) is known as one of the significant deep neural networks that has been broadly leveraged for the task of emotional analysis and achieved amazing results [10].

[33]Introduction - p.4: Ultimately, upon identifying limitations of the traditional Informer model within MER tasks, we endeavored to enhance it to better align with the demands of MER [33].

[38]Music-induced emotion - Emotion flow on Arousal-Valence emotional space - para.1: It contains two dimensions: Arousal and valence. Arousal: indicates the intensity of people’s feelings. Valence: indicates the degree of pleasure in the people’s feelings [38].

[43]Model description - Efficient Net Layer - para.3: The use of convolutional operations is to extract local features [43].

**Response to reviewer #2：**

**Comment 1：The proposed solution is thoroughly evaluated on two publicly available benchmark datasets, however a comparison with other studies and systems is missing (several benchmark tests and studies on the same datasets have been published recently, e.g. MediaEval's Emotion in Music challenge, [19] /in the references, and later ones).**

Response 1: Thank you for your valuable suggestions. As you said, although our study did a lot of comparative experiments, it lacked comparisons with previous studies. Therefore, we compared the RMSE index in ENMI (our model) with the excellent models in recent years to highlight the better regression accuracy of our model.

Revision 1: The following models are compared and a brief description of these models can be found in the new manuscript:

RMSE of dynamic music emotion results of different models on the Emomusic dataset: CBSA [29] , RND [48] , BSL [48], TUM [48] , MLR [49] , SVR [49] , GPR [49] , ConvNet D-SVM [50] , CNN-BiLSTM [51]

RMSE of dynamic music emotion results of different models on the DEAM dataset:

BLSTM-RNN [22] , Deep LSTM-RNN [22] , BCRSN [26] , CBSA [29] , ResNets-audioLIME [52] , AC2DConvstat [53] (See the details in the manuscript)

Reference:

[22] Aljanaki A, Yang YH, Soleymani M. Developing a benchmark for emotional
 analysis of music. PloS one. 2017;12(3):e0173392.

[26] Dong Y, Yang X, Zhao X, Li J. Bidirectional convolutional recurrent sparse
 network (BCRSN): An efficient model for music emotion recognition. IEEE
 Transactions on Multimedia. 2019;21(12): 3150-3163.
 doi:10.1109/TMM.2019.2918739.

[29] Zhong ZP, Wang HL, Su GB, Liu L, Pei DM. Music Emotion Recognition Fusion on CNN-BiLSTM and Self-Attention Model [in Chinese]. Computer Engineering and Applications. 2023;59(3): 94-103.

[48] Soleymani M, Aljanaki A, Yang YH, Caro MN, Eyben F, Markov K, et al. Emotional analysis of music: A comparison of methods. In: Proceedings of the 22nd ACM International Conference on Multimedia;2014.1161-1164. doi:10.1145/2647868.2655019.

[49] Soleymani M, Caro MN, Schmidt EM, Sha CY, Yang YH. 1000 Songs Database.
In: Proceedings of ACM International Workshop on Crowdsourcing for
Multimedia;2014.4-7.

[50] Russo M, Kraljevi ́CL, Stella M, Sikora M. Cochleogram-based approach for detecting perceived emotions in music. Information Processing & Management.
2020;57(5): 102270. doi:10.1016/j.ipm.2020.102270.

[51] Du P, Li X, Gao Y. Dynamic Music emotion recognition based on CNN-BiLSTM.
In: 2020 IEEE 5th Information Technology and Mechatronics Engineering
Conference (ITOEC). IEEE;2020.1372-1376. doi:
10.1109/ITOEC49072.2020.9141729.

[52] Chowdhury S, Praher V, Widmer G. Tracing back music emotion predictions to
 sound sources and intuitive perceptual qualities. ArXiv:2106.07787. [Preprint].
 2021.
 [53] Bathigama T, Madushika S. Multi-representational music emotion recognition
 using deep convolution neural networks. Authorea Preprints. 2023.

Location: Discussion - Contrast experiment - P27-28

**Comment 2：The related work review in the Introduction should be updated - the latest cited work on music emotion prediction in continuous scale is [19], which is from 2019 –more recent works (if there are some) needs to be added.**

Response 2: Thank you for pointing out the deficiencies in our work, the work from 2019 onwards is missing from the paper. Therefore, we have studied the research on the application of regression theory to MER tasks in recent years, and summarized and learned from it. In addition, we added research on dynamic music emotion prediction to the “Contrast experiment” to illustrate the effectiveness of our model. Finally, we included the corresponding references.

Revision 2: Introduction - P.3-4. In recent years, researchers have sought to incorporate attention mechanisms into the MER task to further enhance accuracy. ...... These studies highlight the importance of considering two conditions simultaneously to enhance the accuracy of dynamic music emotion recognition: a) A complete and effective set of musical features. ...... b) Models with perfect long sequence prediction capabilities. ......To our knowledge, there are limited studies in Music Emotion Recognition (MER) employing regression methods to simultaneously investigate dynamic emotional prediction. ......(See the details in the manuscript)

**Comment 3：Please also clarify the statement (p.2): " Although good progress has been made, *the models are unstable*" I do not agree that the classical probability based machine learning models (such as HMM or SVM) are unstable for MER tasks.**

**Mentioning a *RReliefF* method in the related work review (p2) seems be redundant.**

Response 3: Thank you for your valuable suggestions. We reviewed the literature in the MER field on the use of traditional machine learning algorithms such as SVM and HMM. As you said, traditional machine learning models are stable when used in MER tasks and have the advantages of strong generalization ability. Its place in the field of MER research cannot be ignored, and we are deeply sorry for this mistake. By comparing the performance of traditional machine learning models with those of deep learning models in recent years, we find that deep learning is more capable of processing data than traditional machine learning, and has advantages in both classification and predictive regression problems. We have revised the statements in the manuscript to make them more rigorous. In addition, we also have made adjustments to the redundant parts.

Revision 3: P.2 line 6-10 from the end. "Despite significant progress, these models often struggle with emotion recognition accuracy. Yang et al. were the first to employ regression methods in MER, directly predicting arousal and valence values for each music sample, thus circumventing the inherent ambiguity in traditional classification methods. "

**Comment 4：This question arises from the first comment: Did you apply the same training/testing methodology as is recommended for the DEAM dataset? [15,19]. This is necessary in order to make a comparison with the other solutions proposed recently (if they make evaluation on the same data).**

Response 4: Our research follows the same training and testing approach recommended by the DEAM dataset. The differences lie in the following aspects:

1) We did not utilize the benchmark feature set from the dataset for our research. Instead, we extracted features manually in parallel from two temporal scales. This approach helped us avoid the inclusion of redundant features in the feature set and ensured compatibility with the input of our subsequent learning models.

2) While our evaluation metrics partially overlap with the recommended ones, the recommended metrics include RMSE and CCC, whereas ours include RMSE and MAE. CCC focuses on predicting changes in direction and shape trajectories, while the latter prioritizes interpretability and directly reflects the absolute magnitude of errors. Therefore, we believe these studies can be compared based on the RMSE evaluation metric.

Revision 4: (See revision 1)

**Comment 5：Please clarify, why you decided to use mean absolute error (MAE) as an additional metric to RMSE. Cross-Correlation is more common for time series comparison (cross-correlation based metrics were also use in the Mediaeval Emotion in Music benchmark [15].**

Response 5: Thanks for your suggestions. By comparing the studies on MER in the last decade, the RMSE metric has been used most often due to its high interpretability, but since RMSE is sensitive to outliers, this can easily be dominated by large errors. We find that MAE can just compensate for this shortcoming, MAE is robust to outliers and has good interpretability. Cross-correlation, on the other hand, is still sensitive to outliers, especially to the absolute magnitude of the amplitude, which leads to unrepresentative evaluation of the data in a dataset with large fluctuations in sentiment. In addition, Cross-Correlation is more suitable for considering the shape and phase information of signals in areas such as signal processing and pattern matching. For regression problems, MAE is more applicable.

Revision 5: We have updated the section on indicator descriptions to make our intentions clearer to readers. We have corrected it at this location: P.18 - para.2 - line 3-6 ---correct is: Mean Squared Error (MSE) served as the loss function, while Root Mean Square Error (RMSE) was adopted as the primary metric. Recognizing the necessity of accountability for each sample in the test set and the susceptibility of RMSE to outliers and large errors, Mean Absolute Error (MAE) was introduced as an additional metric.

**Comment 6：P. 11, fig 5 - I do not understand why mp3 files are converted to flac format, thus, from lossy to lossless one. If there is already lossy compression, transformation to lossless format has no benefit. In addition, flac format is not suitable for data manipulation. If the reason was to have a format for easy access of data, raw or wav format suits better than flac. I recommend, withdraw information about flac from the text. In figure, replace the label *.flac with "raw audio".**

Response 6: Based on your suggestion, we realized that it is unscientific to process mp3 format into flac format for MER tasks, so we converted all mp3 files in the dataset into lossless and uncompressed wave files, which ensures that the audio is lossless and easy to do post-processing in python. In addition, we note that the following studies have done similar treatment: (Zhong Zhipeng, Wang Hailong, Su Guibin, Liu Lin, Pei Dongmei. Music Emotion Recognition Fusion on CNN-BiLSTM and Self-Attention Model[J]. Computer Engineering and Applications, 2023, 59(3): 94-103.)

Revision 6: We have updated Fig 5. The relevant narrative in the text has also been corrected: P.15 - line 5-6 from the end, ---correct is "Consequently, after converting all MP3 files in the dataset into lossless and uncompressed WAV files, we applied Gaussian white noise addition and a specific range of time stretching processing to augment the data.".

**Comment 7：p. 18-19, Tables 4,5: I recommend to highlight (e.g. by bold font) the best results for all columns (e.g. 4 top ranked values either MSE or MAE) to better visualize the most salient features.**

Response 7: We think this is an excellent suggestion. This makes it easier to highlight the best performance metrics in the experimental results.

Revision 7: We've bolded the top 3-4 indicators for each column in the table, and changed the format of the headers. At the same time, we've added a note to this location: P.26 - para.2 - line 2-3.

**Comment 8：p.2 - "*invisible* Markov model" - correct is "hidden Markov model"**

Response 8: Thank you for pointing out the inappropriate use of scientific vocabulary in our article. We carefully read the paper on the hidden Markov model and revised it according to your comments.

Revision 8: We have corrected it at this location: P.2 - para.2 - line 8.

**Comment 9：p.12 - " .. spectral correlation features (including MFCC ... " - the word 'correlation' is confusing. Instead, use simply "spectral features".**

Response 9: Thank you for pointing out the problem of our inaccurate wording. We have removed it from the article. In addition, we have corrected all errors related to it.

Revision 9: We have corrected it at these location: P.17 - para.1.

**Comment 10：Regarding the name "non-time series": I do not think that Mel spectrogram features are non-time series, because one dimension of the spectrogram is time, just there is a different time-scale as in the case of selected features extracted by Librosa lib. I recommend to use a name for the Mel-spectrogram features other than "non-time".**

Response 10: Thanks for your suggestions. We carefully studied your comments and revised all "non-time series features" in the manuscript to "the Mel-spectrogram features". We are clear that one dimension of the spectrogram is time. Our previous consideration for naming lies in the fact that the 1×n tensor formed after the spectrogram is processed by Efficient Net no longer holds the meaning of time. For the 1×n tensor, n represents the number of features, which means that for each sample file there are n features, but these features do not correspond to time frames. So this is different from the features extracted on each time frame using the Librosa tool. However, our statement may tend to confuse the reader, so we fully accept your suggestion.

Revision 10: We have corrected them in our new manuscript.

**Comment 11：p.13 - What difference is between "cycles"and "epochs"? Please, unify this.**

Response 11: Thank you for pointing out the mistakes in our writing, and we're sorry for that. What we want to express is “epochs” in deep learning. So we modified "cycles" to "epochs".

Revision 11: We have corrected it at this location: P.18 - para.2.

**Comment 12：p. 14 - Fig 6 capture does not match with graphs in Figure 6.**

Response 12: We feel sorry that we did not provide enough information in Fig 6. Our model needs 100 epochs to converge, and the first 55 epochs have indeed converged to a low level. But after 55 epochs, the model continues to converge until about 100 epochs. The convergence curve after 55 epochs is not represented in the graph, so we modified Fig 6 to make the convergence process clearer.

Revision 12: We have updated Fig 6. It shows the changes in the loss curve from 55 epochs to 100 epochs to illustrate that the model needs 100 epochs to converge.

**Comment 13：p.6, 7 - Please cite references for both the Efficient Net model and Music Informer.**

Response 13 : Thanks for your suggestions. We have cited references at the location you specified,

which would be more in line with the regulations.

Revision 13 : We have added it at these location: P.8 - line 2 from the end, P.10 - para.2.

**Comment 14：Please provide a source, from which the DEAM and Emomusic datasets can be accessed.**

Response 14: Thanks for your suggestions. We fully take your comments on board as they make it easier for readers to access the dataset.

Revision 14 : We have provided the source of the datasets at this location: P.15 - para.2.
