# Supplementary material for: Music-induced emotion flow modeling by ENMI Network
Source: PLoS One. 2024 Oct 21;19(10):e0297712. doi: 10.1371/journal.pone.0297712 (PMC11493256; doi:10.1371/journal.pone.0297712)
Supplement: Attachment — Submitted filename: Response to Reviewers.docx [file pone.0297712.s007.docx]

**PONE-D-24-01232**

**Music-induced emotion flow modeling by ENMI Network**

**Dear editor:**

**On behalf of all authors, I would like to express our sincere gratitude for your time and the valuable feedback provided by the reviewers regarding our manuscript. After receiving your review comments, we have revised the manuscript with high quality as fast as possible. We hope that this revision can meet the publication standard of your journal.**

**We apologize and have reflected deeply on the issue mentioned by reviewer 2 in the last revision regarding the short and inadequate response to the letter. This time, we have provided a more detailed revision and response, indicating the exact location of all changes. We have also responded to reviewer 3's comments about the methodology of the emotional model, the data preprocessing, and some ambiguous expressions. We have ensured that we will pay more attention to them in our future writing.**

**Thank you again for recognizing our manuscript and giving us a second chance to revise it. Please feel free to contact us with any other questions regarding the revision of the manuscript, and we will be sure to cooperate fully.**

**Yours sincerely**

**Lead author: Yunrui Shang**

**E-mail:** [**m15031528057@163.com**](mailto:m15031528057@163.com)

**Corresponding author: Yinhua Liu**

**E-mail:** [**liuyinhua@qdu.edu.cn**](mailto:liuyinhua@qdu.edu.cn)

**Response to reviewer #2：**

**Dear reviewer #2,**

**I am deeply honored to have received your review comments, and we are also very grateful for the opportunity to revise our manuscript.**

**In the last version of the modification our reply was too simple and did not indicate the location of the modification clearly enough, we deeply reflect on this and sincerely apologize to you. In this revision, we have carefully studied your suggestions and replied to each of them, at the same time, in the version of Revised Manuscript with Track Changes, we have shown you the specific location of each of your comments.**

**Thank you again for your time and for recognizing the manuscript, we have gained a lot from your comments. Please feel free to contact us if you have additional comments on this revision.**

**Yours sincerely**

**Lead author: Yunrui Shang**

**Corresponding author: Yinhua Liu**

**Comment 1：The proposed solution is thoroughly evaluated on two publicly available benchmark datasets, however a comparison with other studies and systems is missing (several benchmark tests and studies on the same datasets have been published recently, e.g. MediaEval's Emotion in Music challenge, [19] /in the references, and later ones).**

Response: Thank you for your valuable suggestions. As you said, although our study did a lot of comparative experiments, it lacked comparisons with previous studies. We have fully recognized the strengths of the current study that can be highlighted by comparing results with other research methods. Therefore, we compared the RMSE index in ENMI (our model) with the excellent models in recent years to highlight the better regression accuracy of our model. The following models are compared and a brief description of these models can be found in the new manuscript:

RMSE of dynamic music emotion results of different models on the Emomusic dataset: **CBSA** [29] , **RND** [48] , **BSL** [48], **TUM** [48] , **MLR** [49] , **SVR** [49] , **GPR** [49] , **ConvNet D-SVM** [50] , **CNN-BiLSTM** [51]

RMSE of dynamic music emotion results of different models on the DEAM dataset:

**BLSTM-RNN** [22] , **Deep LSTM-RNN** [22] , **BCRSN** [26] , **CBSA** [29] , **ResNets-audioLIME** [52] , **AC2DConvstat** [53]

We have added “Contrast experiment” with the following modifications:

**Contrast experiment**

To further validate the effectiveness of the ENMI model, we compared it with other leading dynamic music emotion recognition models using the same evaluation metrics. As shown in Tables 7 and 8, the ENMI model exhibits the lowest RMSE across any two dimensions of the two datasets, indicating its superior regression performance. Below is a description of the models considered in the comparison for dynamic music emotion recognition using regression methods:
**BLSTM-RNN, Deep LSTM-RNN [22]:** These models are recognized as the two leading methods in MediaEval 2015. Baseline features are derived from the DEAM dataset, consisting of 260 low-level features extracted using the Opensmile Tool. Through PCA whitening (retaining 99% variance), the original audio signals, initially with 2646 dimensions per frame (60ms), are reduced to 43. Subsequently, after dimensionality reduction, audio signals within each 0.5s segment are sequentially fed into these models frame by frame.
**RND, BSL, TUM [48]:** The Technische Universit ̈at M ̈unchen (TUM) team’s approach is based on supra-segmental features derived from statistical functions applied to framewise low-level descriptors (LLDs) across either one-second segments or entire songs. (Abbreviations: RND for random level, BSL for Baseline)

**MLR, SVR, GPR [49]:** These three models serve as the dataset baseline, while Aizu University and Utrecht University employ their respective recognition methods fortraining and evaluating the EmoMusic dataset.

**ConvNet D-SVM [50]:** This study explores the integration of contextual information into emotional analysis by utilizing dilated convolutions to expand the receptive fields of network layers, and then feeding this information into an SVM regression model. **CNN-BiLSTM [51]:** Dynamic music emotion recognition is achieved by integrating CNNs and BiLSTM Networks.
**ResNets-audioLIME [52]:** The source separation interpreter and the residual network are integrated to analyze both intermediate perceptual features and spectrogram features.

**AC2DConvstat [53]:** Employing a two-dimensional CNN model, both in audio and computational domains, to analyze the audio feature representations formed from a fusion of raw audio, audio signals, and spectrograms. (P.28-P.29)

Reference:

[22] Aljanaki A, Yang YH, Soleymani M. Developing a benchmark for emotional analysis of music. PloS one. 2017;12(3): e0173392.

[26] Dong Y, Yang X, Zhao X, Li J. Bidirectional convolutional recurrent sparse network (BCRSN): An efficient model for music emotion recognition. IEEE Transactions on Multimedia. 2019;21(12): 3150-3163. doi:10.1109/TMM.2019.2918739.

[29] Zhong ZP, Wang HL, Su GB, Liu L, Pei DM. Music Emotion Recognition Fusion on CNN-BiLSTM and Self-Attention Model [in Chinese]. Computer Engineering and Applications. 2023;59(3): 94-103.

[48] Soleymani M, Aljanaki A, Yang YH, Caro MN, Eyben F, Markov K, et al. Emotional analysis of music: A comparison of methods. In: Proceedings of the 22nd ACM International Conference on Multimedia;2014.1161-1164. doi:10.1145/2647868.2655019.

[49] Soleymani M, Caro MN, Schmidt EM, Sha CY, Yang YH. 1000 Songs Database.
In: Proceedings of ACM International Workshop on Crowdsourcing for Multimedia;2014.4-7.

[50] Russo M, Kraljevi ́CL, Stella M, Sikora M. Cochleogram-based approach for detecting perceived emotions in music. Information Processing & Management.
2020;57(5): 102270. doi:10.1016/j.ipm.2020.102270.

[51] Du P, Li X, Gao Y. Dynamic Music emotion recognition based on CNN-BiLSTM. In: 2020 IEEE 5th Information Technology and Mechatronics Engineering Conference (ITOEC). IEEE;2020.1372-1376. doi: 10.1109/ITOEC49072.2020.9141729.

[52] Chowdhury S, Praher V, Widmer G. Tracing back music emotion predictions to
sound sources and intuitive perceptual qualities. ArXiv:2106.07787. [Preprint].2021.
[53] Bathigama T, Madushika S. Multi-representational music emotion recognition
using deep convolution neural networks. Authorea Preprints. 2023.

**Comment 2：The related work review in the Introduction should be updated - the latest cited work on music emotion prediction in continuous scale is [19], which is from 2019 –more recent works (if there are some) needs to be added.**

Response: Thank you for pointing out the deficiencies in our work, the work from 2019 onwards is missing from the paper. The Introdution section of a good article should contain the latest research methods in the field in recent years. Keeping abreast of the latest research is good for us to learn, and it will be more convincing in the process of doing comparative experiments. We apologize for the omission of this section. Related research in recent years reveals that attention mechanisms and LSTM networks have been widely used in dynamic music emotion recognition with good results. This is why we borrowed the main body of the Informer model, focusing on adding LSTM networks and retaining the multi-head attention mechanism. We have studied the research on the application of regression theory to MER tasks in recent years, and summarized and learned from it. In addition, we added research on dynamic music emotion prediction to the “Contrast experiment” to illustrate the effectiveness of our model. Finally, we included the corresponding references. The modifications are as follows:

In recent years, researchers have sought to incorporate attention mechanisms into the MER task to further enhance accuracy. Sanga et al. [27] markedly enhanced the predictive performance of arousal and valence by utilizing attention mechanisms in LSTM. Huang et al. [28] proposed the ADFF method for music emotion recognition. This method employs an end-to-end attention-based deep feature fusion mechanism, utilizing logarithmic Mel spectrograms as input to effectively capture salient features related to emotion in music clips. Zhong et al. [ 29] introduced the CBSA model, which integrates CNN, BILSTM, and self-attention mechanisms. This model emphasizes local key features of music emotion, the serialization of music emotion information from local features, and global key points of music emotion. Improved regression results were observed in both the Emomusic dataset and the DEAM dataset. These studies highlight the importance of considering two conditions simultaneously to enhance the accuracy of dynamic music emotion recognition:

a) A complete and effective set of musical features.

At present, music feature sets often exhibit feature redundancy, and the incorporation of excessive features into the model may result in overfitting. This phenomenon causes the model to memorize the noise in the data during training, thereby limiting its generalizability.

b) Models with perfect long sequence prediction capabilities.

The current model still has limitations in handling long sequences. Additionally, it does not consider multi-scale temporal feature learning, resulting in suboptimal performance and efficiency. (P.3-P.4)

**Comment 3：Please also clarify the statement (p.2): " Although good progress has been made, *the models are unstable*" I do not agree that the classical probability based machine learning models (such as HMM or SVM) are unstable for MER tasks. Mentioning a *RReliefF* method in the related work review (p2) seems be redundant.**

Response: Thank you for your valuable suggestions. We reviewed the literature in the MER field on the use of traditional machine learning algorithms such as SVM and HMM. As you said, traditional machine learning models are stable when used in MER tasks and have the advantages of strong generalization ability. Its place in the field of MER research cannot be ignored, and we are deeply sorry for this mistake. By comparing the performance of traditional machine learning models with those of deep learning models in recent years, we find that deep learning is more capable of processing data than traditional machine learning, and has advantages in both classification and predictive regression problems. We have revised the statements in the manuscript to make them more rigorous. In addition, we also have made adjustments to the redundant parts: "Despite significant progress, these models often struggle with emotion recognition accuracy. Yang et al. were the first to employ regression methods in MER, directly predicting arousal and valence values for each music sample, thus circumventing the inherent ambiguity in traditional classification methods. " (P.2-Para.2)

**Comment 4：This question arises from the first comment: Did you apply the same training/testing methodology as is recommended for the DEAM dataset? [15,19]. This is necessary in order to make a comparison with the other solutions proposed recently (if they make evaluation on the same data).**

Response: Thanks for your comments. Our research follows the same training and testing approach recommended by the DEAM dataset. The differences lie in the following aspects:

1) We did not utilize the benchmark feature set from the dataset for our research. Instead, we extracted features manually in parallel from two temporal scales. This approach helped us avoid the inclusion of redundant features in the feature set and ensured compatibility with the input of our subsequent learning models.

2) While our evaluation metrics partially overlap with the recommended ones, the recommended metrics include RMSE and CCC, whereas ours include RMSE and MAE. CCC focuses on predicting changes in direction and shape trajectories, while the latter prioritizes interpretability and directly reflects the absolute magnitude of errors. Therefore, we believe these studies can be compared based on the RMSE evaluation metric.

**Comment 5：Please clarify, why you decided to use mean absolute error (MAE) as an additional metric to RMSE. Cross-Correlation is more common for time series comparison (cross-correlation based metrics were also use in the Mediaeval Emotion in Music benchmark [15].**

Response: Thanks for your suggestions. By comparing the studies on MER in the last decade, the RMSE metric has been used most often due to its high interpretability, but since RMSE is sensitive to outliers, this can easily be dominated by large errors. We find that MAE can just compensate for this shortcoming, MAE is robust to outliers and has good interpretability. Cross-correlation, on the other hand, is still sensitive to outliers, especially to the absolute magnitude of the amplitude, which leads to unrepresentative evaluation of the data in a dataset with large fluctuations in sentiment. In addition, Cross-Correlation is more suitable for considering the shape and phase information of signals in areas such as signal processing and pattern matching. For regression problems, MAE is more applicable. We have updated the section on indicator descriptions to make our intentions clearer to readers. We have corrected it: Mean Squared Error (MSE) served as the loss function, while Root Mean Square Error (RMSE) was adopted as the primary metric. Recognizing the necessity of accountability for each sample in the test set and the susceptibility of RMSE to outliers and large errors, Mean Absolute Error (MAE) was introduced as an additional metric.

(P.18-Para.2)

**Comment 6：P. 11, fig 5 - I do not understand why mp3 files are converted to flac format, thus, from lossy to lossless one. If there is already lossy compression, transformation to lossless format has no benefit. In addition, flac format is not suitable for data manipulation. If the reason was to have a format for easy access of data, raw or wav format suits better than flac. I recommend, withdraw information about flac from the text. In figure, replace the label *.flac with "raw audio".**

Response: Thanks for your suggestions. Based on your suggestion, we realized that it is unscientific to process mp3 format into flac format for MER tasks, so we converted all mp3 files in the dataset into lossless and uncompressed wave files, which ensures that the audio is lossless and easy to do post-processing in python. In addition, we note that the following studies have done similar treatment: (Zhong Zhipeng, Wang Hailong, Su Guibin, Liu Lin, Pei Dongmei. Music Emotion Recognition Fusion on CNN-BiLSTM and Self-Attention Model[J]. Computer Engineering and Applications, 2023, 59(3): 94-103.). We have updated Fig 5. The relevant narrative in the manuscript has also been corrected "Consequently, after converting all MP3 files in the dataset into lossless and uncompressed WAV files, we applied Gaussian white noise addition and a specific range of time stretching processing to augment the data.". (P.17-Para.2/ P.17-Fig 5)

**Comment 7：p. 18-19, Tables 4,5: I recommend to highlight (e.g. by bold font) the best results for all columns (e.g. 4 top ranked values either MSE or MAE) to better visualize the most salient features.**

Response: We think this is an excellent suggestion. This makes the optimal results in each group more intuitive and easier for the reader to read. We've bolded the top 3-4 indicators for each column in the table, and changed the format of the headers. At the same time, we've added a note: (3-4 top-ranked values either MSE or MAE are highlighted in bold), For the newly added parts “Contrast experiment”, our model is optimized for comparison with other models. Based on your suggestion, we have also bolded the data that performs optimally. Thanks for your suggestion again and your suggestion is very helpful! (P.27-Table 5 and 6)

**Comment 8：p.2 - "*invisible* Markov model" - correct is "hidden Markov model"**

Response: Thank you for pointing out the inappropriate use of scientific vocabulary in our article. For some outstanding models or methods, they belong to the proprietary vocabulary and cannot be changed by other authors. We carefully read the paper on the hidden Markov model and revised it according to your comments. The modified version is: Previous studies have utilized traditional machine learning models such as Decision Trees [6], Hidden Markov Models (HMM) [7], and Support Vector Machines (SVM) [8] for speech emotion recognition. Thank you for pointing out our mistakes in the writing process, we will strengthen our learning to avoid such mistakes from happening again. (P.2-Para.2)

**Comment 9：p.12 - " .. spectral correlation features (including MFCC ... " - the word 'correlation' is confusing. Instead, use simply "spectral features".**

Response: Thank you for pointing out the problem of our inaccurate wording. We have removed it from the article. In addition, we found that the description of the "Music Feature Extraction" section was vague and redundant, so we have rewritten the section. We hope that this revision will result in a more concise description of the language. If there are still problems after the revision, we would like to get more comments from you. Here are the modifications: Table 1 illustrates the selection of features at various levels of music analysis. Spectral features, including MFCC, spectral centroid, spectral bandwidth, spectral flatness, spectral contrast, and roughness, along with time domain features like zero crossings, constitute the physical level of music, totaling 19 dimensions. Chromaticity characteristics, tempogram, and tonal centroid, totaling 14 dimensions, are regarded as characteristics of music theory. To address the limitations of feature extraction using the Librosa Library and better integrate different feature types, deep learning techniques are employed to extract music features from Mel spectrograms, referred to as image features. Considering the complexity of cognitive features, emotional characteristics at higher levels are directly studied as observational measures. (P.10-para.2)

**Comment 10：Regarding the name "non-time series": I do not think that Mel spectrogram features are non-time series, because one dimension of the spectrogram is time, just there is a different time-scale as in the case of selected features extracted by Librosa lib. I recommend to use a name for the Mel-spectrogram features other than "non-time".**

Response: We couldn't agree more with your suggestion. As you said, the spectrogram contains the time dimension. In our task, time series features and non-time series features are formulated in a way that favors feature extraction. In time series features, we require that each time frame corresponds to a musical feature vector. However, in the case of non-time series features, the extracted features are given in terms of computer vision and are a 1×10 tensor, where the "10" in the vector does not correspond to a time frame, but is the result of the Efficient Net output. However, our use of "non-time" nomenclature may be misleading to the reader. So we have renamed all "non-time" to "Mel-spectrogram features", this name was chosen because the features extracted from Efficient Net are derived from the Mel spectrograms. We believe this change will be accurate. Thank you again for your comments.

**Comment 11：p.13 - What difference is between** **"cycles" and "epochs"? Please, unify this.**

Response: Thank you for pointing out the mistakes in our writing, and we're sorry for that. There is no difference between "cycles" and "epochs". What we want to express is “epochs” in deep learning. So we modified "cycles" to "epochs". The modified version is: ReduceLROnPlateau function dynamically adjusted the learning rate by monitoring validation loss; if the loss didn’t improve for 5 consecutive epochs, the learning rate was halved, with a minimum value of 1e-8. EarlyStopping ensured that if the loss didn’t improve for 10 consecutive epochs, training was terminated, and the best weights were restored to prevent overfitting. Thank you again for pointing out the problems with our writing, and we will definitely pay more attention to the accuracy of the words we use in our future writing. (P.18-Para.2)

**Comment 12：p. 14 - Fig 6 capture does not match with graphs in Figure 6.**

Response: We feel sorry that we did not provide enough information in Fig 6. Our model needs 100 epochs to converge, and the first 55 epochs have indeed converged to a low level. But after 55 epochs, the model continues to converge until about 100 epochs. The convergence curve after 55 epochs is not represented in the graph, so we modified Fig 6 to make the convergence process clearer. We have updated Fig 6. It shows the changes in the loss curve from 55 epochs to 100 epochs to illustrate that the model needs 100 epochs to converge. Thanks again for pointing out our shortcomings, and please let us know if there is still something that is not clearly represented in the figure.

(P.21-Fig 6)

**Comment 13：p.6, 7 - Please cite references for both the Efficient Net model and Music Informer.**

Response: Thank you for pointing out the citation flaws. When borrowing from others, it is important to cite the appropriate literature resources so as to comply with the normative nature of scientific research. We incorrectly assume that results located in the principles section of a manuscript do not need to be cited. Following your suggestion, we have labeled the references to the Efficient Net model and the Informer model in the appropriate places:

31). Tan M, Le Q. Efficientnet: Rethinking model scaling for convolutional neural networks. In: International Conference on Machine Learning;2019.6105-6114. doi:10.48550.
32). Zhou H, Zhang S, Peng J, Zhang S, Li J, Xiong H, et al. Informer: Beyond efficient transformer for long sequence time-series forecasting. In: Proceedings of the AAAI Conference on Artificial Intelligence. 2021;35(12): 11106-11115. doi:10.48550.

We believe that this revision is in line with the norms and thank you again for your guidance. (P.11-Para.1/P.12-Para.1)

**Comment 14：Please provide a source, from which the DEAM and Emomusic datasets can be accessed.**

Response：Thanks for your suggestions. We fully take your comments on board as they make it easier for readers to access the dataset. We will take care to cite the source of the dataset in future manuscripts to facilitate its study and use by other researchers and

we have provided the source of the datasets: (Access to the Emomusic dataset: <https://cvml.unige.ch/databases/emoMusic/>)(Access to the DEAM dataset: <https://cvml.unige.ch/databases/DEAM/>). Thanks again for your valuable suggestions.

(P.15-Para.4)

**Response to reviewer #3：**

**Dear Dr.Li,**

**It is my honor to receive your review comments and we appreciate the opportunity to revise the manuscript. Your nine comments contain suggestions in three areas: methodology of sentiment modeling, data preprocessing, and some presentation issues with ambiguity.**

**Your comments make us realize that you are rigorous and serious about your research. We have fully studied your comments and revised the errors and inappropriateness that appeared in the manuscript, and have listed our responses and revisions in detail. Please feel free to contact us with any additional questions regarding the following revisions.**

**Thank you for reviewing and recognizing our manuscript.**

**Yours sincerely**

**Lead author: Yunrui Shang**

**Corresponding author: Yinhua Liu**

1- **I found the wording of the 1st contribution of the paper (top of page 5) to be somewhat fuzzy. It would be advised to replace some vague wording like "creatively explored" with more specific sentences.**

Response：Thank you for your valuable suggestions. We have realized that some of the words used in the description of the first contribution in the manuscript are ambiguous. Based on your comments, we have modified the description of the first contribution: "Emotion flow" is considered to be the result of music-induced changes in a person's emotion. It is a dynamic response with musical elements and emotional history as inputs, so it can be modeled as a multivariate time series problem. This contribution is an application of the definition of emotion flow proposed by the American psychologist Csikszentmihalyi in 1990. (Specific reference: Csikszentmihalyi M, Csikzentmihaly M. Flow: The psychology of optimal experience. New York: Harper & Row, 1990. doi:10.5860/choice.28-0597.) We have removed ambiguous words such as "creatively explored", and we will pay more attention to the accuracy and rigor of the words we use in our future writing to avoid ambiguity. (P5-para.1)

2- **Section "Emotion flow on Arousal-Valence emotional space": it is not clear to me what is the "music element vector" ∆y in Equation (1). The Pearson correlation being computed between ∆x and ∆y implies that both vectors have the same dimension, and ∆x is assumed to be 2D (vector in the arousal/valence space). It would be required to provide a more detailed definition of ∆y to make the understanding of Equation (1) easier.**

Response：Thank you for your suggestion. As you said, the definition of the music element vector ∆y is vague. Let me now explain in more detail the subsection " Emotion flow on Arousal-Valence emotional space".

This section was set up to follow the theme of "emotion flow". This is because the term has not been used in recent accounts of dynamic music emotion research to describe the effects of music on human emotions. We use this term to highlight this detailed process and to pave the way for the use of time series modeling to address this issue. The process of "emotion flow" formation consists of two parts, the first of which I will highlight in this response and the second in the next.

The first process of the formation of "emotion flow" is to describe that when a person is first exposed to music, the correlation between the musical characteristics and the change of emotion is almost zero; in other words, it is a process that a person goes from hearing music - transmitting music signals to the cerebral cortex - the cerebral cortex processing the music signals - and mapping the emotion. Currently, there is no study with a theoretical system about modeling the mechanism of music-induced emotion. What we can determine, however, is that the process is time consuming and that we have difficulty predicting emotional changes (responses) when music signals are used as (inputs) over short periods of time. This leads to the formula given in the manuscript.

In response to your comments, the definition of the music element vector ∆y is indeed missing in the manuscript, and we use the 2-norm difference of the music feature vector per unit time to specifically define the magnitude of ∆y in order to do a Pearson correlation calculation with the amount of change in the emotion labeling values. The specific modifications are as follows: (P.6)

3- **Subsection "Emotion flow on Arousal-Valence emotional space": similarly to the previous point, it is not so clear to me what** $\boldsymbol{f}$**/**$\boldsymbol{f}_{\boldsymbol{A}}$ **and** $\boldsymbol{g}$**/**$\boldsymbol{g}_{\boldsymbol{A}}$ **represent in Equation (2). f and g are introduced as the "laws of the arousal dimension and the valence dimension", which is quite fuzzy to me.** $\boldsymbol{f}_{\boldsymbol{A}}$ **and** $\boldsymbol{g}_{\boldsymbol{A}}$ **are not introduced at all. It is better to be more specific regarding these definitions as Equation (2) is currently difficult to understand.**

Response：We appreciate your keen observation regarding the issues with our formula. Indeed, some parameters within the formula were not adequately explained in the manuscript, which led to confusion around Equation (2), and for that, we apologize. In this response, I will detail the second phase of Emotion Flow formation.

Following the initial unstable annotation part, human emotions become increasingly susceptible to musical induction. This phase can be conceptualized as a system where music acts as the input and emotion as the output, and the mechanism by which the human brain perceives music has stabilized. At this stage, changes in human emotion (output) are primarily determined by the music being played (input). However, due to the lack of systematic research on the brain's response to music, we can only envisage this process as a black box—a fuzzy system. This is precisely what the deep learning algorithms aim to learn: the rules $f_{A}(t)$ and $g_{A}(t)$ governing the interaction between music and emotion. Moreover, during the calibration of emotional labels, we were uncertain about whether arousal and valence dimensions would influence each other. Therefore, their interrelation should also be taken into account, i.e., the effect of valence on arousal, $f_{AV}(V(t))$, and the effect of arousal on valence, $g_{VA}(A(t))$.

After careful consideration of your comment, we have revised this section to ensure greater rigor and clarity. The modifications are as follows: (P.7)

where $A_{0}$ represents the initial arousal value, A(t) represents the updated arousal value, $V_{0}$ represents the initial valence value, V(t) represents the updated valence value, $f_{A}(t)$ and $g_{A}(t)$ represent the amount of emotion change in the music-induced arousal dimension and the valence dimension, respectively, which is the part of the deep learning model that needs to be learned. In addition, $f_{AV}(V(t))$ and $g_{VA}(A(t))$ represent the effects of the two emotions on each other, and this interaction is also important for human emotional change.

4- **What does "Ascension" in Figure 3 mean? Since it is currently not described in the manuscript, it would be better to add an explanation there.**

Response：Thank you for your suggestions. You have made a very valuable suggestion. It is true that the word "Ascension" is not explained in detail in the manuscript. And we have realized that the use of this word is inappropriate. Due to the data format, the Mel spectrogram features extracted using Efficient Net are not directly used as input to the simplified Informer Layer. For each music sample, Efficient Net's output format for this work was a tensor of 1×10 size. In order to better integrate with time-series data from manual feature extraction, a temporal dimension needs to be added to that tensor, i.e., each piece of music has 10 constant-value sequence features that do not change over time. In response to your suggestion, we have modified the original text by replacing the word "Ascension" with "dimension increase", and added a note about it after the title of Fig 3, which reads as follows: "dimension increase" adds a temporal dimension to the original tensor, which is used for fusion with time series features by transforming them into constant value series features. (P.9-Fig 3)

5- **Subsection "Informer layer": the authors justify their decision to add the song ID in the Time Feature Embedding with the sentence "Considering that the time series between different songs are much less relevant than the same song," which is quite unclear to me. Additionally, this choice raises the question of how the model is used at inference time on unseen data when the song ID is not known (e.g. for a song that doesn't belong to the DEAM or Emomusic datasets, without any ID). A more detailed and clearer explanation of these two points is necessary in my opinion. It could also be interesting to report the results of the proposed approach without using the song ID in the embedding.**

Response：Thank you for your valuable suggestion, you have considered the problem very carefully and thoroughly. The time embedding layer included in the Informer model cannot be used directly in this task. Because the time series in this task does not have the periodicity of "year-month-week-day-hour-minute-second". Therefore, we need to find another way to model the temporal characteristics when building the model. Because the data splicing of this task is made by splicing different songs by the first and last of the time dimension, and the sample length of each song is fixed for both datasets, which is 15s-45s, we decided to tell the model to go to the next training cycle after each Id is finished. This results in an "ID-second" cycle characteristic. In your suggestion, you mentioned that the prediction of a song that does not belong to one of the two base datasets cannot be defined in this way. We fully agree with this. Especially when the model is trained with samples of different sample lengths, it is obvious that this way of defining the time period is wrong. The definition of the time period in the manuscript is only meant to serve datasets with specific sample lengths, and we are already acutely aware of the limitations of this way of defining the time period. As you commented, the model remains valid without embedding song IDs. We have removed the reference to defining song IDs from the manuscript and removed the section of Fig 4 on IDs. We hope our modifications are successful and thank you again for your valuable comments. (P.15-Fig 4)

6- **Subsection "Preprocessing": the first paragraph describing the datasets that were used in the study is not so clear, more specifically regarding to what the "new dataset [introduced] to achieve balance" refers to. The abstract mentions an "augmented version" of the Emomusic dataset, but the wording used in this Subsection seems to imply this augmented version is the DEAM dataset. It would be indicated to clarify this part and how each of the 3 datasets was used (since the experiments were apparently carried out only on Emomusic and DEAM).**

Response：Thank you for your comments. The description of the Augmented dataset in the subsection "Preprocessing" seems to be unclear. Let me now clarify the original intent of the article: the Emomusic dataset contains 744 pieces of music, the DEAM dataset contains 1744 pieces of music, and it should be noted that the samples in the Emomusic dataset are a subset of the DEAM dataset. It is necessary to understand whether the newly added music samples are as valid as the samples in the Emomusic dataset. In addition, we believe that using the audio enhancement technique is a good choice, whether considering the generalization of the model or the comparison of the number of the two audio datasets. The audio enhancement technique generates 2 × 744 new music samples in the Emomusic dataset by adding both white noise and time scaling. Therefore, the enhanced dataset contains 3×744 samples. So it is not the Emomusic dataset, nor the DEAM dataset, but a new dataset that can be involved in the comparison, named Augmented dataset. This dataset is involved in the validation of the effectiveness of the Efficient Net, the predictive ability of the ENMI model for long sequences, and the statistics of the training rounds. In order to present the relationship between the three datasets more clearly, a rewrite has been done based on your suggestion: "The Emomusic dataset is a subset of the DEAM dataset. In order to fully evaluate the two datasets and validate the generalization of the model, we added 2×744 audio-enhanced samples to the Emomusic dataset to form a new dataset called Augmented dataset, which is used to make a comparison with the two base datasets". I hope this modification will make the logic clearer. If there are any other problems with my narrative, I am always open to your pointing out and criticizing. (P.16-Preprocessing)

7- **Subsection "Preprocessing": the sentence "To ensure that the addition of white Gaussian noise ..." seems to be incomplete and should therefore be revised.**

Response：I apologize for the annoyance caused to you by the "section on adding white noise" in the subsection "Preprocessing". As you said, this section is incomplete and difficult to understand. We consider that the sound intensity of white noise should be strictly controlled. Too much white noise, even to the extent of drowning out the original audio, will seriously affect the musical character, and a musical character that differs too much from the original audio will indirectly contaminate the label, which defeats the purpose of data enhancement. Therefore, we have modified the original text according to your suggestion. The modification reads: "In order to ensure a better data enhancement effect and avoid the contamination of audio data labels by high-intensity white noise, we control the intensity of white noise addition to avoid the contamination of audio data labels. We have controlled the intensity of white noise added in a reasonable range. The intensity of the added white noise is related to the intensity of the original audio as follows:..." (P.17-Para.3)

8- **Subsection "Feature extraction": the description of the features that were manually extracted from the audio time series should be moved to the "Model description" Section in my opinion, as it is part of the methodological description according to Figure 3.**

Response：Thank you for your question about the structure of the article, and we fully agree with your suggestion. Previously, the article was organized according to the data processing process, specifically: data preprocessing - data feature extraction - data-driven modeling. This seems to be an irrational way of organizing the article. As you said, feature extraction is an important part of Figure 3. For modeling, the first step should be feature extraction, then feature fusion, and finally dynamic sentiment prediction and regression. Obviously, it is more appropriate to move the "Feature Extraction" section to the "Model Description" section. We have changed the location of the "Feature Extraction" section and changed the order in which the references are cited. Thanks again for pointing out the problem. (P.9-P.10)

9- **The "Discussion" Section could be renamed "Experimental results" as it contains the main results of the study as well as the ablation studies.**

Response：Thanks to your valuable suggestion, we have renamed the "Discussion" section as " Experimental results". This change makes it easier for readers to grasp the content of the manuscript, and the precise wording makes the article more scientific and rational. We will pay more attention to the generalization of the headings in future writing. (P.22- Experimental results)
