# Supplementary material for: Music-induced emotion flow modeling by ENMI Network
Source: PLoS One. 2024 Oct 21;19(10):e0297712. doi: 10.1371/journal.pone.0297712 (PMC11493256; doi:10.1371/journal.pone.0297712)
Supplement: Attachment — Submitted filename: Response to Reviewers.docx [file pone.0297712.s008.docx]

**Dear Dr. Li,**

**Thank you for your approval of our revised manuscript in the last round of review.**

**After receiving your comments, we made further adjustments to the areas that needed improvement. To make sure there are no careless mistakes left in the paper, we asked a professional editor to polish it. We hope that in the next round of peer review, our manuscript will meet the standards for acceptance and publication.**

**We appreciate your continued attention and review of our manuscript. We have learned a lot from this process. Please feel free to contact us with any additional questions regarding the following revisions.**

**Yours sincerely**

**Lead author: Yunrui Shang**

**Corresponding author: Yinhua Liu**

**Comment #1- Wording of the first paper contribution:
The authors have disambiguated the description of the first contribution, but its wording now seems to be somewhat generic to me. I would recommend to more specifically state that the contribution consists in** **modelling emotion flow recognition as a regression problem using time series.**

**Response：Thank you for your valuable comment. We are fully aware of the fact that the description of the first contribution of the revised version is still not clear enough and the language is not sufficiently cohesive and concise. As you said, the description of the first contribution should be biased towards the solution of the emotion flow problem and should not describe too much music-related content. We have learnt from your comment and reworked the summary of the first contribution. The revision is as follows:** **Emotions change under the influence of music in the dimension of time. So we model emotion flow recognition as a regression problem using time series. We hope that such a modification is more accurate.**

**Comment #3 - Equation (2):
It seems the authors are using A and V as subscript for arousal and valence respectively. The notation $g_A$ to designate the emotion change according to the valence dimension is therefore not so clear. Maybe $g_V$ would be more appropriate.**

**Response：Thank you for pointing out a very obvious error in the presentation of the formula. We have corrected the incorrect subscripts in the formula, and at the same time, the relevant textual presentation has been reworked. Thank you again for your careful examination and review. Specific changes have been marked in the manuscript.**

**Comment #5 - song ID:
The authors' answer for this point was not completely clear to me on some aspects. For instance, I am unsure of what "data splicing different songs by the first and last of the time dimension" refers to. Furthermore, from my understanding, a timestamp of the format "ID - second" was used as input of the time embedding layer in the carried out experiments. Because this information may not be available for all songs, the authors seem to indicate in their answer that their proposed approach can be applied only on data following a very specific format. This seems to be a strong limitation to me, that should a minima be discussed in the "Conclusion and future work" section.**

**Response：Thank you for your comment. We apologize that we still haven't clarified the meaning of the “ID-second” format timestamp in our last reply. We would like to clarify again the issue you raised:**

**In the current dataset of dynamic music emotion recognition, DEAM contains 1744 music samples of 15s-45s and Emomusic contains 744 music samples of 15s-45s. We need to articulate the data when inputting the data into the model, e.g., the last sample point of song ID=1 is articulated with the first sample point of song ID=2, and so on, to form a long time series. However, we know that the articulation of a long sequence after articulation is very unfavorable for time series prediction. This is because the two pieces of music before and after the articulation may have different musical styles or expressions, in other words, the correlation between two different pieces of music is much weaker than the correlation within one piece of music. Therefore, by adding the “ID-second” format timestamp, we are telling the model to pay attention to this.**

**On reflection, we agree with the limitations of timestamps that you mentioned in the first round of reviews, and have therefore removed the description of timestamps. This is because this timestamp format can really only be used for specific data. In practical engineering, it is not possible to guarantee that the duration of each music is the same. So, following your guidance, we will explain the problem in the Conclusion and future work section and try to find strategies to overcome this problem in our future research.**

**Thank you again for your review and guidance.**

**Comment #6 - dataset description:
From my understanding of the authors' response, the "Augmented dataset" consists of music samples from the Emomusic dataset, that were corrupted with white noise and scaled to create augmented samples in order to compensate for the lower sample size of Emomusic compared to DEAM. In my opinion, presenting these augmented samples as a third dataset may lead to confusion, because it was jointly used with Emomusic for the carried out experiments. It would be better to mention in the manuscript that the Emomusic dataset was augmented with the techniques described in Section "Processing" to account for its lower size compared to DEAM.**

**Response：I apologize that the description of the Augmented dataset in the manuscript remains confused and vague. Your understanding in the comment is correct. The Augmented dataset consists of three parts: the original Emomusic dataset (744 samples), the Gaussian white noise augmented data (744 samples), and the time-stretched augmented data (744 samples). It should be emphasized that the DEAM dataset (1744 samples) contains all the samples in the Emomusic dataset. This is the reason for introducing the Augmented dataset in our work. The relationship between the three datasets is illustrated in the figure below:**

**According to your suggestion, we have updated the first paragraph of the Preprocessing section, in particular to include more specific information about the reasons for introducing the Augmented dataset. The updated content is as follows:**

**In order to comprehensively validate the generalization of the model, we incorporated 2×744 audio-enhanced samples into the Emomusic dataset to form a new dataset called the Augmented dataset. Moreover, all the enhancement data are drawn from the samples in the Emomusic dataset. The particular enhancement techniques used are**

**shown in 5. Both the Augmented dataset and the DEAM dataset contain samples from the Emomusic dataset. Therefore, the effect of the difference in the number of samples between the two original datasets can be compensated through the integration of the Augmented dataset. This approach was similarly employed by Bhuwan Bhattarai in his study [46].**

**We hope that the modifications will make the relationship between the three datasets and the reason for introducing the enhanced dataset clearer. Thank you for your guidance.**

**As a minor remark, I would suggest the authors to perform a last spell/grammar check of the modified parts of the manuscript (e.g. redundant sentences in the added paragraph before Equation 16).**

**Response：Thank you for your meticulous examination of the manuscript. As you mentioned, redundancy was found in the paragraph before Equation 16. We have removed the redundancies and checked the rest of the article. In addition, we have asked a professional teacher to touch up the manuscript and correct problems with word spelling, grammatical structure, and so on. Thank you again for pointing out the problems.**
